# Enhancing the CuAAC efficiency of a Cu(i)–NHC complex in biological media by encapsulation†

**DOI:** 10.1039/d5cc01891a

**Published:** 2025-05-20

**Authors:** A. P. Prakasham, Harshit Singh, Anja R. A. Palmans

**Affiliations:** a Department of Chemical Engineering & Chemistry and Institute for Complex Molecular Systems, Eindhoven University of Technology P.O. Box 513 5600 MB Eindhoven The Netherlands a.palmans@tue.nl a.p.prakasham@tue.nl

## Abstract

A novel [(NHC)_2_Cu]Br (Cu(i)–NHC) complex promotes CuAAC reactions in organic solvents, water, buffers and even complex biological media in good yields. Encapsulation of the Cu catalyst in an amphiphilic polymer enhances its performance in complex media, increases catalyst stability in the presence of glutathione and reduces cytotoxicity in HeLa cells.

## Abstract 2

A stable Cu(i)–NHC catalyzes CuAAC in organic solvents, water, buffers and DMEM; its encapsulation in an amphiphilic polymer improves the catalytic activity in complex media, enhances the stability in glutathione and reduces toxicity in HeLa cells.

## Graphical abstract

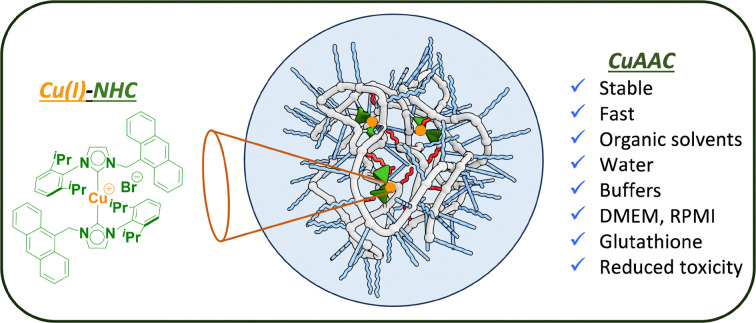

Copper(i) catalysed azide–alkyne cycloaddition (CuAAC) has found widespread application in the fields of organic synthesis,^1^ glycoscience,^2^ polymers,^3^ and functional (bio)macromolecules.^4^ CuAAC is one of the best C–N bond forming reactions for the selective covalent functionalization of peptides, proteins and other biomacromolecules and has been evaluated in drug delivery,^5^ in bioorthogonal chemistry,^6^ and for various other biological applications,^7,8^ resulting in a chemistry Nobel prize in 2022.^9–11^ Despite its high appeal and convenience, CuAAC still possesses limitations such as the need for excess reducing agent (Na-ascorbate) to reduce Cu(ii) to Cu(i), and for Cu(i) stabilising ligands to enhance reactions rates.^12–14^ CuAAC reactions work well in neat/aqueous media,^15–18^ but the performance of many Cu(i) complexes in complex and cellular media is poor and their toxicity is high. Despite these challenges, bio-orthogonal reactivity has been achieved for Cu(i)-based systems using triazole-based ligands for CuAAC and BTTAA ligands for NH insertion reactions.^19–21^

Recently, the addition of Cu(i) with N-heterocyclic carbene (NHC) ligands attached to polymeric carriers showed good biocompatibility and allowed the generation of hydroxyl radicals in *in vitro* and *in vivo* conditions.^22^ The NHC ligand is a perfect candidate for stabilizing the Cu(i) ion in CuAAC chemistry through its strong σ-electron donation capability and steric bulk, thereby overcoming the oxygen sensitivity of Cu(i) species to form Cu(ii). While efficient [(NHC)_2_Cu]X type catalysts have been reported for CuAAC reactions,^23^ many Cu–NHC complexes require either stringent conditions (absence of air/moisture) for the synthesis of the catalysts^24,25^ and/or need to be activated by chemical,^26^ thermal,^27,28^ photochemical^29^ or mechanical means^30,31^ to yield 1,2,3-triazoles (Table S3, ESI†). Given the high activity of Cu(i)–NHC found in CuAAC reactions in organic solvents, we here set out to develop Cu(i)–NHC complexes for CuAAC reactions in water and in complex cellular media. In this work, we report the synthesis of a new Cu(i)–NHC complex, assess its catalytic activity in a variety of media and in the presence of glutathione (GSH), and investigate its compatibility with cells.

In order to stabilize the Cu(i) state, a bulky NHC ligand was chosen having a steric 2,6-(^i^Pr)_2_-C_6_H_3_ group and an anthracene pendant substitution on the N-atoms. The NHC ligand precursor (1) was synthesised by the *N*-alkylation of 1-(2,6-diisopropylphenyl)-imidazole with 9-(bromomethyl) anthracene (Fig. S1–S5, ESI†). Subsequent reaction of (1) with CuBr in the presence of K_2_CO_3_ in acetone afforded the Cu(i)–NHC complex (2) in quantitative yield as an off-white solid (Scheme 1). Noticeably, the Cu(i) complex (2) is stable towards air and moisture. The coordination of the NHC ligand to the Cu was verified by the disappearance of the NC*H̲*N resonance and the formation of the bis-NHC complex [(NHC)_2_Cu]Br was corroborated by the *m*/*z* peak at 899.40 that corresponds to the [M–Br]^+^ molecular ion of the complex [C_60_H_60_CuN_4_]^+^ through MALDI-ToF-MS, in line with structurally similar complexes (Fig. S6–S10, ESI†).^32^ The Cu(i)–NHC complex (2) showed distinct absorption bands around 300–425 nm and weak fluorescence emissions around *ca.* 375–500 nm characteristics of anthracene transitions in solution as well as in the solid state (Fig. S11 and S12, ESI†).

**Scheme 1:**
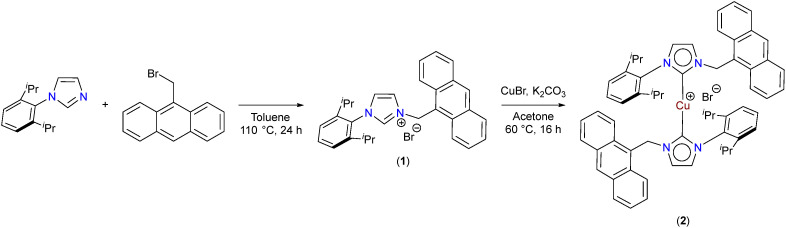
Synthesis of Cu(i)–NHC complex (2).

With the perspective of utilizing the Cu(i)–NHC in bioconjugation reactions, we here set out to test benzyl azide and phenylacetylene as model substrates in the CuAAC reaction using 0.5–2.5 mol% Cu(i)–NHC (2) to yield 1,4-substituted 1,2,3-triazole (3a) (Table S1, ESI†). The reaction was initially performed in water, in which (2) did not dissolve, at 37 °C. With a catalyst loading of 1 mol%, the 1,2,3-triazole (3a) was isolated in 90% yield in 2 h, while lower loading of 0.5 mol% catalyst gave 69% yield (Table S1, entries 1–3, ESI†). The Cu(i)–NHC (2), also gave high yields using a lower temperature of 20 °C, and with reduced reaction times of 1 h and 10 minutes (Table S1, entries 4–6, ESI†). Mixing of the two substrates in neat conditions, without water in the presence of Cu(i)–NHC (2), quantitatively offered 3a. The absence of Cu-complex (2) in the reaction did not give 3a (Table S1, entry 9, ESI†), which highlights the importance of Cu(i) catalysts in the CuAAC reactions. To ascertain uniform conditions in all catalysis reactions, we selected 1 mol% catalyst loading, *T* = 37 °C and 2 h reaction time as the optimum conditions.

With these conditions, we screened the activity of (2) in various solvents (Table 1). Interestingly, Cu(i)–NHC (2) performed well in various organic solvents, namely, the non-polar toluene to polar dichloromethane (DCM), tetrahydrofuran (THF), acetonitrile (ACN), and also protic ^*t*^BuOH and water:DMSO mixtures (Table 1, entries 1–6). In DMF, in contrast, only 36% of 3a was isolated. Notably, the reaction proceeded in good yield in basic NaOH (0.1 M) solution (92%), and in basic buffers, PBS (90%) and Tris (88%) (Table 1, entries 9–11). Remarkably, the Cu(i)–NHC (2) effectively catalysed the cycloaddition of benzyl azide and phenylacetylene in biological cell culture media such as RPMI (Roswell Park Memorial Institute 1640) and DMEM (Dulbecco's modified Eagle's medium), affording high yields >80% (Table 1, entries 12–14).

**Table 1:** Solvent scope of CuAAC reaction using Cu(i)–NHC (2)^a^

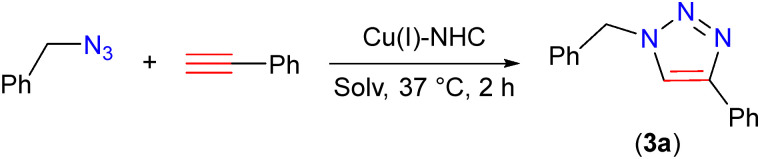			
Entry	Catalyst (mol%)	Solvent	Yield^b^ (%)
1	1	^*t*^BuOH	99
2	1	THF	86
3	1	DCM	99
4	1	ACN	>99
5	1	Toluene	>99
6	1	H_2_O:DMSO^c^	98
7	1	DMF	36
8	1	H_2_O	90
9	1	NaOH (0.1 M)	92
10	1	PBS	90
11	1	Tris (0.5 M)	88
12	1	RPMI	83
13	1	DMEM	81
14	2.5	DMEM	97

a Conditions: azide (0.25 mmol), alkyne (0.3 mmol), solvent (0.5 mL), 37 °C, 2 h.

b Isolated yield.

c H_2_O : DMSO (9 : 1).

We further assessed the functional group tolerance by screening a wide variety of substrates in the CuAAC reaction using Cu(i)–NHC (2). For this purpose, various substituted alkynes were reacted with benzyl azide (eqn (1)) and azidomethyl pivalate (eqn (2)) in water at 37 °C (Table 2). As was evident from the substrate scope, the electron withdrawing or donating substituents on the phenylacetylene did not show significant differences in yield (all above 87%). Also, both azides gave good yields of the respective 1,2,3-triazoles (for example, 4-Br (3b) *vs.* 4-OMe (3i) entries 2 and 9). Interestingly, alkynes bearing phenol (3e), carboxylic acid (3f) and aniline (3g) also gave good yields of the corresponding 1,2,3-triazole products. Moreover, both benzylic and aliphatic azide gave good to excellent isolated yields of 1,2,3-triazoles 3(a–j) and 4(a–h). Interestingly, both liquid as well as solid substrates (in the case of 3b, 3(f–g), 3j, 3(m–n), 4e, 5(a–b), and 6(a–c)) resulted in high isolated yields, which is remarkable in view of the catalyst being a solid in water.

**Table 2:** Substrate scope of CuAAC reaction using Cu(i)–NHC (2)^a^

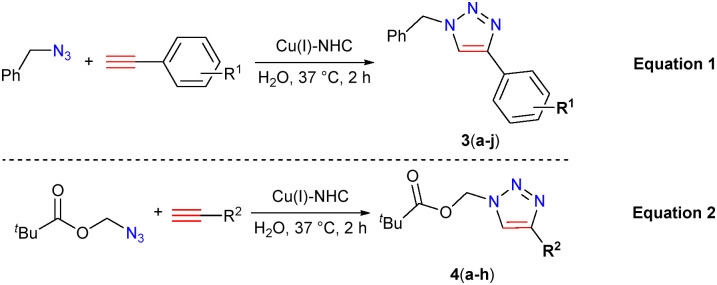				
S. no.	3(a–j) R^1^ =	Yield^b^ (%)	4(a–h) R^2^ =	Yield^b^ (%)
1	H (3a)	90	Ph (4a)	82
2	4-Br (3b)	94	3,5-F_2_-C_6_H_3_ (4b)	92
3	3,5-F_2_ (3c)	96	3-Me-C_6_H_4_ (4c)	96
4	3,5-(CF_3_)_2_ (3d)	87	4-OMe-C_6_H_4_ (4d)	90
5	3-OH (3e)	92	3,5-(OMe)_2_-C_6_H_3_ (4e)	90
6	3-COOH (3f)	94	CH_2_Ph (4f)	94
7	4-NH_2_ (3g)	90	CH_2_OPh (4g)	96
8	3-Me (3h)	90	CH(OEt)_2_ (4h)	93
9	4-OMe (3i)	92		
10	3,5-(OMe)_2_ (3j)	88		

a Azide (0.25 mmol), alkyne (0.3 mmol), Cu(i)–NHC 1 mol%, H_2_O (0.5 mL), 37 °C, 2 h.

b Isolated yield.

The scope of functional group tolerance was further assessed in Table 3. The reaction with a bis-alkyne and a highly functional 5-bromo-3-ethynylpyrazin-2-amine afforded the respective triazoles (3m; 82%) and (3n; 81%) in good yields. Cu(i)–NHC (2) promoted the cycloaddition of benzyl azide with aliphatic alkynes having a free alcohol (3o), acid (3p), NHBoc (3q), Si(^i^Pr)_3_ (3r) or ether (3t) substituent in good yield. Notably, the formation of 3q, from *N*-Boc-propargylamine in DMEM proceeded smoothly using 2. We further extended the applicability of the Cu(i)–NHC complex (2) in the synthesis of galactose derived triazoles 5(a–b) and the anthracene derived fluorescent triazoles 6(a–c) in good yields. Reaction of phenyl azide and benzylacetylene provided the expected product in 86% yield (Table 3). Most importantly, the purification of the end products mostly involved a simple extraction using EtOAc or Et_2_O, followed by washing with Et_2_O or cold pentane, respectively, to remove traces of alkynes and to get the clean triazole products. In a few cases, a simple filtration type column was used to obtain pure triazoles.

**Table 3:** Substrate scope of CuAAC reaction using Cu(i)–NHC (2)^a^

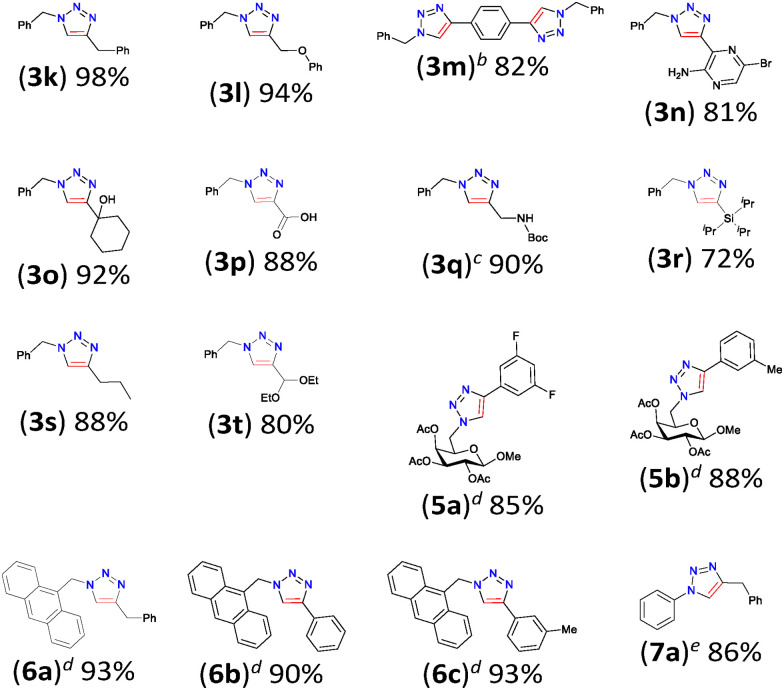

a Azide (0.25 mmol), alkyne (0.3 mmol), Cu(i)–NHC 1 mol%, H_2_O (0.5 mL), 37 °C, 2 h, isolated yield.

b Azide (0.625 mmol), alkyne (0.25 mmol), Cu(i)–NHC 2 mol%.

c In DMEM 89% yield at 2.5 mol% catalyst.

d Azide (0.125 mmol), alkyne (0.15 mmol), (2) 1 mol%.

e Rection performed in 2-Me-THF.

All liquid substrates (azide and alkyne) underwent smooth conversion to product triazoles in both water and DMEM. In contrast, the solid substrates resulted in full conversion to product only in water, but not in biological media, as seen for 9-(azidomethyl)anthracene and phenylacetylene that gave only ≈43% and 57% conversion to the product (6b) in DMEM and RPMI, respectively (Fig. S131, ESI†). Remarkably, the addition of an amphiphilic heterograft polymer (8) containing randomly distributed hydrophilic and hydrophobic grafts,^33–35^ aided the reaction in DMEM to reach completion in 2 h at 37 °C. This is a result of the hydrophobic interior formed after collapse of the polymer in aqueous media, which increases the local concentration of the reaction components in the media through a micellar-type encapsulation (Fig. 1).

**Fig. 1:**
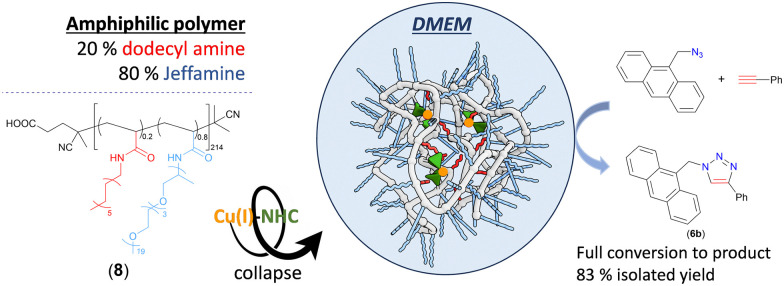
Encapsulation of Cu(i)–NHC (2) in an amphiphilic polymer (8) ensures full conversion of solid substrates in complex media.

The generality of the catalyst was tested in the multicomponent click reaction of halides in the presence of NaN_3_ and phenylacetylene (*in situ* azide formation) using the Cu(i)–NHC (2) (Table S2, ESI†). As anticipated, the reaction of benzyl bromide and benzyl chloride gave triazole in 73% and 41% yields, respectively, at 37 °C in 2 h. Overnight stirring provided complete conversion to the product (Table S2, ESI†).

We also tested the applicability of the Cu(i)–NHC (2) in the presence of glutathione (GSH), a biologically abundant (up to 10 mM) thiol nucleophile in cells that poison metals.^36,37^ Surprisingly, 2 catalysed the formation of 3a in the presence of 10 mM concentration of GSH, while it gave no product at 15 mM GSH. Remarkably, almost full activity of the Cu(i)–NHC (2) was retained when encapsulated in the amphiphilic polymer (8) when 15 mM GSH was present in the reaction mixture. Likely, the hydrophobic micro-environment prevents the GSH inhibition of Cu (Table 4). These interesting results prompted us to check the cell viability of Cu(i)–NHC (2) in the presence of HeLa cells. Using a CCK8 assay showed that Cu(i)–NHC (2) encapsulated in the polymer (8) was not toxic up to 24 h and up to an 18 μM concentration (Fig S13, ESI†). In contrast, Cu(i)–NHC (2) alone was toxic starting from 3 μM, highlighting that encapsulation safeguarded the Cu(i) catalyst in the hydrophobic interior of the polymer, thus reducing Cu(i) toxicity. The analysis of the leached-out copper from the polymer using ICP-OES showed a remarkable encapsulation efficiency of 99% (Fig. S129, ESI†).

**Table 4:** CuAAC reaction in the presence of glutathione (GSH)^a^

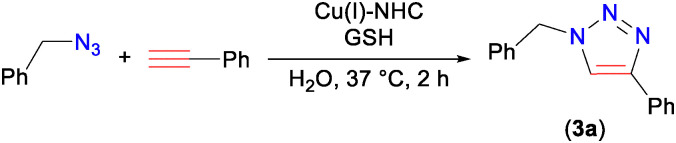				
Entry	Cu(i)–NHC (2) (mol%)	GSH^e^ (equiv.)	Polymer	Yield^b^ (%)
1	1^c^	1	—	80
2	1^d^	1	—	81
3	1^d^	2	—	83
4	1^d^	3	—	n.d.^f^
5	1%^d^	3	Yes	75

a Conditions: azide (0.25 mmol), alkyne (0.3 mmol), Cu(i)–NHC 1 mol%, H_2_O (0.5 mL).

b Isolated yield.

c GSH added after Cu(i).

d GSH added before Cu(i).

e With respect to Cu(i). 2 equiv. GSH = 10 mM.

f Not detected. Polymer 1.25 μmol (1 : 20 : 2000, polymer : catalyst : substrate).

In summary, the novel Cu(i)–NHC complex efficiently catalyzed CuAAC reactions for a wide range of azides and alkynes in organic and aqueous solvents. Importantly, high conversions are obtained in complex biological media. The use of an amphiphilic polymer accelerated the CuAAC reaction in complex media for solid substrates. Moreover, the polymer encapsulated Cu(i)–NHC performed very well even in the presence of 15 mM glutathione and was less toxic to HeLa cells. Currently, the application of Cu(i)–NHC in the synthesis and modification of complex (bio)macromolecules is underway in our laboratory.

The project has received funding from the European Union's Horizon 2020 research and innovation programme under the Marie Skłodowska-Curie grant agreement no. 899987. We gratefully acknowledge support by The Dutch Research Council (NWO Gravitation; IPM program), the TU/e and Peter Lipman for ICP-OES analysis.

## Conflicts of interest

There are no conflicts to declare.

## Supplementary Material

CC-061-D5CC01891A-s001

## Data Availability

The data supporting this article have been included as part of the ESI.†

## References

[cit1] Jaiswal M. K., Tiwari V. K. (2023). Chem. Rec..

[cit2] Agrahari A. K., Bose P., Jaiswal M. K., Rajkhowa S., Singh A. S., Hotha S., Mishra N., Tiwari V. K. (2021). Chem. Rev..

[cit3] Ali M. F., Ochiai B. (2024). Polymers.

[cit4] Leier S., Wuest F. (2024). Pharmaceuticals.

[cit5] Saraiva N. M., Alves A., Costa P. C., Correia-da-Silva M. (2024). Pharmaceuticals.

[cit6] Fu Y., Zhang X., Wu L., Wu M., James T. D., Zhang R. (2025). Chem. Soc. Rev..

[cit7] Heble A. Y., Chen C.-L. (2024). Biomacromolecules.

[cit8] Khandelwal R., Vasava M., Abhirami R. B., Karsharma M. (2024). Bioorg. Med. Chem. Lett..

[cit9] Taiariol L., Chaix C., Farre C., Moreau E. (2022). Chem. Rev..

[cit10] Tornøe C. W., Christensen C., Meldal M. (2002). J. Org. Chem..

[cit11] Rostovtsev V. V., Green L. G., Fokin V. V., Sharpless K. B. (2002). Angew. Chem., Int. Ed..

[cit12] Szkółka A., Szafrański P. W., Kasza P., Talik P., Krośniak M., Cegła M., Zajdel P. (2024). Org. Process Res. Dev..

[cit13] Yang M., Jalloh A. S., Wei W., Zhao J., Wu P., Chen P. R. (2014). Nat. Commun..

[cit14] Rudolf G. C., Sieber S. A. (2013). ChemBioChem.

[cit15] Aflak N., Essebbar F.-E., Bahsis L., Ben El Ayouchia H., Anane H., Julve M., Stiriba S.-E. (2024). RSC Sustainable.

[cit16] Bahsis L., Ablouh E.-H., Hanani Z., Sehaqui H., El Achaby M., Julve M., Stiriba S.-E. (2024). Carbohydr. Polym..

[cit17] Díez-González S., Correa A., Cavallo L., Nolan S. P. (2006). Chem. – Eur. J..

[cit18] Díez-González S., Escudero-Adán E. C., Benet-Buchholz J., Stevens E. D., Slawin A. M. Z., Nolan S. P. (2010). Dalton Trans..

[cit19] Gutiérrez S., Tomás-Gamasa M., Mascareñas J. L. (2021). Angew. Chem., Int. Ed..

[cit20] Miguel-Ávila J., Tomás-Gamasa M., Olmos A., Pérez P. J., Mascareñas J. L. (2018). Chem. Sci..

[cit21] Bai Y., Feng X., Xing H., Xu Y., Kim B. K., Baig N., Zhou T., Gewirth A. A., Lu Y., Oldfield E., Zimmerman S. C. (2016). J. Am. Chem. Soc..

[cit22] Zheng D., Tao J., Jiang L., Zhang X., He H., Shen X., Sang Y., Liu Y., Yang Z., Nie Z. (2025). J. Am. Chem. Soc..

[cit23] Díez-González S., Nolan S. P. (2008). Angew. Chem., Int. Ed..

[cit24] Yagmurlu A., Buğday N., Yaşar Ş., Boulebd H., Mansour L., Koko W. S., Hamdi N., Yaşar S. (2024). Appl. Organomet. Chem..

[cit25] González-Lainez M., Gallegos M., Munarriz J., Azpiroz R., Passarelli V., Jiménez M. V., Pérez-Torrente J. J. (2022). Organometallics.

[cit26] Kim H., Kim H., Kim K., Lee E. (2021). Inorg. Chem..

[cit27] Riethmann M., Föhrenbacher S. A., Keiling H., Ignat’ev N. V., Finze M., Radius U. (2024). Inorg. Chem..

[cit28] Díez-González S., Stevens E. D., Nolan S. P. (2008). Chem. Commun..

[cit29] Islam Sk A., Ghosh A., Kundu K., Murugan I., Kundu P. K. (2024). Chem. – Eur. J..

[cit30] Shinde K. S., Michael P., Fuhrmann D., Binder W. H. (2023). Macromol. Chem. Phys..

[cit31] Biewend M., Neumann S., Michael P., Binder W. H. (2019). Polym. Chem..

[cit32] Santoro O., Lazreg F., Cordes D. B., Slawin A. M. Z., Cazin C. S. J. (2016). Dalton Trans..

[cit33] Artar M., Souren E. R. J., Terashima T., Meijer E. W., Palmans A. R. A. (2015). ACS Macro Lett..

[cit34] Sathyan A., Croke S., Pérez-López A. M., de Waal B. F. M., Unciti-Broceta A., Palmans A. R. A. (2022). Mol. Syst. Des. Eng..

[cit35] Sathyan A., Loman T., Deng L., Palmans A. R. A. (2023). Nanoscale.

[cit36] Wilson Y. M., Dürrenberger M., Nogueira E. S., Ward T. R. (2014). J. Am. Chem. Soc..

[cit37] Zhao H., Liu Z., Wei Y., Zhang L., Wang Z., Ren J., Qu X. (2022). ACS Nano.

