# Supplementary material for: Enhancing the CuAAC efficiency of a Cu(i)–NHC complex in biological media by encapsulation
Source: Chem Commun (Camb). 2025 May 20;61(53):9697–700. doi: 10.1039/d5cc01891a (PMC12138323; doi:10.1039/d5cc01891a)
Supplement: CC-061-D5CC01891A-s001 [file CC-061-D5CC01891A-s001.pdf]

Supplementary Information (SI) for ChemComm.
This journal is © The Royal Society of Chemistry 2025

Supplementary Information

Enhancing the CuAAC efficiency of a Cu(I)-NHC complex in biological media by encapsulation‡

A. P. Prakasham,[a,*] Harshit Singh[a] and Anja R. A. Palmans[a,*]

[a] Department of Chemical Engineering & Chemistry and Institute for Complex Molecular Systems, Eindhoven University of Technology, P.O. Box 513, 5600 MB Eindhoven, The Netherlands. * Corresponding author, E-mail: a.palmans@tue.nl, a.p.prakasham@tue.nl

Table of Contents

1	General information	S3
2	Experimental section	S4
	2.1 Synthesis of pre-NHC ligand (**1**)	S4
	2.2 Synthesis of Cu(I)-NHC complex (**2**)	S4
	2.3 Synthesis amphiphilic polymer (**8**)	S5
	2.4 Encapsulation of Cu(I)-NHC (**2**) in polymer (**8**)	S6
	**Table S1**. Screening of the CuAAC reaction conditions	S7
	**Table S2**. Multicomponent click reaction	S7
	**Table S3**. Comparison of Cu(I)-NHC catalysts in the CuAAC for the synthesis of (**3a**)	S8
	**Table S4**. Toxicity of Cu(I) catalysts used for the CuAAC in live cells	S11
	2.5 General procedure for the click reactions	S14
	2.5.1 General procedures (A, B, C)	S14
	2.5.2 Click reaction in presence of polymer (**8**) in DMEM	S14
	2.5.3 Click reaction in presence of glutathione (GSH)	S14
	2.5.4 General procedure for the multicomponent click reaction	S14
	2.5.5 Reusability of Cu(I)-NHC (**2**) complex	S15
	2.6 Encapsulation efficiency study	S15
	1,2,3-triazoles **3**(**a**-**t**), **4**(**a**-**h**), **5**(**a**-**b**), **6**(**a**-**c**) and **7a**	S15-S26
	2.4 Cell viability study	S26
3	Characterization data	S27
	Data for pre-NHC ligand (**1**)	S27-S29
	Data for Cu(I)-NHC complex (**2**)	S29-S31
	UV-Vis and fluorescence data of Cu(I)-NHC complex (**2**)	S32
	Cell viability data for Cu(I)-NHC complex (**2**)	S33
	Data for 1,2,3-triazoles **3**(**a**-**t**), **4**(**a**-**h**), **5**(**a**-**b**), **6**(**a**-**c**) and **7a**	S33-S90
	Encapsulation efficiency ICP-OES data	S91
	CuAAC in complex media	S92
	Polymer data	S93-S96
	Cu(I) stabilizing ligands	S97
4	References	S98-S100

‡ Azide containing reactions were performed in safe and permissible quantity as copious amounts might lead to explosion. Aqueous solutions containing azide were disposed of in alkaline solution (pH > 9) to prevent the formation of HN_3.

1. General information

The synthesis of pre-NHC ligand (**1**) and Cu(I)-NHC complex (**2**) were performed in open air avoiding stringent conditions. All the azide containing reactions were performed in safe and permissible quantity as copious amount might lead to explosion. Any aqueous solutions containing azide were carefully disposed off in alkaline solution (pH>9) to prevent the formation of HN_3. All chemicals were purchased from Merck, Thermo Fisher Scientific, Alfa Aesar, Aaron chemicals and used without further purification. Deuterated solvents ($CDCl_3$ from Cambridge Isotope Laboratories, DMSO-d_6 and CD_3OD from Deutero GmbH) were purchased and used as received. Cell Counting Kit-8 (CCK-8) kit was obtained from Sigma-Aldrich and used directly. The molecular weight cut-off of 6-8 kDa dialysis membrane was purchased from Spectra/Por®. 1-(2,6-Diisopropylphenyl)-1H-imidazole,[1] β-D-galactopyranoside, methyl 6-deoxy-6-azido,2,3,4-triacetate,[2] 9-(azidomethyl)anthracene,[3] and the amphiphilic 80 % Jeffamine®M1000 and 20 % dodecyl amine) polymer[4] were synthesised using a modified procedure from the literature. NMR spectra were recorded on Bruker Avance III 400 MHz spectrometer. ^1H and $^{13}C\{^1H\}$ NMR signals are reported in ppm downfield from TMS. ^1H signals are referenced to the residual proton of a deuterated solvent 7.26 ppm for $CDCl_3$, 2.50 ppm for DMSO-d_6, and 3.31 ppm for CD_3OD. $^{13}C\{^1H\}$ signals are referenced to the solvent signal at 77.16 ppm for $CDCl_3$, 39.52 ppm for DMSO-d_6, and 49.00 ppm for CD_3OD. ^1H NMR peaks are labelled as singlet (s), doublet (d), triplet (t), broad (b), quartet (q), septet (sept), doublet of triplets (dt), triplet of doublets (td), doublet of doublets (dd), triplet of triplets (tt) and multiplet (m). Matrix-assisted laser desorption/ionization time-of-flight followed by mass spectrometry (MALDI-ToF-MS) measurements were performed on a Bruker Autoflex Speed using α-cyano-4-hydroxycinnamic acid (CHCA) and trans-2-[3-(4-tert-butylphenyl)-2-methyl-2- propenylidene]malononitrile (DCTB) as matrices. LCMS (ESI) measurements were performed on LTQ XL linear ion trap Thermo Fisher Scientific mass spectrometer. UV-Vis measurements were performed on Agilent cary 3500 multicell UV-Vis spectrophotometer using 1 cm pathlength quartz cuvettes. Fluorescence measurements were performed on Agilent cary eclipse fluorescence spectrophotometer using 1 cm × 1 cm pathlength quartz cuvettes. Size-exclusion chromatography (SEC) measurements in THF were carried out on a Shimadzu prominence-I LC-2030C 3D system equipped with a Shimadzu RI detector and a LC-2030/2040 PDA detector on a PLgel 5 μm mixed-C (200-2000 kDa) and PLgel 5 μm mixed-D (200-40 kDa) columns combined in series (exclusion limit = 2000 kDa, i.d. = 7.5 mm, L = 300 mm), with THF as eluent at a constant flow rate of 1 mL/min at 40 °C. The column was calibrated against polystyrene standard (Polymer Laboratories). Cell viability visualization absorbance was recorded at Tecan SPARK microplate reader. Inductively coupled plasma-optical emission spectrometry (ICP-OES) was performed on thermo scientific icap pro X instrument.

2. Experimental section

2.1 Synthesis of pre-NHC ligand (1)

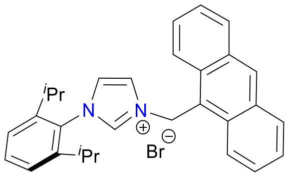

(1)

A mixture of 1-(2,6-diisopropylphenyl)-1H-imidazole (0.750 g, 3.28 mmol) and 9-(bromomethyl)anthracene (0.891 g, 3.28 mmol) in toluene (*ca*. 25 mL) was heated at 110 °C for 24 h in a 100 mL round bottom flask fitted with a water flow condenser. The reaction mixture was cooled down to room temperature and toluene was evaporated under reduced pressure. The obtained brown residue washed with Et_2O (3 × 25 mL) to obtain the pre-NHC ligand, 3-(anthracen-9-ylmethyl)-1-(2,6-diisopropylphenyl)-imidazolium bromide (**1**) as light brown solid (1.465 g) in 89% yield. ^{1}H NMR (400 MHz, $CDCl_3$, 25 ˚C, δ ppm): 10.63 (s, 1H, NC*H*N), 8.61 (s, 1H), 8.51 (d, *J* = 8.0 Hz, 2H), 8.09 (d, *J* = 8.0 Hz, 2H), 7.67 (t, *J* = 8.0 Hz, 2H), 7.55 (t, *J* = 8.0 Hz, 2H), 7.50 (t, *J* = 8.0 Hz, 1H), 7.39 (t, *J* = 4.0 Hz, 1H), 7.27-7.25 (m, 2H), 7.10 (s, 2H), 6.95 (t, *J* = 4.0 Hz, 1H), 2.20 (sept, *J* = 8.0 Hz, 2H, {C*H*(CH_3)$_2$}$_2$), 1.19 (d, *J* = 8.0 Hz, 6H, {CH(C*H*$_3$)$_2$}$_2$), 1.06 (d, *J* = 8.0 Hz, 6H, {CH(C*H*$_3$)$_2$}$_2$). ^{13}C{^{1}H} NMR (100 MHz, DMSO-*d*$_6$, 25 ˚C, δ ppm): 144.89, 137.86, 131.31, 131.16, 130.63, 130.50, 130.22, 129.48, 127.63, 125.54, 125.30, 124.24, 123.41, 123.31, 123.15, 45.65, 28.03, 23.63, 23.56. MALDI-ToF-MS: *m/z* calcd. for $C_{30}H_{31}N_2^+$: 419.25, found: 419.24 [M–Br]$^+$.

2.2 Synthesis of Cu(I)-NHC complex (2)

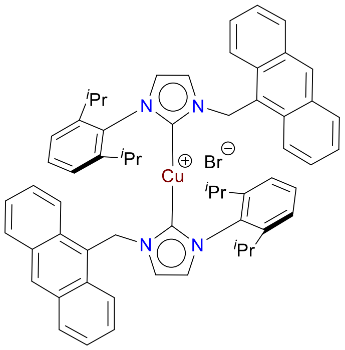

(2)

In a 50 mL round bottom flask, 3-(anthracen-9-ylmethyl)-1-(2,6-diisopropylphenyl)-imidazolium bromide (**1**) (0.250 g, 0.501 mmol), CuBr (0.072 g, 0.501 mmol) and K_2CO_3 (0.138 g, 1.00 mmol) were charged followed by acetone (*ca*. 20 mL). The mixture was refluxed at 60 °C for 16 h. After the completion of the reaction, the reaction mixture was cooled to room temperature and the insolubles were filtered off using celite, and further washed with DCM (3 × 25 mL). The combined organic solvents were evaporated in a rotary evaporator. The obtained residue was washed with Et_2O (3 × 25 mL) to obtain the Cu(I)-NHC complex (**2**) as light brown solid (0.238 g, 97% yield). ^{1}H NMR (400 MHz, $CDCl_3$, 25 ˚C, δ ppm): 8.61 (s, 1H), 8.44 (d, *J* = 8.0 Hz, 2H), 8.11 (d, *J* = 8.0 Hz, 2H), 7.66-7.62 (m, 2H), 7.56 (t, *J* = 8.0 Hz, 2H), 7.46 (t, *J* = 8.0 Hz, 1H), 7.27-7.25 (m, 2H), 6.70 (d, *J* = 2.0 Hz, 1H), 6.61 (d, *J* = 2.0 Hz, 1H), 6.49 (s, 2H), 2.39 (sept, *J* = 8.0 Hz, 2H, {C*H*(CH_3)$_2$}$_2$), 1.34 (d, *J* = 8.0 Hz, 6H, {CH(C*H*$_3$)$_2$}$_2$), 1.04 (d, *J* = 8.0 Hz, 6H, {CH(C*H*$_3$)$_2$}$_2$). ^{13}C{^{1}H} NMR (100 MHz, $CDCl_3$, 25 ˚C, δ ppm): 179.71, 145.82, 134.56, 131.57, 130.94, 130.54,

130.01, 129.71, 127.69, 125.57, 124.76, 124.26, 123.54, 123.19, 119.82, 47.37, 28.43, 24.96, 24.17. MALDI-ToF-MS: *m/z* calcd. for $C_{60}H_{61}CuN_4^+$: 899.41, found: 899.40 $[M{-}Br]^+$.

2.3 Synthesis amphiphilic polymer (8)

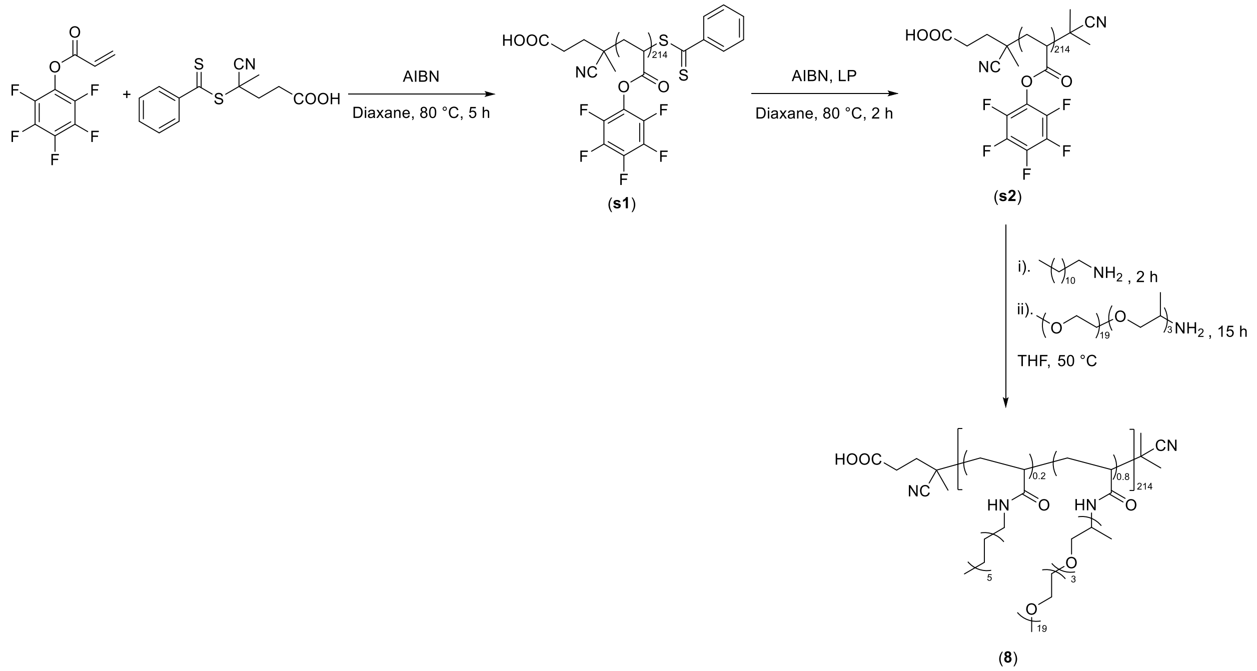

Synthesis poly(pentafluorophenyl) acrylate polymer (s1)

Perfluorophenyl acrylate monomer (3.110 g, 13.061 mmol) was weighed directly in a 25 mL oven dried Schlenk tube. Azobisisobutyronitrile (AIBN) (0.75 mg, 4.57 μmol) and RAFT reagent, 4-cyano-4-((phenylcarbonothioyl)thio)pentanoic acid (12.77 mg, 45.71 μmol) were weighed separately in oven dried HPLC vials using a microbalance. AIBN and RAFT were dissolved in three portions of 0.7 mL dry dioxane and all contents were transferred to the Schlenk tube containing the monomer under Ar atmosphere. Then thorough degassing was performed by bubbling the solution with Ar for 45 min. The reaction mixture was placed in a preheated oil bath at 80 °C under Ar atmosphere and stirred. The monomer conversion to polymer was monitored using ^{19}F NMR and found to be 75 % conversion at 5 h (Fig. S132). The polymerization reaction was quenched by freezing the reaction mixture by placing the Schlenk tube immediately in liquid nitrogen. The pink colour reaction mixture was thawed to ambient temperature by gentle warming in water bath, and purified by precipitating in cold pentane (*ca.* 100 mL). The formed solid was filtered, redissolved in DCM (*ca.* 3 mL) and precipitated again in cold pentane (*ca.* 100 mL) twice. Finally, the pink solid was dried in rotavapor for about an hour to get poly(pentafluorophenyl) acrylate polymer (**s1**) 2.018 g, 87 % yield. The degree of polymerization DP = $[M]_{PFPA}$ / $[M]_{RAFT}$ × 75 % = 13.061 / 0.0457 × 0.75 = 214. The theoretical number-average molecular weight M_n = DP × $M.wt_{(PFPA)}$ + $M.wt_{(RAFT)}$ = 214 × 238.11 + 279.38 = 51235. ^{1}H NMR (400 MHz, $CDCl_3$, 25 ˚C, δ ppm): 3.08 (br), 2.49 (br), 2.11 (br). ^{19}F{^{1}H} NMR (376 MHz, $CDCl_3$, 25 ˚C, δ ppm): −153.23 (br), −156.76 (br), −162.18 (br). Size-exclusion chromatography (SEC) (1 mg / 2 mL THF): PDA detector: M_n = 32.16 kDa, M_w = 42.62 kDa, Đ = 1.32. RI detector: M_n = 32.01 kDa, M_w = 41.93 kDa, Đ = 1.31.

Synthesis end group modified poly(pentafluorophenyl) acrylate polymer (s2)

Polymer (**s1**) (1.991 g, 38.86 μmol), AIBN (0.128 g, 0.78 mmol) and lauroyl peroxide (LP) (0.031 g, 77.76 μmol) were separately weighed in glass vials and were charged in an oven dried Schlenk tube carefully under Ar. The residues in the vials were dissolved in three portions of 3 mL dry dioxane and all contents were transferred to the Schlenk tube under Ar. Then, thorough degassing was performed by bubbling the solution with Ar for 45 min. The reaction mixture was placed in a preheated oil bath sat 80 °C and stirred for 2 h. During the course of the reaction, the pink colour gradually turned colourless. After 2 h, the reaction was quenched by freezing the reaction mixture by placing the tube immediately in liquid nitrogen. The reaction mixture was thawed to ambient temperature by gentle warming in water bath. The reaction volume was reduced to half by evaporating in a rotary evaporator. The obtained viscous solid was purified by precipitating in cold pentane (*ca.* 100 mL). The formed solid was filtered, redissolved in DCM (*ca.* 3 mL) and precipitated again in cold pentane (*ca.* 100 mL) twice. Finally, the colourless solid was dried in rotavapor for about an hour to get end group modified poly(pentafluorophenyl) acrylate polymer (**s2**) 1.661 g, 84 % yield. The theoretical number-average molecular weight $M_n = DP \times M.wt_{(PFPA)} + M.wt_{(End\text{-}group)} = 214 \times 238.11 + 194.23 = 51150$. ^{1}H NMR (400 MHz, $CDCl_3$, 25 ˚C, δ ppm): 3.08 (br), 2.49 (br), 2.11 (br). ^{19}F{^{1}H} NMR (376 MHz, $CDCl_3$, 25 ˚C, δ ppm): −153.22 (br), −156.76 (br), −162.19 (br).

Synthesis amphiphilic polymer (8)

In a 50 mL oven dried Schlenk tube, a mixture of end group removed polymer (**s2**) (0.200 g, 3.91 μmol) and dodecyl amine (20 Equiv, (3.91 × 2.14) × 20 = 167 μmol, 31 mg) in dry THF (*ca.* 5 mL) was stirred at 50 °C under Ar atmosphere. The incorporation of dodecyl amine in the polymer was followed through ^{19}F NMR and was found to 20 % at 2 h (Fig. S138). The degree of functionalization was determined based on the ratio of ^{19}F signal of pentafluorophenol on polymer backbone and free pentafluorophenol. Then, Jeffamine®M1000 (pre-dried at 50 °C over P_2O_5 in a vacuum oven overnight, 180 Equiv, (3.91 × 2.14) × 180 = 1.506 mmol, 1.570 g) added to the reaction mixture and continued heating at 50 °C for overnight, where complete incorporation of Jeffamine®M1000 was observed in the polymer (Fig. S138). After the reaction, the reaction mixture was cooled down to room temperature, and purified by dialysis using molecular weight cut-off of 6-8 kDa dialysis membrane over MeOH (2 × 1 L) and then with THF (3 × 1 L), by changing the solvent every 24 h. The colourless polymer solution in the membrane was transferred in a round-bottom flask and the solvent was evaporated. Thus obtained viscous solid was dissolved in DCM (*ca.* 3 mL) and was added to cold pentane (*ca.* 100 mL) to get a sticky solid, which was collected and dried in a rotary evaporator to get the amphiphilic polymer (**8**) as waxy solid (0.694 g, 90 % yield). ^{1}H NMR (400 MHz, $CDCl_3$, 25 ˚C, δ ppm): 3.83-3.81 (m), 3.66 (br), 3.65 (br), 3.56-3.54 (m), 3.48-3.47 (m), 3.38 (br), 1.26 (br), 1.13 (br).

2.4 General procedure for the encapsulation of Cu(I)-NHC (2) in amphiphilic polymer (8)

Polymer (**8**) and Cu(I)-NHC complex (**2**) were co-dissolved in DCM in a glass vial. DCM was gently evaporated by argon gas flow, which resulted a thin film on the glass vial. The film was dried in a vacuum oven at 40 °C for 1 h. This thin film was dissolved in water by vortex, sonicated for 45 min and left to equilibrate for 30 min.

Table S1. Screening of the CuAAC reaction conditions[a]

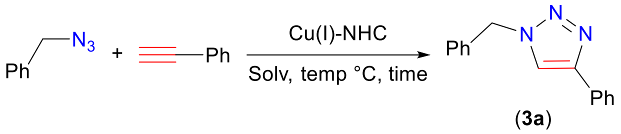

(**3a**)

Entry	Catalyst	Solvent	Temp	Time	Yield[b]
1	2.5 mol %	H_2O	37 °C	2 h	93 %
**2**	**1 mol %**	**H_2O**	**37 °C**	**2 h**	**90 %**
3	0.5 mol %	H_2O	37 °C	2 h	69 %
4	1 mol %	H_2O	20 °C	2 h	93 %
5	1 mol %	H_2O	37 °C	1 h	86 %
6	1 mol %	H_2O	37 °C	10 mins	81 %
7	1 mol %	Neat	37 °C	2 h	98 %
8	1 mol %	Neat	37 °C	10 mins	85 %
9	-	Neat	37 °C	2 h	n.d.[c]

a: Conditions: azide (0.25 mmol), alkyne (0.3 mmol), H_2O (0.5 mL), *b:* Isolated yield. *c:* Not detected.

Table S2. Multicomponent click reaction[a]

Entry	Multi Component Reaction	2 h	12 h
1	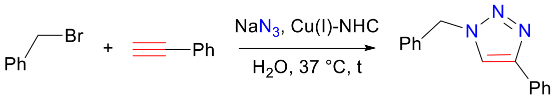 (**3a**)	73 %	93 %
2	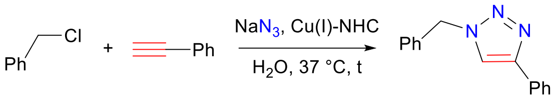 (**3a**)	41 %	81 %

a: Halide (0.25 mmol), NaN_3 (0.31 mmol), alkyne (0.3 mmol), Cu(I)-NHC 1 mol %, H_2O 0.5 mL, 37 °C, isolated yield

Table S3. Comparison of Cu(I)-NHC catalysts in the CuAAC for the synthesis of (**3a**)

**S.No**	**Cu(I)-NHC**	**Solvent**	**T (°C)**	**Time**	**Yield**	**Remarks**	**Ref**
1	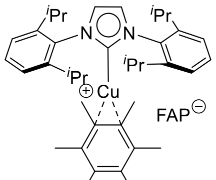	C_6D_6	90 °C	5 h	> 99 %	NMR yield	5
2	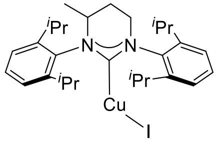	H_2O	25 °C	3 h	90 %	GC yield	6
3	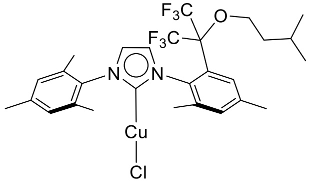	Hexane	40 °C	24 h	99 %	1 mol %	7
4	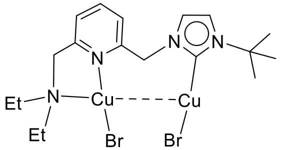	- (neat)	RT	5 min	98 %	Catalysis performed under Ar atmosphere<br>Catalyst in glovebox	8
5	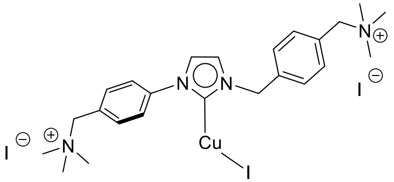	MES buffer (25 % v/v DMSO)	-	3 h	100 % (conversion)	Cucurbit[7]uril (CB[7]) encapsulated Cu(I)-NHC showed no catalysis<br>When Cu(I)-NHC released from (CB[7]) CuAAC worked	9
6	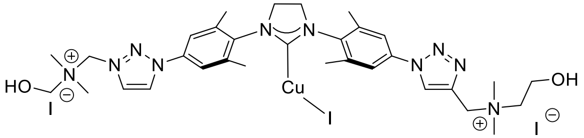	0.2 M HEPES, pH 7.6.	-	18 h	HPLC	Water soluble catalyst<br>For peptide-triazole synthesis	10

7	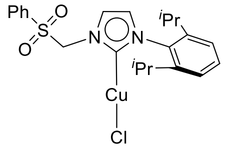	MeOH	RT	1.5	75 %	1 mol %	11
8	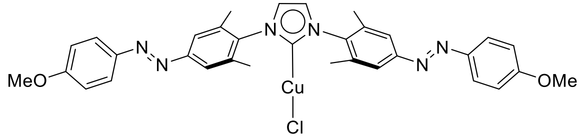	$CDCl_3$	RT	12 h	90 % (By NMR)	In dark For 1-(4-methylbenzyl)-4-phenyl-1,2,3-triazole	12
9	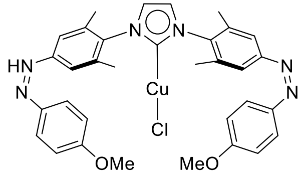	$CDCl_3$	RT	12 h	50 % (By NMR)	Under 365 nm UV light For 1-(4-methylbenzyl)-4-phenyl-1,2,3-triazole	
10	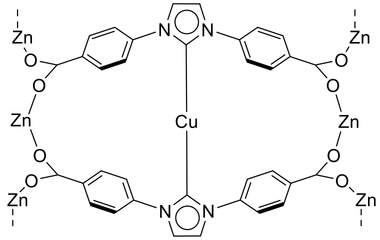	THF-d_8:MeOH (9:1)	-	≈ 15 h	26.5 % (conversion)	By sonication 20 cycles (Each cycle: 5 s Pulse, 10 s Pause for 30 min)	13
11	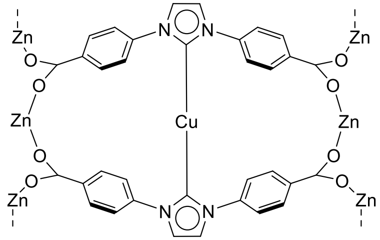	THF-d_8:MeOH (9:1)	60 °C	50 h	10 % (conversion)	Assisted by heat N_2 atmosphere	
12	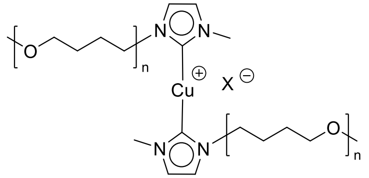	THF-d_8:MeOH (30:1)	-	≈ 25 h	97 % (conversion)	By sonication 10 cycles (Each cycle: 5 s Pulse, 10 s Pause for 60 min)	14

13	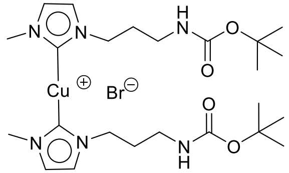	THF-d_8:MeOH (30:1)	RT	42.5 h	10 % (conversion)	By sonication 17 cycles (Each cycle: 5 s Pulse, 10 s Pause for 60 min)	15
14	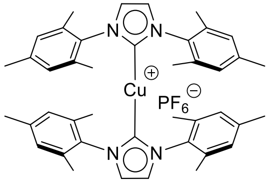	CH_3CN	120 °C (17 bar)	20 mins	89 % (conversion)	Electrochemical flow	16
15	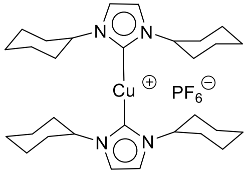	- (neat)	RT	5 mins	99 %	0.5 mol % cat (air sensitive precarbene ligand)	17
16	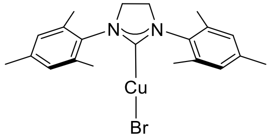	- (neat)	RT	20 mins	98 %	0.8 mol % cat (air sensitive precarbene ligand)	18
17	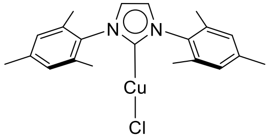	H_2O	80 °C	0.25 h	78 %	Under microwave conditions For 1,3-bis(1-benzyl-1,2,3-triazol-4-yl)benzene synthesis	19
18	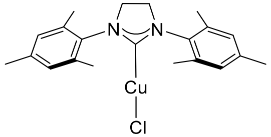	Lidocaine / decanoic acid	RT	24 h	100 % (conversion)	Catalyst dissolved at 45 °C For (1-benzyl-1,2,3-triazol-4-yl)methanol synthesis	20

Table S4. Toxicity of Cu(I) catalysts used for the CuAAC in live cells

S.No	Cu(I) catalyst	Cells	Toxicity	Remarks	Ref
1	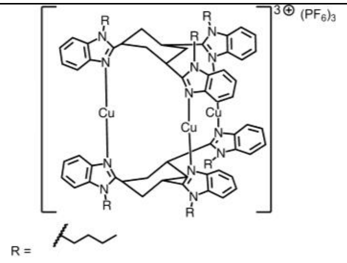	MDA-MB-468<br>SUM-159	IC_{50} = 47 μM<br>IC_{50} = 84 μM	MTT assay	21
2	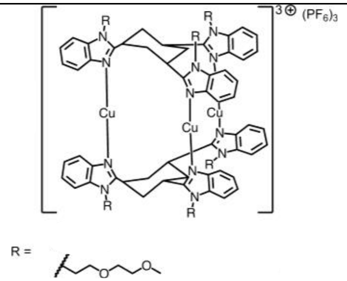	MDA-MB-468<br>SUM-159	IC_{50} = 79 μM<br>IC_{50} = > 100 μM	Used in intracellular labelling<br>MTT assay	
3	Cu(I) microdevice<br>(resin)	MDA-MB-231	2 mg/mL toxic	PrestoBlue assay	22
4	$CuSO_4$/BTTP/Na-asc	Jurkat	75 μM 26 % viability	For live-cell labelling<br>Cell survival by gating (forward scatter)	23
5	$CuSO_4$/BTTPS/Na-asc		75 μM 60 % viability		
6	$CuSO_4$/BTTPS/Na-asc<br>With chelating azide	Jurkat	75 μM non-toxic	For *in vivo* glycosylation<br>Cell viability at 1.5 and 3 mins<br>Cell survival by gating (forward scatter)	24
7	$CuSO_4$/TBTA/Na-asc	Jurkat	50 μM toxic	Cell viability at 3 mins<br>Trypan blue assay	25
8	$CuSO_4$/THPTA/Na-asc		50 μM non-toxic		

9	$CuSO_4$/BTTES/Na-asc		50 µM non-toxic		
10	$CuSO_4$/BTTAA/Na-asc		50 µM non-toxic		
11	$CuSO_4$/BTTES/Na-asc	Jurkat	75 µM non-toxic	For in vivo imaging of glycans	[26]
12		CHO			
13		HEK			
14	$CuSO_4$/BTTAA/Na-asc	A375	50 µM non-toxic	Protein and Cell Surface Modifications<br>MTT assay	[27]
15		HeLa			
16		SKBR-3			
17	MOF based Cu(I)	MCF7	100 µg/mL non-toxic	For in vivo drug synthesis<br>MTT assay	[28]
18	$CuSO_4$/BTTAA/Na-asc	HUVEC	100 µM 75 % viability	For CuAAC in live cells<br>MTS assay	[29]
19	$CuSO_4$/Tat*/Na-asc		100 µM 70 % viability		
20	$CuSO_4$/BTTAA/Na-asc	OVCAR5	100 µM 75 % viability		
21	$CuSO_4$/Tat*/Na-asc		100 µM 72 % viability		
22	Cu-MONPs/Na-asc	HEK-293	15 µg/mL non-toxic	For CuAAC in cells<br>MTT assay	[30]
23		MDA-MB-231	15 µg/mL non-toxic		
24	Cu(I)-BPS	MDA-MB-468	IC_{50} = 8.65 µM	MTT assay	[31]
25	Cu(I)-THPTA	MDA-MB-468	IC_{50} = 22.4 µM		
26	Cu(I)-TBTA	MDA-MB-468	IC_{50} = 29.6 µM		
27	Cu(I)-BPS	HeLa	IC_{50} = 45.6 µM		
28	Cu(I)-THPTA	HeLa	IC_{50} = 183 µM		

29	Cu(I)-TBTA	HeLa	IC_{50} = 12.2 μM		
30	$CuSO_4$/BTTE/Na-asc	HeLa	75 μM 80 % viability	For CuAAC in cells<br>MTT assay	32
31	$CuSO_4$/BTTEP/Na-asc		75 μM 80 % viability		
32	[Cu(BTTE)]PF_6		50 μM 70 % viability		
33	$CuSO_4$/THPTA/Na-asc<br>With chelating azide	HeLa	40 μM less toxic	For protein labelling<br>CellTiter-Glo	33
34	$CuSO_4$/BTTAA/Na-asc<br>With chelating azide		40 μM less toxic		
35	Cu@LANPs<br>Lipoic acid nanoparticles	HeLa	100 ppm non-toxic	For intracellular catalysis<br>CCK-8 assay	34
36	$CuSO_4$		50 ppm toxic		
37	Cu-SCPN	HeLa	33.3 μM toxic	For bio-orthogonal catalysis<br>Presto Blue assay	35
38	E-Cu-NPs<br>(resin)	HeLa	0.3 mg non-toxic	In situ drug synthesis<br>MTT assay	36
39	$Cu(OAc)_2$/THPTA/Na-asc	HeLa S3	50 μM toxic	Used in protein labelling<br>CCK-8 assay	37
40	$CuSO_4$/chelating azide/Na-asc	HeLa	100 μM non-toxic	For conjugation reactions<br>MTS assay	38
41		Huh7			

2.5 General procedure for the click reactions

2.5.1. General method A (In aqueous medium, (milliQ water, buffers) and biological media (DMEM and RPMI) Tables 1-4): In a 5 mL glass vial, azide (0.25 mmol), alkyne (0.3 mmol) and Cu(I)-NHC complex (**2**) (1 mol %, 2.5 µmol) were added followed by solvent (0.5 mL). The mixture was stirred at 37 °C for 2 h in a preheated oil bath (variable conditions as mentioned in the tables). After the completion of the reaction, the formed solid was extracted from water using either EtOAc or Et_2O (*ca*. 25 mL). The extracted organic solvent was dried over anhydrous $MgSO_4$ and the solvent was evaporated under reduced pressure to get the clean 1,2,3-triazole products. In some cases, to remove traces of the alkyn, the crude product was washed with Et_2O (for compounds extracted with EtOAc) or cold pentane (for compounds extracted with Et_2O) to get the clean 1,2,3-triazole products, or purified by a short silica column chromatography.

General method B (Neat, Table 1, entries 7-8): In a 5 mL glass vial, azide (0.25 mmol), alkyne (0.3 mmol) and Cu(I)-NHC complex (**2**) (1 mol %, 2.5 µmol) were added. The mixture was stirred at 37 °C for 2 h in a preheated oil bath. After the completion of the reaction, the formed crude product was washed with Et_2O to get the 1,2,3-triazole product.

General method C (In organic solvent, Table 2, entries 1-7): In a 5 mL glass vial, azide (0.25 mmol), alkyne (0.3 mmol) and Cu(I)-NHC complex (**2**) (1 mol %, 2.5 µmol) were added followed by solvent (0.5 mL). The mixture was stirred at 37 °C for 2 h in a preheated oil bath (variable conditions as mentioned in the tables). After the completion of the reaction, volatile components were evaporated under reduced pressure and the obtained residue was washed with Et_2O to get the 1,2,3-triazole products.

2.5.2. Click reaction in presence of amphiphilic polymer (8) in DMEM for solid substrates

In a 5 mL glass vial, azide (0.125 mmol), alkyne (0.15 mmol), 80:20 (Jeffamine®M1000:dodecylamine) amphiphilic polymer[4] (**8**) (0.1 mol %, 0.125 µmol) and Cu(I)-NHC complex (**2**) (1 mol %, 1.25 µmol) were added followed by DMEM (0.5 mL). The mixture was stirred at 37 °C for 2 h in a preheated oil bath. After the completion of the reaction, the reaction mixture was diluted with water (*ca.* 5 mL) and extracted using EtOAc (*ca*. 25 mL). The extracted organic solvent was dried over anhydrous $MgSO_4$ and the solvent was evaporated under reduced pressure to get the clean 1,2,3-triazole product.

2.5.3. Click reaction in presence of glutathione

In a 5 mL glass vial, to a mixture of benzylazide (0.25 mmol) and phenylacetylene (0.30 mmol), aqueous glutathione (0.5 mL, 10 mM) solution was added followed by Cu(I)-NHC complex (**2**) (1 mol %, 2.5 µmol). The mixture was stirred at 37 °C for 2 h in a preheated oil bath. After the completion of the reaction, the reaction mixture was diluted with water (*ca.* 5 mL) and extracted using EtOAc (*ca*. 25 mL). The extracted organic solvent was dried over anhydrous $MgSO_4$ and the solvent was evaporated under reduced pressure, and the obtained residue was washed with Et_2O to get the clean 1,2,3-triazole product (**3a**). Polymer (**8**) encapsulated Cu(I)-NHC complex (**2**) was prepared with 1:20 molar ratio of polymer:Cu(I) as stated in the general procedure below and with a 1:20:2000 molar ratio of polymer : catalyst : substrate.

2.5.4. General procedure for the multicomponent click reaction

In a 5 mL glass vial, halide (0.25 mmol), NaN_3 (0.020g, 0.31 mmol), phenylacetylene (33 µl, 0.3 mmol) and Cu(I)-NHC complex (**2**) (1 mol %, 2.5 µmol) were added followed by water (0.5 mL). The mixture was stirred at 37 °C for 2 h in a preheated oil bath (variable conditions as mentioned in the table 5). After the completion of the reaction, the formed solid was extracted from water using EtOAc (*ca*. 25 mL). The extracted organic solvent was dried over anhydrous $MgSO_4$ and the solvent was evaporated under reduced pressure and the obtained residue was washed with Et_2O to get the 1,2,3-triazole products.

2.5.5. Reusability of Cu(I)-NHC (2) complex

The reusability of the catalyst was accessed by adding a different alkyne in the same reaction pot, after the initial reaction time of 2 h, i.e., after reacting benzyl azide (0.25 mmol) and phenylacetylene for 2 h, in the same reaction, benzylacetylene and benzyl azide (0.25 mmol) were added to check if the catalyst was still active to produce the second benzylacetylene derived triazole. Interestingly, the benzylacetylene derived triazole was also formed, as clearly seen in the NMR (Fig. S128), confirming that the catalyst remained active after the first cycle. In some cases, the crude NMR of the catalysis product showed the presence of the NHC•HBr salt (NCHN) resonance (Fig. S130), validating the formation of (NHC)Cu-acetylide intermediate, by displacing one of the NHC ligand in (**2**).

2.6 Encapsulation efficiency study

The Cu-NHC complex was encapsulated in the polymer and the encapsulation efficiency was calculated by measuring the concentration of Cu leached out from the polymer using ICP-OES.[39] This was done by centrifuge filtration using a 10 kD cutoff filter that allows only the small molecules to pass through while retaining the higher molecular weight molecules (the encapsulated Cu-NHC stays with polymer). The polymer to catalyst encapsulation ratio (P:C, 1.2 µM:12 µM) was kept as 1:10 molar ratio. Polymer (4.893 mg, 48 µM) stock prepared in 514 µL DCM. Cu-NHC (0.645 mg, 2.5 mM) stock prepared in 263 µL DCM. To make P:C 4.8 µM:48.0 µM in 1 mL water, the required amounts of polymer (100 µL) and catalyst (19.2 µL) were co-dissolved in DCM in a HPLC glass vial. DCM was gently allowed to evaporate by argon flow to form a thin film on glass, and this film was dried completely at 40 °C for 1 h in a vacuum oven. To this vial, 1000 µL milliQ water was added, vortexed, and sonicated for 45 min. After sonication, the solution was kept for equilibration for the required time intervals. This solution was centrifuge filtered over a 10 kD ultra – 4 centrifugal filter (Amicon®, 10 kDa) with 3000 rpm for 15 minutes twice. The filtrate was finally diluted to 4 mL (at this stage concentration is P:C 1.2 µM:12.0 µM, Cu = 0.763 ppm) and was analysed using ICP-OES. 1000 mg/l Cu standard solution was used to determine the calibration curves for quantification of leached copper. The encapsulation efficiency when keeping the encapsulated copper complex in water for 3 days over time showed 99 % efficiency (Fig. S129).

1-Benzyl-4-phenyl-1H-1,2,3-triazole (3a)[40]

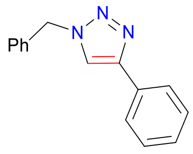

(**3a**)

Off white solid, yield: 90%, ^{1}H NMR (400 MHz, $CDCl_3$, 25 ˚C, δ ppm): 7.80 (d, J = 8.0 Hz, 2H), 7.66 (s, 1H), 7.42-7.36 (m, 5H), 7.33-7.30 (m, 3H), 5.58 (s, 2H, C$\underline{H_2}$). $^{13}C\{^1H\}$ NMR (100 MHz,

$CDCl_3$, 25 ˚C, δ ppm): 148.19, 134.78, 130.61, 129.15, 128.83, 128.76, 128.17, 128.06, 125.72, 119.66, 54.18. MALDI-ToF-MS: *m/z* calcd. for $C_{15}H_{14}N_3^+$: 236.12, found: 236.12 $[M+H]^+$.

1-Benzyl-4-(4-bromophenyl)-1H-1,2,3-triazole (3b)[41]

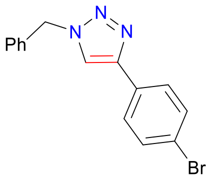

(**3b**)

Off white solid, yield: 94 %, ^{1}H NMR (400 MHz, $CDCl_3$, 25 ˚C, δ ppm): 7.68 (s, 1H), 7.66 (d, *J* = 4.0 Hz, 2H), 7.52 (d, *J* = 8.0 Hz, 2H), 7.40–7.38 (m, 3H), 7.33–7.30 (m, 2H), 5.58 (s, 2H, C*H*$_2$). ^{13}C{^{1}H} NMR (100 MHz, $CDCl_3$, 25 ˚C, δ ppm): 147.32, 134.63, 132.08, 129.65, 129.34, 129.02, 128.25, 127.35, 122.17, 119.69, 54.44 ppm. MALDI-ToF-MS: *m/z* calcd. for $C_{15}H_{13}N_3Br^+$: 314.03, found: 314.03 $[M+H]^+$.

1-Benzyl-4-(3,5-difluorophenyl)-1H-1,2,3-triazole (3c)

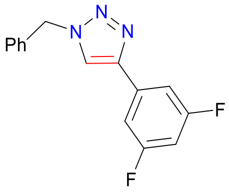

(**3c**)

Off white solid, yield: 96 %, ^{1}H NMR (400 MHz, $CDCl_3$, 25 ˚C, δ ppm): 7.67 (s, 1H), 7.40-7.38 (m, 3H), 7.32-7.30 (m, 4H), 6.74 (tt, J_{HF} = 8.0 Hz, J_{HH} = 4.0 Hz, 1H), 5.57 (s, 2H, C*H*$_2$). ^{13}C{^{1}H} NMR (100 MHz, $CDCl_3$, 25 ˚C, δ ppm): 164.72 (d, J_{CF} = 13.0 Hz), 162.24 (d, J_{CF} = 13.0 Hz), 146.39 (s), 134.42 (s), 133.80 (t, J_{CF} = 11.0 Hz), 129.38 (s), 129.11 (s), 128.27 (s), 120.34 (s), 108.64 (d, J_{CF} = 27.0 Hz), 108.63 (d, J_{CF} = 11.0 Hz), 103.45 (t, J_{CF} = 25.0 Hz), 54.51 (s). ^{19}F{^{1}H} NMR (376 MHz, $CDCl_3$, 25 ˚C, δ ppm): −109.30. MALDI-ToF-MS: *m/z* calcd. for $C_{15}H_{12}\ F_2N_3^+$: 272.10, found: 272.14 $[M+H]^+$.

1-Benzyl-4-(3,5-bis(trifluoromethyl)phenyl)-1H-1,2,3-triazole (3d)[42]

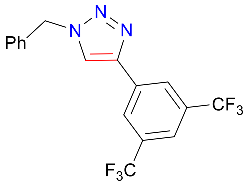

(**3d**)

Off white solid, yield: 87 %, ^{1}H NMR (400 MHz, $CDCl_3$, 25 ˚C, δ ppm): 8.25 (s, 2H), 7.83 (s, 1H), 7.80 (s, 1H), 7.42-7.40 (m, 3H), 7.35-7.33 (m, 2H), 5.61 (s, 2H, C*H*$_2$). ^{13}C{^{1}H} NMR (100 MHz, $CDCl_3$, 25 ˚C, δ ppm): 145.67, 134.25, 132.83, 132.51, 132.18, 129.47, 129.24, 128.33, 125.74, 124.69, 121.97, 121.75-121.61 (m, CF_3), 120.65, 54.67. ^{19}F{^{1}H} NMR (376 MHz, $CDCl_3$, 25 ˚C, δ ppm): −63.00. LCMS ESI: *m/z* calcd. for $C_{17}H_{12}F_6N_3^+$: 372.09, found: 372.25 $[M+H]^+$.

3-(1-Benzyl-1H-1,2,3-triazol-4-yl)phenol (3e)

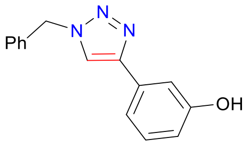

(3e)

Off white solid, yield: 92 %, ^{1}H NMR (400 MHz, CD_3OD, 25 ˚C, δ ppm): 8.17 (s, 1H), 7.36-7.30 (m, 5H), 7.25-7.18 (m, 3H), 6.77 (dt, ^{3}J = 8.0 Hz, ^{4}J = 4.0 Hz, 1H), 5.56 (s, 2H, $C\underline{H}_2$). $^{13}C\{^{1}H\}$ NMR (100 MHz, CD_3OD, 25 ˚C, δ ppm): 159.04, 149.22, 136.71, 132.78, 131.06, 130.01, 129.55, 129.02, 122.20, 117.99, 116.37, 113.40, 54.97. MALDI-ToF-MS: *m/z* calcd. for $C_{15}H_{14}N_3O^+$: 252.11, found: 252.13 $[M+H]^+$, 274.08 $[M+Na]^+$.

3-(1-Benzyl-1H-1,2,3-triazol-4-yl)benzoic acid (3f)

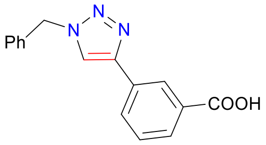

(3f)

Off white solid, yield: 94 %, ^{1}H NMR (400 MHz, CD_3OD, 25 ˚C, δ ppm): 8.45 (s, 1H), 8.39 (s, 1H), 8.01 (d, J = 8.0 Hz, 1H), 7.97 (d, J = 8.0 Hz, 1H), 7.54 (t, J = 8.0 Hz, 1H), 7.38-7.33 (m, 5H), 5.63 (s, 2H, $C\underline{H}_2$). $^{13}C\{^{1}H\}$ NMR (100 MHz, CD_3OD, 25 ˚C, δ ppm): 169.12, 148.33, 137.05, 136.73, 132.16, 130.89, 130.51, 130.22, 130.06, 129.63, 129.14, 127.92, 122.66, 55.09. MALDI-ToF-MS: *m/z* calcd. for $C_{16}H_{14}N_3O_2^+$: 280.11, found: 280.11 $[M+H]^+$, 302.10 $[M+Na]^+$.

4-(1-Benzyl-1H-1,2,3-triazol-4-yl)aniline (3g)[43]

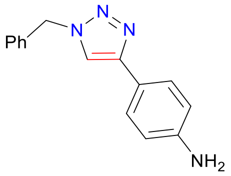

(3g)

Off white solid, yield: 90 %, ^{1}H NMR (400 MHz, $CDCl_3$, 25 ˚C, δ ppm): 7.59 (d, J = 8.0 Hz, 2H), 7.52 (s, 1H), 7.38-7.36 (m, 3H), 7.30-7.28 (m, 2H), 6.70 (d, J = 8.0 Hz, 2H), 5.54 (s, 2H, $C\underline{H}_2$), 3.74 (s, 2H, $N\underline{H}_2$). $^{13}C\{^{1}H\}$ NMR (100 MHz, $CDCl_3$, 25 ˚C, δ ppm): 148.70, 146.61, 135.01, 129.23, 128.81, 128.15, 127.04, 121.21, 118.33, 115.33, 54.27. MALDI-ToF-MS: *m/z* calcd. for $C_{15}H_{15}N_4^+$: 251.13, found: 251.14 $[M+H]^+$, 273.12 $[M+Na]^+$.

1-Benzyl-4-(*m*-tolyl)-1H-1,2,3-triazole (3h)[44]

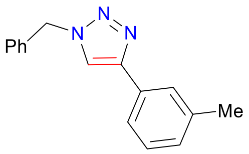

(**3h**)

Off white solid, yield: 90 %, ^{1}H NMR (400 MHz, $CDCl_3$, 25 ˚C, δ ppm): 7.66 (s, 1H), 7.65 (s, 1H), 7.57 (d, J = 8.0 Hz, 1H), 7.39-7.36 (m, 3H), 7.31-7.28 (m, 3H), 7.13 (d, J = 8.0 Hz, 1H), 5.57 (s, 2H, $C\underline{\mathit{H}}_2$), 2.38 (s, 3H, $C\underline{\mathit{H}}_3$). $^{13}C\{^1H\}$ NMR (100 MHz, $CDCl_3$, 25 ˚C, δ ppm): 148.45, 138.60, 134.85, 130.52, 129.26, 129.04, 128.87, 128.81, 128.16, 126.49, 122.91, 119.58, 54.32, 21.51. MALDI-ToF-MS: *m/z* calcd. for $C_{16}H_{16}N_3^+$: 250.13, found: 250.14 $[M+H]^+$.

1-Benzyl-4-(4-methoxyphenyl)-1H-1,2,3-triazole (3i)[45]

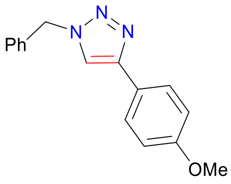

(**3i**)

Off white solid, yield: 92 %, ^{1}H NMR (400 MHz, $CDCl_3$, 25 ˚C, δ ppm): 7.72 (d, J = 8.0 Hz, 2H), 7.57 (s, 1H), 7.39-7.37 (m, 3H), 7.32-7.30 (m, 2H), 6.93 (d, J = 8.0 Hz, 2H), 5.57 (s, 2H, $C\underline{\mathit{H}}_2$), 3.83 (s, 3H, $OC\underline{\mathit{H}}_3$). $^{13}C\{^1H\}$ NMR (100 MHz, $CDCl_3$, 25 ˚C, δ ppm): 159.71, 148.20, 134.90, 129.24, 128.84, 128.15, 127.11, 123.39, 118.82, 114.31, 55.42, 54.29. MALDI-ToF-MS: *m/z* calcd. for $C_{16}H_{16}N_3O^+$: 266.13, found: 266.15 $[M+H]^+$.

1-Benzyl-4-(3,5-dimethoxyphenyl)-1H-1,2,3-triazole (3j)

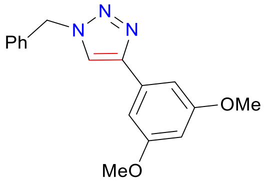

(**3j**)

Off white solid, yield: 88 %, ^{1}H NMR (400 MHz, $CDCl_3$, 25 ˚C, δ ppm): 7.64 (s, 1H), 7.38-7.35 (m, 3H), 7.31-7.29 (m, 2H), 6.97 (d, J = 4.0 Hz, 2H), 6.43 (t, J = 4.0 Hz, 1H), 5.55 (s, 2H, $C\underline{\mathit{H}}_2$), 3.82 (s, 6H, $OC\underline{\mathit{H}}_3$). $^{13}C\{^1H\}$ NMR (100 MHz, $CDCl_3$, 25 ˚C, δ ppm): 161.25, 148.20, 134.75, 132.47, 129.26, 128.89, 128.16, 119.93, 103.72, 100.74, 55.57, 54.33. MALDI-ToF-MS: *m/z* calcd. for $C_{17}H_{18}N_3O_2^+$: 296.14, found: 296.15 $[M+H]^+$, 318.08 $[M+Na]^+$.

1,4-Dibenzyl-1H-1,2,3-triazole (3k)[46]

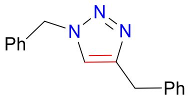

(**3k**)

Off white solid, yield: 98 %, ^{1}H NMR (400 MHz, $CDCl_3$, 25 ˚C, δ ppm): 7.36-7.33 (m, 3H), 7.29-7.19 (m, 8H), 5.49 (s, 2H, NC$\underline{H}_2$), 4.06 (s, 2H, C$\underline{H}_2$). ^{13}C{^{1}H} NMR (100 MHz, $CDCl_3$, 25 ˚C, δ ppm): 148.32, 139.09, 134.90, 129.12, 128.78, 128.70, 128.67, 128.03, 126.54, 121.54, 54.14, 32.38. MALDI-ToF-MS: *m/z* calcd. for $C_{16}H_{16}N_3^+$: 250.13, found: 250.17 $[M+H]^+$, 272.15 $[M+Na]^+$.

1-Benzyl-4-(phenoxymethyl)-1H-1,2,3-triazole (3l)[47]

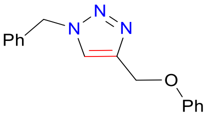

(3l)

Off white solid, yield: 94 %, ^{1}H NMR (400 MHz, $CDCl_3$, 25 ˚C, δ ppm): 7.53 (s, 1H), 7.38-7.36 (m, 3H), 7.30-7.26 (m, 4H), 6.97-6.95 (m, 3H), 5.52 (s, 2H), 5.18 (s, 2H). ^{13}C{^{1}H} NMR (100 MHz, $CDCl_3$, 25 ˚C, δ ppm): 158.27, 144.77, 134.56, 129.63, 129.25, 128.91, 128.22, 122.69, 121.34, 114.83, 62.11, 54.33. MALDI-ToF-MS: *m/z* calcd. for $C_{16}H_{16}N_3O^+$: 266.13, found: 266.17 $[M+H]^+$.

1,4-Bis(1-benzyl-1H-1,2,3-triazol-4-yl)benzene (3m)[48]

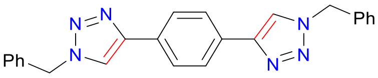

(3m)

Off white solid, yield: 82 %, ^{1}H NMR (400 MHz, DMSO-d_6, 25 ˚C, δ ppm): 8.68 (s, 2H), 7.92 (s, 4H), 7.42-7.34 (m, 10H), 5.65 (s, 4H, C$\underline{H}_2$). ^{13}C{^{1}H} NMR (100 MHz, $CDCl_3$, 25 ˚C, δ ppm): 146.37, 136.0, 130.12, 128.85, 128.21, 127.95, 125.65, 121.70, 53.08. LCMS ESI: *m/z* calcd. for $C_{24}H_{21}N_6^+$: 393.18, found: 393.50 $[M+H]^+$, 415.42 $[M+Na]^+$.

3-(1-Benzyl-1H-1,2,3-triazol-4-yl)-5-bromopyrazin-2-amine (3n)

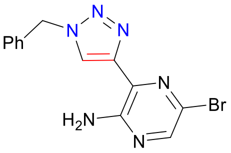

(3n)

Off white solid, yield: 81 %, ^{1}H NMR (400 MHz, $CDCl_3$, 25 ˚C, δ ppm): 8.17 (s, 1H), 8.01 (s, 1H), 7.39-7.38 (m, 3H), 7.33-7.30 (m, 2H), 6.64 (b, 2H, N$\underline{H}_2$), 5.59 (s, 2H, C$\underline{H}_2$). ^{13}C{^{1}H} NMR (100 MHz, $CDCl_3$, 25 ˚C, δ ppm): 151.02, 147.05, 143.06, 134.02, 129.69, 129.42, 129.24, 128.42, 124.65, 123.69, 54.76. MALDI-ToF-MS: *m/z* calcd. for $C_{13}H_{12}BrN_6^+$: 331.03, found: 331.03 $[M+H]^+$.

1-(1-Benzyl-1H-1,2,3-triazol-4-yl)cyclohexan-1-ol (3o)[49]

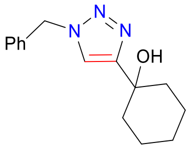

(3o)

Off white solid, yield: 92 %, ^{1}H NMR (400 MHz, $CDCl_3$, 25 °C, δ ppm): 7.37-7.35 (m, 4H), 7.27-7.25 (m, 2H), 5.49 (s, 2H, NC$\underline{H}_2$), 2.56 (s, 1H, O$\underline{H}$), 1.98-1.91 (m, 2H), 1.86-1.83 (m, 2H), 1.77-1.68 (m, 2H), 1.63-1.60 (m, 1H), 1.55-1.50 (m, 2H), 1.37-1.29 (m, 1H). ^{13}C{^{1}H} NMR (100 MHz, $CDCl_3$, 25 °C, δ ppm): 156.17, 134.76, 129.19, 128.80, 128.20, 119.57, 69.65, 54.20, 38.17, 25.44, 22.03. MALDI-ToF-MS: *m/z* calcd. for $C_{15}H_{20}N_3O^+$: 258.16, found: 258.18 $[M+H]^+$, 280.15 $[M+Na]^+$.

1-Benzyl-1H-1,2,3-triazole-4-carboxylic acid (3p)[50]

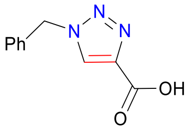

(3p)

Off white solid, yield: 88 %, ^{1}H NMR (400 MHz, CD_3OD, 25 °C, δ ppm): 8.51 (s, 1H), 7.39-7.35 (m, 5H), 5.66 (s, 2H, C$\underline{H}_2$). ^{13}C{^{1}H} NMR (100 MHz, CD_3OD, 25 °C, δ ppm): 136.05, 130.13, 129.83, 129.32, 129.22 (due to the poor solubility, quaternary carbons are not observed), 55.38. LCMS ESI: *m/z* calcd. for $C_{10}H_{10}N_3O_2^+$: 204.08, found: 204.75 $[M+H]^+$, 226.67 $[M+Na]^+$.

***tert*-Butyl ((1-benzyl-1H-1,2,3-triazol-4-yl)methyl)carbamate (3q)**[51]

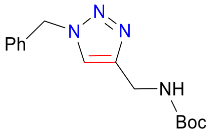

(3q)

Off white solid, yield: 90 %, ^{1}H NMR (400 MHz, $CDCl_3$, 25 °C, δ ppm): 7.46 (s, 1H), 7.36-7.34 (m, 3H), 7.25-7.24 (m, 2H), 5.49 (s, 2H, NC$\underline{H}_2$), 5.31 (b, 1H, N$\underline{H}$), 4.36 (d, J = 4.0 Hz, 2H, NHC$\underline{H}_2$), 1.40 (s, 9H). ^{13}C{^{1}H} NMR (100 MHz, $CDCl_3$, 25 °C, δ ppm): 155.89, 146.07, 134.63, 129.11, 128.75, 128.08, 121.99, 79.62, 54.16, 36.12, 28.37. MALDI-ToF-MS: *m/z* calcd. for $C_{15}H_{21}N_4O_2^+$: 289.17, found: 289.19 $[M+H]^+$, 311.17 $[M+Na]^+$, 327.15 $[M+K]^+$.

1-Benzyl-4-(triisopropylsilyl)-1H-1,2,3-triazole (3r)[52]

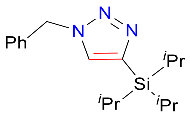

(3r)

Off white solid, yield: 72 %, ^{1}H NMR (400 MHz, $CDCl_3$, 25 °C, δ ppm): 7.45 (s, 1H), 7.37-7.33 (m, 3H), 7.23-7.20 (m, 2H), 5.59 (s, 2H, C$\underline{H}_2$), 1.35 (sept, J = 8.0 Hz, 3H, {C$\underline{H}$(CH_3)$_2$}$_3$), 1.08 (d, J = 8.0 Hz, 18H, {CH(C$\underline{H}_3$)$_2$}$_3$). ^{13}C{^{1}H} NMR (100 MHz, $CDCl_3$, 25 °C, δ ppm): 142.61, 135.41,

130.18, 129.16, 128.59, 127.82, 53.48, 18.70, 11.22. MALDI-ToF-MS: *m/z* calcd. for $C_{18}H_{30}N_3Si^+$: 316.22, found: 316.22 $[M+H]^+$, 338.19 $[M+Na]^+$, 354.41 $[M+K]^+$.

1-Benzyl-4-propyl-1H-1,2,3-triazole (3s)[53]

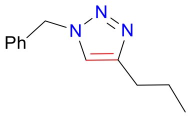

(**3s**)

Off white solid, yield: 88 %, ^{1}H NMR (400 MHz, $CDCl_3$, 25 ˚C, δ ppm): 7.31-7.26 (m, 3H), 7.21-7.16 (m, 2H), 7.11 (s, 1H), 5.41 (s, 2H, $NC\underline{H}_2$), 2.59 (t, J = 8.0 Hz, 2H, $C\underline{H}_2CH_2CH_3$), 1.59 (q, J = 8.0 Hz, 2H, $CH_2C\underline{H}_2CH_3$), 0.87 (t, J = 8.0 Hz, 3H, $CH_2CH_2C\underline{H}_3$). $^{13}C\{^1H\}$ NMR (100 MHz, $CDCl_3$, 25 ˚C, δ ppm): 148.84, 135.14, 129.14, 128.67, 128.04, 120.65, 54.05, 27.83, 22.76, 13.87. LCMS ESI: *m/z* calcd. for $C_{12}H_{16}N_3^+$: 202.13, found: 202.17 $[M+H]^+$, 224.08 $[M+Na]^+$.

1-Benzyl-4-(diethoxymethyl)-1H-1,2,3-triazole (3t)[54]

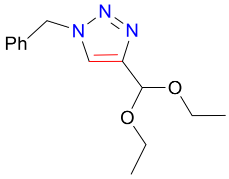

(**3t**)

Off white solid, yield: 80 %, ^{1}H NMR (400 MHz, $CDCl_3$, 25 ˚C, δ ppm): 7.52 (s, 1H), 7.38-7.36 (m, 3H), 7.29-7.27 (m, 2H), 5.70 (s, 1H), 5.51 (s, 2H), 3.69-3.57 (m, 4H), 1.22 (t, J = 8.0 Hz, 6H). $^{13}C\{^1H\}$ NMR (100 MHz, $CDCl_3$, 25 ˚C, δ ppm): 147.58, 134.52, 129.12, 128.76, 128.17, 122.01, 96.85, 61.66, 54.22, 15.15. MALDI-ToF-MS: *m/z* calcd. for $C_{14}H_{20}N_3O_2^+$: 262.16, found: 262.12 $[M+H]^+$, 284.09 $[M+Na]^+$, 300.07 $[M+K]^+$.

(4-Phenyl-1H-1,2,3-triazol-1-yl)methyl pivalate (4a)[55]

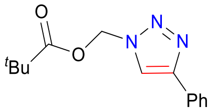

(**4a**)

Off white solid, yield: 82 %, ^{1}H NMR (400 MHz, $CDCl_3$, 25 ˚C, δ ppm): 8.03 (s, 1H), 7.86 (d, J = 4.0 Hz, 2H), 7.44 (t, J = 8.0 Hz, 2H), 7.35 (t, J = 8.0 Hz, 1H), 6.29 (s, 2H), 1.20 (s, 9H). $^{13}C\{^1H\}$ NMR (100 MHz, $CDCl_3$, 25 ˚C, δ ppm): 178.07 (C=O), 148.41, 130.15, 128.99, 128.57, 125.95, 121.07, 69.84, 38.92, 26.93. MALDI-ToF-MS: *m/z* calcd. for $C_{14}H_{18}N_3O_2^+$: 260.14, found: 260.10 $[M+H]^+$, 282.11 $[M+Na]^+$.

(4-(3,5-Difluorophenyl)-1H-1,2,3-triazol-1-yl)methyl pivalate (4b)

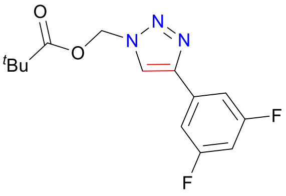

(**4b**)

Off white solid, yield: 92 %, ^{1}H NMR (400 MHz, $CDCl_3$, 25 ˚C, δ ppm): 8.05 (s, 1H), 7.40-7.35 (m, 2H), 6.78 (tt, J_{HF} = 8.0 Hz, J_{HH} = 4.0 Hz, 1H), 6.27 (s, 2H), 1.20 (s, 9H). $^{13}C\{^{1}H\}$ NMR (100 MHz, $CDCl_3$, 25 ˚C, δ ppm): 178.14 (C=O), 164.75 (d, J_{CF} = 13.0 Hz), 162.28 (d, J_{CF} = 13.0 Hz), 146.45 (t, J_{CF} = 4.0 Hz), 133.27 (t, J_{CF} = 10.0 Hz), 121.98 (s), 108.85 (d, J_{CF} = 26.0 Hz), 108.85 (d, J_{CF} = 12.0 Hz), 103.80 (t, J_{CF} = 25.0 Hz), 69.85 (s), 38.95 (s), 26.93 (s). $^{19}F\{^{1}H\}$ NMR (376 MHz, $CDCl_3$, 25 ˚C, δ ppm): −109.03. MALDI-ToF-MS: *m/z* calcd. for $C_{14}H_{16}F_2N_3O_2^{+}$: 296.12, found: 296.14 $[M+H]^{+}$, 318.11 $[M+Na]^{+}$.

(4-(*m*-Tolyl)-1H-1,2,3-triazol-1-yl)methyl pivalate (4c)

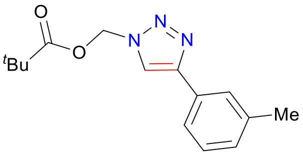

(**4c**)

Off white solid, yield: 96 %, ^{1}H NMR (400 MHz, $CDCl_3$, 25 ˚C, δ ppm): 8.01 (s, 1H), 7.70 (s, 1H), 7.62 (d, J = 8.0 Hz, 1H), 7.31 (t, J = 8.0 Hz, 1H), 7.16 (d, J = 8.0 Hz, 1H), 6.27 (s, 2H), 2.40 (s, 3H), 1.19 (s, 9H). $^{13}C\{^{1}H\}$ NMR (100 MHz, $CDCl_3$, 25 ˚C, δ ppm): 178.09 (C=O), 148.50, 138.70, 129.97, 129.34, 128.89, 126.59, 123.02, 121.06, 69.80, 38.91, 26.92, 21.52. MALDI-ToF-MS: *m/z* calcd. for $C_{15}H_{20}N_3O_2^{+}$: 274.16, found: 274.16 $[M+H]^{+}$.

(4-(4-Methoxyphenyl)-1H-1,2,3-triazol-1-yl)methyl pivalate (4d)

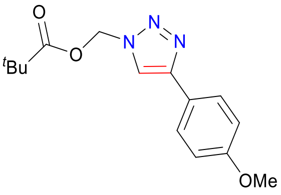

(**4d**)

Off white solid, yield: 90 %, ^{1}H NMR (400 MHz, $CDCl_3$, 25 ˚C, δ ppm): 7.93 (s, 1H), 7.78 (d, J = 8.0 Hz, 2H), 6.97 (d, J = 8.0 Hz, 2H), 6.27 (s, 2H), 3.85 (s, 3H), 1.20 (s, 9H). $^{13}C\{^{1}H\}$ NMR (100 MHz, $CDCl_3$, 25 ˚C, δ ppm): 178.09 (C=O), 159.95, 148.30, 127.28, 122.86, 120.19, 114.41, 69.84, 55.45, 38.93, 26.95. MALDI-ToF-MS: *m/z* calcd. for $C_{15}H_{20}N_3O_3^{+}$: 290.15, found: 290.16 $[M+H]^{+}$, 312.13 $[M+Na]^{+}$.

(4-(3,5-Dimethoxyphenyl)-1H-1,2,3-triazol-1-yl)methyl pivalate (4e)

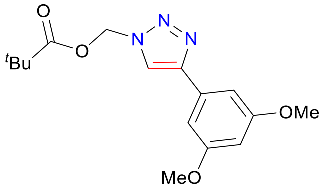

(**4e**)

Off white solid, yield: 90 %, ^{1}H NMR (400 MHz, $CDCl_3$, 25 ˚C, δ ppm): 8.00 (s, 1H), 7.01 (d, *J* = 4.0 Hz, 2H), 6.45 (t, *J* = 4.0 Hz, 1H), 6.27 (s, 2H), 3.84 (s, 6H), 1.19 (s, 9H). $^{13}C\{^1H\}$ NMR (100 MHz, $CDCl_3$, 25 ˚C, δ ppm): 178.10 (C=O), 161.31, 148.28, 131.93, 121.40, 103.82, 101.04, 69.82, 55.62, 38.93, 26.93. MALDI-ToF-MS: *m/z* calcd. for $C_{16}H_{22}N_3O_4^+$: 320.16, found: 320.17 $[M+H]^+$, 342.14 $[M+Na]^+$.

(4-Benzyl-1H-1,2,3-triazol-1-yl)methyl pivalate (4f)

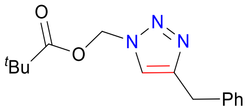

(**4f**)

Off white solid, yield: 94 %, ^{1}H NMR (400 MHz, $CDCl_3$, 25 ˚C, δ ppm): 7.45 (s, 1H), 7.31-7.27 (m, 2H), 7.25-7.20 (m, 3H), 6.17 (s, 2H), 4.09 (s, 2H), 1.17 (s, 9H). $^{13}C\{^1H\}$ NMR (100 MHz, $CDCl_3$, 25 ˚C, δ ppm): 177.85 (C=O), 138.78, 128.74, 126.67, 122.93, 69.72, 38.85, 32.16, 26.87. MALDI-ToF-MS: *m/z* calcd. for $C_{15}H_{20}N_3O_2^+$: 274.16, found: 274.15 $[M+H]^+$, 296.13 $[M+Na]^+$, 312.11 $[M+K]^+$.

(4-(Phenoxymethyl)-1H-1,2,3-triazol-1-yl)methyl pivalate (4g)

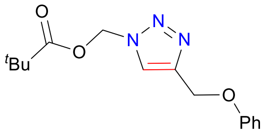

(**4g**)

Off white solid, yield: 96 %, ^{1}H NMR (400 MHz, $CDCl_3$, 25 ˚C, δ ppm): 7.87 (s, 1H), 7.30-7.27 (m, 2H), 6.99-6.95 (m, 3H), 6.22 (s, 2H), 5.21 (s, 2H), 1.18 (s, 9H). $^{13}C\{^1H\}$ NMR (100 MHz, $CDCl_3$, 25 ˚C, δ ppm): 177.81 (C=O), 158.10, 144.90, 129.60, 124.26, 121.40, 114.84, 69.72, 61.79, 38.83, 26.85. MALDI-ToF-MS: *m/z* calcd. for $C_{15}H_{20}N_3O_3^+$: 290.15, found: 290.16 $[M+H]^+$, 312.16 $[M+Na]^+$.

(4-(Diethoxymethyl)-1H-1,2,3-triazol-1-yl)methyl pivalate (4h)

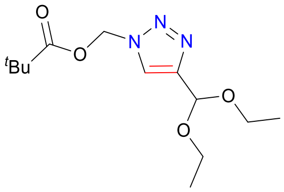

(**4h**)

Off white solid, yield: 93 %, ^{1}H NMR (400 MHz, $CDCl_3$, 25 ˚C, δ ppm): 7.82 (s, 1H), 6.21 (s, 2H), 5.70 (s, 1H), 3.69-3.55 (m, 4H), 1.22 (t, J = 8.0 Hz, 6H), 1.17 (s, 9H). $^{13}C\{^{1}H\}$ NMR (100 MHz, $CDCl_3$, 25 ˚C, δ ppm): 177.82 (C=O), 147.77, 123.67, 96.61, 61.74, 38.88, 26.90, 15.24. MALDI-ToF-MS: m/z calcd. for $C_{13}H_{23}N_3NaO_4^{+}$: 308.16, found: 308.17 $[M+Na]^{+}$, 324.13 $[M+K]^{+}$.

β-D-Galactopyranoside, methyl 6-deoxy-6-(4-(3,5-difluorophenyl)-1H-1,2,3-triazol-1-yl)-, 2,3,4-triacetate (5a)

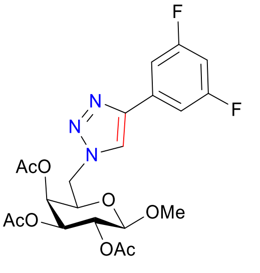

(**5a**)

Off white solid, yield: 85 %, ^{1}H NMR (400 MHz, $CDCl_3$, 25 ˚C, δ ppm): 7.92 (s, 1H), 7.34-7.29 (m, 2H), 6.80-6.75 (m, 1H), 5.52 (d, J = 4.0 Hz, 1H), 5.24 (dd, J = 8.0 Hz and 8.0 Hz, 1H), 5.08 (dd, ^{3}J = 8.0 Hz and 4.0 Hz, 1H), 4.69 (dd, ^{3}J = 12.0 Hz and 4.0 Hz, 1H), 4.52 (dd, ^{3}J = 12.0 Hz and 8.0 Hz, 1H), 4.37 (d, J = 8.0 Hz, 1H), 4.22 (dd, ^{3}J = 8.0 Hz and 4.0 Hz, 1H), 3.41 (s, 3H, $OC\underline{H}_3$), 2.21 (s, 3H, $OCOC\underline{H}_3$), 2.07 (s, 3H, $OCOC\underline{H}_3$), 2.00 (s, 3H, $OCOC\underline{H}_3$). $^{13}C\{^{1}H\}$ NMR (100 MHz, $CDCl_3$, 25 ˚C, δ ppm): 170.35 (s, $\underline{C}OCH_3$), 170.10 (s, $\underline{C}OCH_3$), 169.59 (s, $\underline{C}OCH_3$), 164.70 (d, J_{CF} = 13.0 Hz), 162.22 (d, J_{CF} = 13.0 Hz), 145.84 (t, J_{CF} = 4.0 Hz), 133.55 (t, J_{CF} = 11.0 Hz), 121.96 (s), 108.51 (d, J_{CF} = 26.0 Hz), 108.51 (d, J_{CF} = 12.0 Hz), 103.51 (t, J_{CF} = 26.0 Hz), 102.16 (C-1), 71.76 (C-5), 70.78 (C-3), 68.62 (C-2), 68.00 (C-4), 57.26 ($O\underline{C}H_3$), 50.49 (C-6), 20.81 (s, $CO\underline{C}H_3$), 20.73 (s, $CO\underline{C}H_3$), 20.59 (s, $CO\underline{C}H_3$). $^{19}F\{^{1}H\}$ NMR (376 MHz, $CDCl_3$, 25 ˚C, δ ppm): −108.98. MALDI-ToF-MS: m/z calcd. for $C_{21}H_{24}F_2N_3O_8^{+}$: 484.15, found: 484.15 $[M+H]^{+}$, 506.12 $[M+Na]^{+}$.

β-D-Galactopyranoside, methyl 6-deoxy-6-(4-(*m*-tolyl)-1H-1,2,3-triazol-1-yl)-, 2,3,4-triacetate (5b)

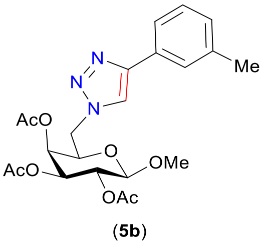

(**5b**)

Off white solid, yield: 88 %, ^{1}H NMR (400 MHz, $CDCl_3$, 25 ˚C, δ ppm): 7.86 (s, 1H), 7.68 (s, 1H), 7.56 (d, J = 8.0 Hz, 1H), 7.32-7.28 (m, 1H), 7.15 (d, J = 8.0 Hz, 1H), 5.49 (s, 1H), 5.23 (t, J = 8.0 Hz, 1H), 5.06 (d, J = 8.0 Hz, 1H), 4.65 (d, J = 16.0 Hz, 1H), 4.49 (dd, J = 12.0 Hz and 8.0 Hz, 1H), 4.33 (d, J = 8.0 Hz, 1H), 4.19 (d, J = 8.0 Hz, 1H), 3.39 (s, 3H, $OC\underline{H}_3$), 2.39 (s, 3H, $C\underline{H}_3$), 2.21 (s, 3H, $OCOC\underline{H}_3$), 2.06 (s, 3H, $OCOC\underline{H}_3$)), 2.00 (s, 3H, $OCOC\underline{H}_3$). $^{13}C\{^{1}H\}$ NMR (100 MHz, $CDCl_3$, 25 ˚C, δ ppm): 170.33 (s, $\underline{C}OCH_3$), 170.08 (s, $\underline{C}OCH_3$), 169.61 (s, $\underline{C}OCH_3$), 147.92, 138.69, 130.28, 129.12, 128.87, 126.39, 122.79, 121.15, 102.13 (C-1), 71.91 (C-5), 70.81 (C-3), 68.68 (C-2), 68.04 (C-4), 57.30 ($O\underline{C}H_3$), 50.37 (C-6), 21.50 ($\underline{C}H_3$), 20.86 (s, $CO\underline{C}H_3$), 20.78 (s,

CO$\underline{C}H_3$), 20.62 (s, CO$\underline{C}H_3$). MALDI-ToF-MS: *m/z* calcd. for $C_{22}H_{28}N_3O_8^+$: 462.19, found: 462.23 $[M+H]^+$, 484.20 $[M+Na]^+$, 500.16 $[M+K]^+$.

1-(Anthracen-9-ylmethyl)-4-benzyl-1H-1,2,3-triazole (6a)

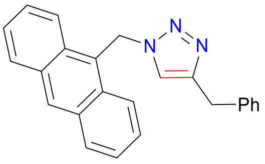

(**6a**)

Yellow solid, yield: 93 %, ^1H NMR (400 MHz, $CDCl_3$, 25 ˚C, δ ppm): 8.56 (s, 1H), 8.29 (d, J = 8.0 Hz, 2H), 8.06 (d, J = 8.0 Hz, 2H), 7.58 (t, J = 8.0 Hz, 2H), 7.51 (t, J = 8.0 Hz, 2H), 7.20-7.17 (m, 2H), 7.14-7.10 (m, 3H), 6.85 (s, 1H), 6.48 (s, 2H), 3.91 (s, 2H). $^{13}C\{^1H\}$ NMR (100 MHz, $CDCl_3$, 25 ˚C, δ ppm): 139.07, 131.48, 130.86, 129.83, 129.50, 128.62, 128.52, 127.64, 126.38, 125.45, 124.02, 123.13, 46.48, 32.18. MALDI-ToF-MS: *m/z* calcd. for $C_{24}H_{19}N_3^+$: 349.16, found: 349.11 $[M]^+$, 372.11 $[M+Na]^+$.

1-(Anthracen-9-ylmethyl)-4-phenyl-1H-1,2,3-triazole (6b)[56]

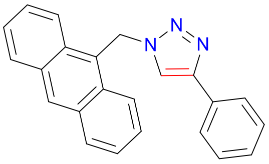

(**6b**)

Yellow solid, yield: 90 %, ^1H NMR (400 MHz, $CDCl_3$, 25 ˚C, δ ppm): 8.61 (s, 1H), 8.36 (d, J = 12.0 Hz, 2H), 8.10 (d, J = 8.0 Hz, 2H), 7.64-7.60 (m, 4H), 7.56-7.53 (m, 2H), 7.30-7.26 (m, 3H), 7.23-7.20 (m, 1H), 6.59 (s, 2H). $^{13}C\{^1H\}$ NMR (100 MHz, $CDCl_3$, 25 ˚C, δ ppm): 147.95, 131.59, 130.96, 130.54, 130.04, 129.62, 128.75, 128.11, 127.86, 125.69, 125.58, 123.85, 123.10, 119.20, 46.63. MALDI-ToF-MS: *m/z* calcd. for $C_{23}H_{17}N_3^+$: 335.14, found: 335.64 $[M]^+$, 358.58 $[M+Na]^+$.

1-(Anthracen-9-ylmethyl)-4-(*m*-tolyl)-1H-1,2,3-triazole (6c)

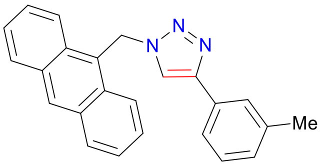

(**6c**)

Yellow solid, yield: 93 %, ^1H NMR (400 MHz, $CDCl_3$, 25 ˚C, δ ppm): 8.58 (s, 1H), 8.34 (d, J = 12.0 Hz, 2H), 8.08 (d, J = 8.0 Hz, 2H), 7.61 (t, J = 8.0 Hz, 2H), 7.53 (t, J = 8.0 Hz, 2H), 7.48 (s, 1H), 7.40 (d, J = 8.0 Hz, 1H), 7.16 (t, J = 8.0 Hz, 1H), 7.03 (d, J = 8.0 Hz, 1H), 6.55 (s, 2H, C$\underline{H}_2$), 2.28 (s, 3H, C$\underline{H}_3$). $^{13}C\{^1H\}$ NMR (100 MHz, $CDCl_3$, 25 ˚C, δ ppm): 148.06, 138.41, 131.57, 130.96, 130.40, 130.00, 129.60, 128.88, 128.63, 127.83, 126.33, 125.56, 123.87, 123.11, 122.79, 119.14, 46.61, 21.40. MALDI-ToF-MS: *m/z* calcd. for $C_{24}H_{19}N_3^+$: 349.16, found: 349.18 $[M]^+$, 372.15 $[M+Na]^+$.

4-Benzyl-1-phenyl-1H-1,2,3-triazole (7a)[57]

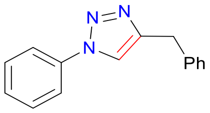

(7a) S

Off white solid, yield: 86 %, ^{1}H NMR (400 MHz, $CDCl_3$, 25 ˚C, δ ppm): 7.68 (d, J = 8.0 Hz, 2H), 7.59 (s, 1H), 7.48 (t, J = 8.0 Hz, 2H), 7.39 (t, J = 8.0 Hz, 1H), 7.35-7.32 (m, 4H), 7.28-7.23 (m, 1H), 4.17 (s, 2H). ^{13}C{^{1}H} NMR (100 MHz, $CDCl_3$, 25 ˚C, δ ppm): 148.62, 138.95, 137.29, 129.78, 128.92, 128.84, 128.65, 126.76, 120.53, 119.78, 32.44. MALDI-ToF-MS: *m/z* calcd. for $C_{15}H_{14}N_3^+$: 236.12, found: 236.07 $[M+H]^+$, 258.44 $[M+Na]^+$.

2.4 Cell viability study

Cytotoxicity of Cu(I)-NHC complex (**2**) was evaluated using CCK-8 assay. 10,000 HeLa cells/well were seeded in a 96-well plate in complete DMEM media and cultured for 24 h. Later, the media was replaced with fresh media and varying concentrations of Cu(I)-NHC complex (**2**) prepared as a DMSO stock solution was added so that the final concentration of DMSO remained 1% and cultured for respective time. After that, the medium was removed and the cells were washed with PBS followed by addition of media (100 µL) and CCK-8 (10 µL) were added to each well and incubated further for 3 h at 37 °C. After 3 h, the plate was taken out and absorbance was recorded at 450 nm in a microplate reader to estimate cell viability. For each concentration, three technical replicates were performed. Polymer (**8**) encapsulated Cu(I)-NHC complex (**2**) was prepared with 1:10 molar ratio of polymer:Cu(I) as stated in the general procedure above at 480 µM of Cu(I) and further diluted to the study concentrations (Fig. S13).

3. Characterization data

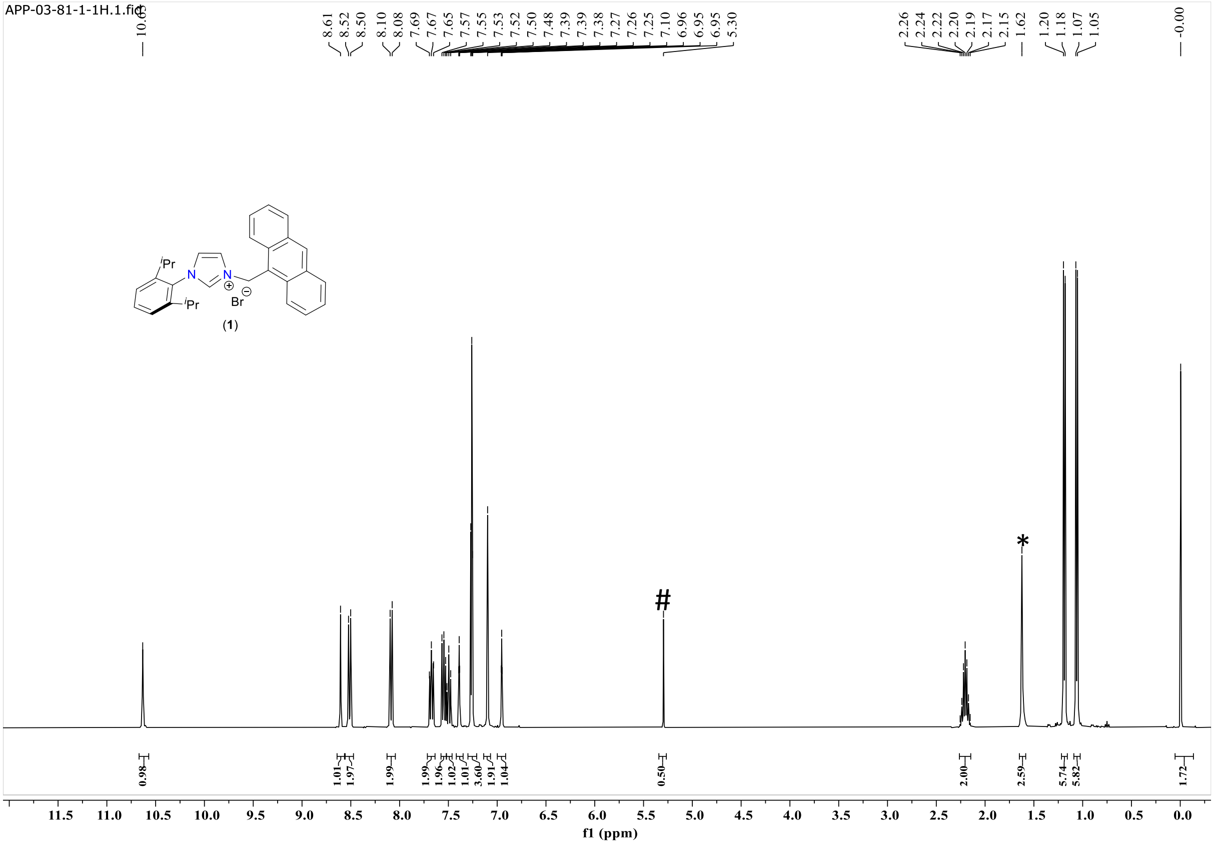

Figure S1. ^{1}H NMR of pre-NHC ligand (**1**) in $CDCl_3$. # DCM, * H_2O.

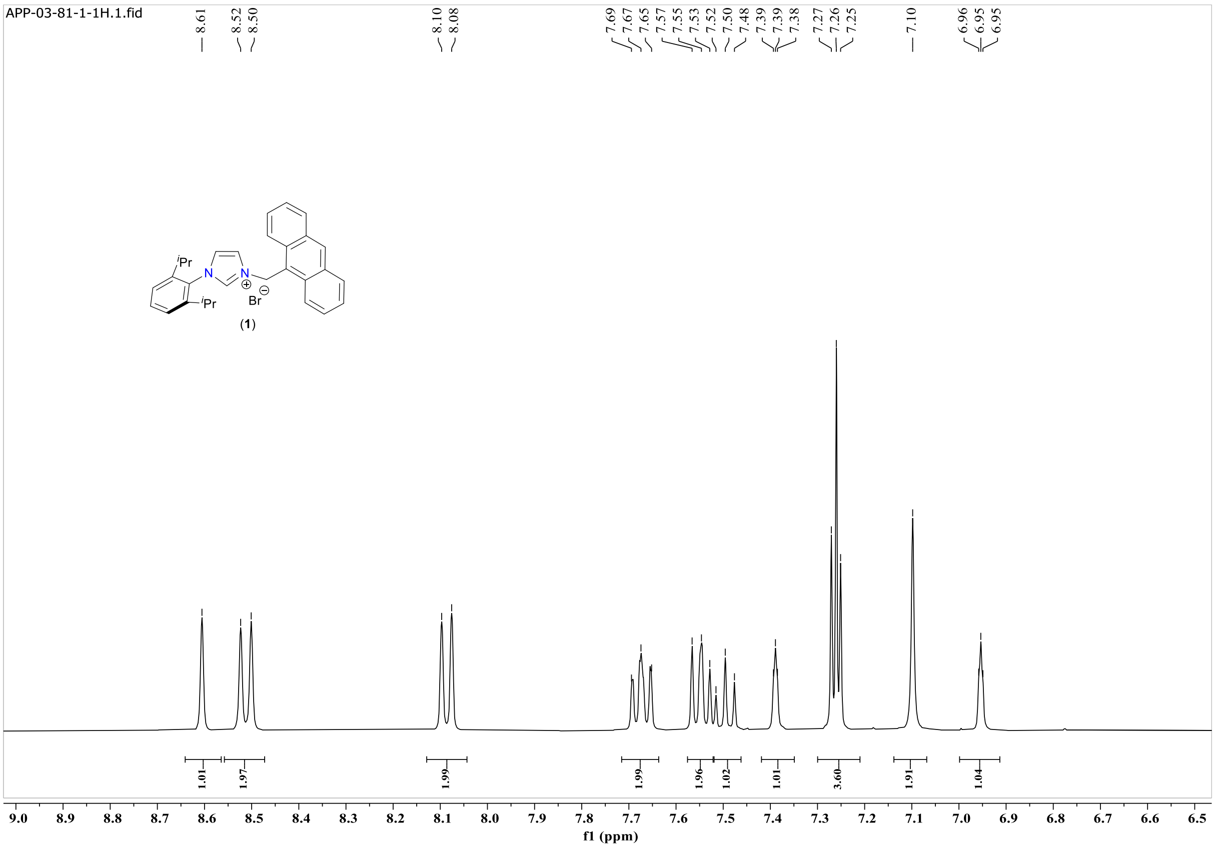

Figure S2. Expanded ^{1}H NMR of pre-NHC ligand (**1**) in $CDCl_3$.

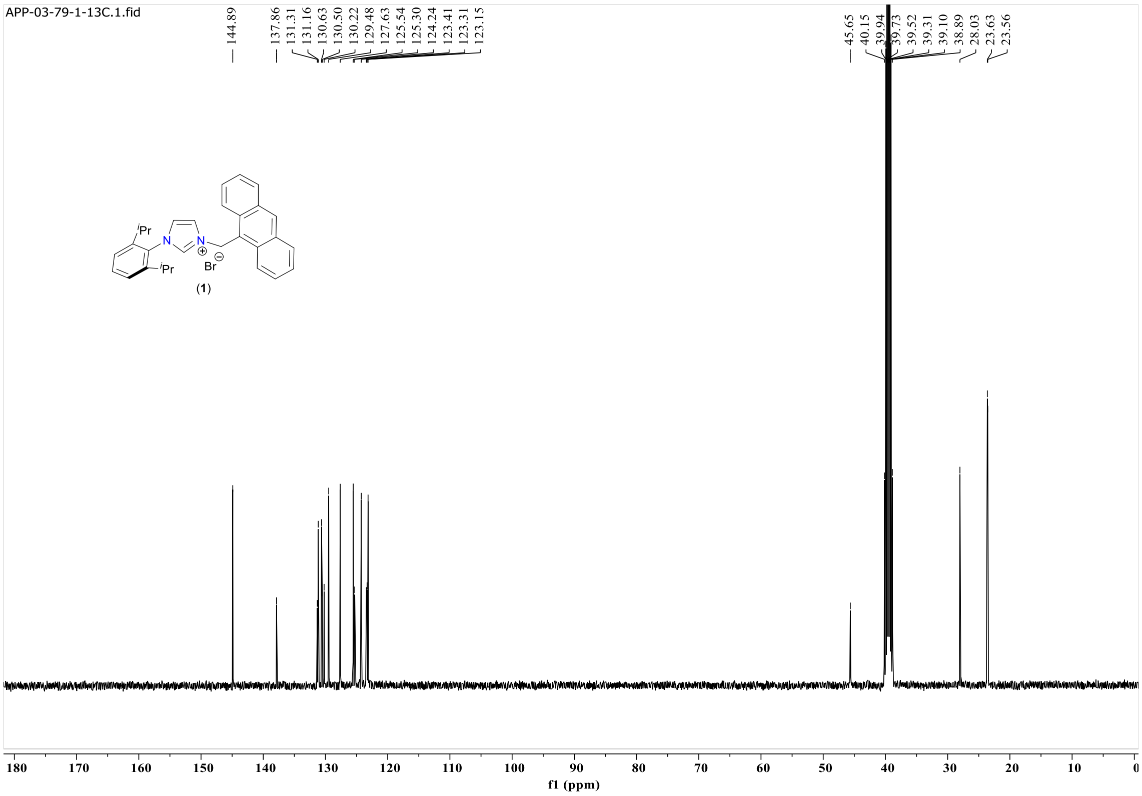

Figure S3. $^{13}C\{^{1}H\}$ NMR of pre-NHC ligand (**1**) in DMSO-d_6.

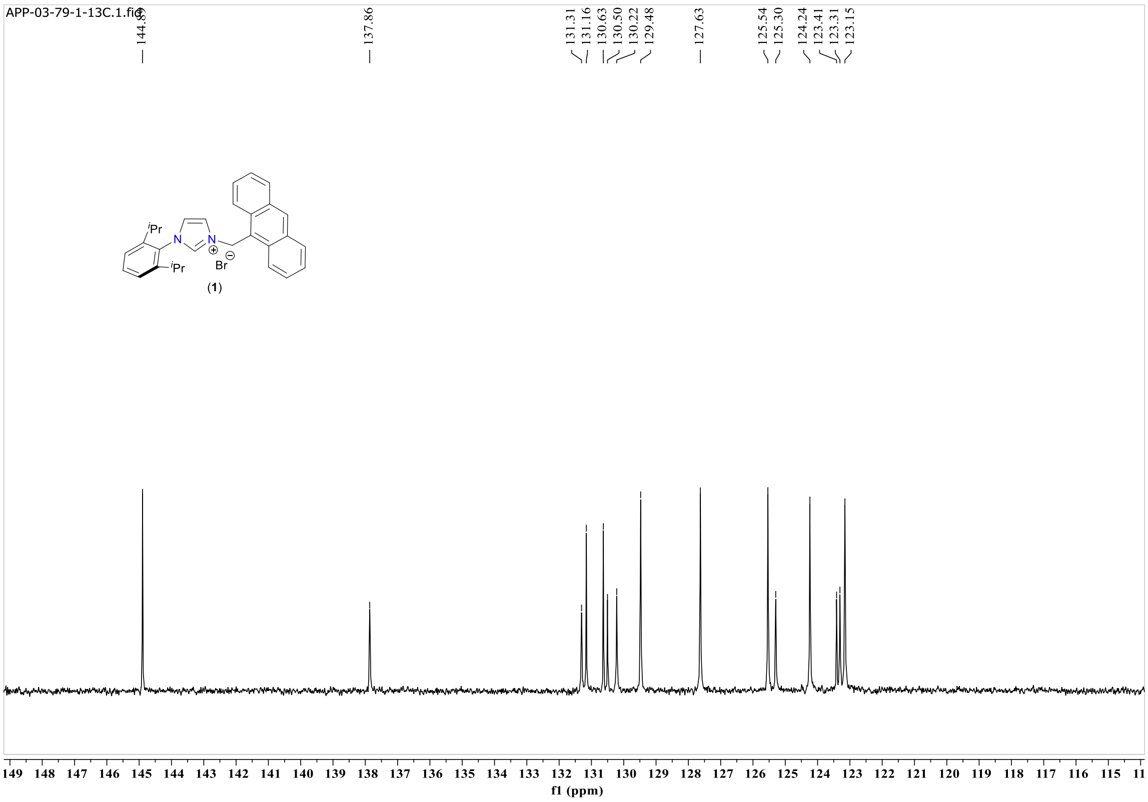

Figure S4. Expanded $^{13}C\{^{1}H\}$ NMR of pre-NHC ligand (**1**) in DMSO-d_6.

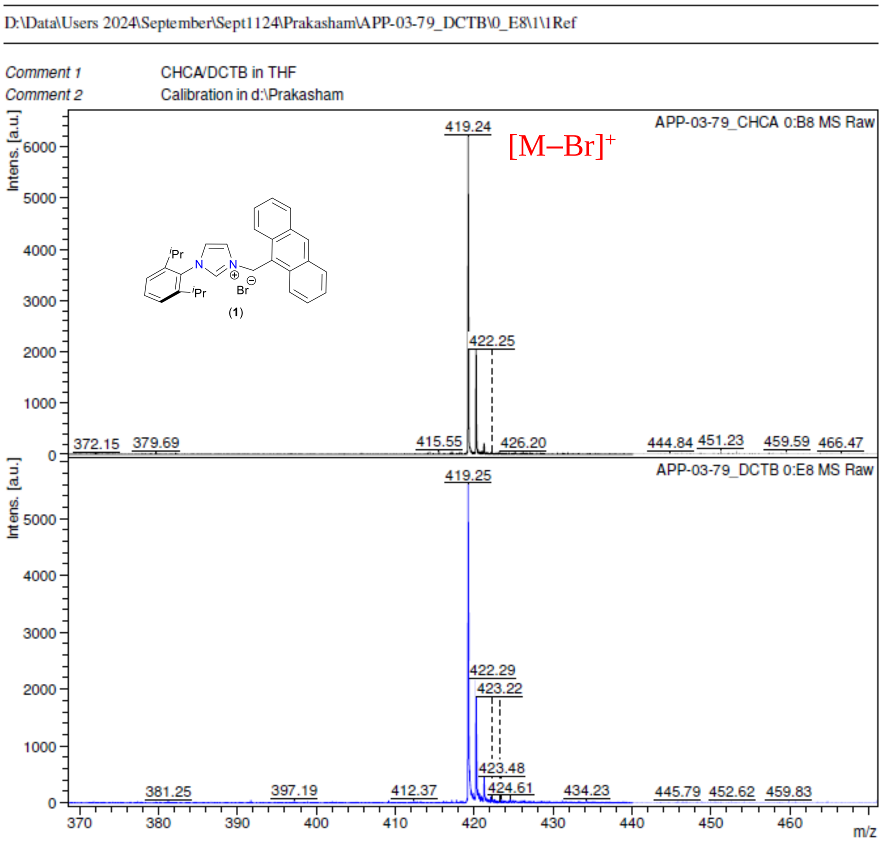

Figure S5. MALDI-ToF-MS spectra of pre-NHC ligand (**1**).

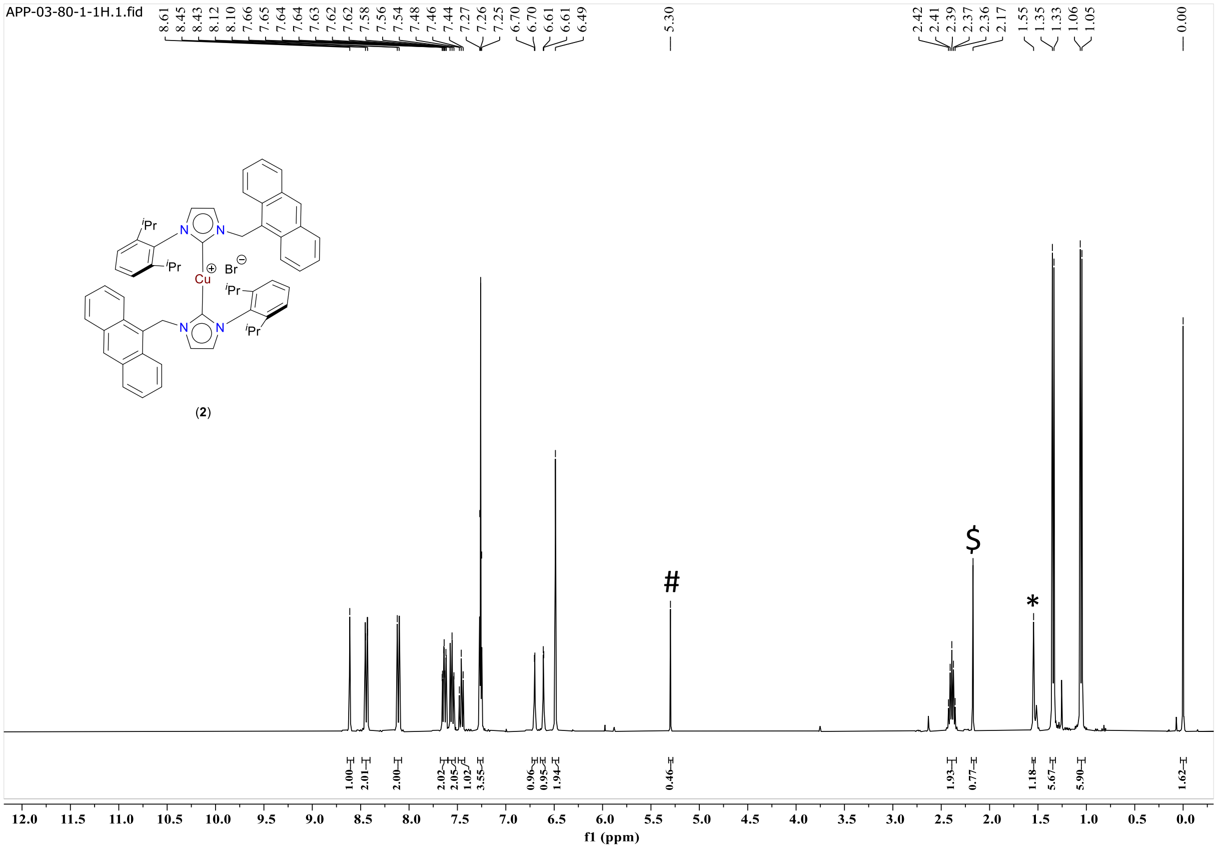

Figure S6. ^{1}H NMR of Cu(I)-NHC complex (**2**) in $CDCl_3$. # DCM, $ acetone,* H_2O.

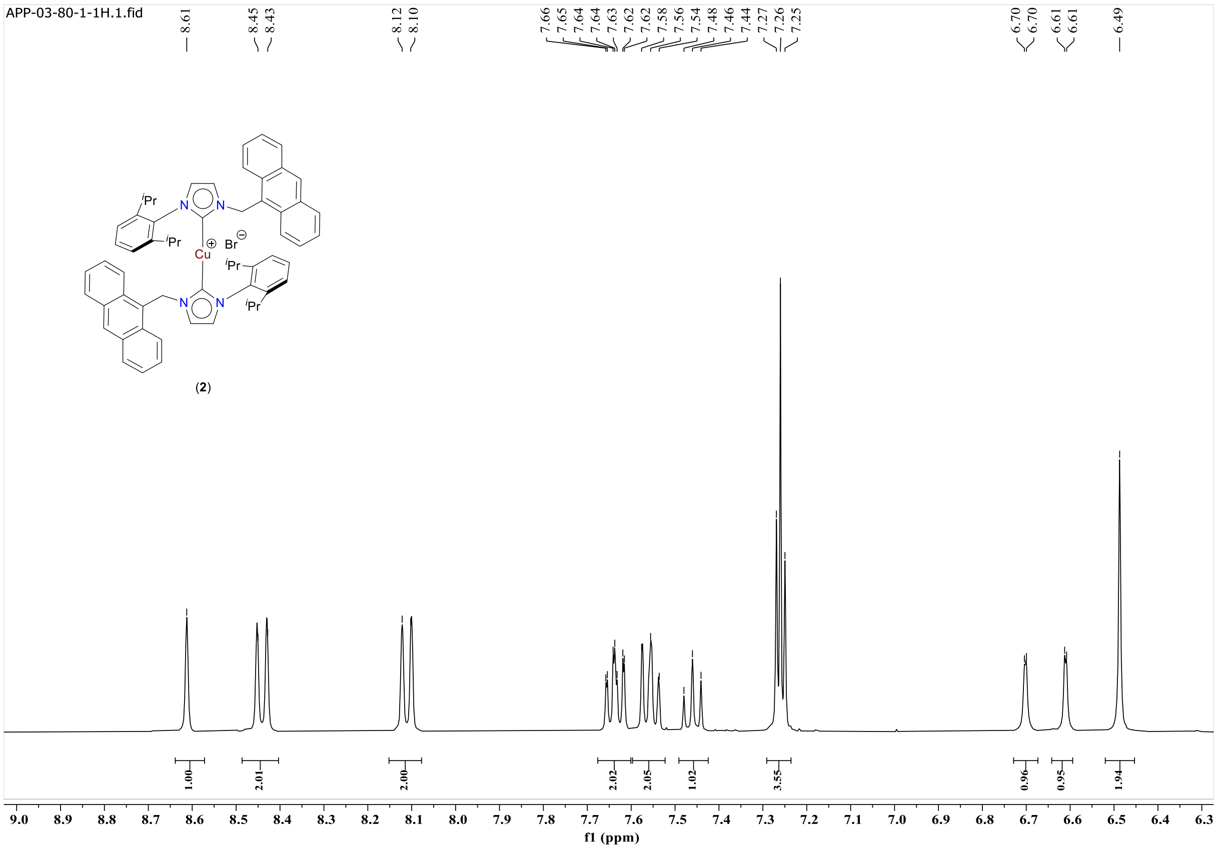

Figure S7. Expanded ^{1}H NMR of Cu(I)-NHC complex (**2**) in $CDCl_3$.

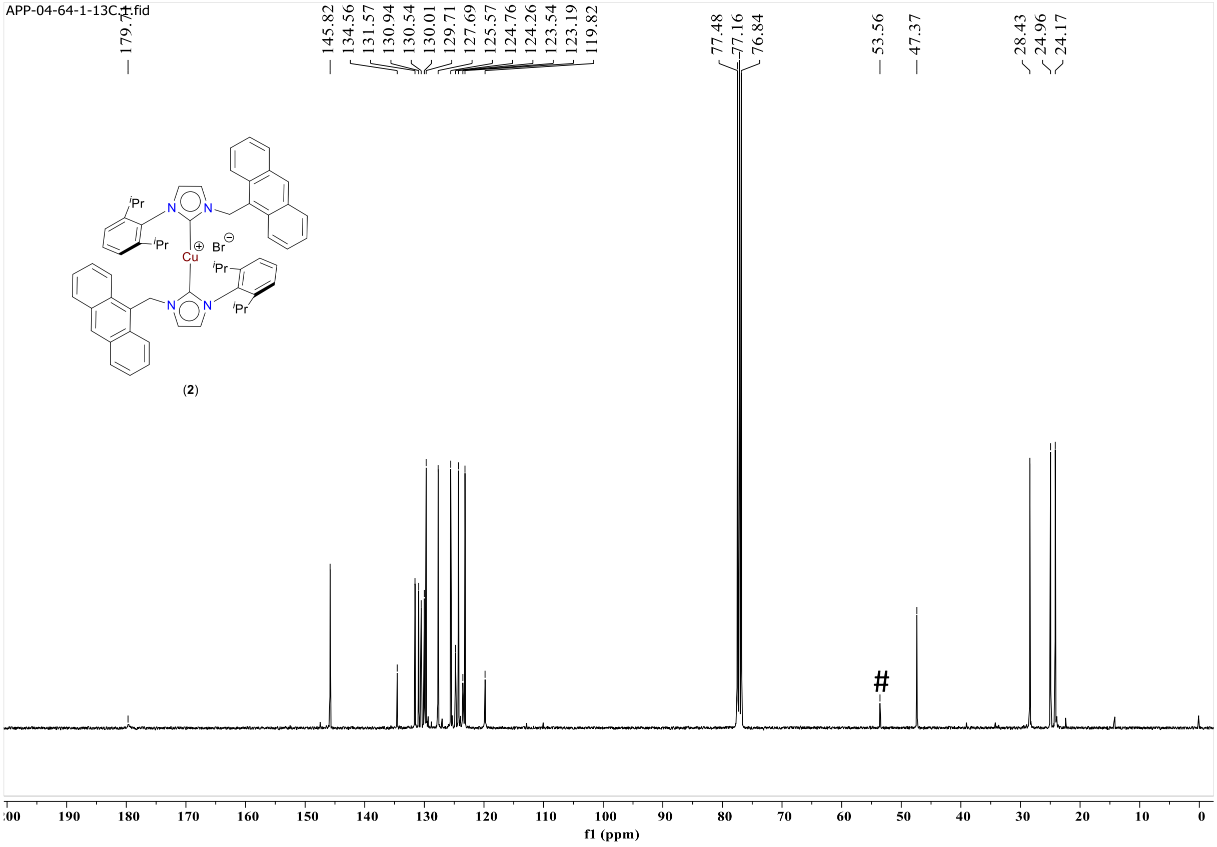

Figure S8. $^{13}C\{^{1}H\}$ NMR of Cu(I)-NHC complex (**2**) in $CDCl_3$, # DCM.

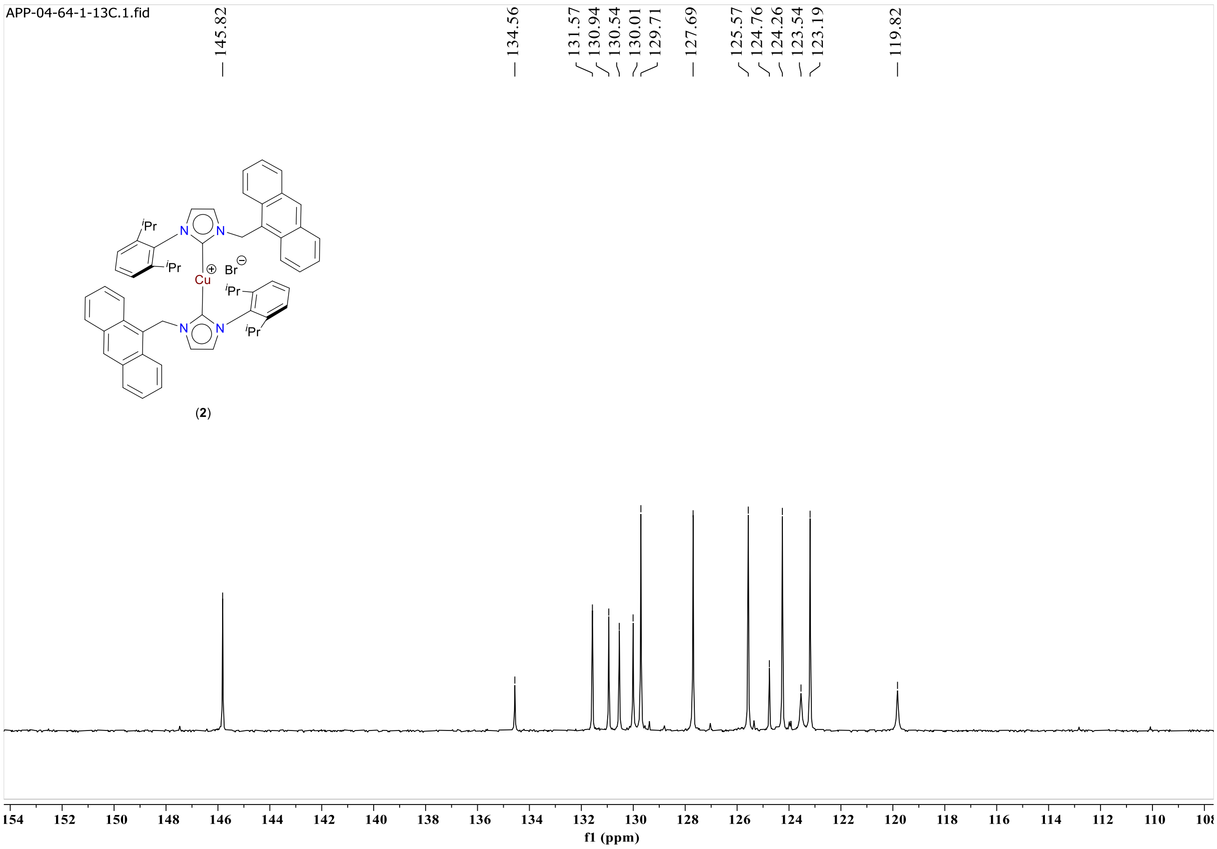

Figure S9. Expanded $^{13}C\{^{1}H\}$ NMR of Cu(I)-NHC complex (**2**) in $CDCl_3$.

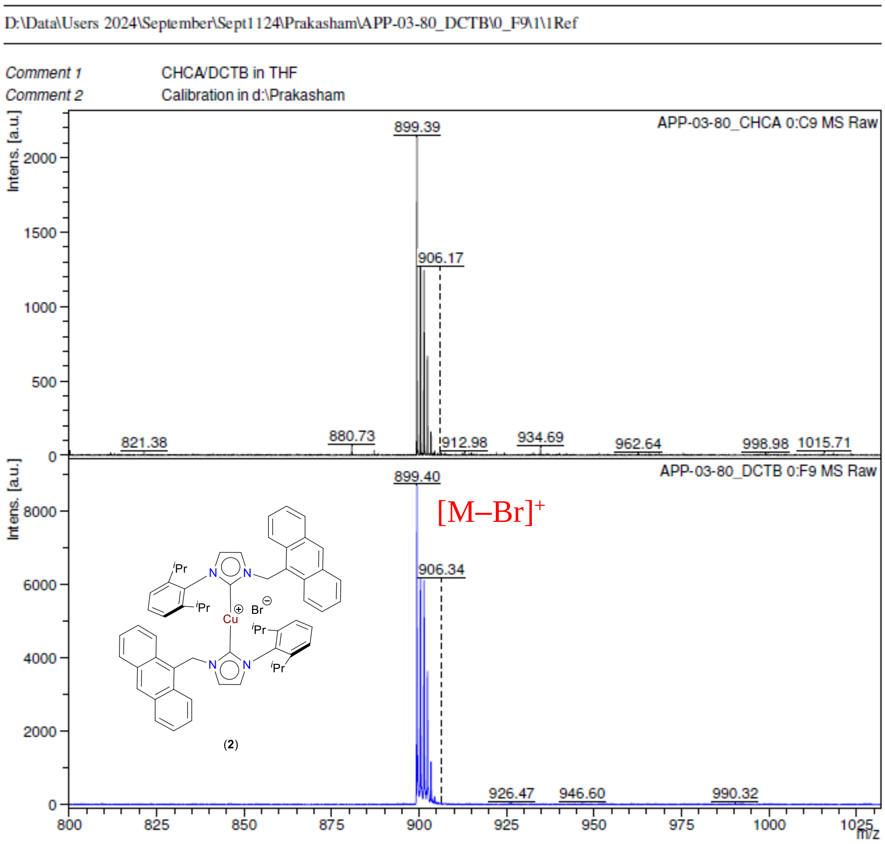

Figure S10. MALDI-ToF-MS spectra of Cu(I)-NHC complex (**2**).

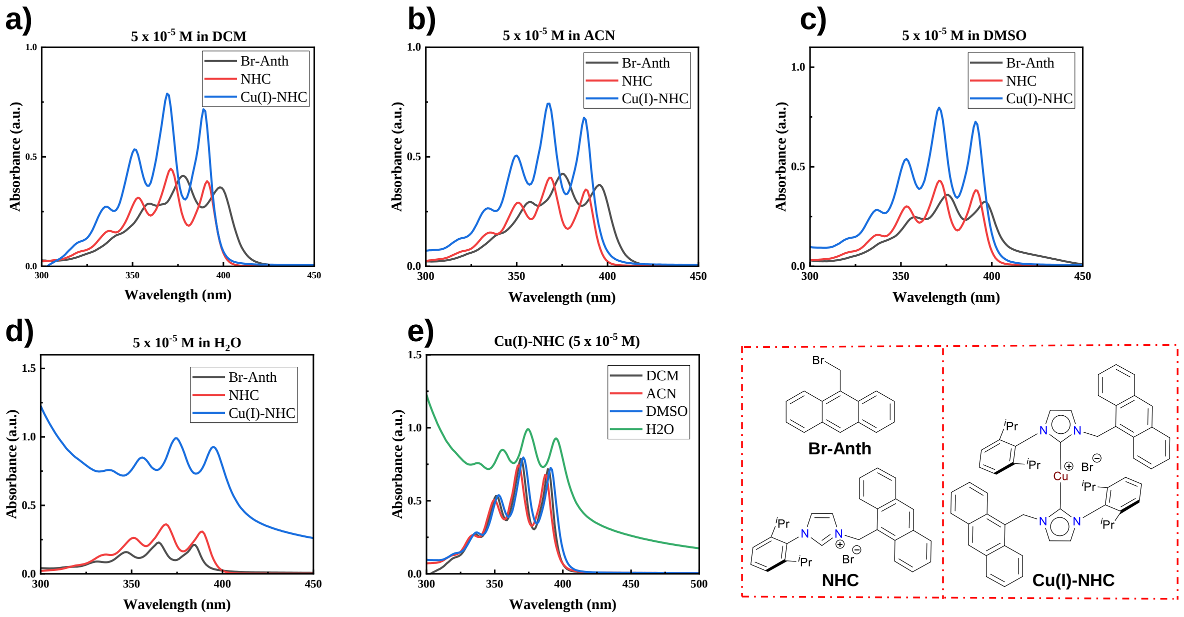

Figure S11. UV-Vis absorption spectra of 9-(bromomethyl)anthracene, pre-NHC ligand (**1**) and Cu(I)-NHC complex (**2**) at 5×10^{-5} M solutions in a) DCM, b) ACN, c) DMSO, d) H_2O (prepared from their respective DMSO stock solutions), e) an overlay of absorption spectra of Cu(I)-NHC complex (**2**) in various solvents.

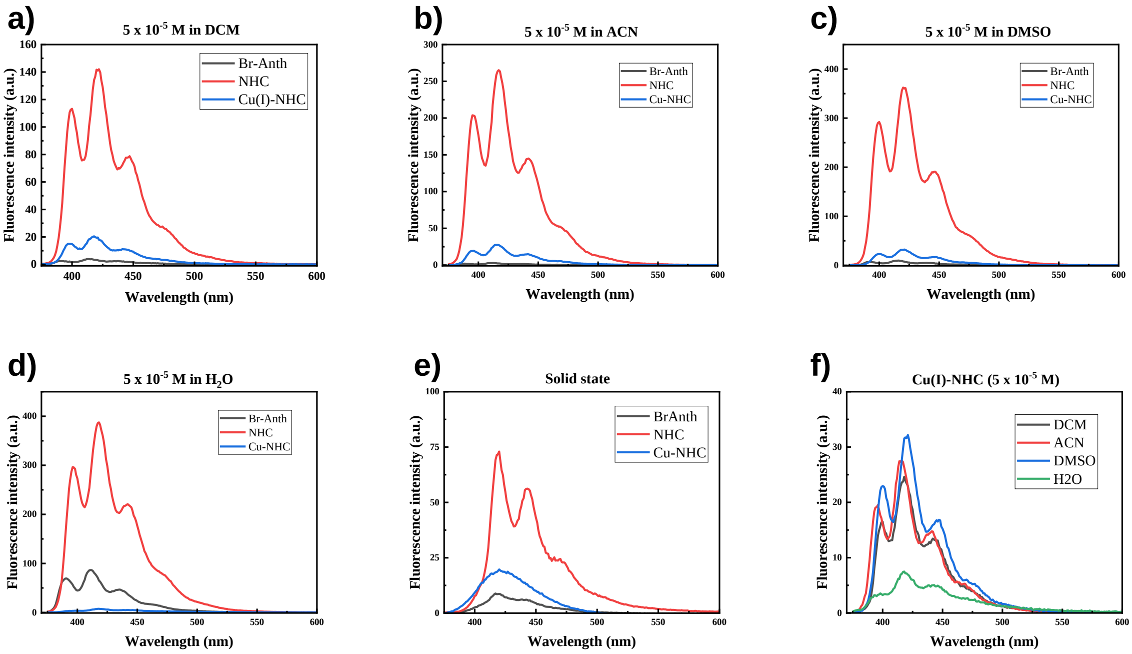

Figure S12. Fluorescence emission (λ_{ex} = 360 nm) spectra of 9-(bromomethyl)anthracene, pre-NHC ligand (**1**) and Cu(I)-NHC complex (**2**) at 5×10^{-5} M solutions in a) DCM, b) ACN, c) DMSO, d) H_2O (prepared from their respective DMSO stock solutions), e) solid state, f) an overlay of emission spectra of Cu(I)-NHC complex (**2**) in various solvents.

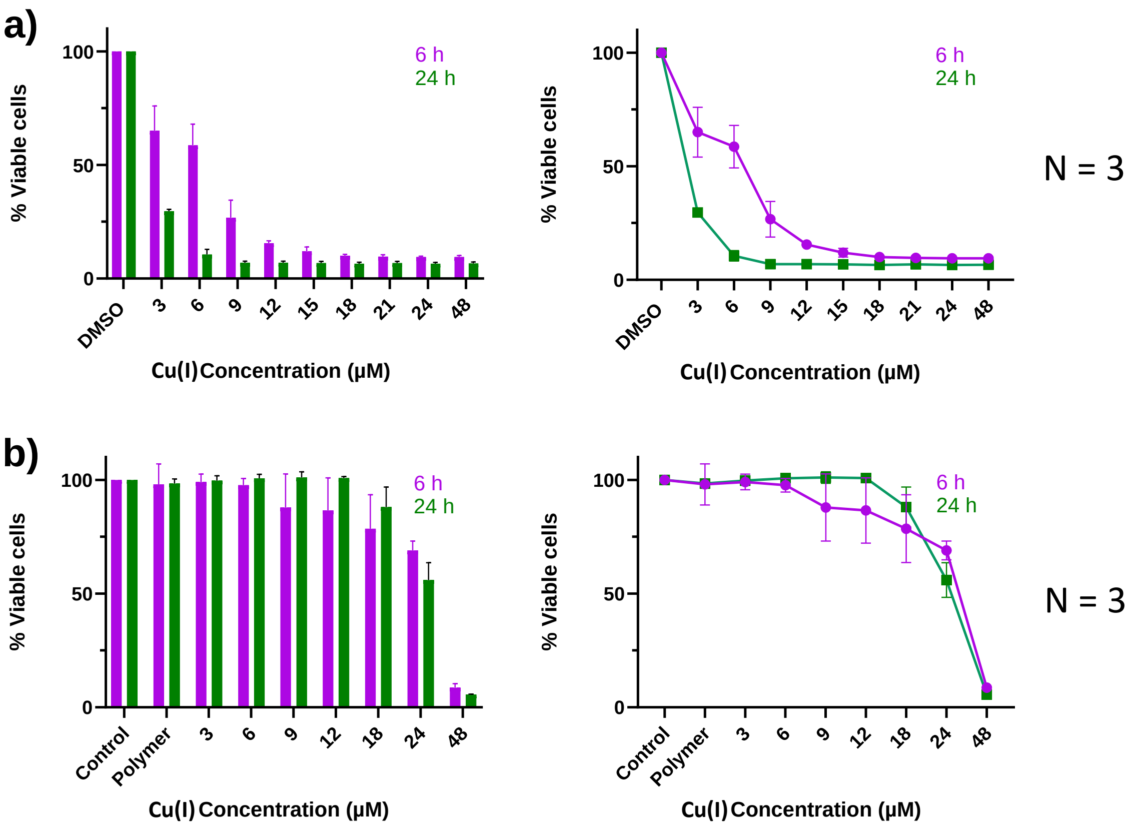

Figure S13. Cell viability assay with a) Cu(I)-NHC complex (**2**) b) Cu(I)-NHC complex (**2**) encapsulated in polymer (**8**), 1:10 molar ratio of polymer : Cu(I) at 6 h and 24 h; the amount of viable cells was analysed by CCK-8 assays in replicates of 3.

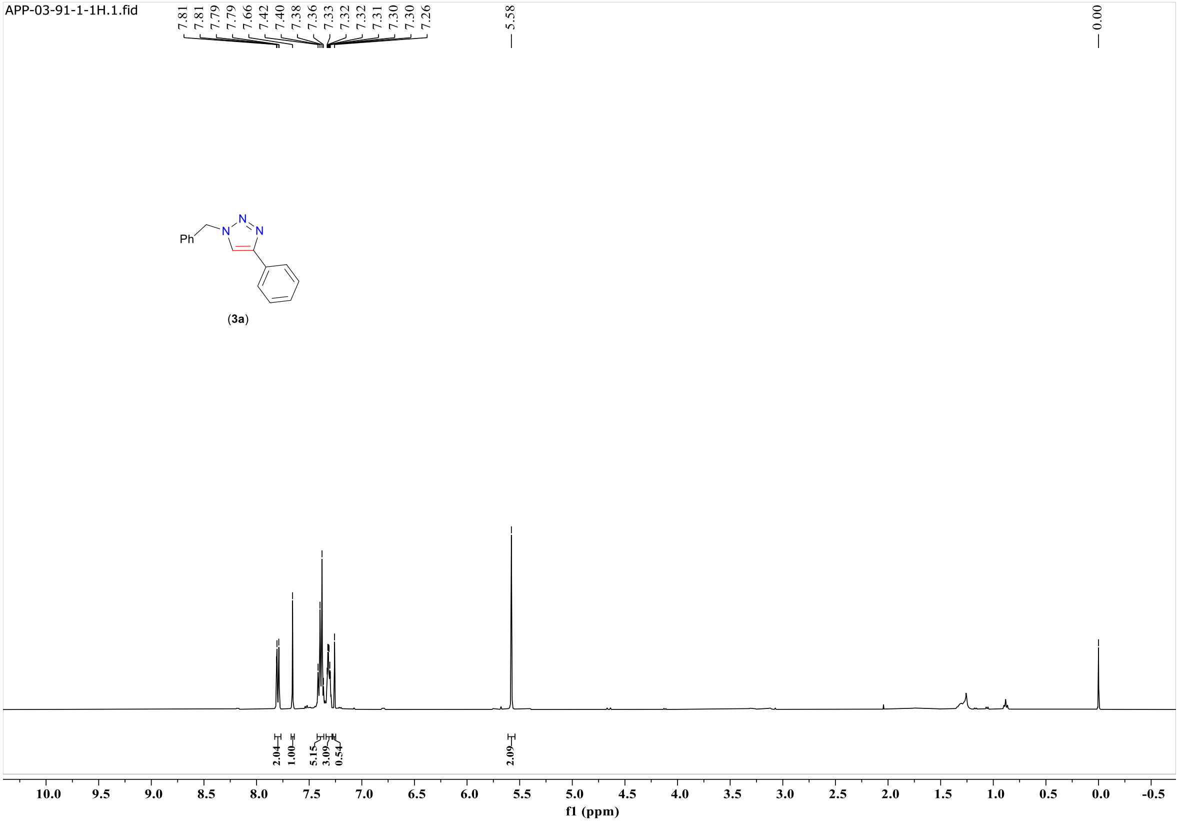

Figure S14. ^{1}H NMR of 1-benzyl-4-phenyl-1H-1,2,3-triazole (**3a**) in $CDCl_3$.

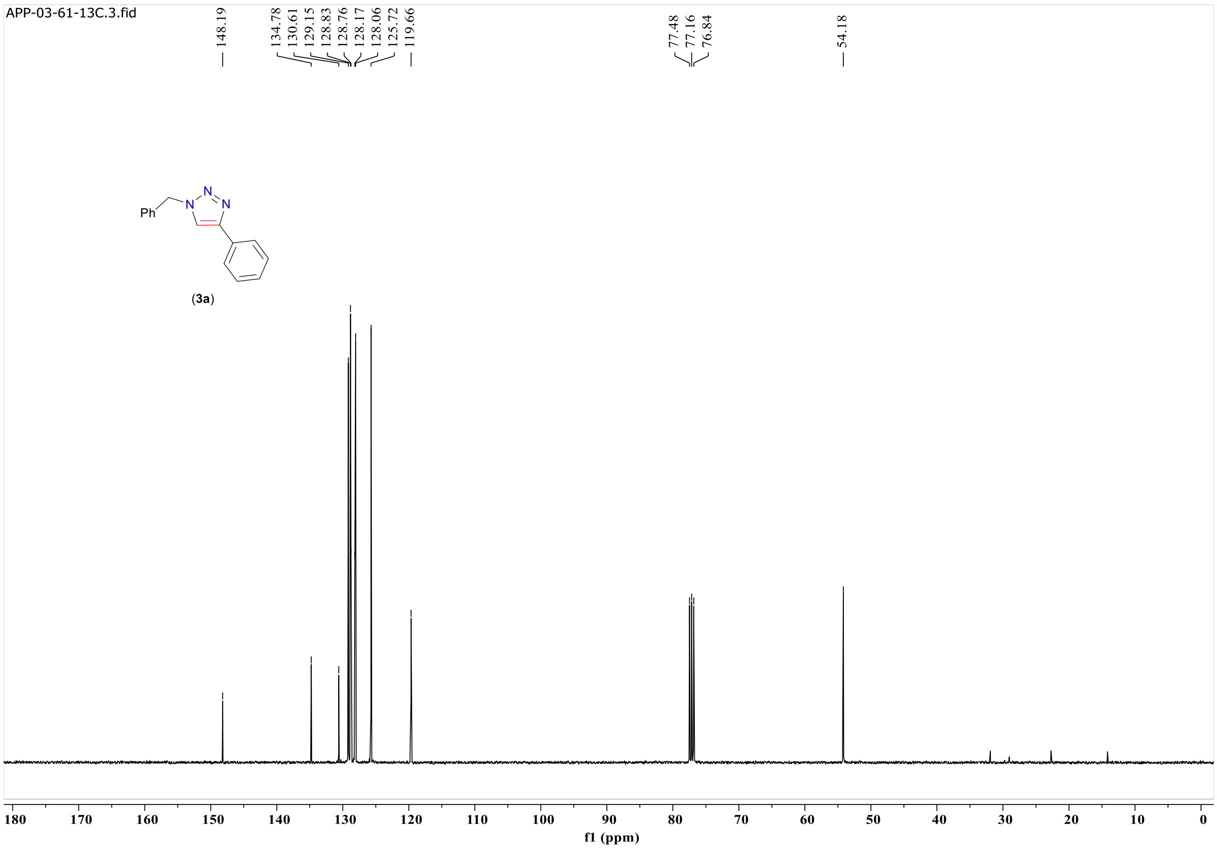

Figure S15. $^{13}C\{^1H\}$ NMR of 1-benzyl-4-phenyl-1H-1,2,3-triazole (**3a**) in $CDCl_3$.

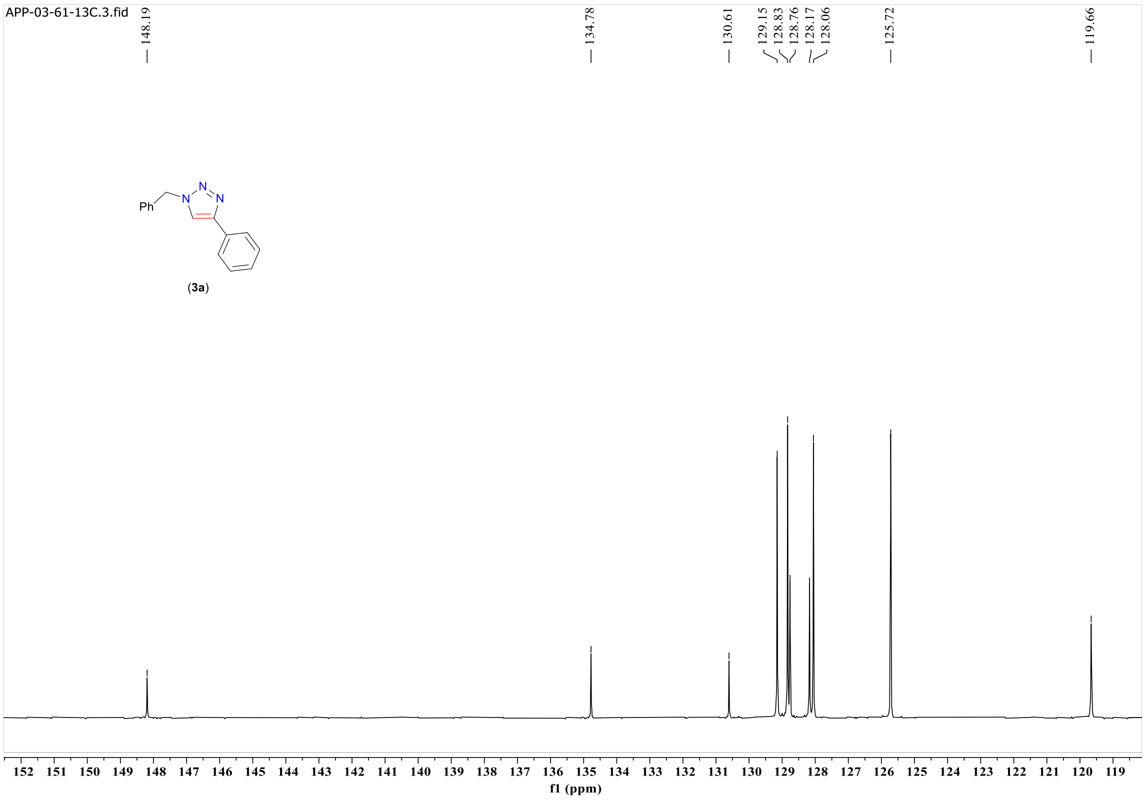

Figure S16. Expanded $^{13}C\{^1H\}$ NMR of 1-benzyl-4-phenyl-1H-1,2,3-triazole (**3a**) in $CDCl_3$.

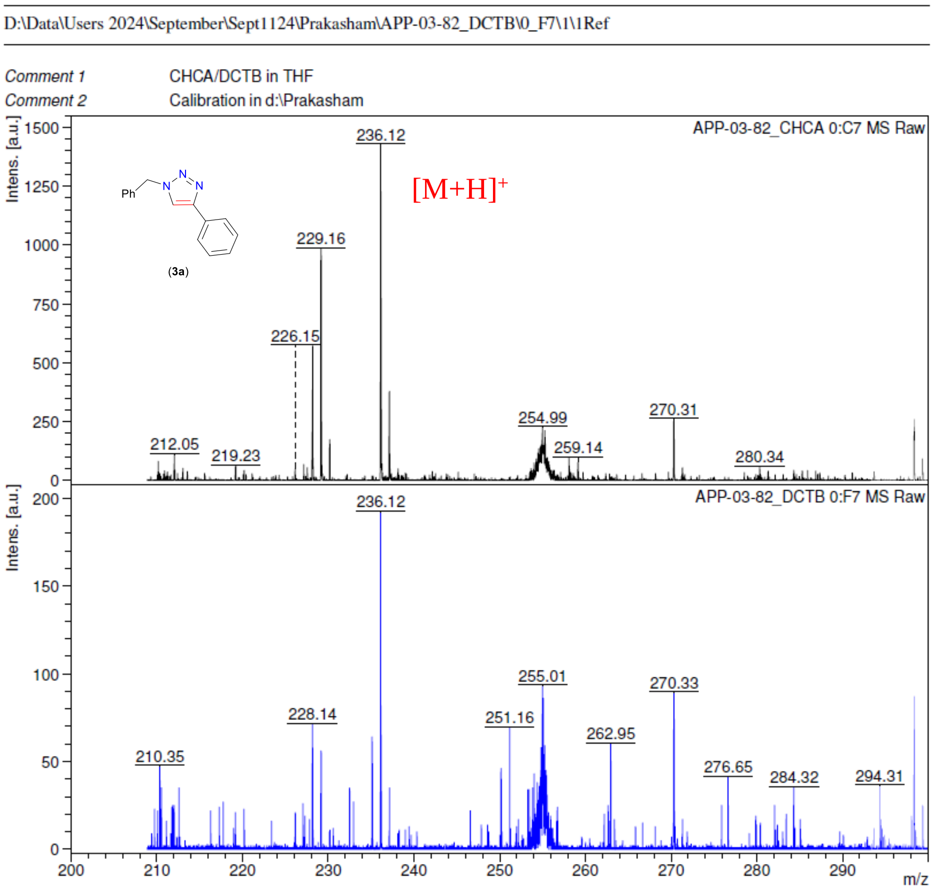

Figure S17. MALDI-ToF-MS spectra of 1-benzyl-4-phenyl-1H-1,2,3-triazole (**3a**).

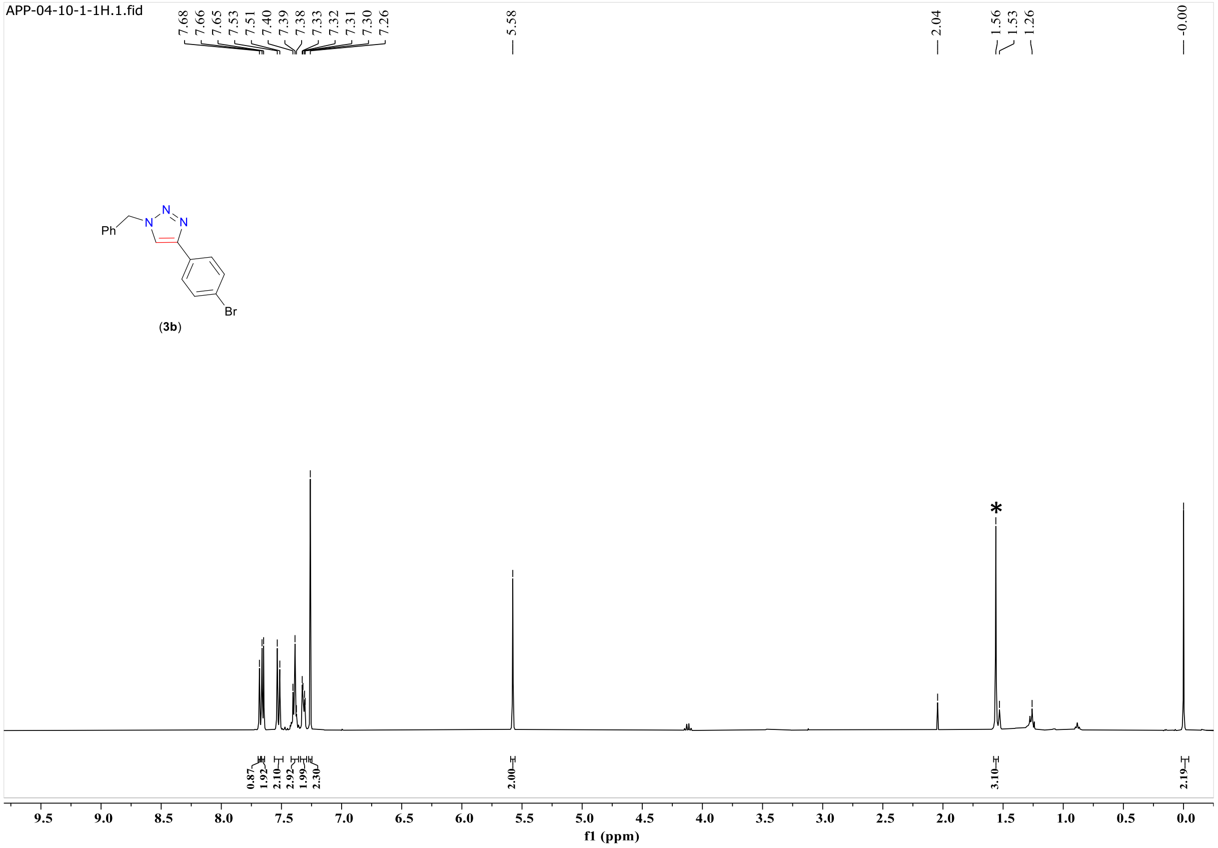

Figure S18. ^{1}H NMR of 1-benzyl-4-(4-bromophenyl)-1H-1,2,3-triazole (**3b**) in $CDCl_3$. * H_2O.

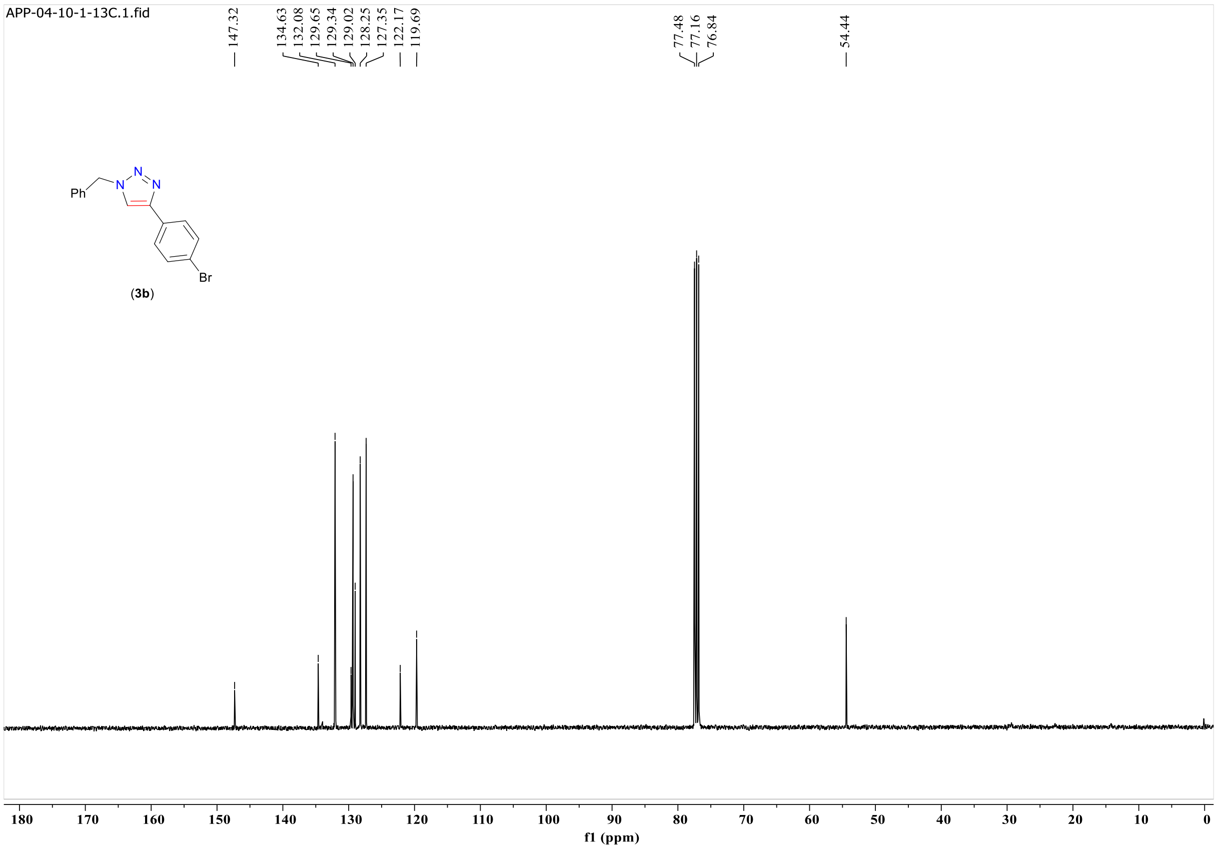

Figure S19. $^{13}C\{^{1}H\}$ NMR of 1-benzyl-4-(4-bromophenyl)-1H-1,2,3-triazole (**3b**) in $CDCl_3$.

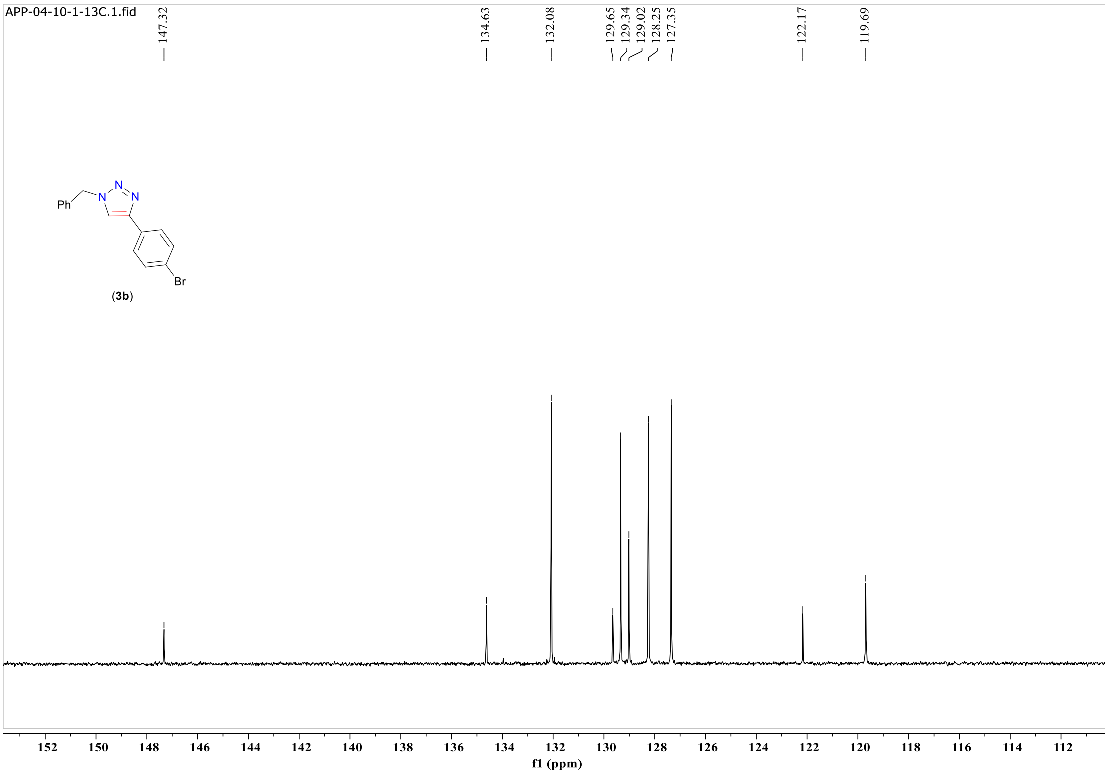

Figure S20. Expanded $^{13}C\{^{1}H\}$ NMR of 1-benzyl-4-(4-bromophenyl)-1H-1,2,3-triazole (**3b**) in $CDCl_3$.

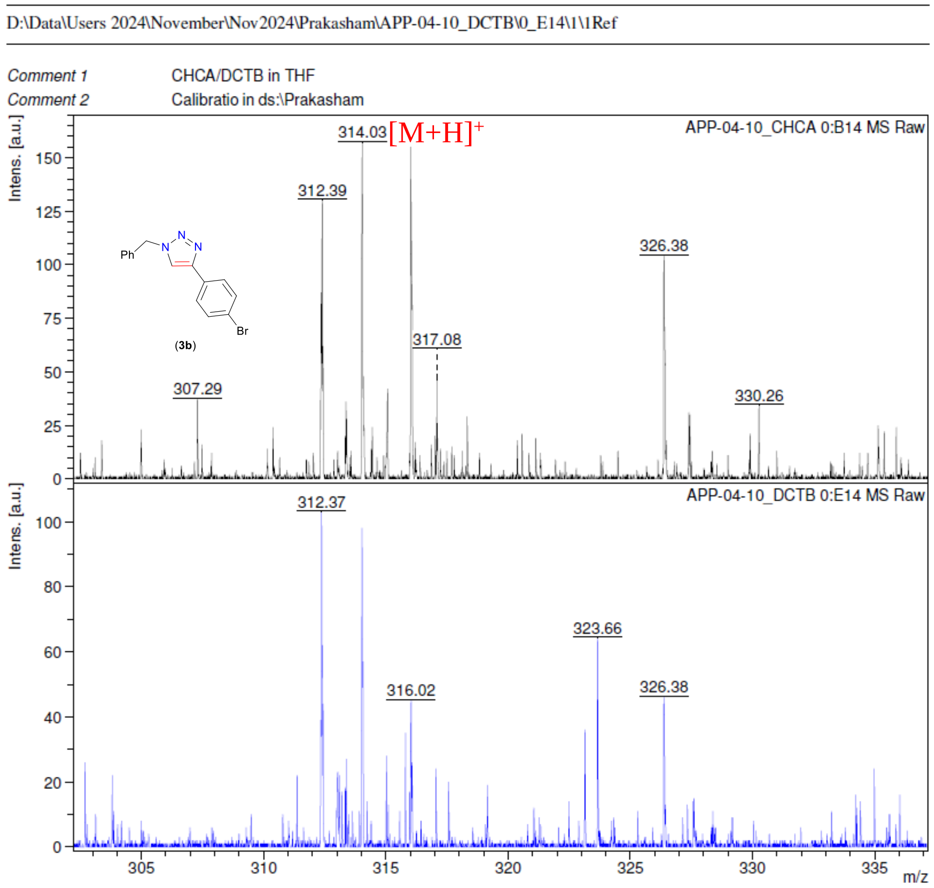

Figure S21. MALDI-ToF-MS spectra of 1-benzyl-4-(4-bromophenyl)-1H-1,2,3-triazole (**3b**).

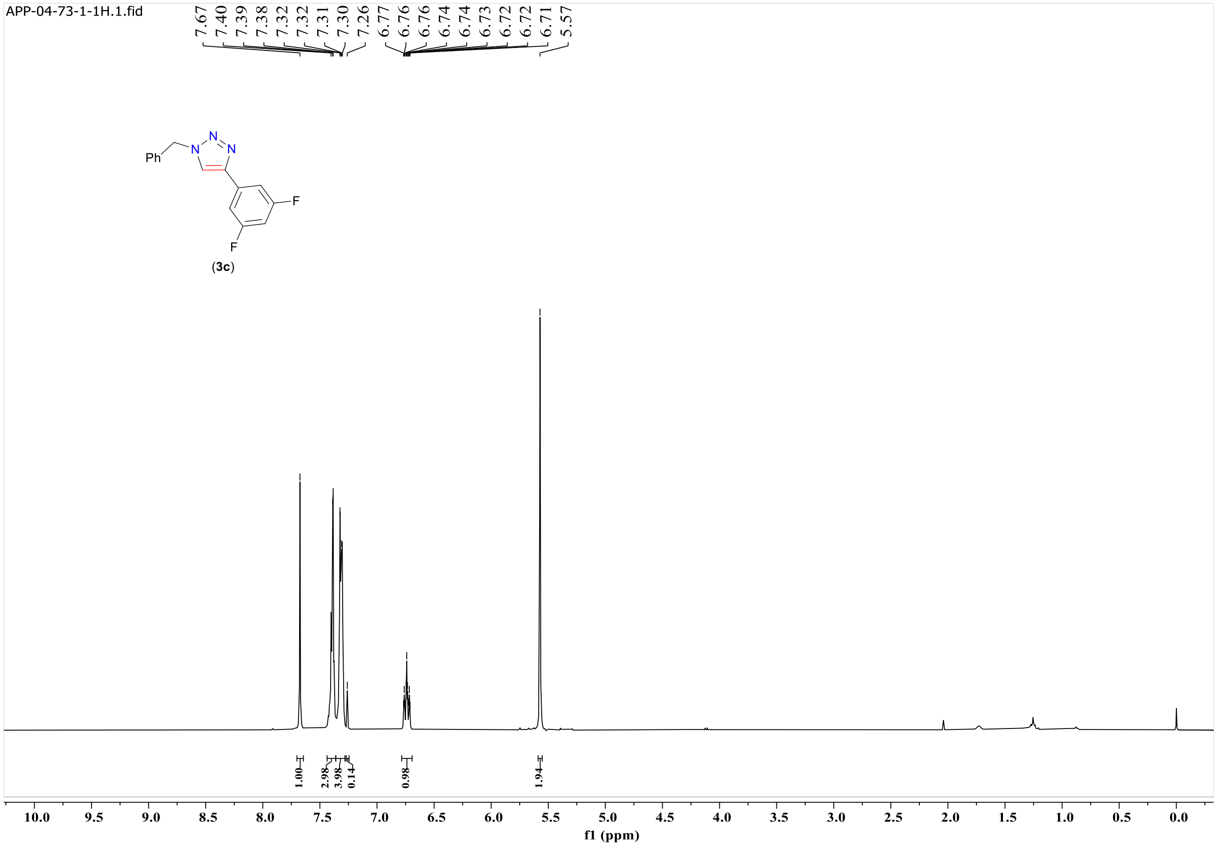

Figure S22. ^{1}H NMR of 1-benzyl-4-(3,5-difluorophenyl)-1H-1,2,3-triazole (**3c**) in $CDCl_3$.

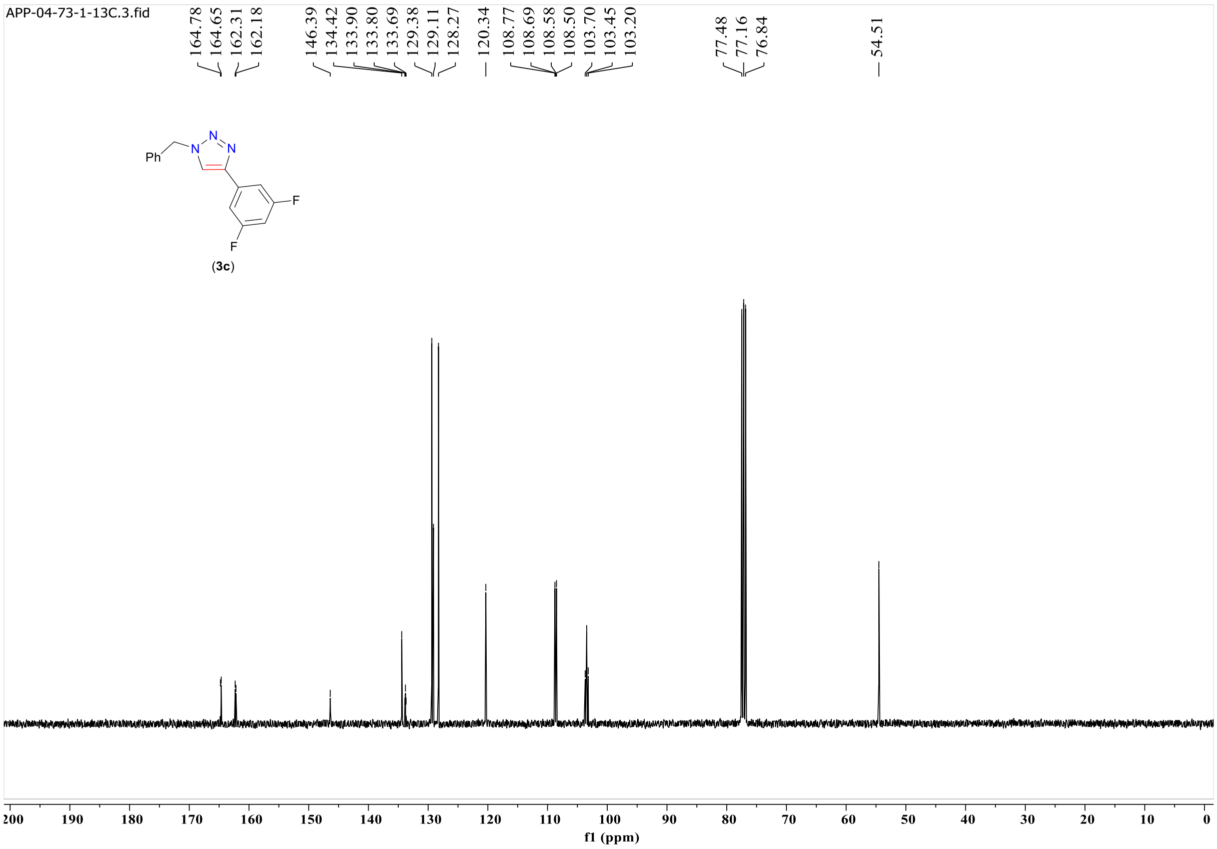

Figure S23. $^{13}C\{^1H\}$ NMR of 1-benzyl-4-(3,5-difluorophenyl)-1H-1,2,3-triazole (**3c**) in $CDCl_3$.

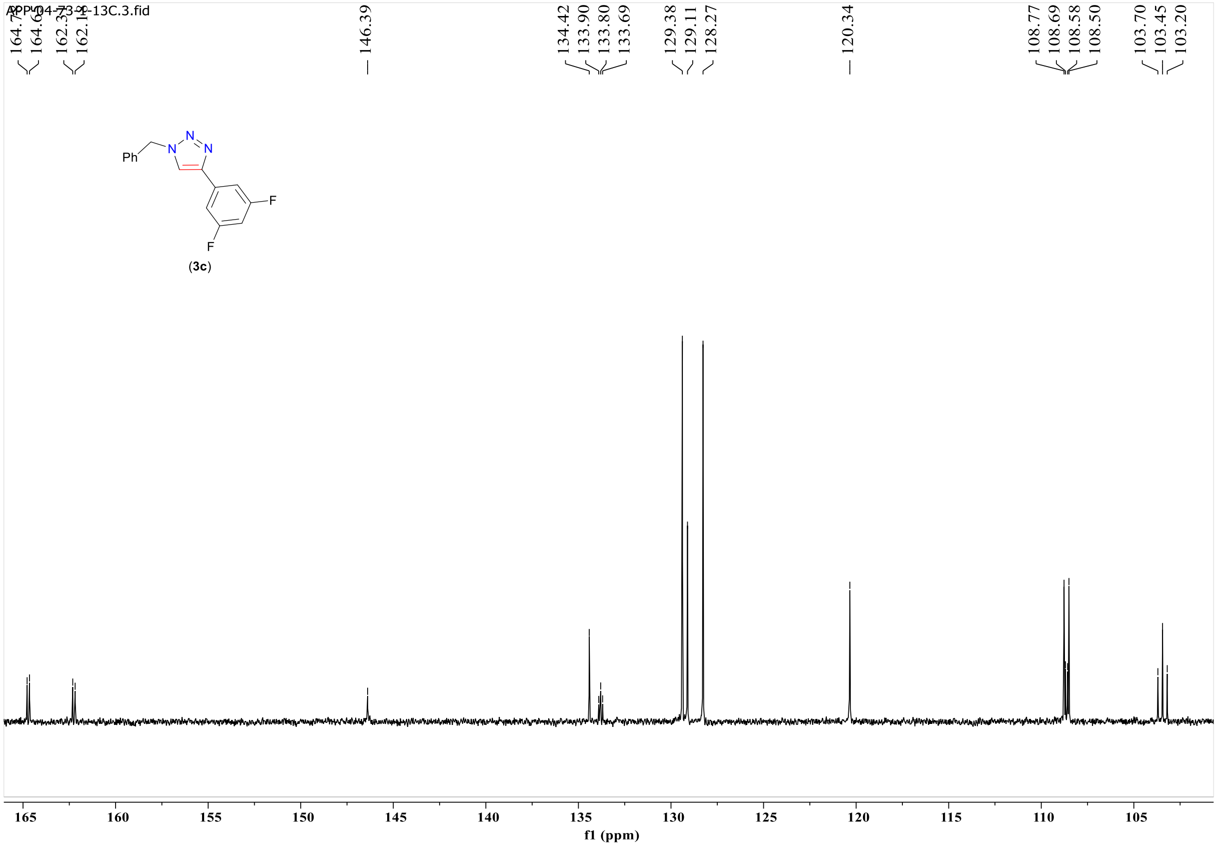

Figure S24. Expanded $^{13}C\{^1H\}$ NMR of 1-benzyl-4-(3,5-difluorophenyl)-1H-1,2,3-triazole (**3c**) in $CDCl_3$.

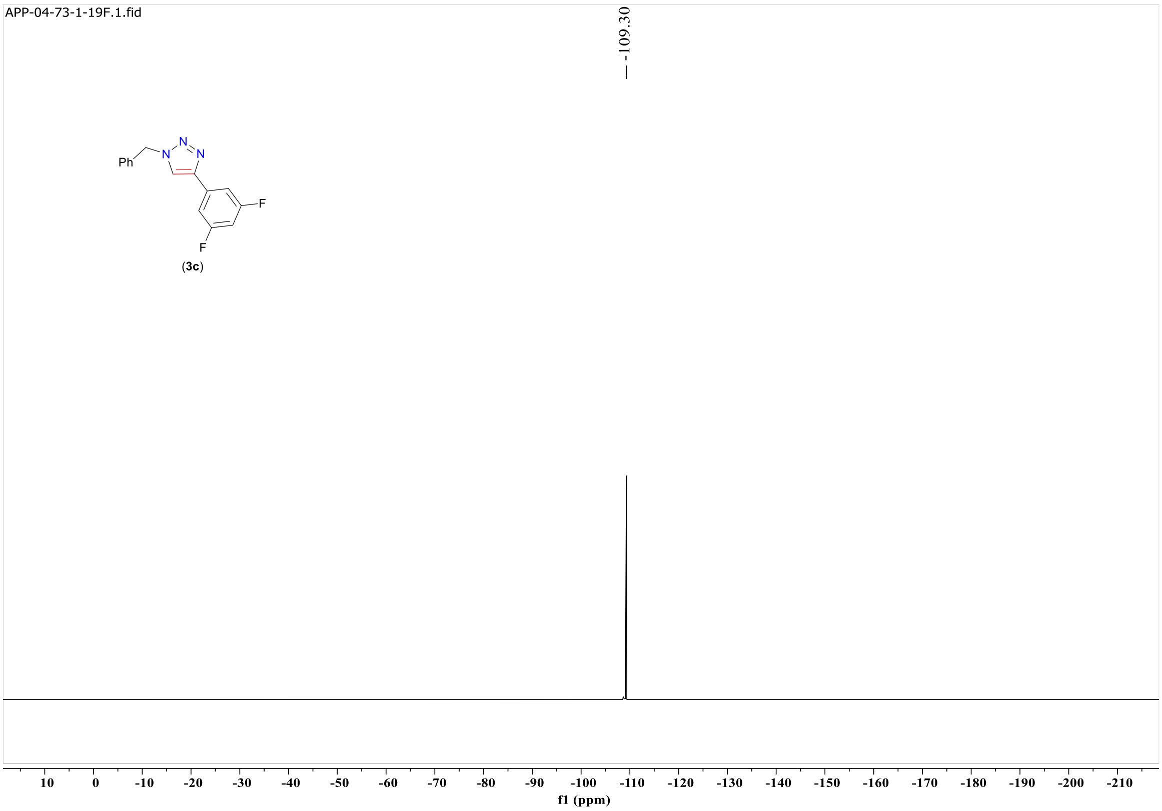

Figure S25. $^{19}F\{^{1}H\}$ NMR of 1-benzyl-4-(3,5-difluorophenyl)-1H-1,2,3-triazole (**3c**) in $CDCl_3$.

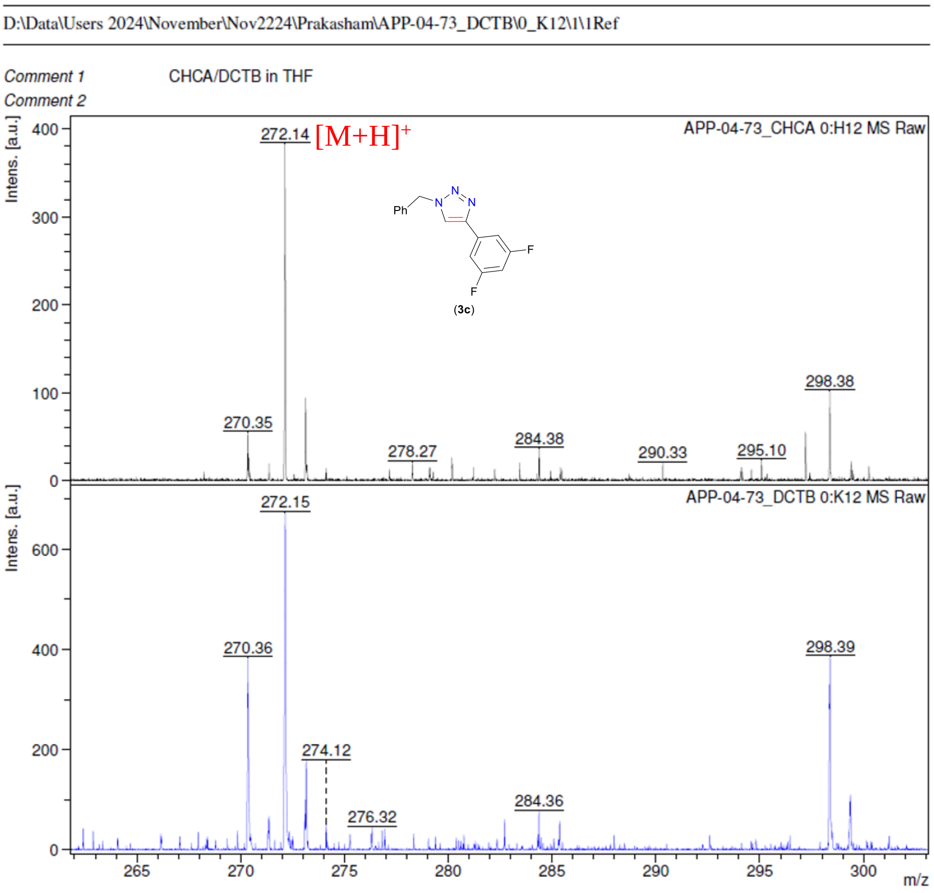

Figure S26. MALDI-ToF-MS spectra of 1-benzyl-4-(3,5-difluorophenyl)-1H-1,2,3-triazole (**3c**).

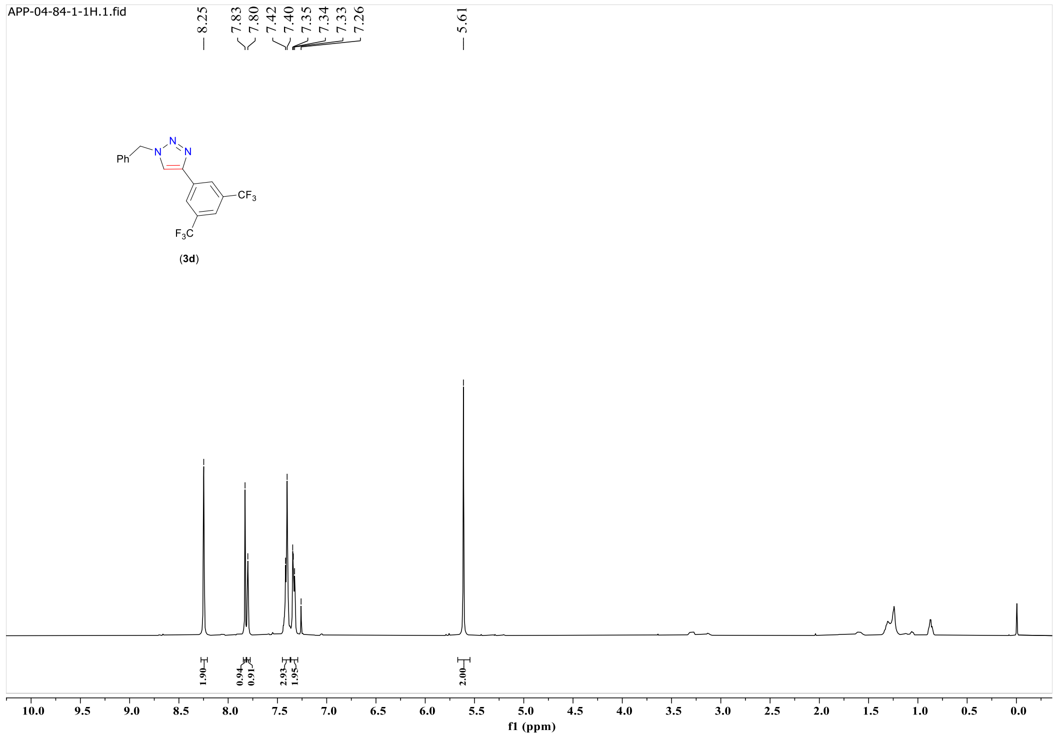

Figure S27. ^{1}H NMR of 1-benzyl-4-(3,5-bis(trifluoromethyl)phenyl)-1H-1,2,3-triazole (**3d**) in $CDCl_3$.

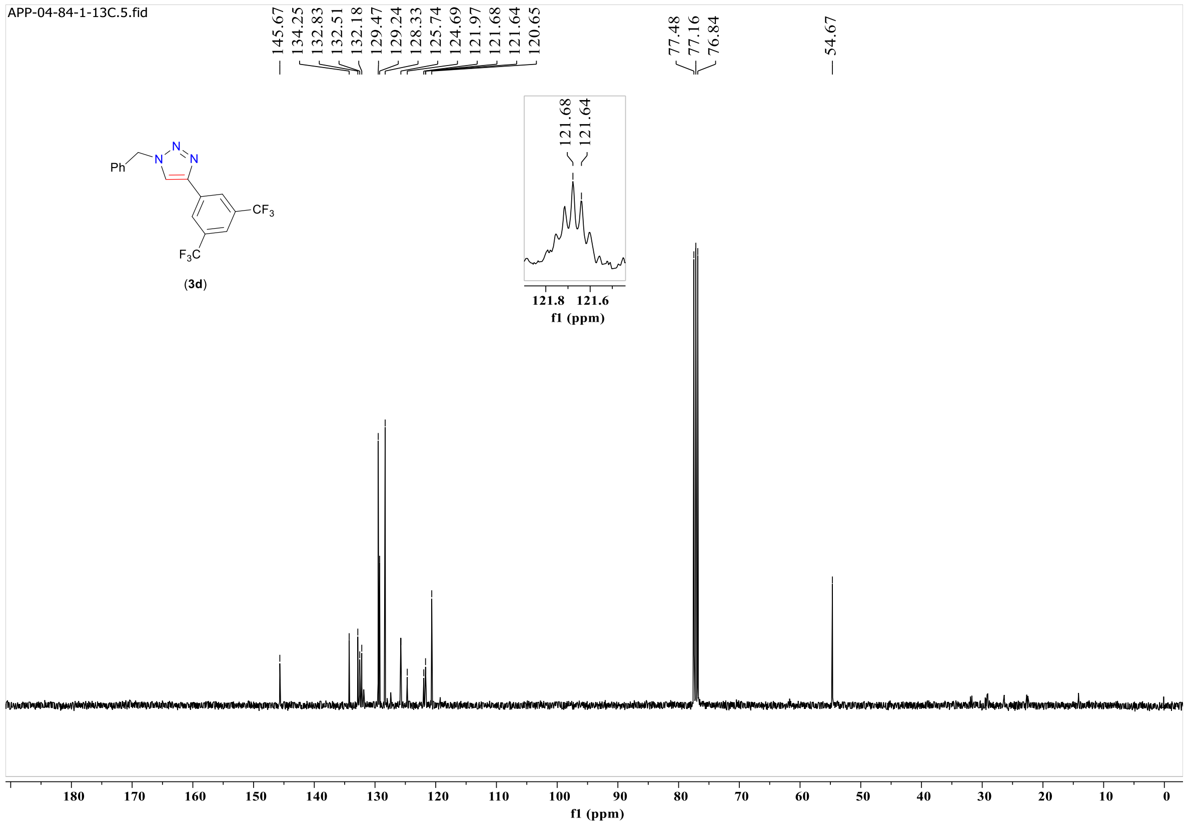

Figure S28. $^{13}C\{^{1}H\}$ NMR of 1-benzyl-4-(3,5-bis(trifluoromethyl)phenyl)-1H-1,2,3-triazole (**3d**) in $CDCl_3$.

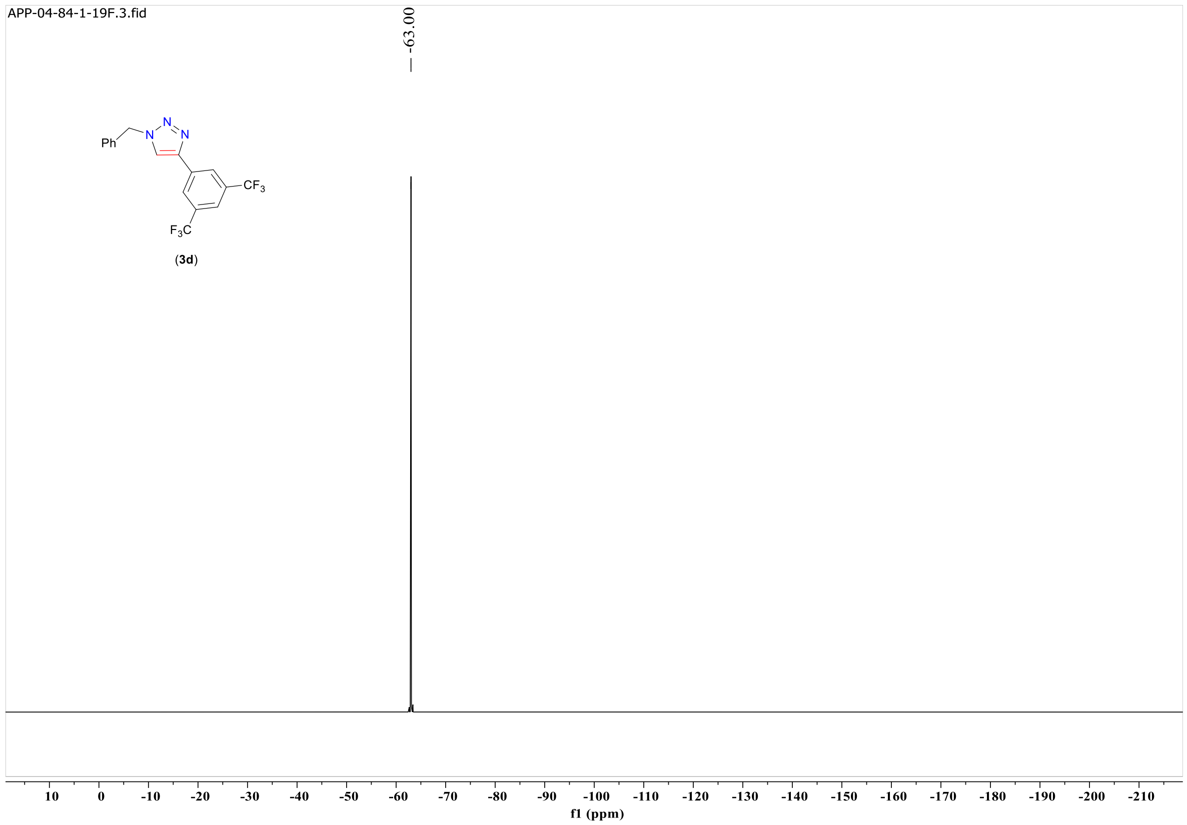

Figure S29. $^{19}F\{^{1}H\}$ NMR of 1-benzyl-4-(3,5-bis(trifluoromethyl)phenyl)-1H-1,2,3-triazole (**3d**) in $CDCl_3$.

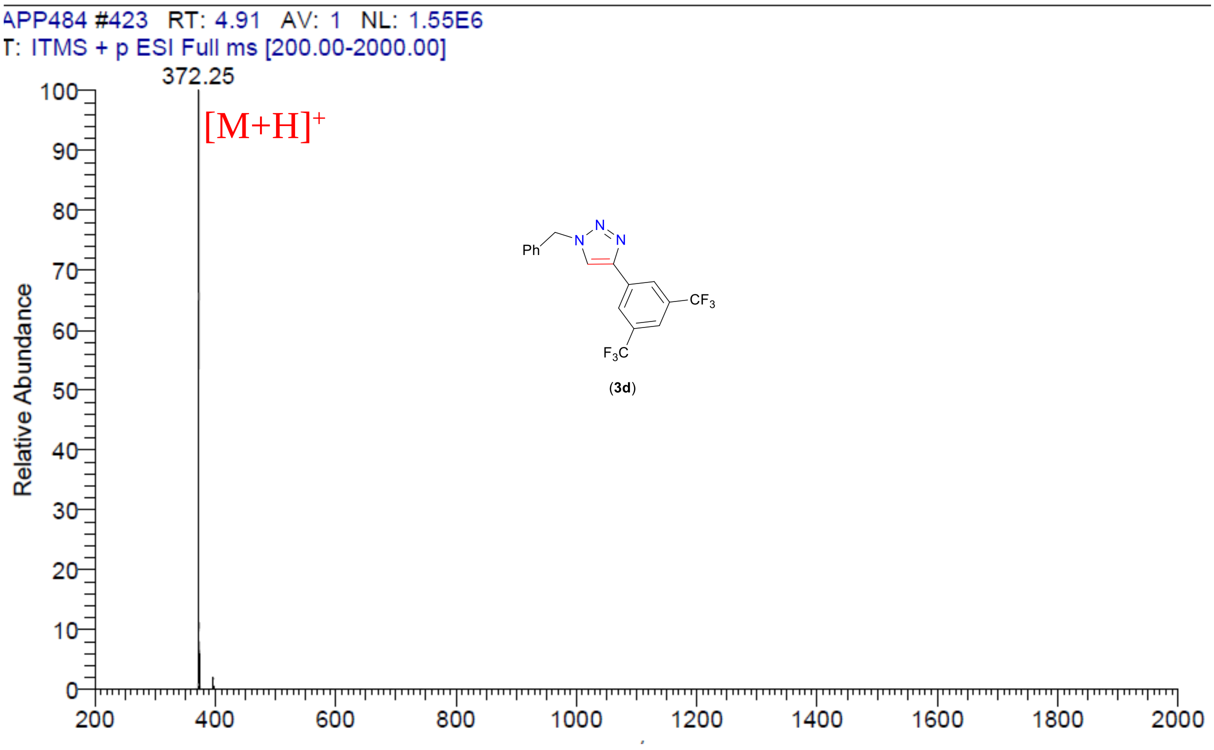

Figure S30. LCMS (ESI) trace of 1-benzyl-4-(3,5-bis(trifluoromethyl)phenyl)-1H-1,2,3-triazole (**3d**).

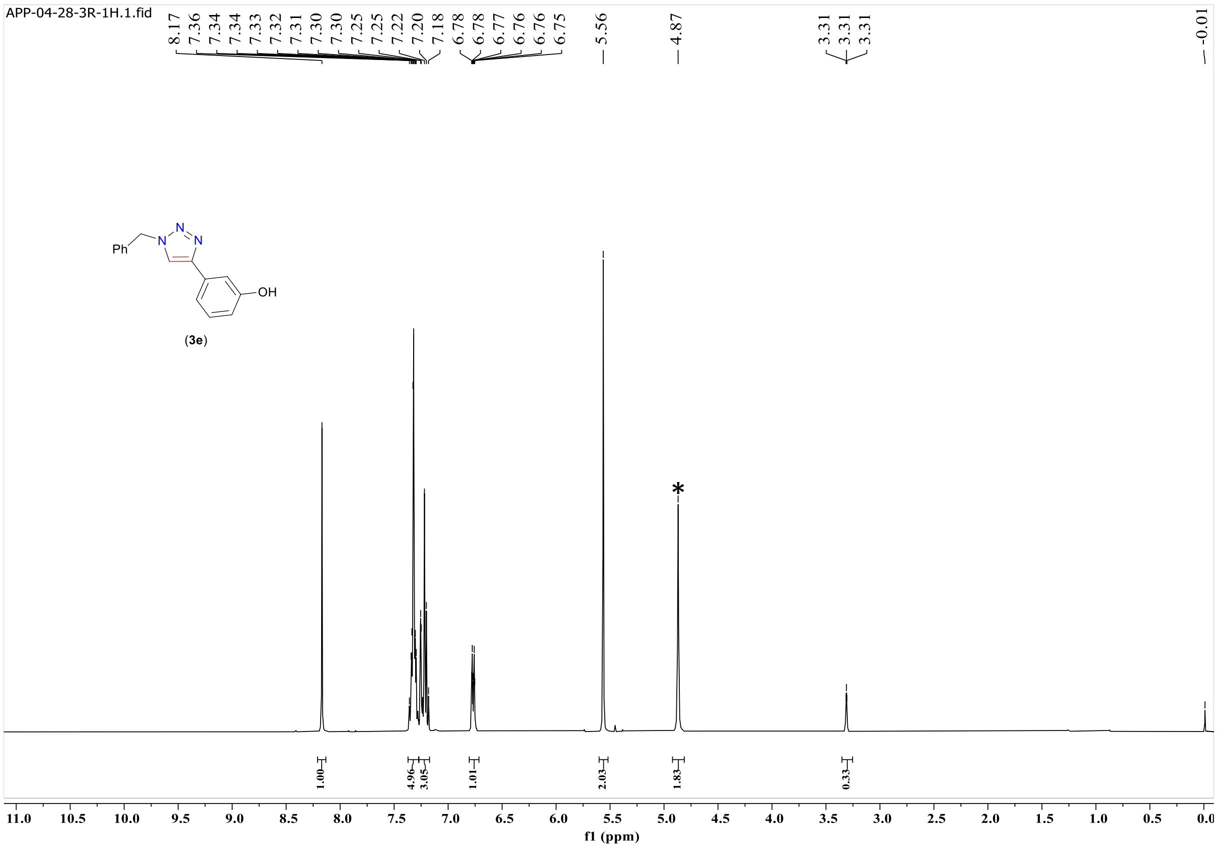

Figure S31. ^{1}H NMR of 3-(1-benzyl-1H-1,2,3-triazol-4-yl)phenol (**3e**) in CD_3OD. * H_2O.

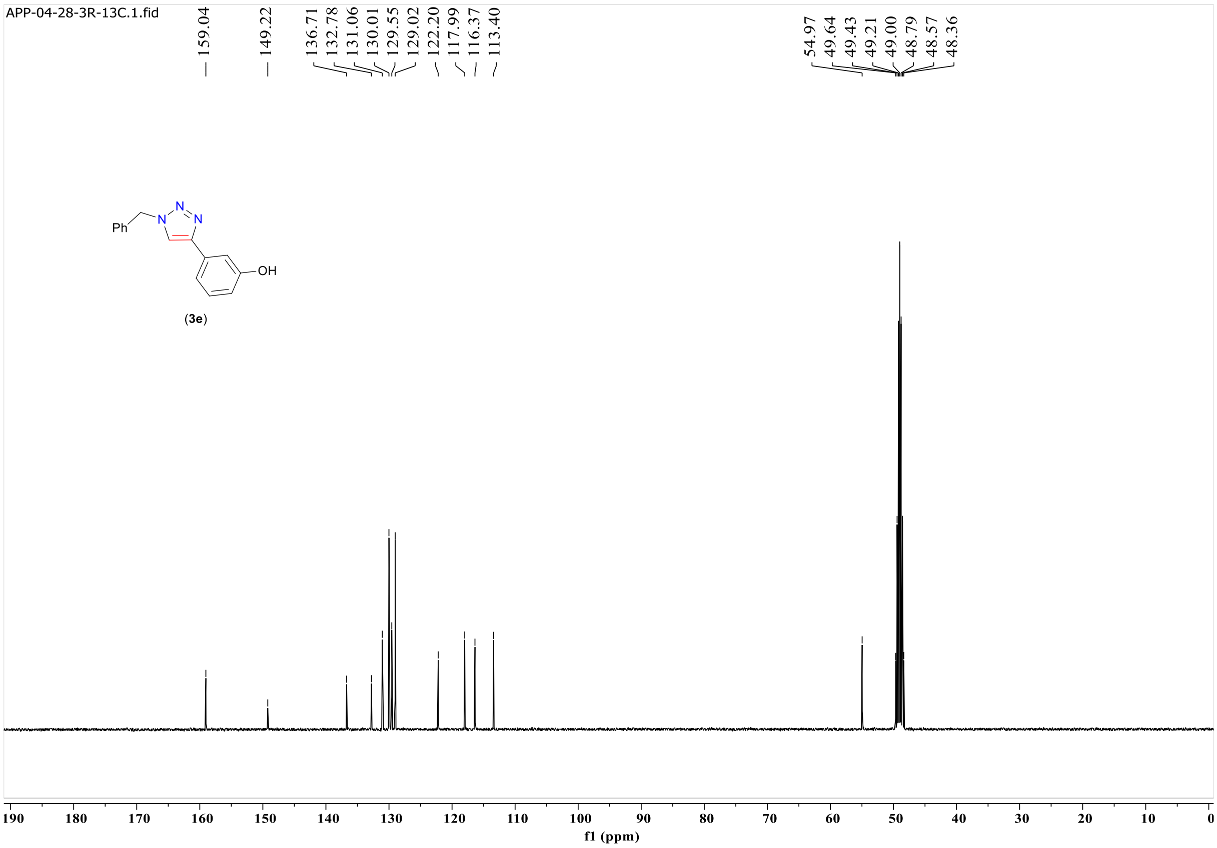

Figure S32. ^{13}C{^{1}H} NMR of 3-(1-benzyl-1H-1,2,3-triazol-4-yl)phenol (**3e**) in CD_3OD.

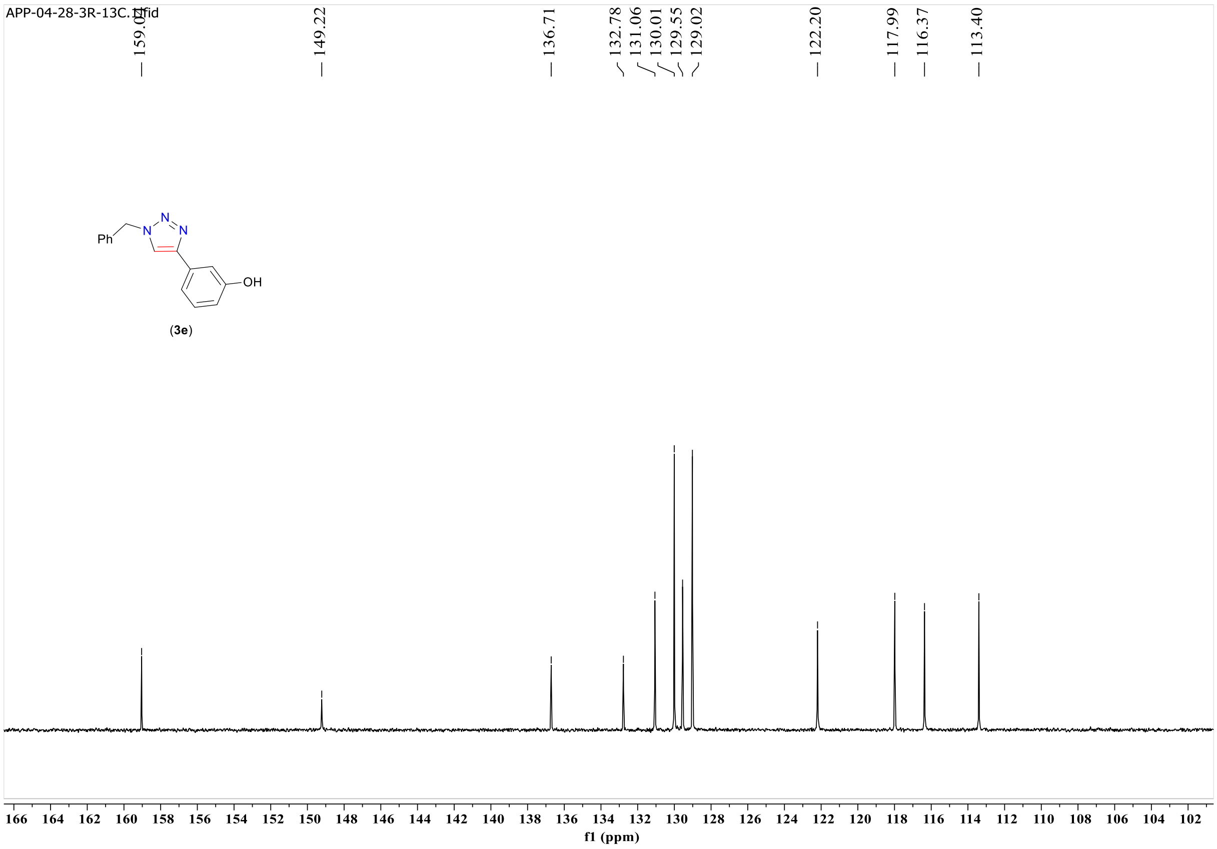

Figure S33. Expanded $^{13}C\{^{1}H\}$ NMR of 3-(1-benzyl-1H-1,2,3-triazol-4-yl)phenol (**3e**) in CD_3OD.

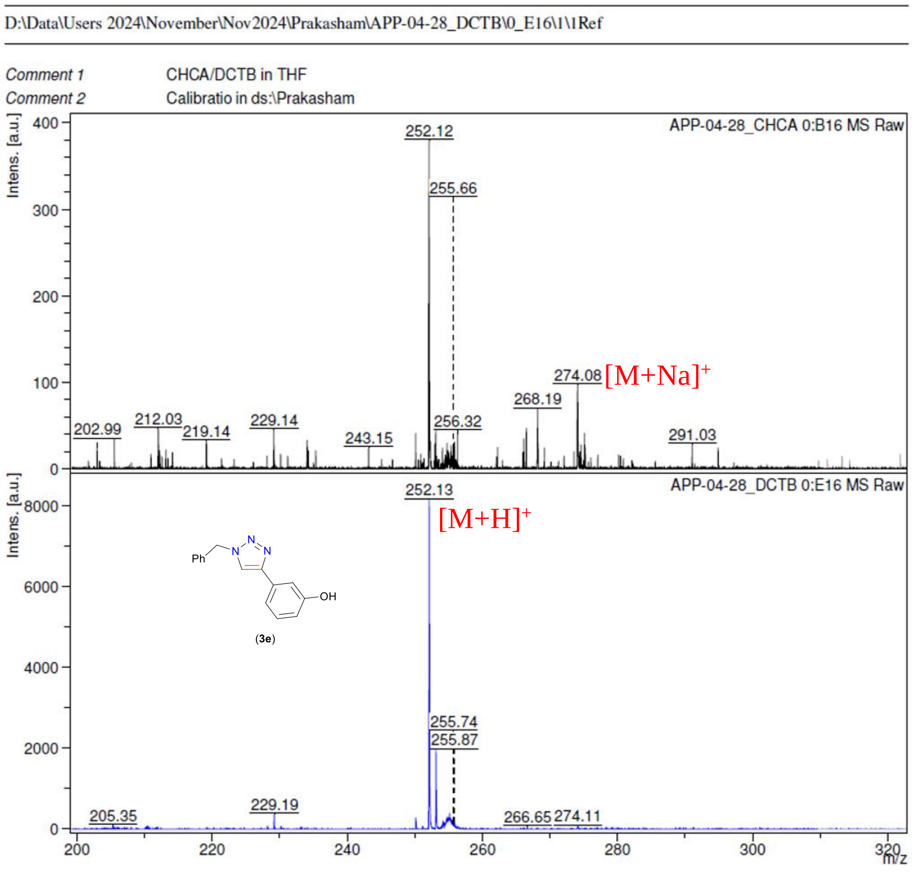

Figure S34. MALDI-ToF-MS spectra of 3-(1-benzyl-1H-1,2,3-triazol-4-yl)phenol (**3e**).

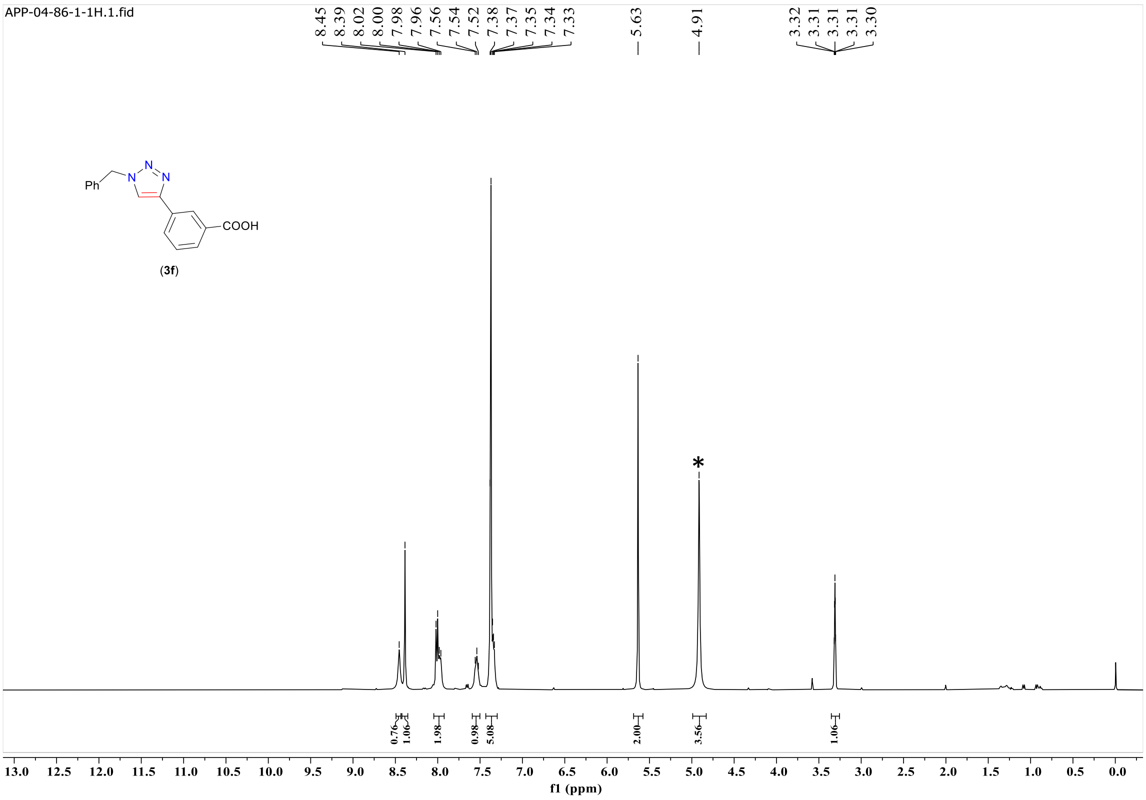

Figure S35. ^{1}H NMR of 3-(1-benzyl-1H-1,2,3-triazol-4-yl)benzoic acid (**3f**) in CD_3OD. * H_2O.

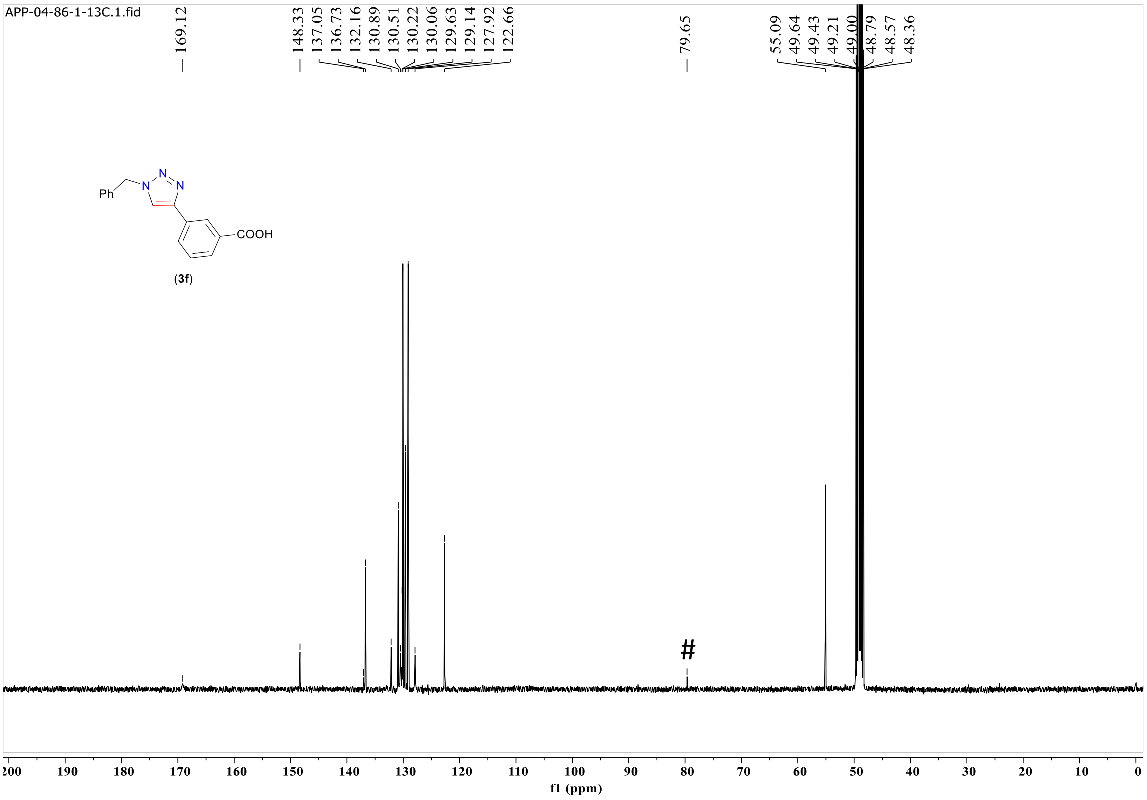

Figure S36. ^{13}C{^{1}H} NMR of 3-(1-benzyl-1H-1,2,3-triazol-4-yl)benzoic acid (**3f**) in CD_3OD. # $CHCl_3$.

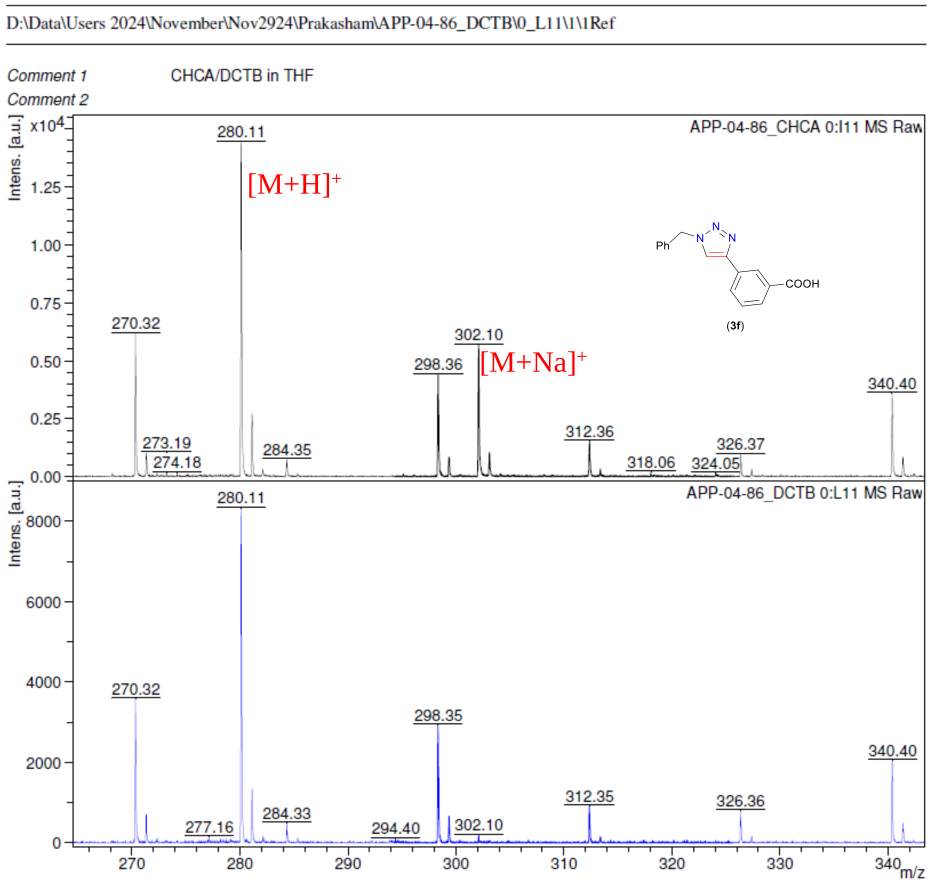

Figure S37. MALDI-ToF-MS spectra of 3-(1-benzyl-1H-1,2,3-triazol-4-yl)benzoic acid (**3f**).

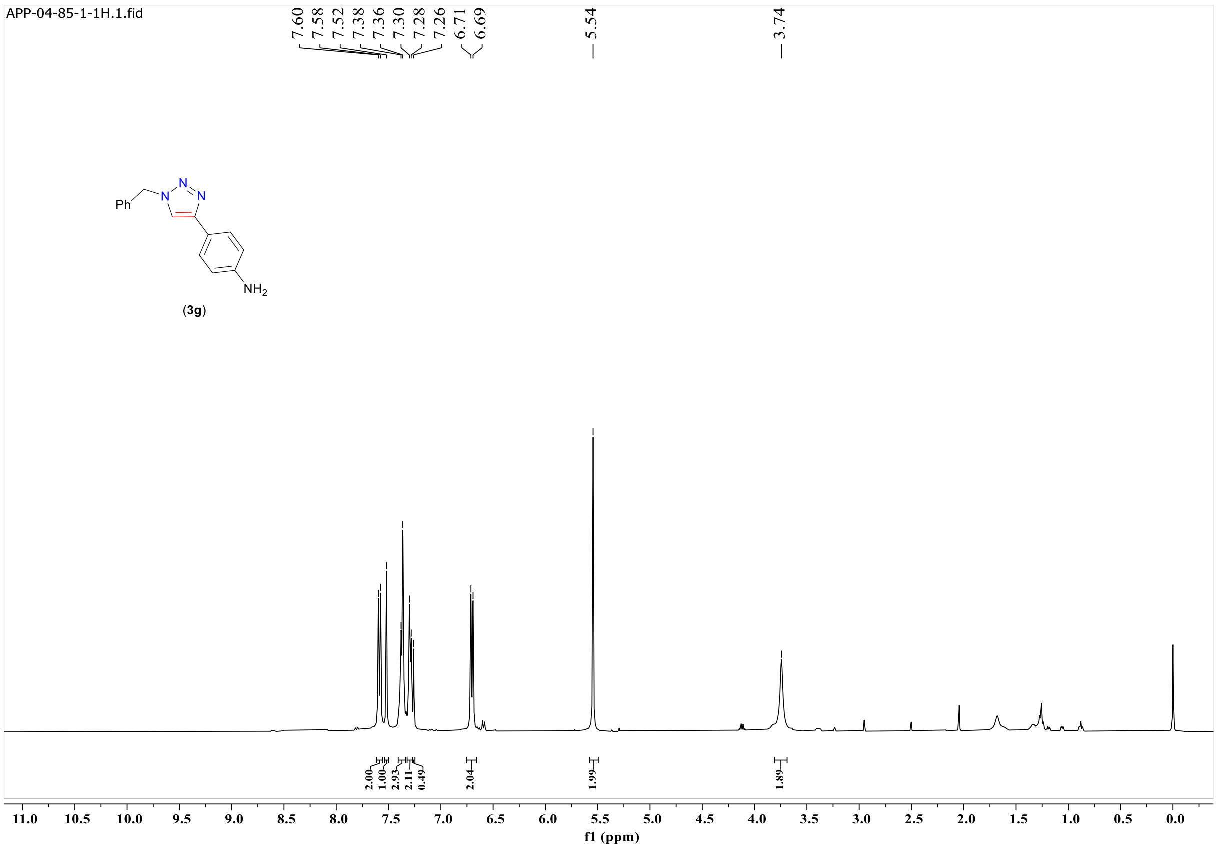

Figure S38. ^{1}H NMR of 4-(1-benzyl-1H-1,2,3-triazol-4-yl)aniline (**3g**) in $CDCl_3$.

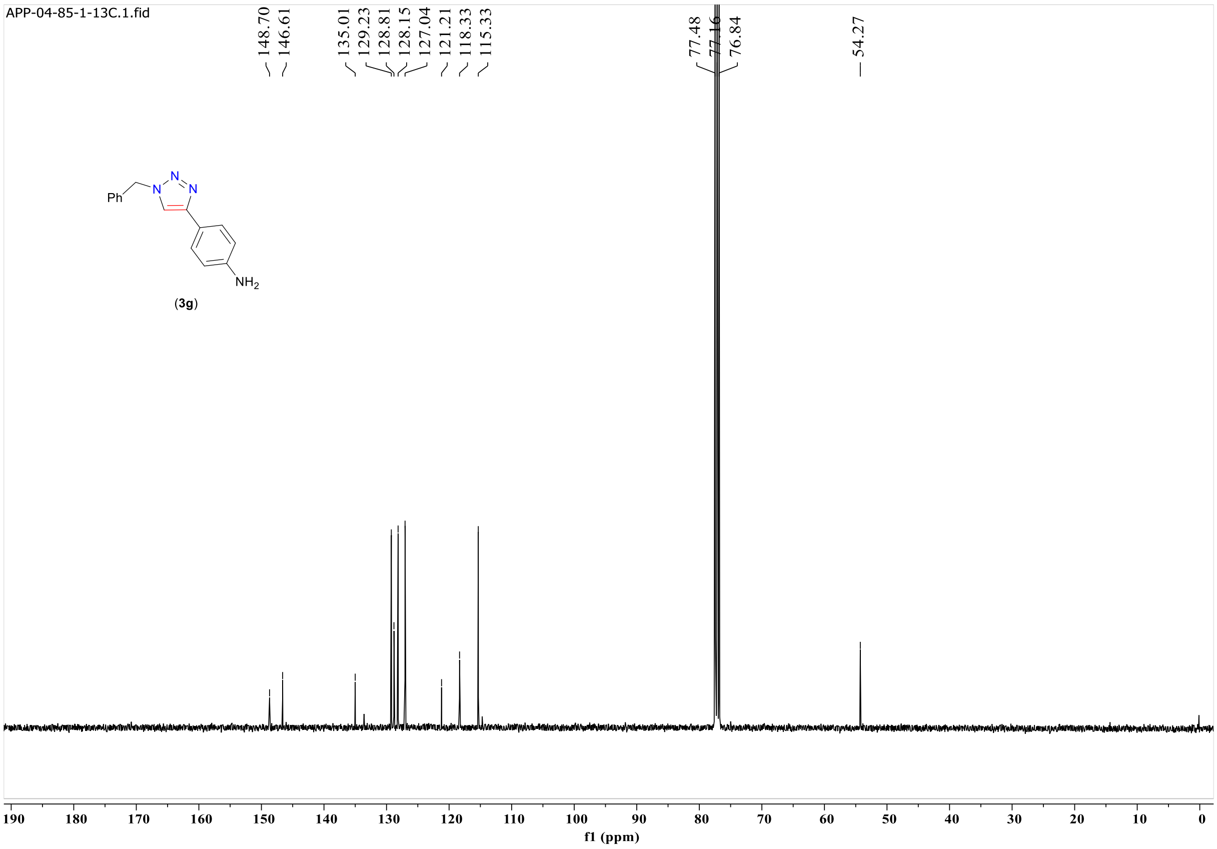

Figure S39. $^{13}C\{^{1}H\}$ NMR of 4-(1-benzyl-1H-1,2,3-triazol-4-yl)aniline (**3g**) in $CDCl_3$.

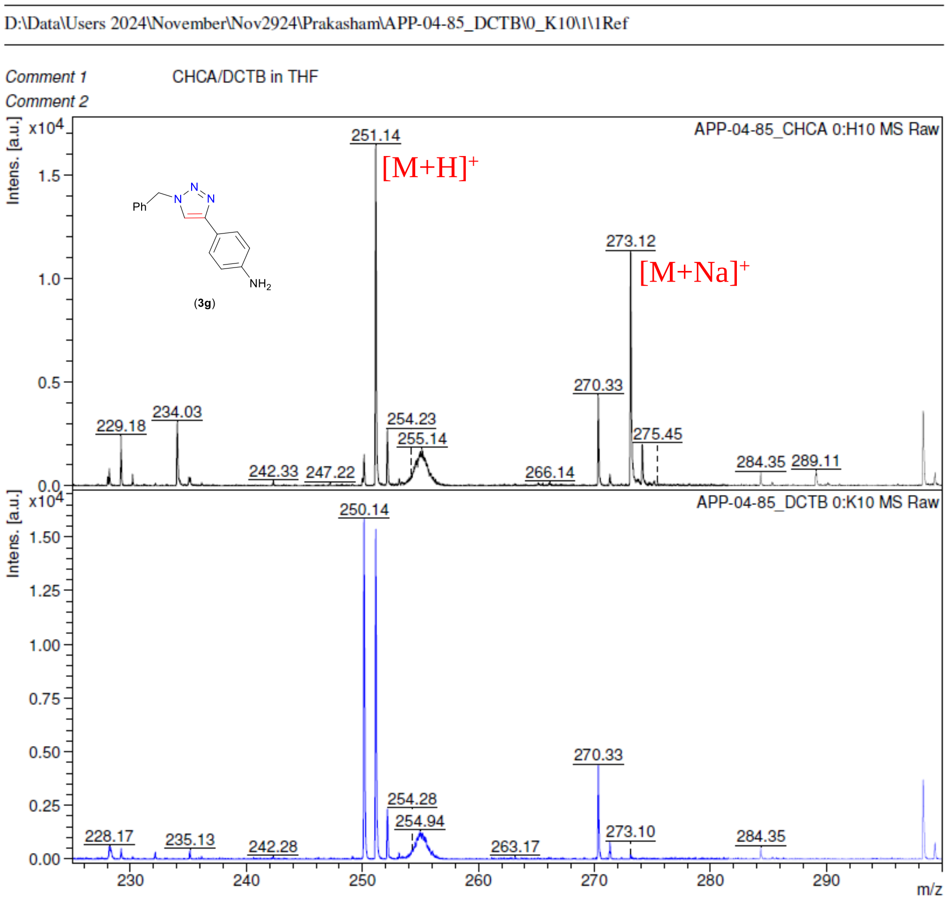

Figure S40. MALDI-ToF-MS spectra of 4-(1-benzyl-1H-1,2,3-triazol-4-yl)aniline (**3g**).

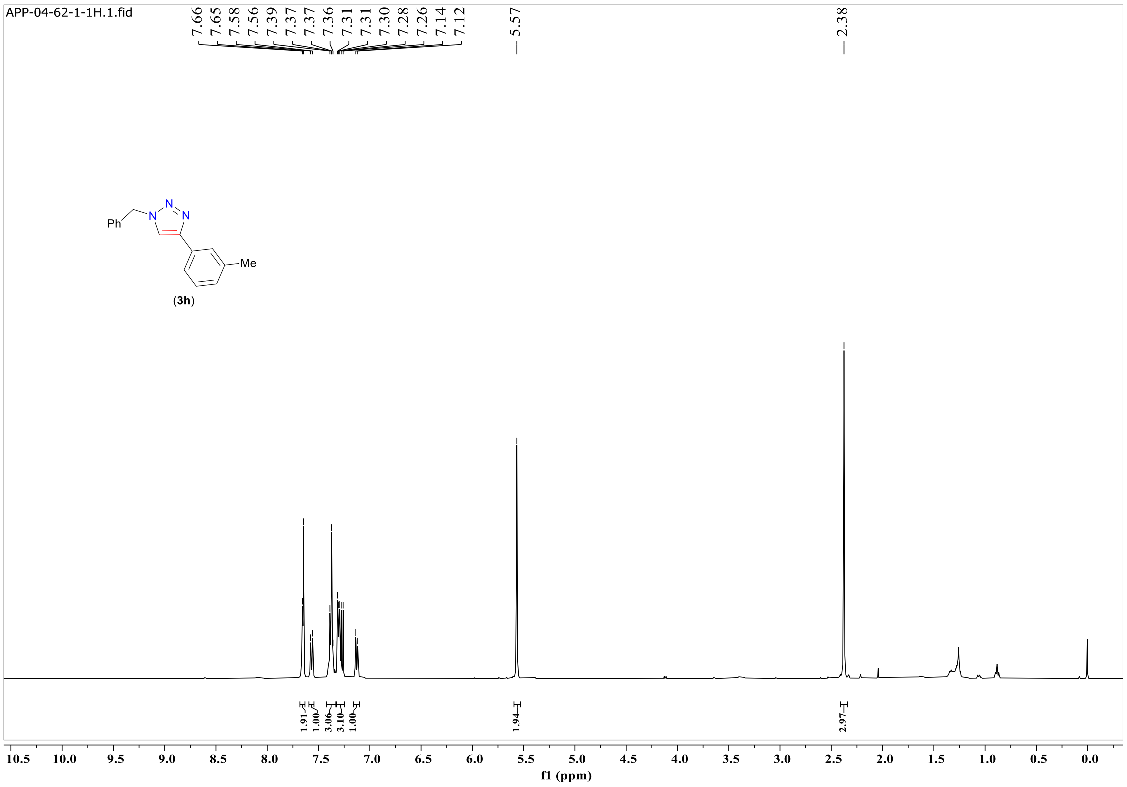

Figure S41. ^{1}H NMR of 1-benzyl-4-(*m*-tolyl)-1H-1,2,3-triazole (**3h**) in $CDCl_3$.

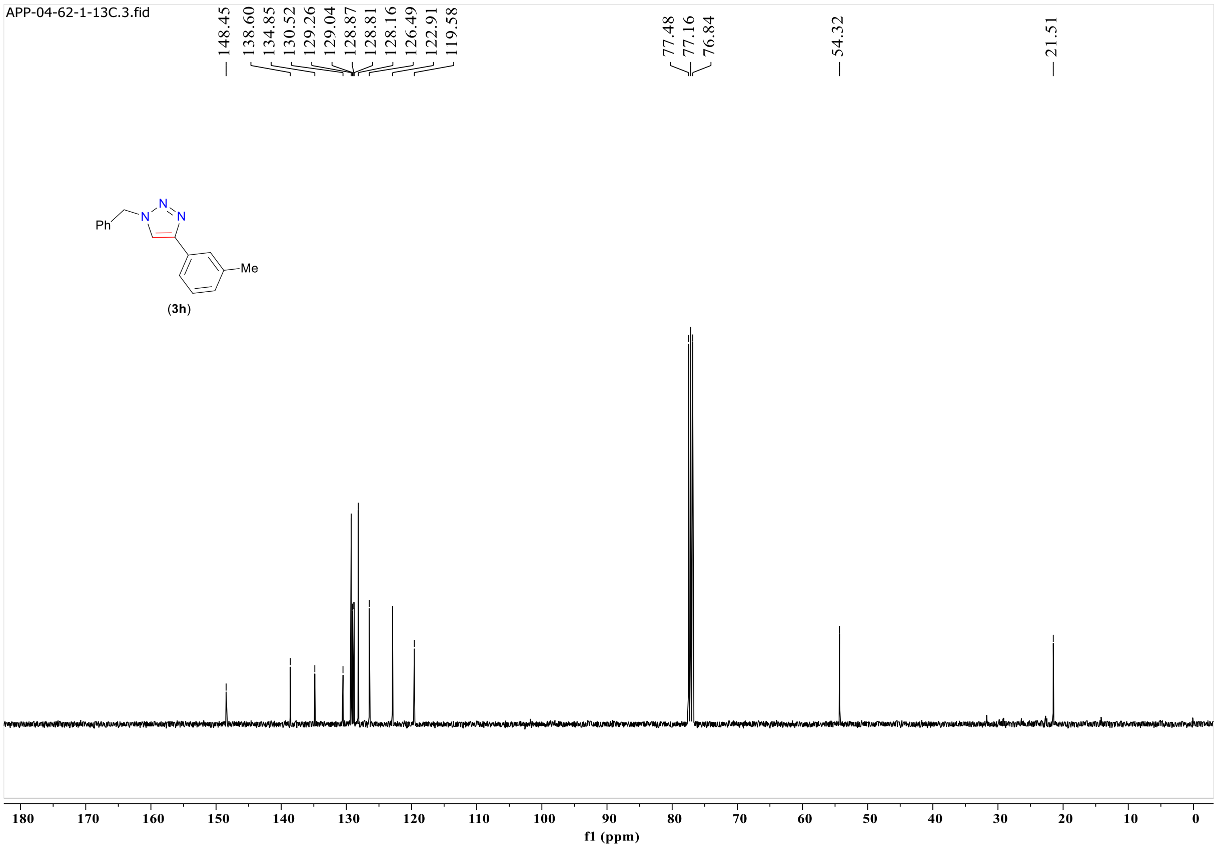

Figure S42. $^{13}C\{^{1}H\}$ NMR of 1-benzyl-4-(*m*-tolyl)-1H-1,2,3-triazole (**3h**) in $CDCl_3$.

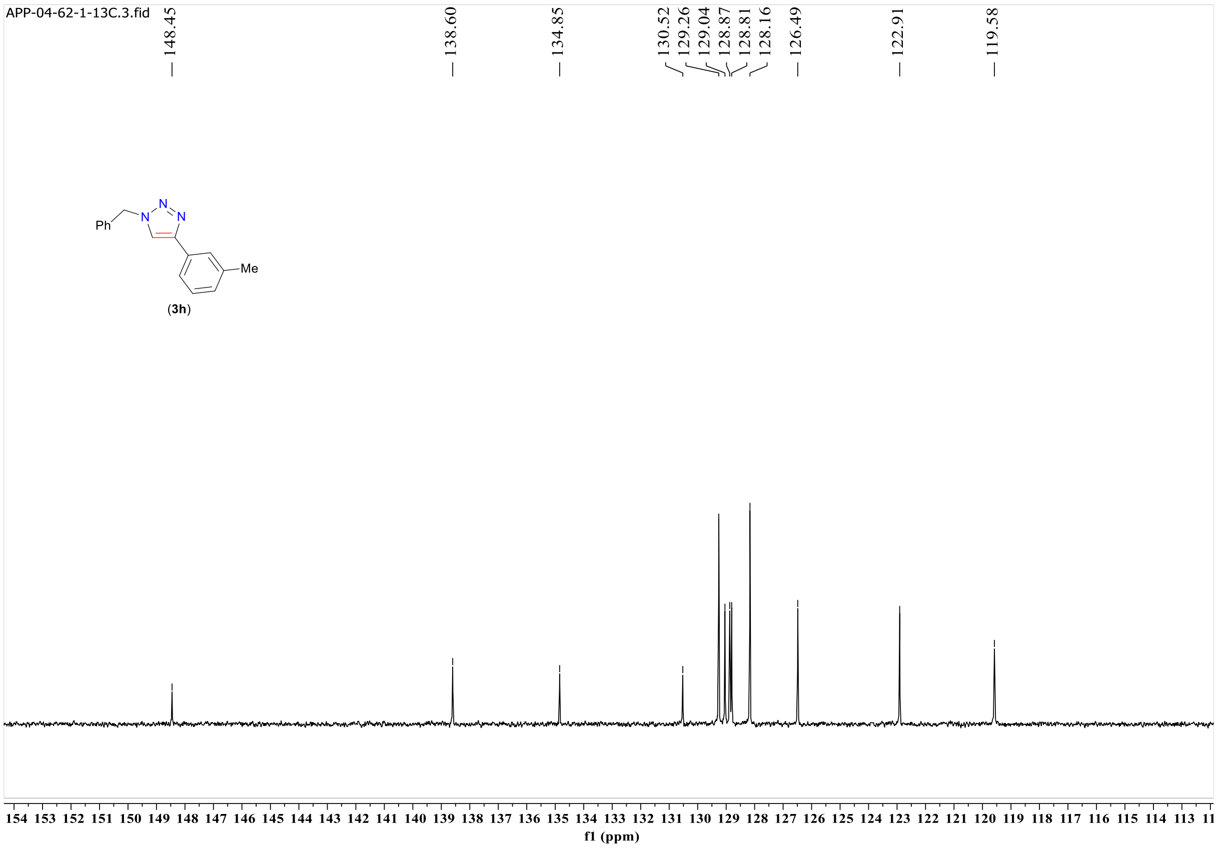

Figure S43. Expanded $^{13}C\{^1H\}$ NMR of 1-benzyl-4-(*m*-tolyl)-1H-1,2,3-triazole (**3h**) in $CDCl_3$.

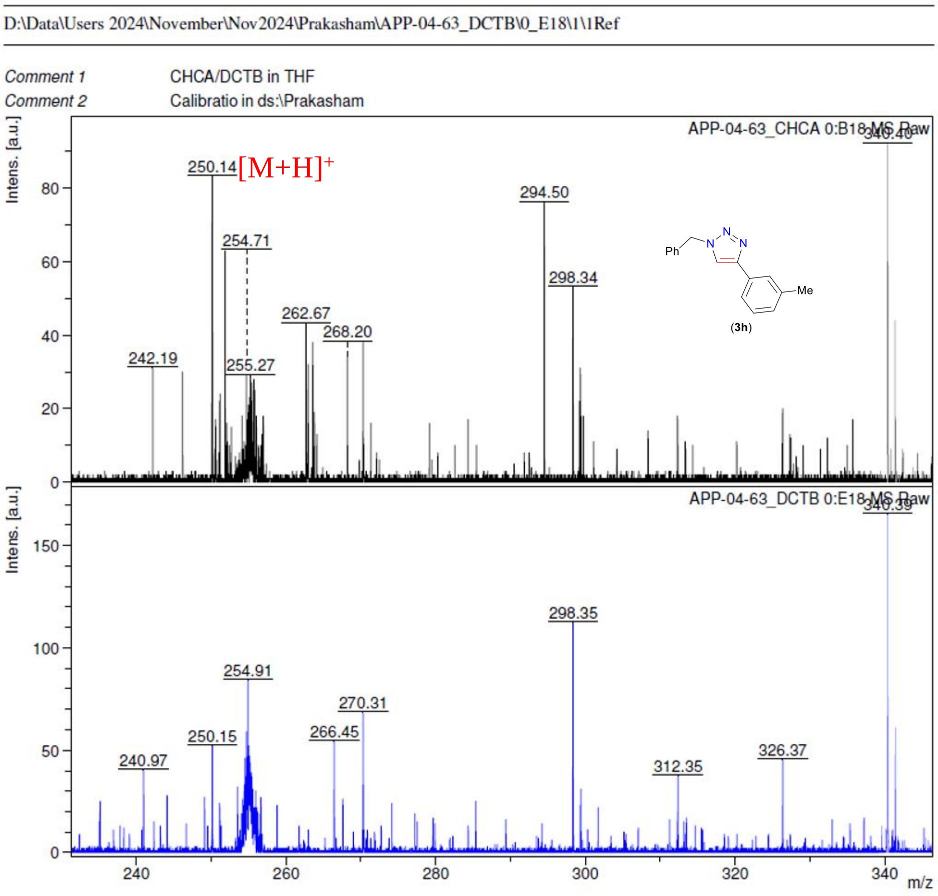

Figure S44. MALDI-ToF-MS spectra of 1-benzyl-4-(*m*-tolyl)-1H-1,2,3-triazole (**3h**).

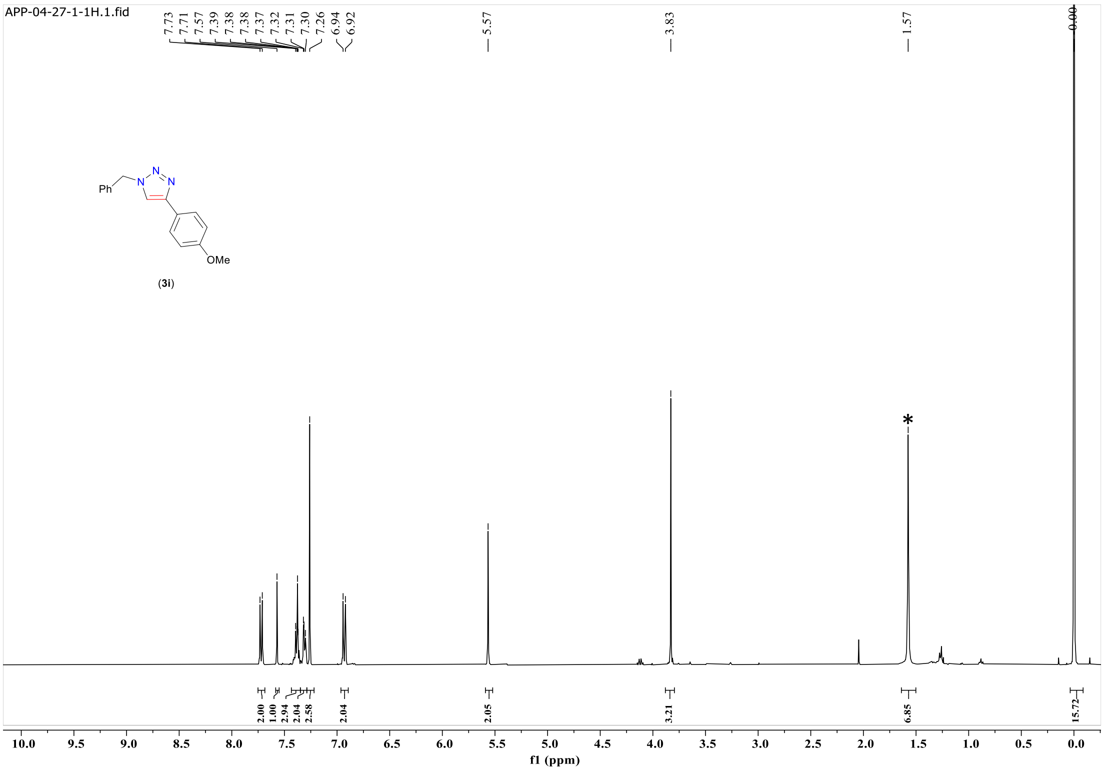

Figure S45. ^{1}H NMR of 1-benzyl-4-(4-methoxyphenyl)-1H-1,2,3-triazole (**3i**) in $CDCl_3$. * H_2O.

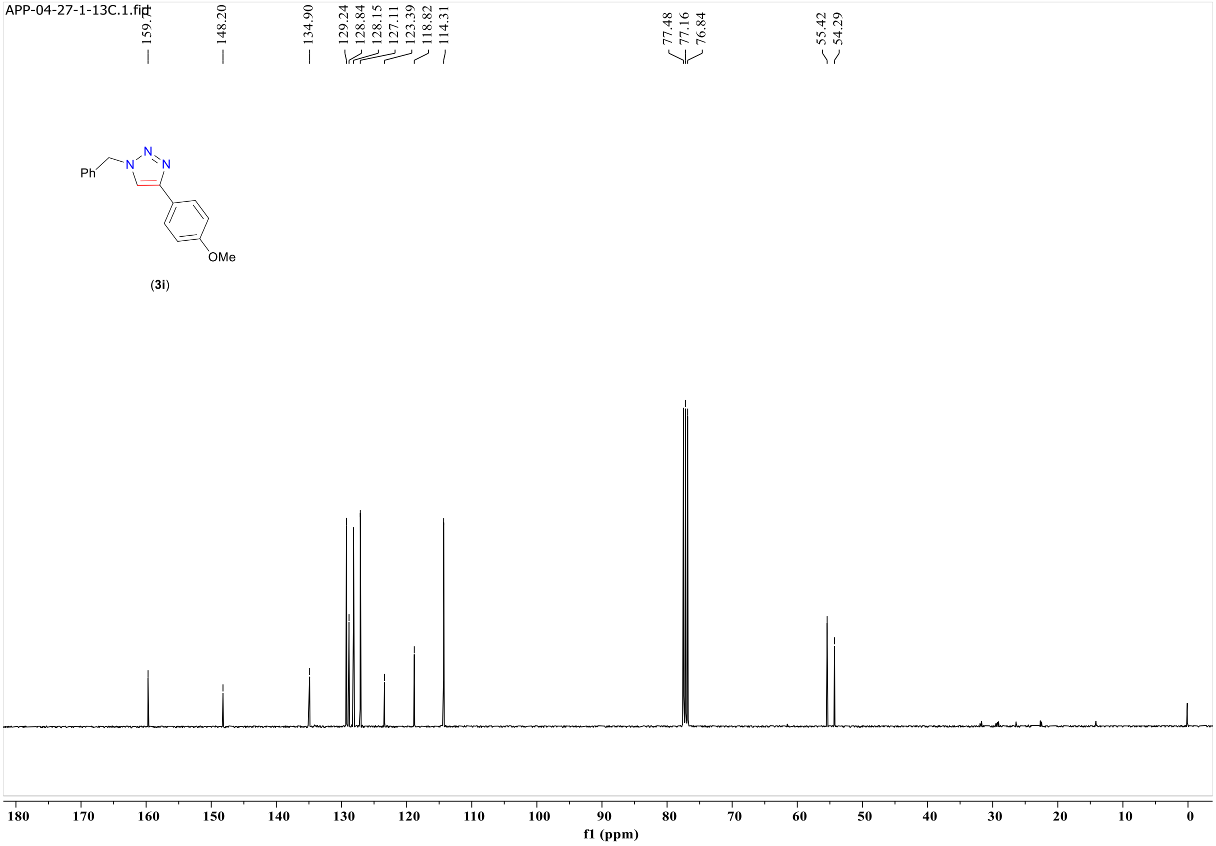

Figure S46. ^{13}C{^{1}H} NMR of 1-benzyl-4-(4-methoxyphenyl)-1H-1,2,3-triazole (**3i**) in $CDCl_3$.

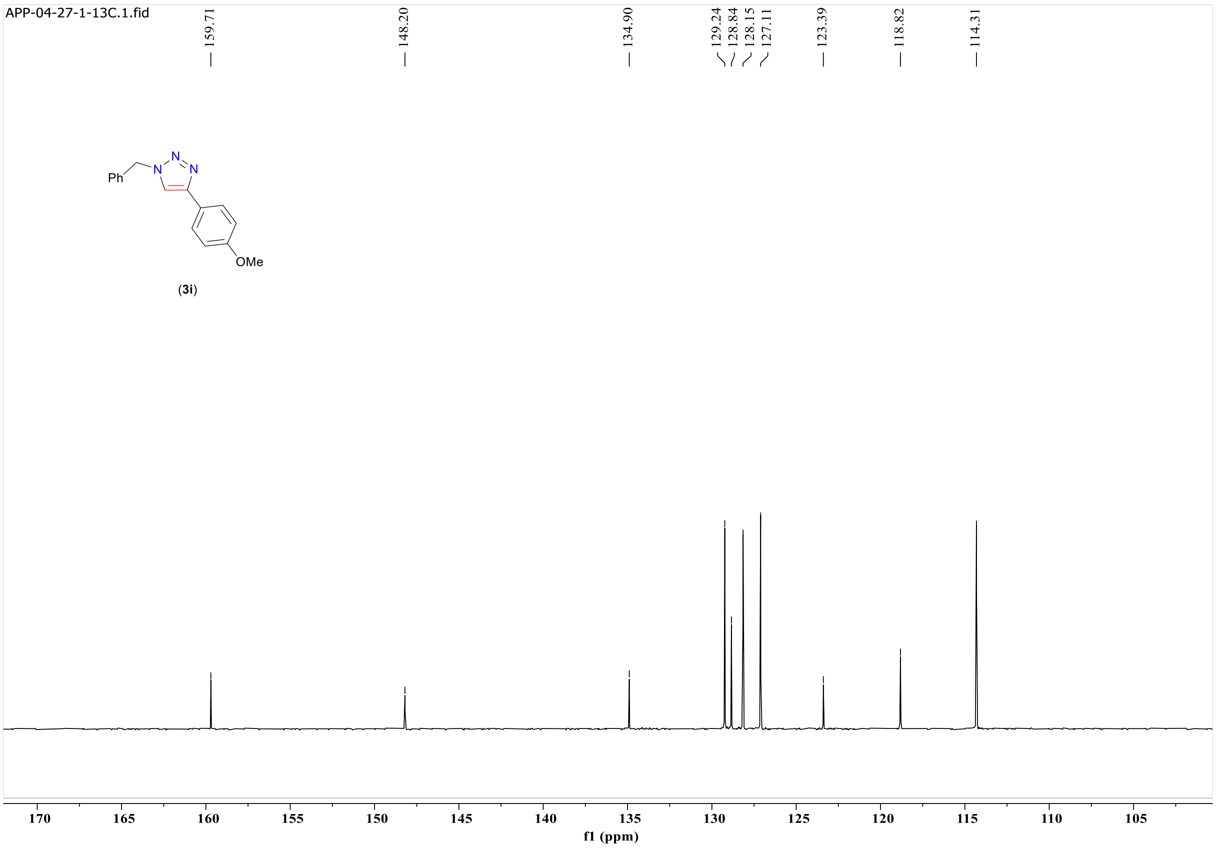

Figure S47. Expanded $^{13}C\{^{1}H\}$ NMR of 1-benzyl-4-(4-methoxyphenyl)-1H-1,2,3-triazole (**3i**) in $CDCl_3$.

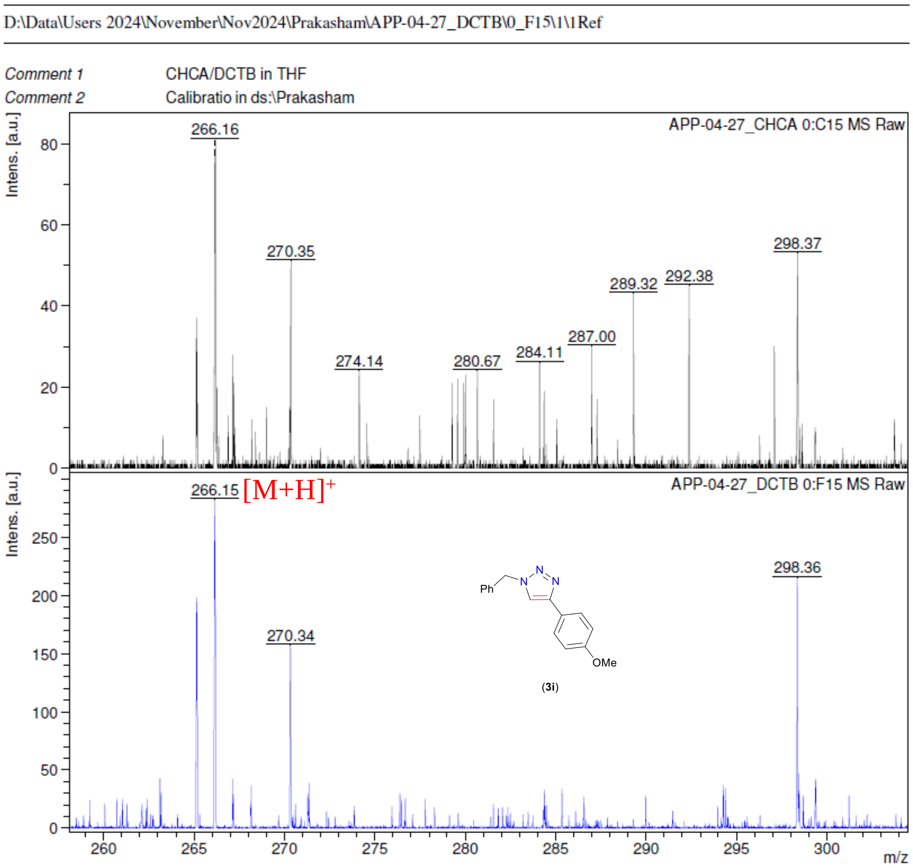

Figure S48. MALDI-ToF-MS spectra of 1-benzyl-4-(4-methoxyphenyl)-1H-1,2,3-triazole (**3i**).

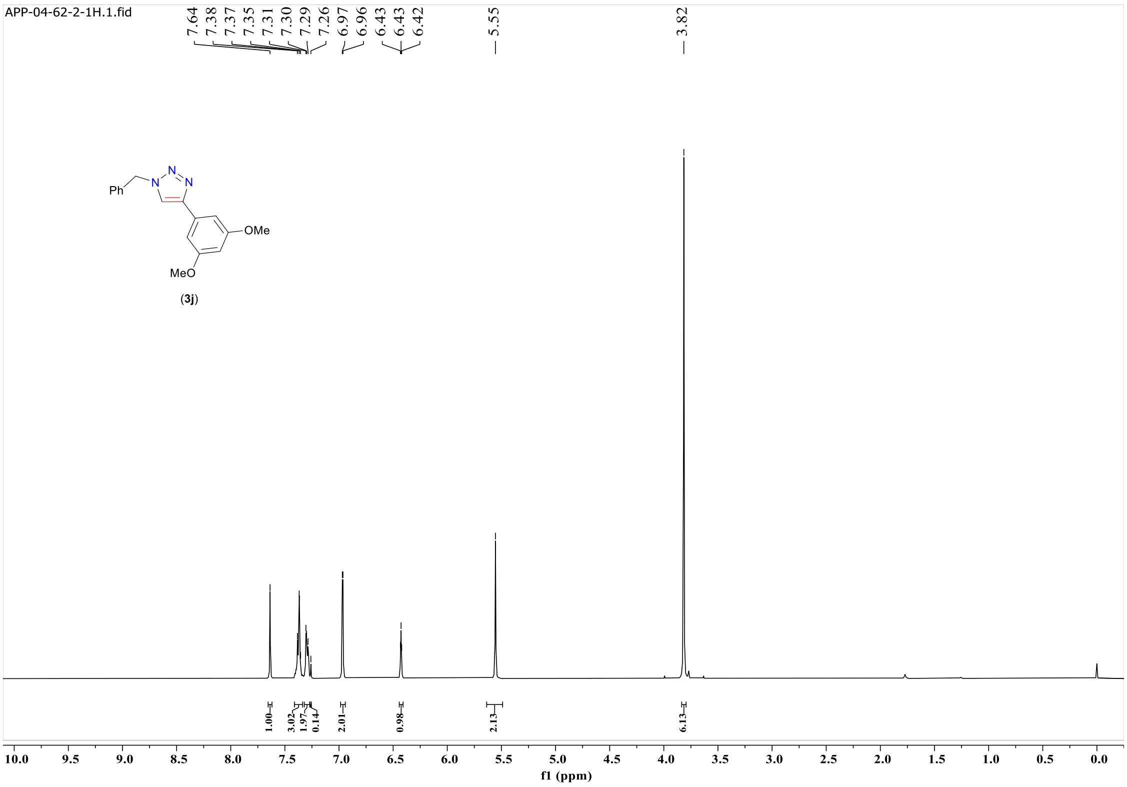

Figure S49. ^{1}H NMR of 1-benzyl-4-(3,5-dimethoxyphenyl)-1H-1,2,3-triazole (**3j**) in $CDCl_3$.

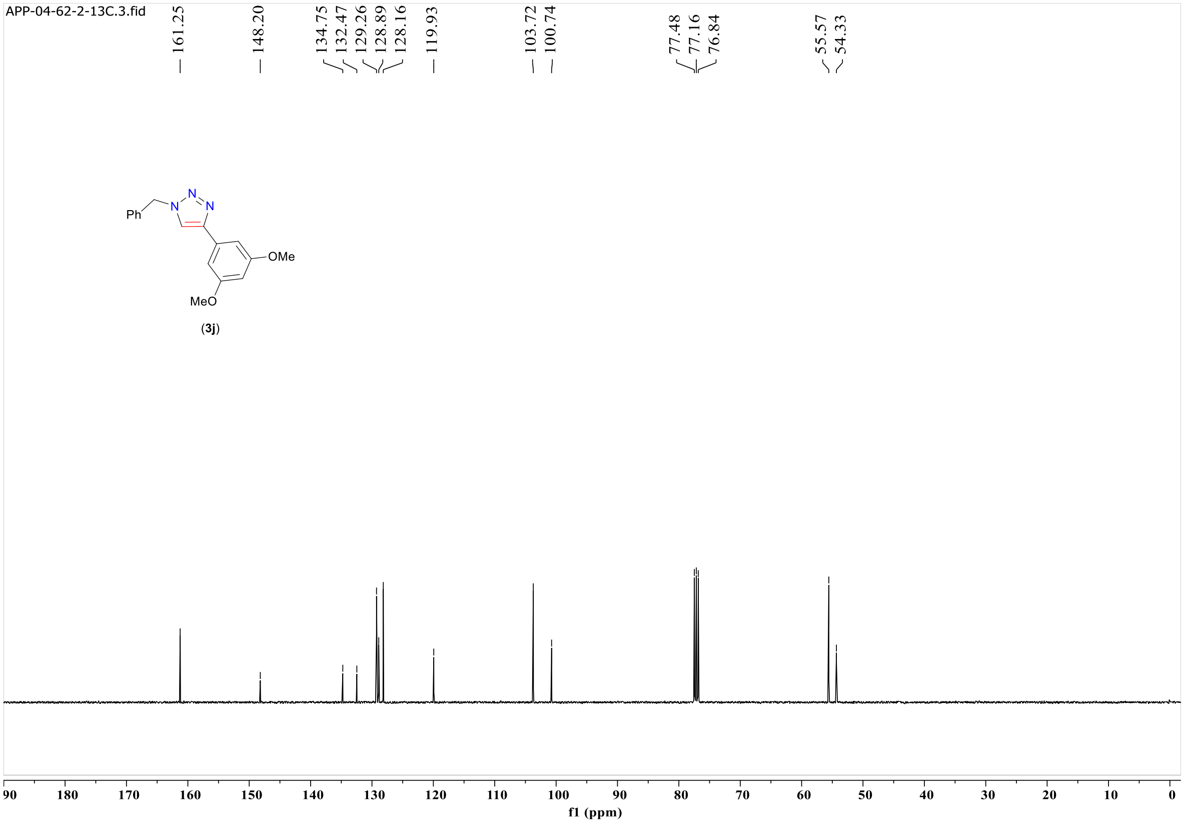

Figure S50. $^{13}C\{^1H\}$ NMR of 1-benzyl-4-(3,5-dimethoxyphenyl)-1H-1,2,3-triazole (**3j**) in $CDCl_3$.

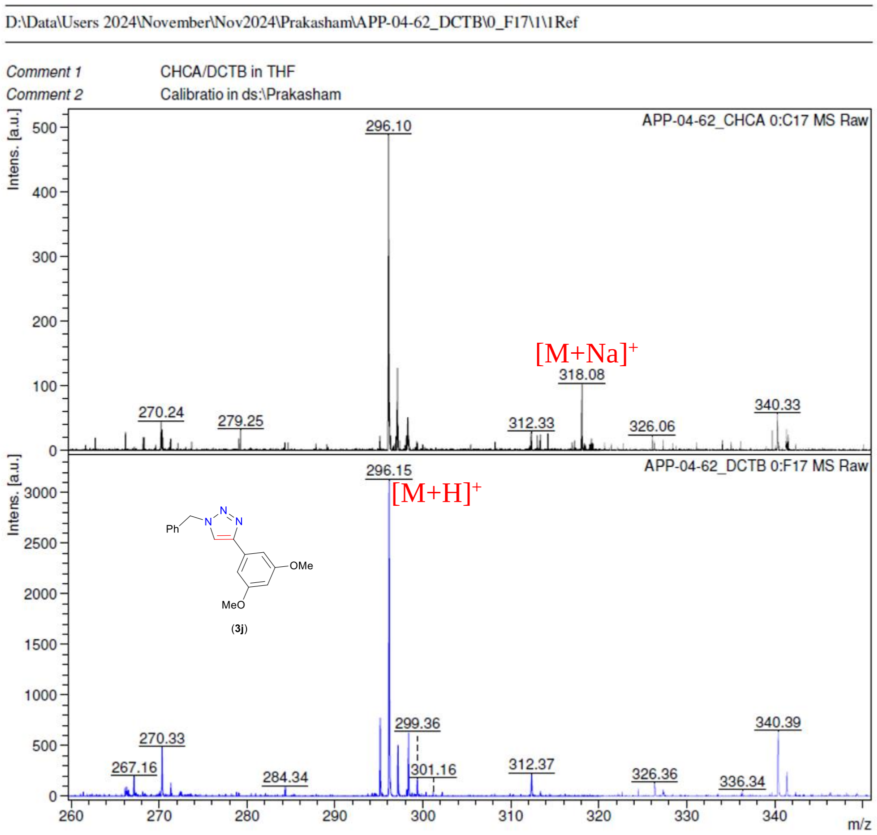

Figure S51. MALDI-ToF-MS spectra of 1-benzyl-4-(3,5-dimethoxyphenyl)-1H-1,2,3-triazole (**3j**).

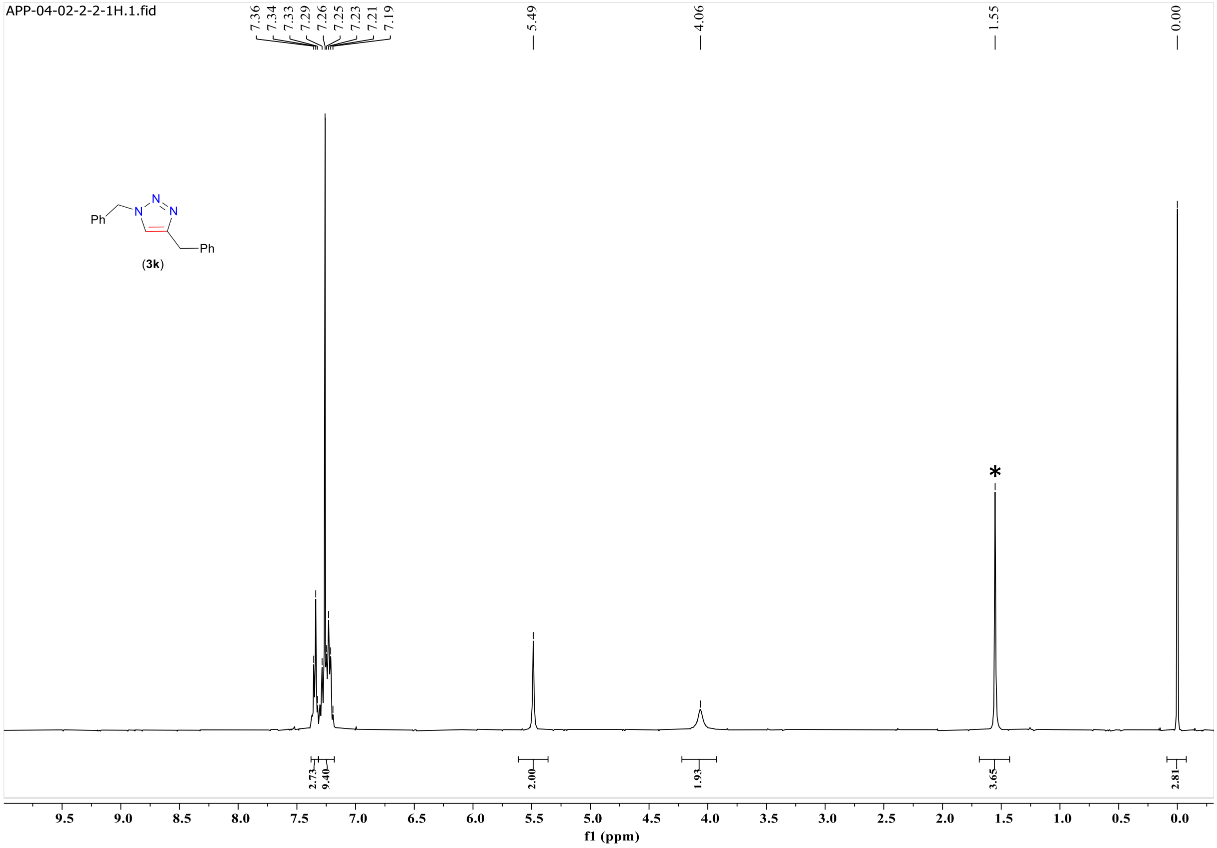

Figure S52. ^{1}H NMR of 1,4-dibenzyl-1H-1,2,3-triazole (**3k**) in $CDCl_3$. * H_2O.

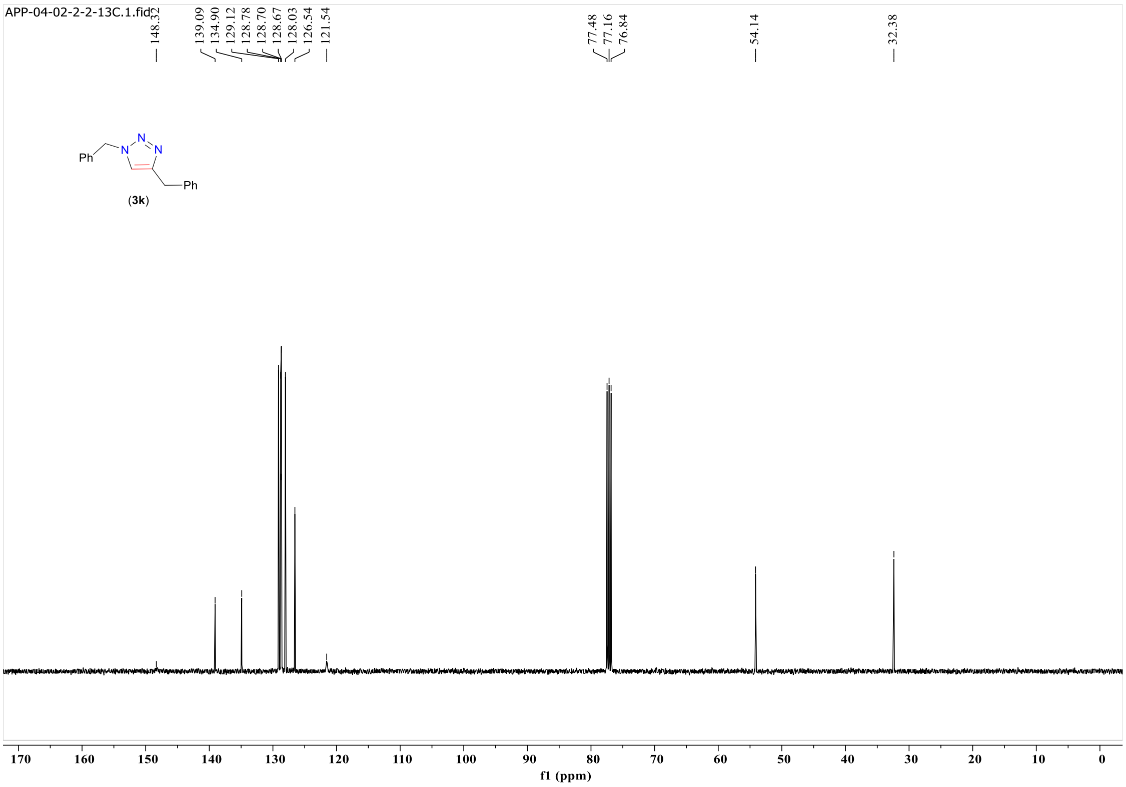

Figure S53. $^{13}C\{^{1}H\}$ NMR of 1,4-dibenzyl-1H-1,2,3-triazole (**3k**) in $CDCl_3$.

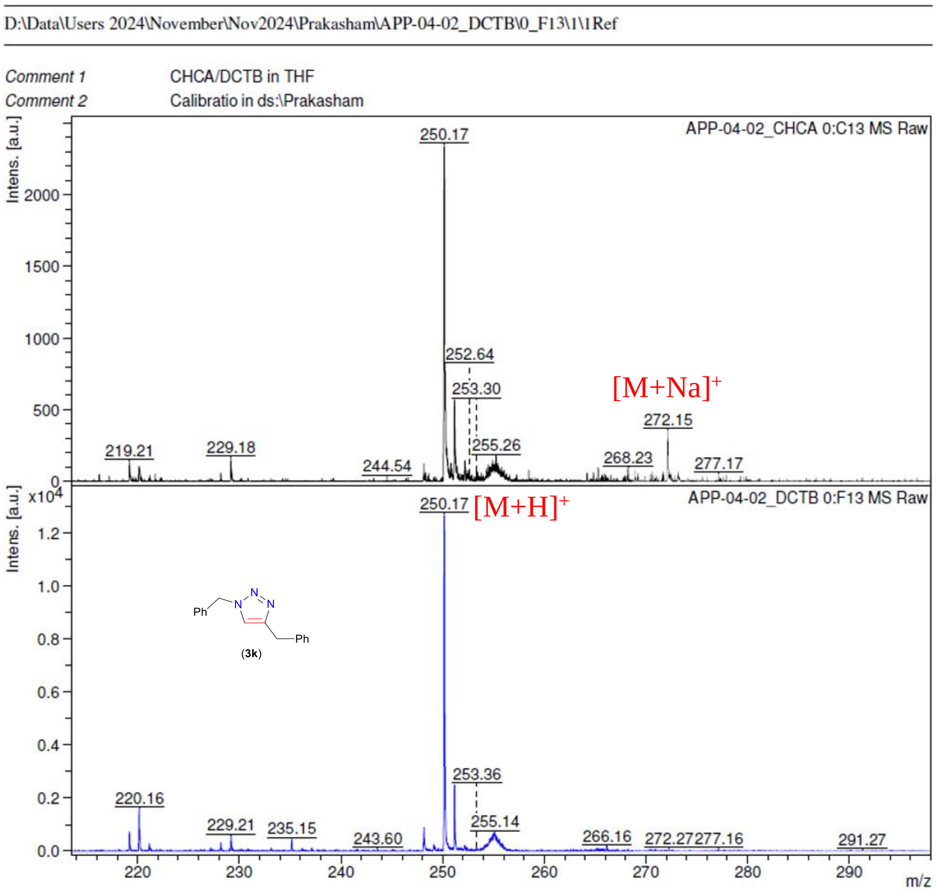

Figure S54. MALDI-ToF-MS spectra of 1,4-dibenzyl-1H-1,2,3-triazole (**3k**).

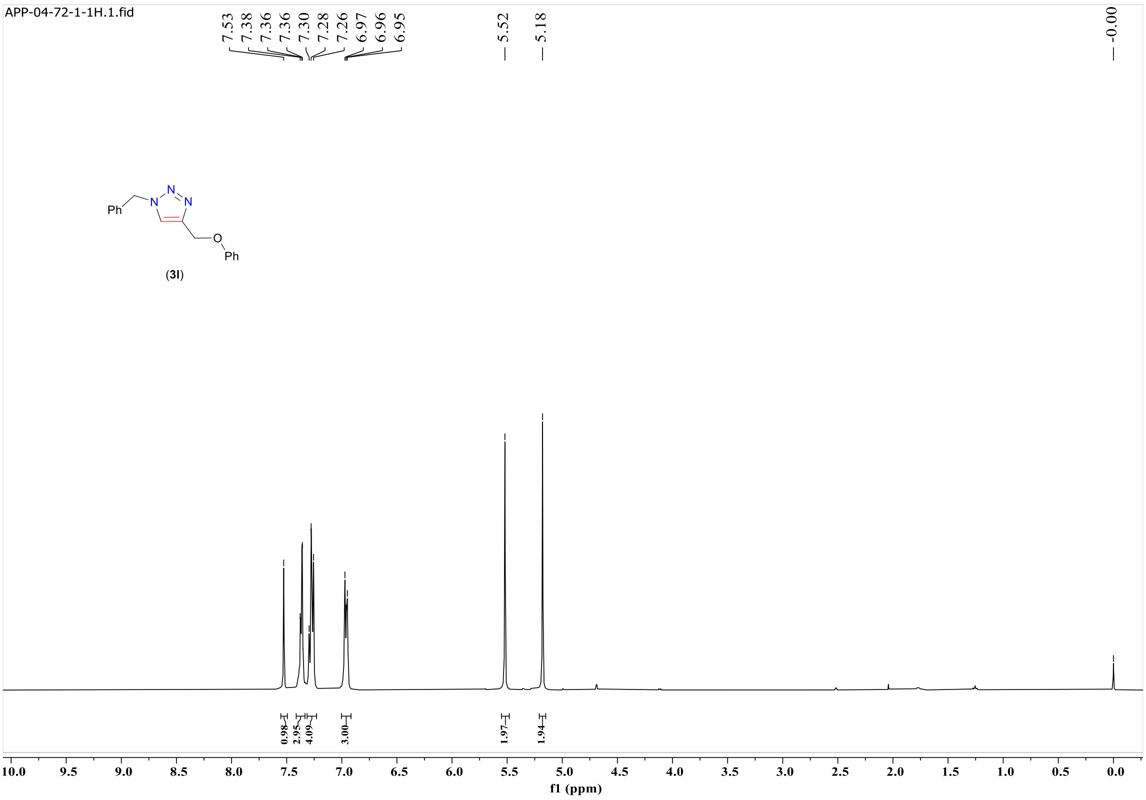

Figure S55. ^{1}H NMR of 1-benzyl-4-(phenoxymethyl)-1H-1,2,3-triazole (**3l**) in $CDCl_3$.

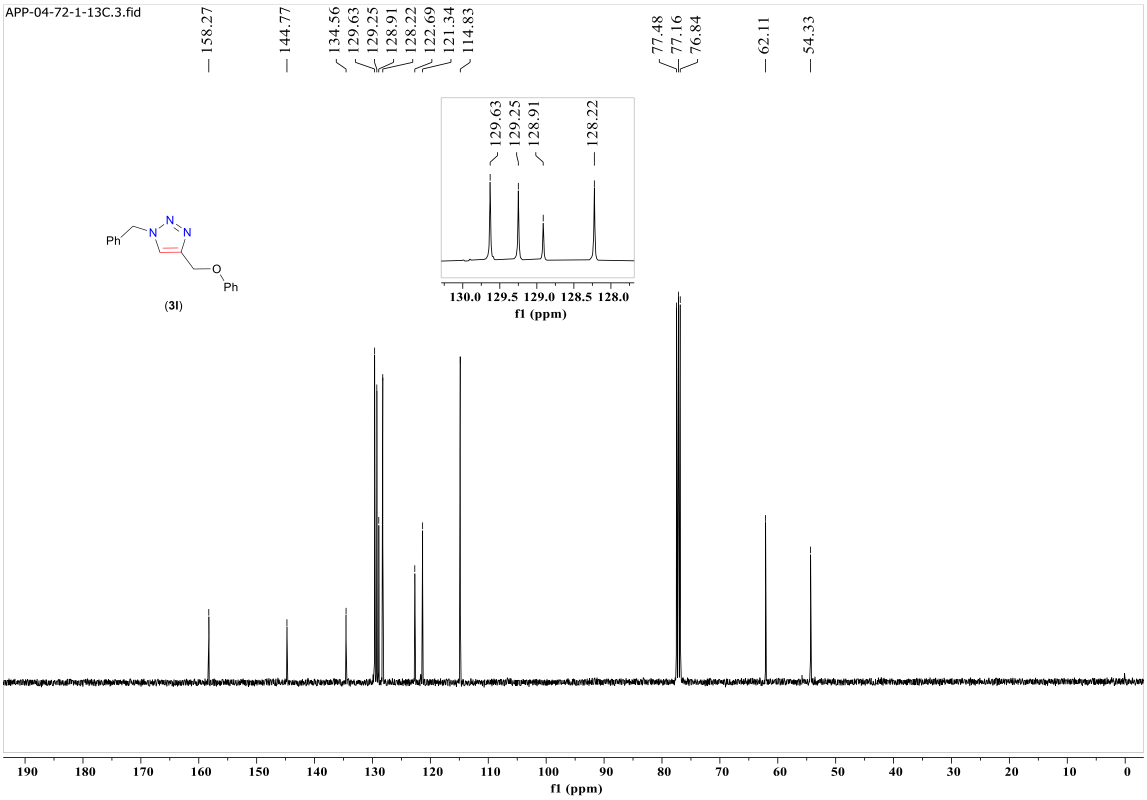

Figure S56. ^{13}C{^{1}H} NMR of 1-benzyl-4-(phenoxymethyl)-1H-1,2,3-triazole (**3l**) in $CDCl_3$.

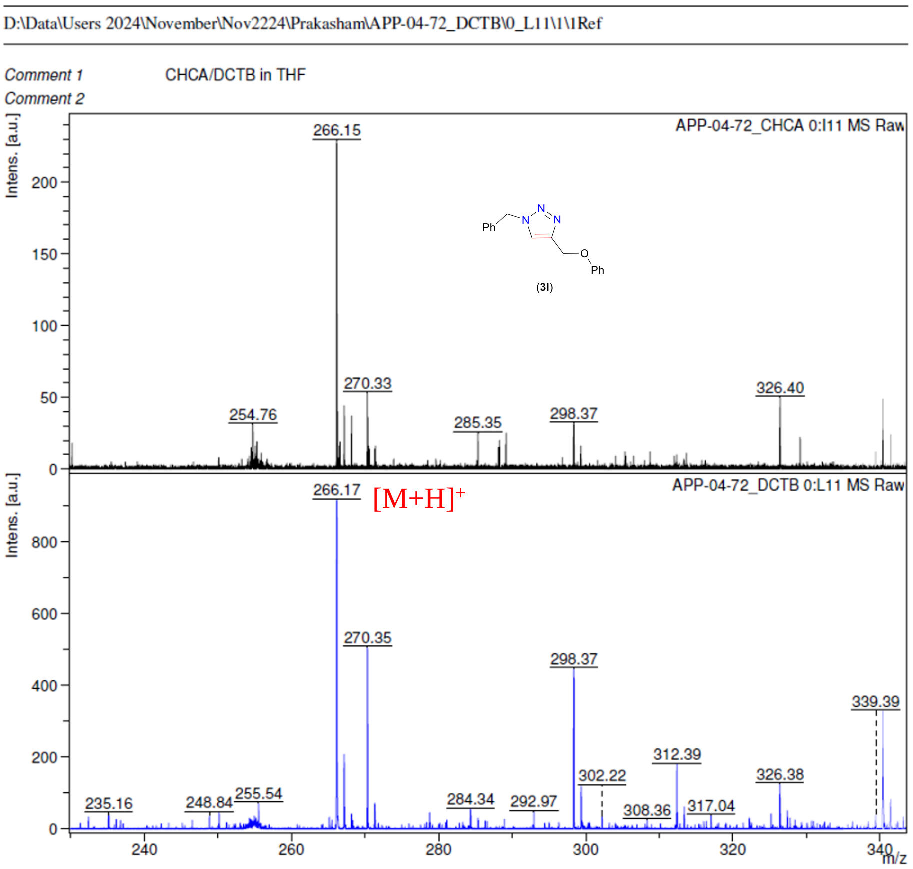

Figure S57. MALDI-ToF-MS spectra of 1-benzyl-4-(phenoxymethyl)-1H-1,2,3-triazole (**3l**).

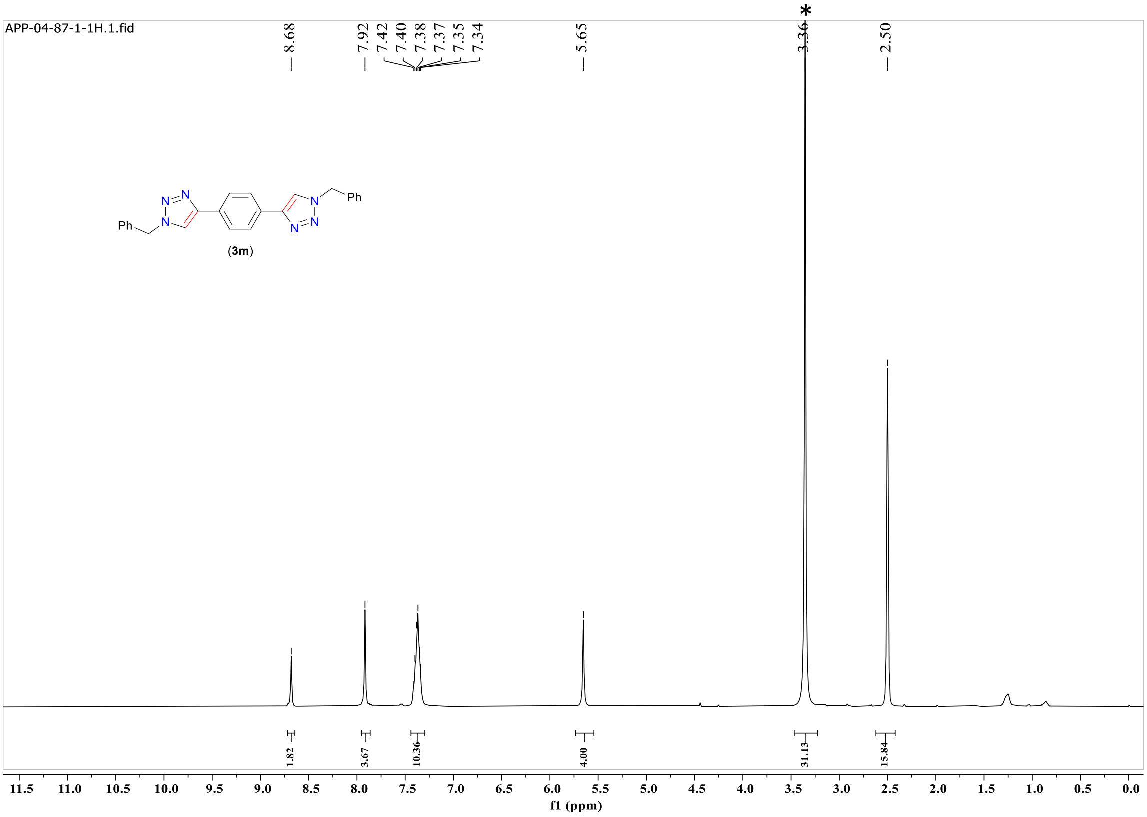

Figure S58. ^{1}H NMR of 1,4-bis(1-benzyl-1H-1,2,3-triazol-4-yl)benzene (**3m**) in DMSO-d_6. * H_2O.

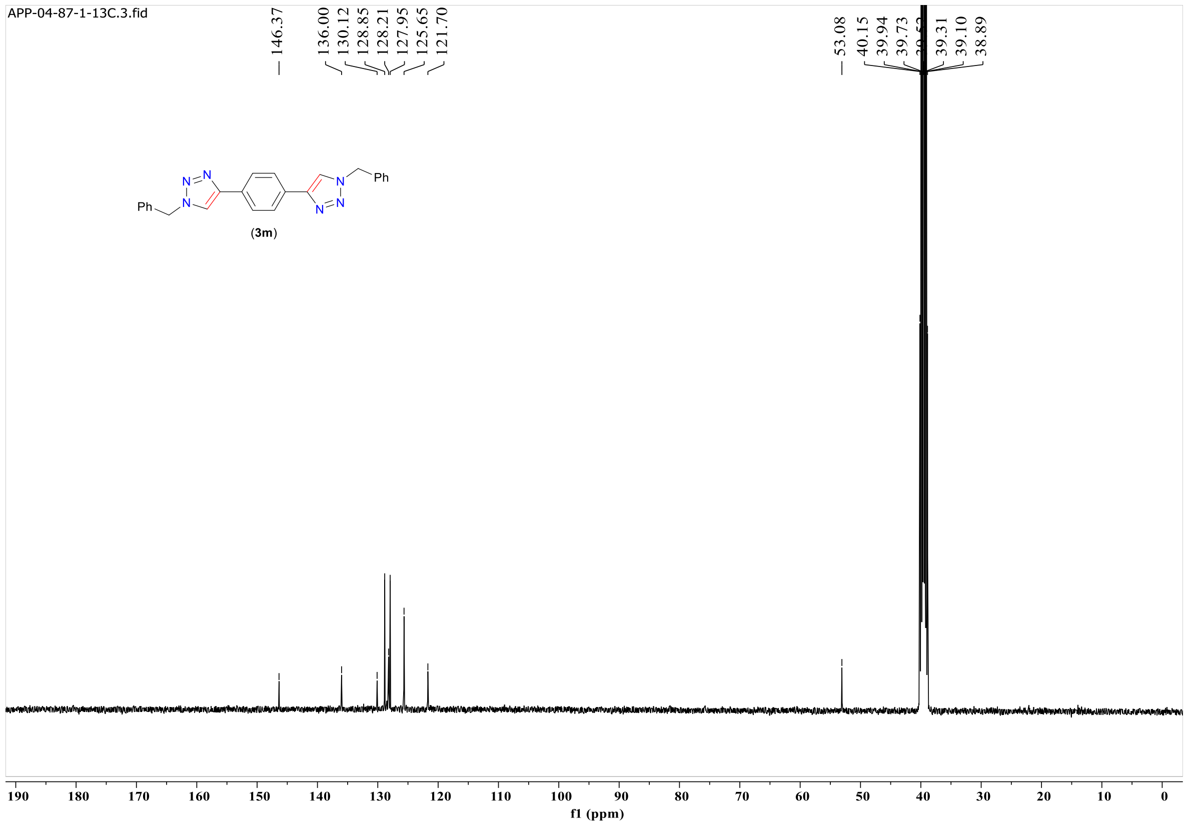

Figure S59. $^{13}C\{^1H\}$ NMR of 1,4-bis(1-benzyl-1H-1,2,3-triazol-4-yl)benzene (**3m**) in DMSO-d_6.

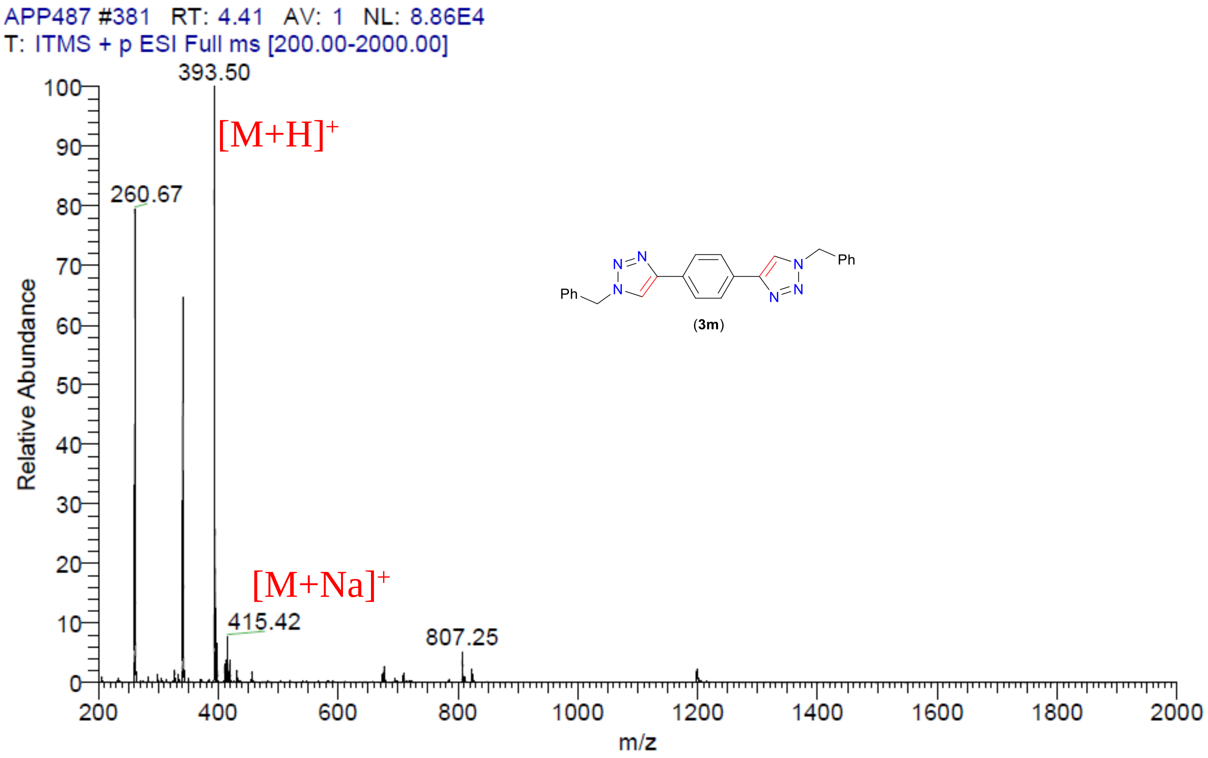

Figure S60. LCMS (ESI) trace of 1,4-bis(1-benzyl-1H-1,2,3-triazol-4-yl)benzene (**3m**).

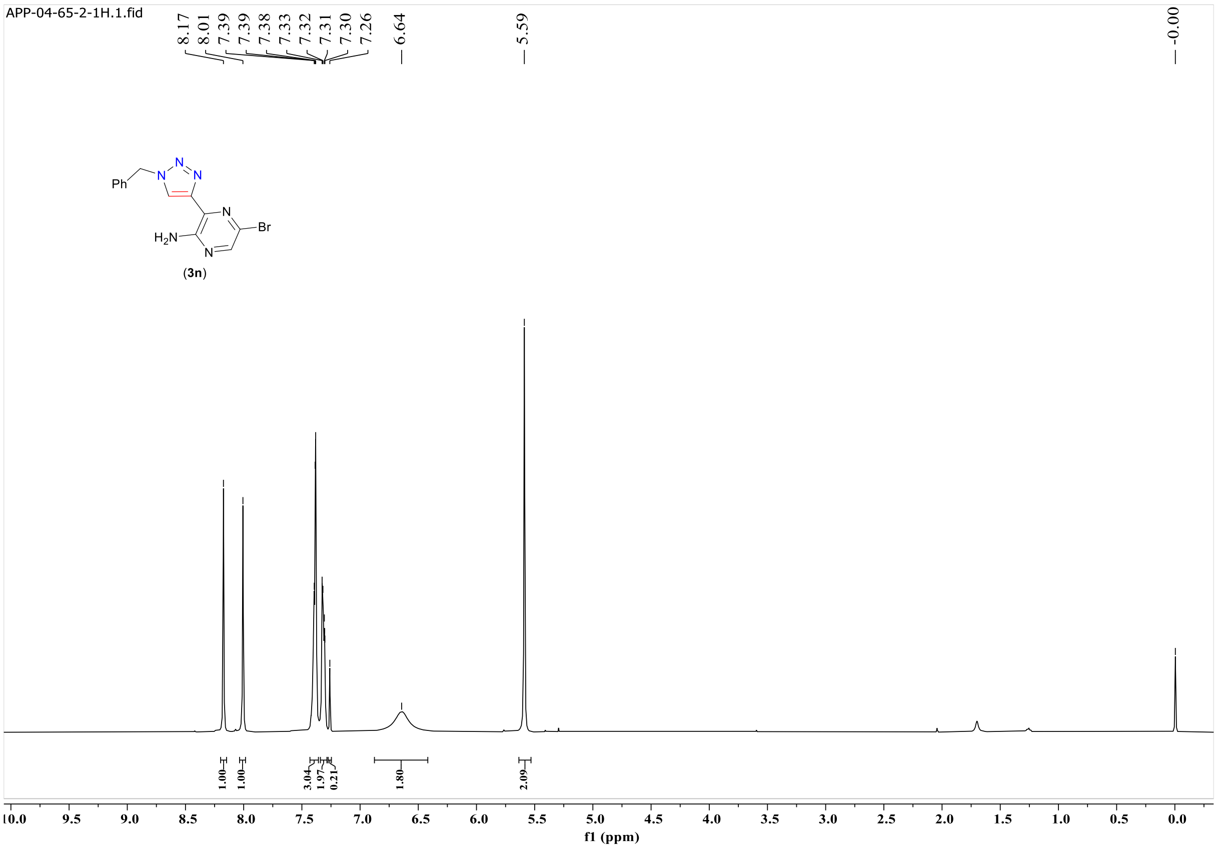

Figure S61. ^{1}H NMR of 3-(1-benzyl-1H-1,2,3-triazol-4-yl)-5-bromopyrazin-2-amine (**3n**) in $CDCl_3$.

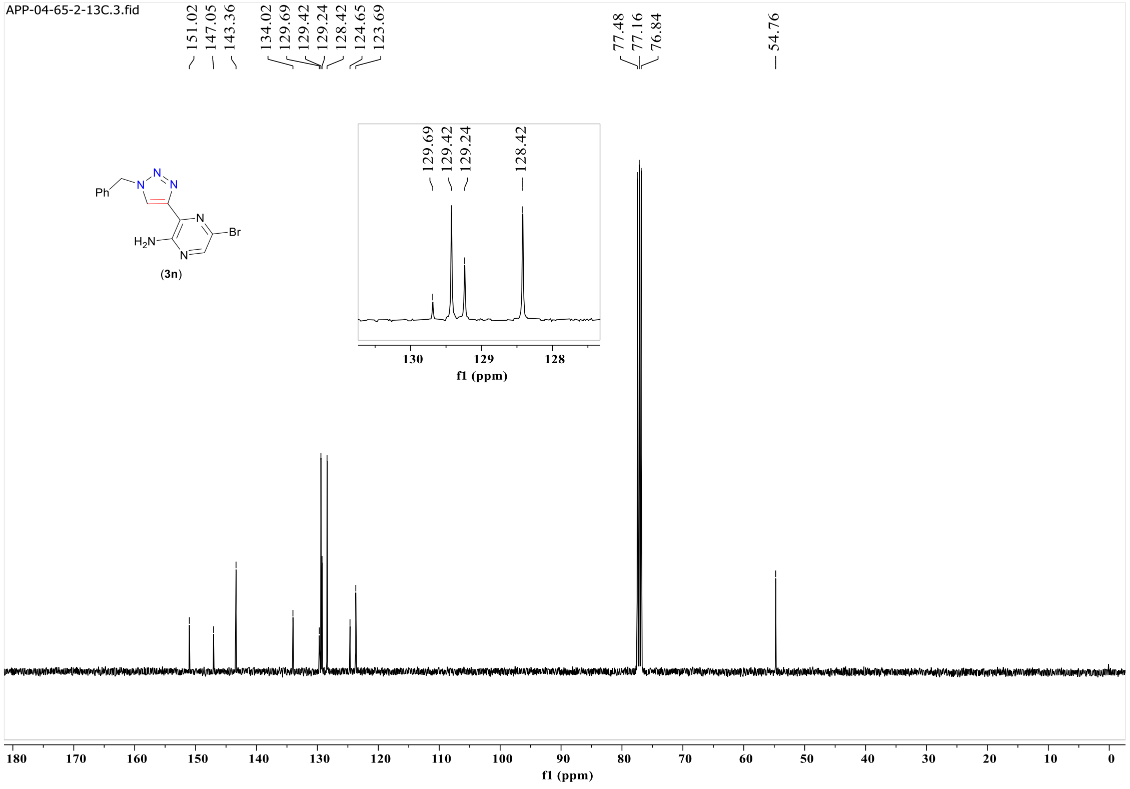

Figure S62. $^{13}C\{^{1}H\}$ NMR of 3-(1-benzyl-1H-1,2,3-triazol-4-yl)-5-bromopyrazin-2-amine (**3n**) in $CDCl_3$.

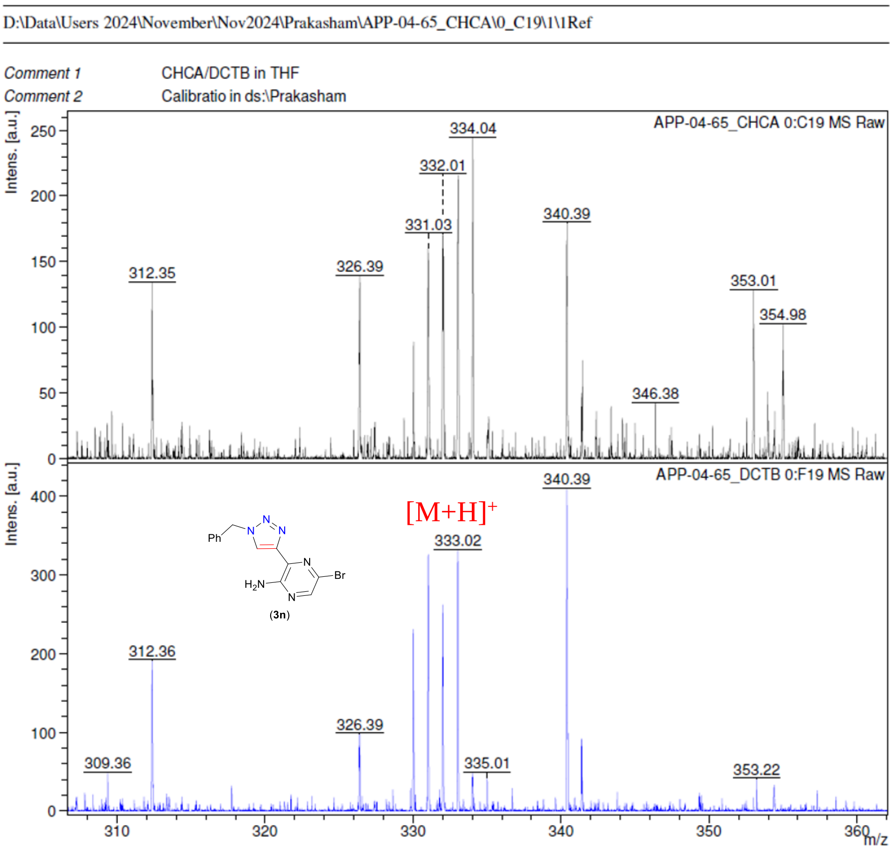

Figure S63. MALDI-ToF-MS spectra of 3-(1-benzyl-1H-1,2,3-triazol-4-yl)-5-bromopyrazin-2-amine (**3n**).

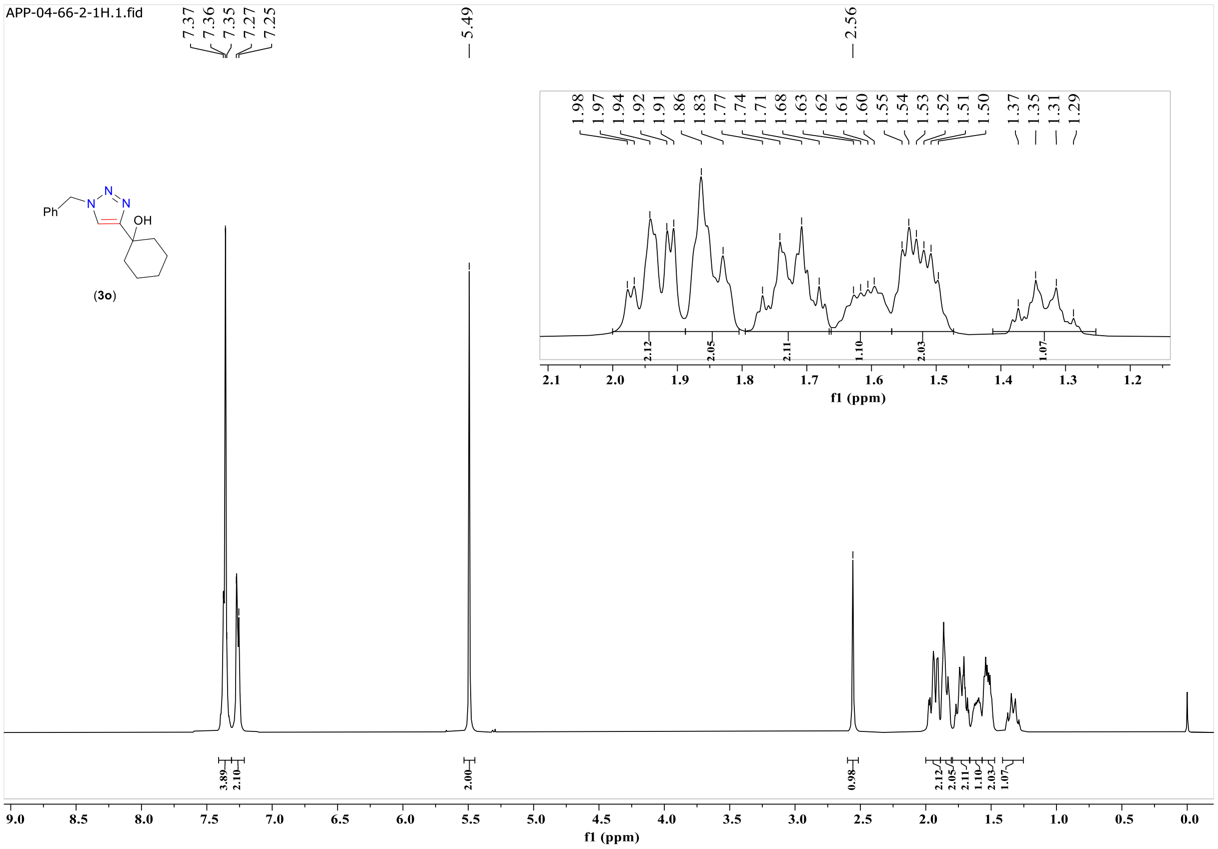

Figure S64. ^{1}H NMR of 1-(1-benzyl-1H-1,2,3-triazol-4-yl)cyclohexan-1-ol (**3o**) in $CDCl_3$.

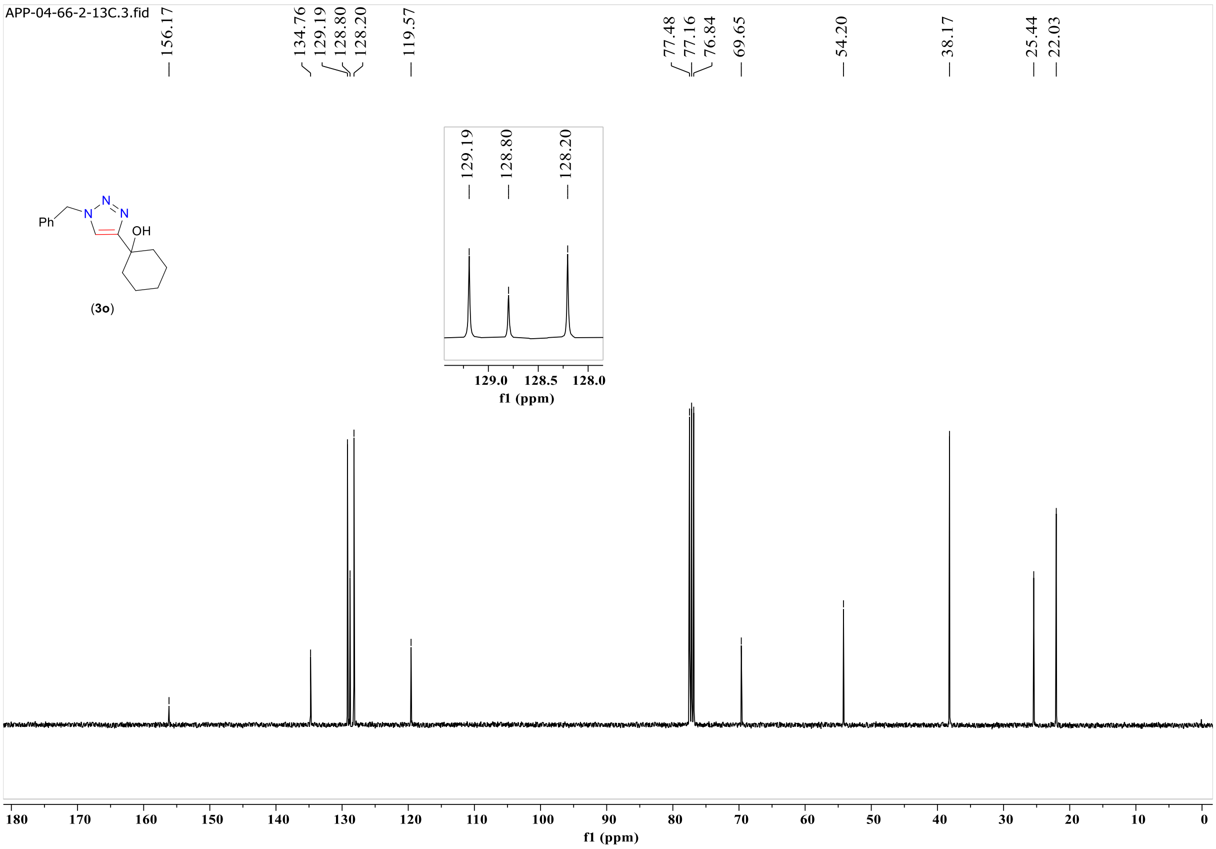

Figure S65. $^{13}C\{^1H\}$ NMR of 1-(1-benzyl-1H-1,2,3-triazol-4-yl)cyclohexan-1-ol (**3o**) in $CDCl_3$.

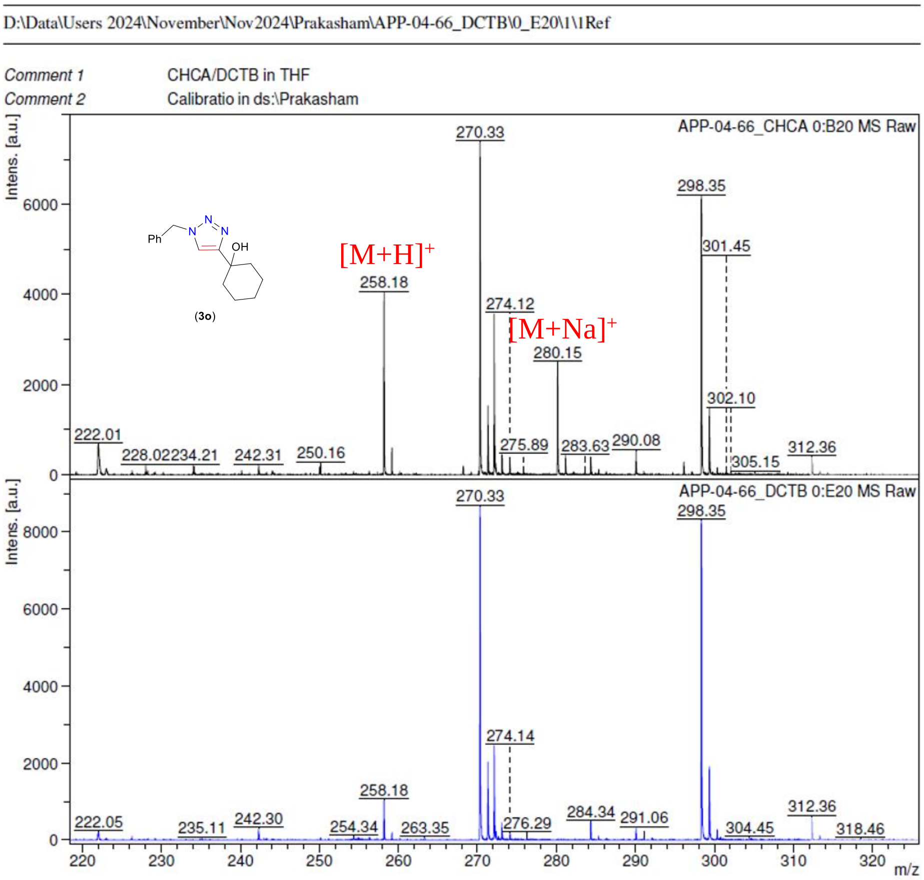

Figure S66. MALDI-ToF-MS spectra of 1-(1-benzyl-1H-1,2,3-triazol-4-yl)cyclohexan-1-ol (**3o**).

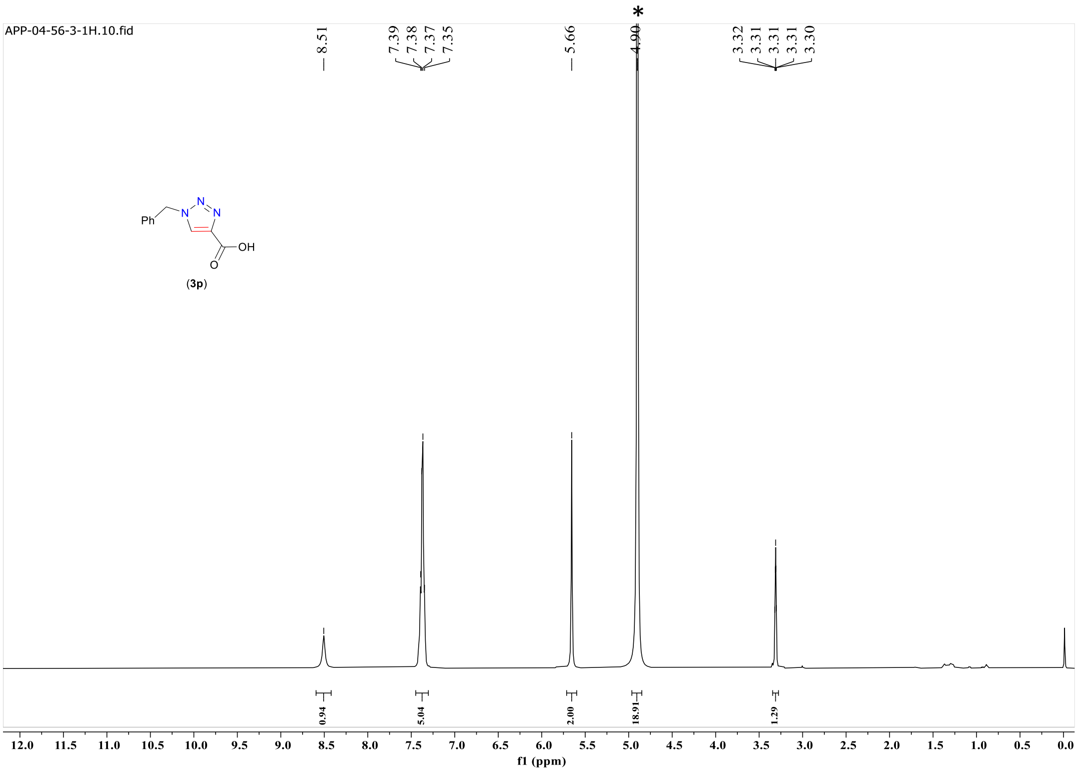

Figure S67. ^{1}H NMR of 1-benzyl-1H-1,2,3-triazole-4-carboxylic acid (**3p**) in CD_3OD. * H_2O.

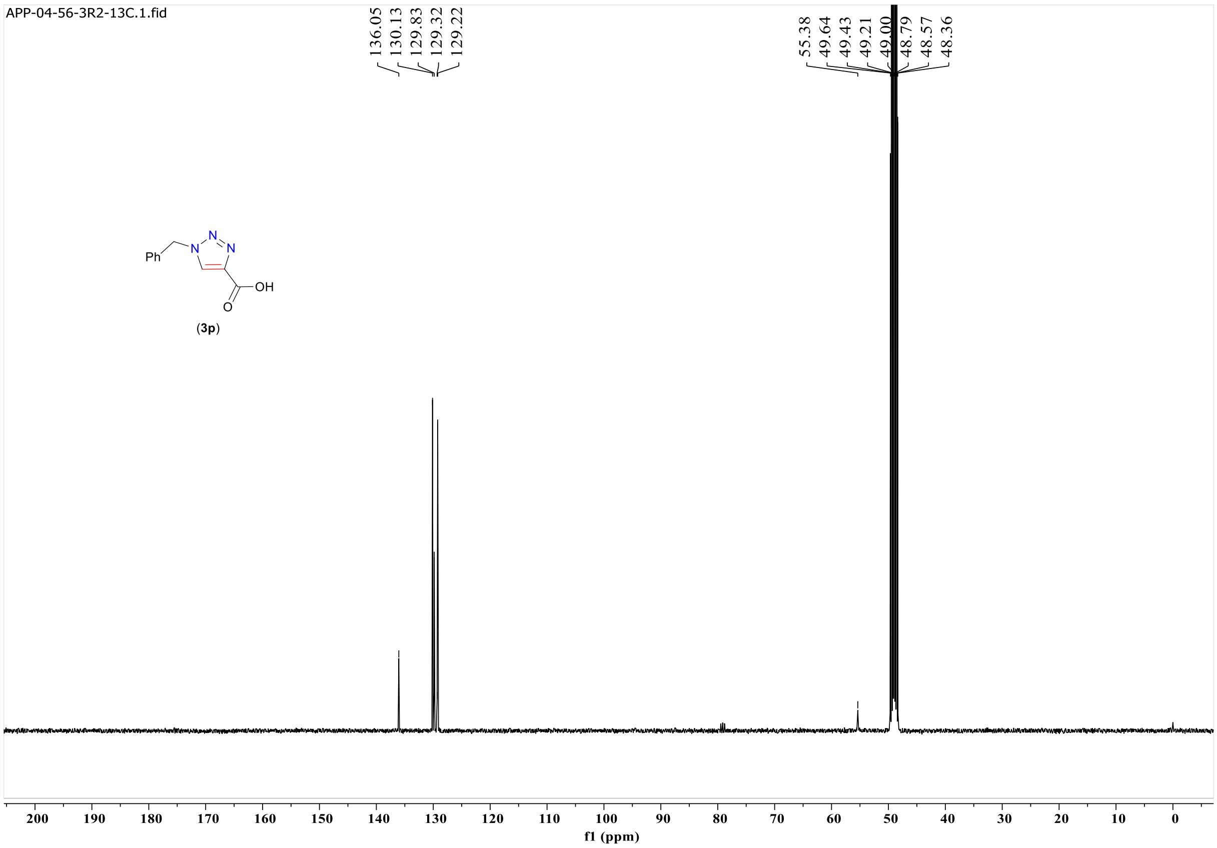

Figure S68. ^{13}C{^{1}H} NMR of 1-benzyl-1H-1,2,3-triazole-4-carboxylic acid (**3p**) in CD_3OD.

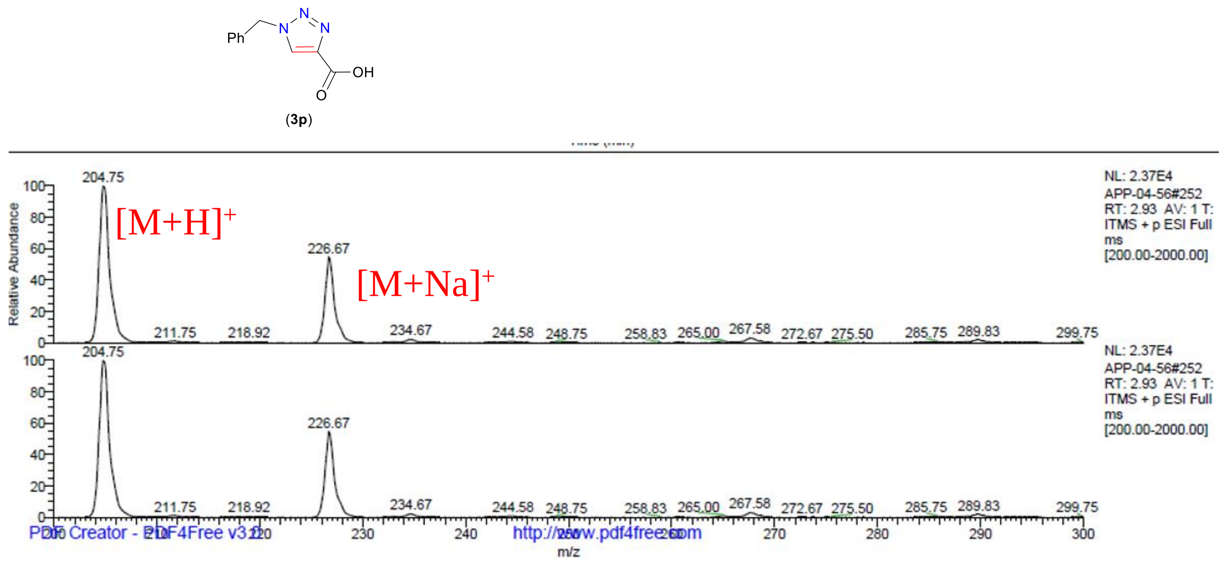

Figure S69. LCMS (ESI) trace of 1-benzyl-1H-1,2,3-triazole-4-carboxylic acid (**3p**).

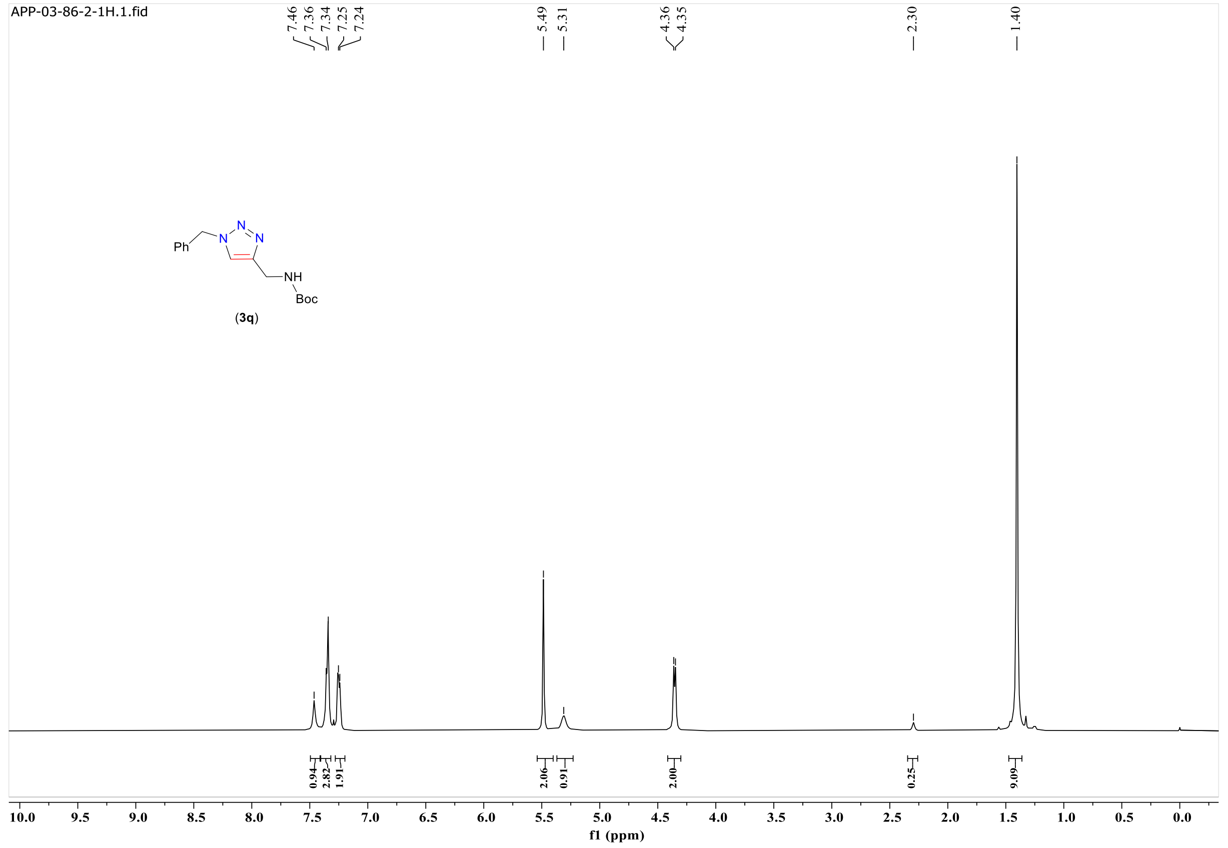

Figure S70. ^{1}H NMR of *tert*-butyl ((1-benzyl-1H-1,2,3-triazol-4-yl)methyl)carbamate (**3q**) in $CDCl_3$.

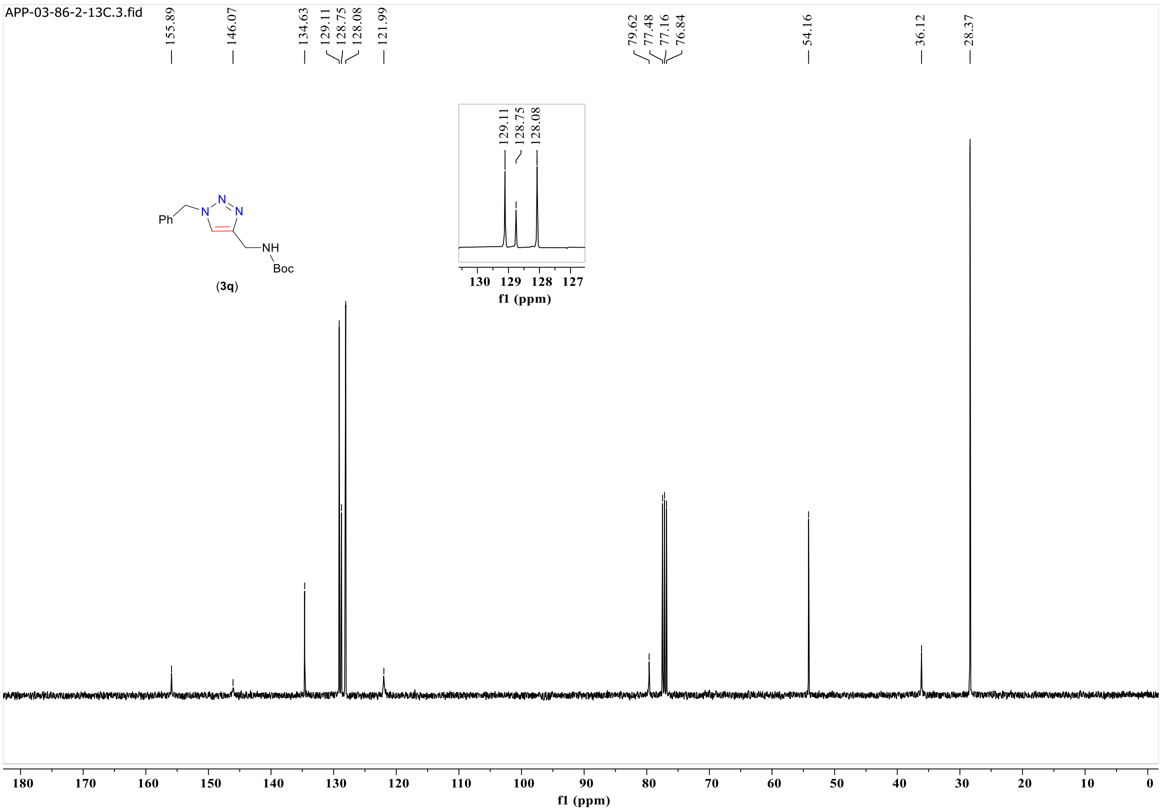

Figure S71. $^{13}C\{^{1}H\}$ NMR of *tert*-butyl ((1-benzyl-1H-1,2,3-triazol-4-yl)methyl)carbamate (**3q**) in $CDCl_3$.

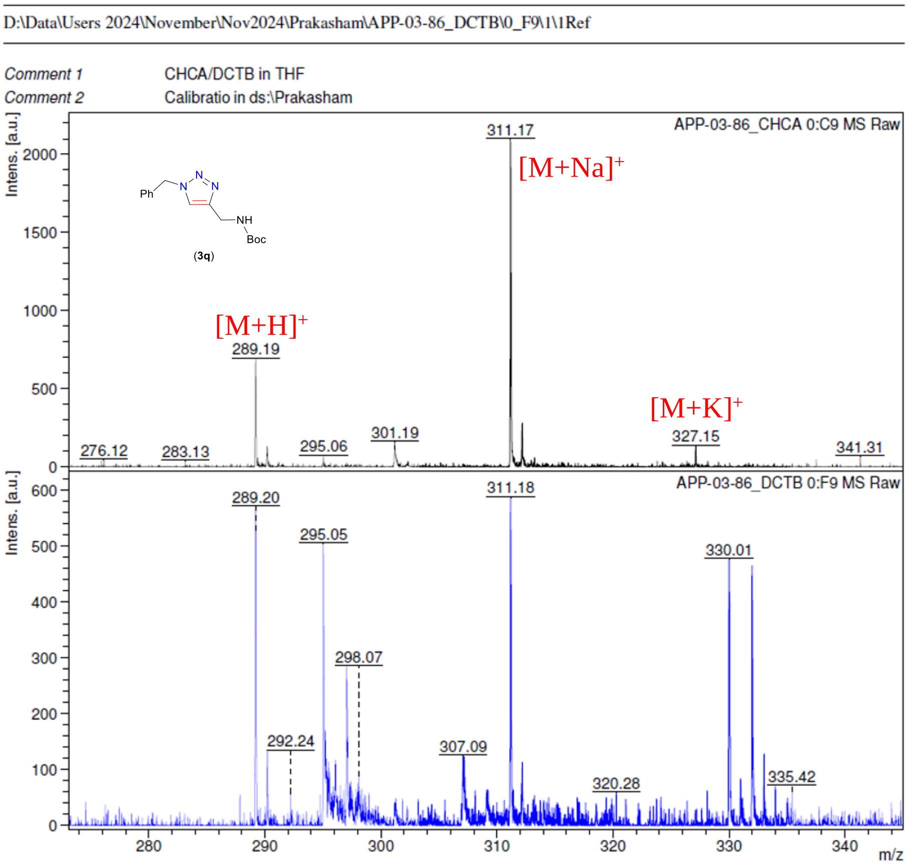

Figure S72. MALDI-ToF-MS spectra of *tert*-butyl ((1-benzyl-1H-1,2,3-triazol-4-yl)methyl)carbamate (**3q**).

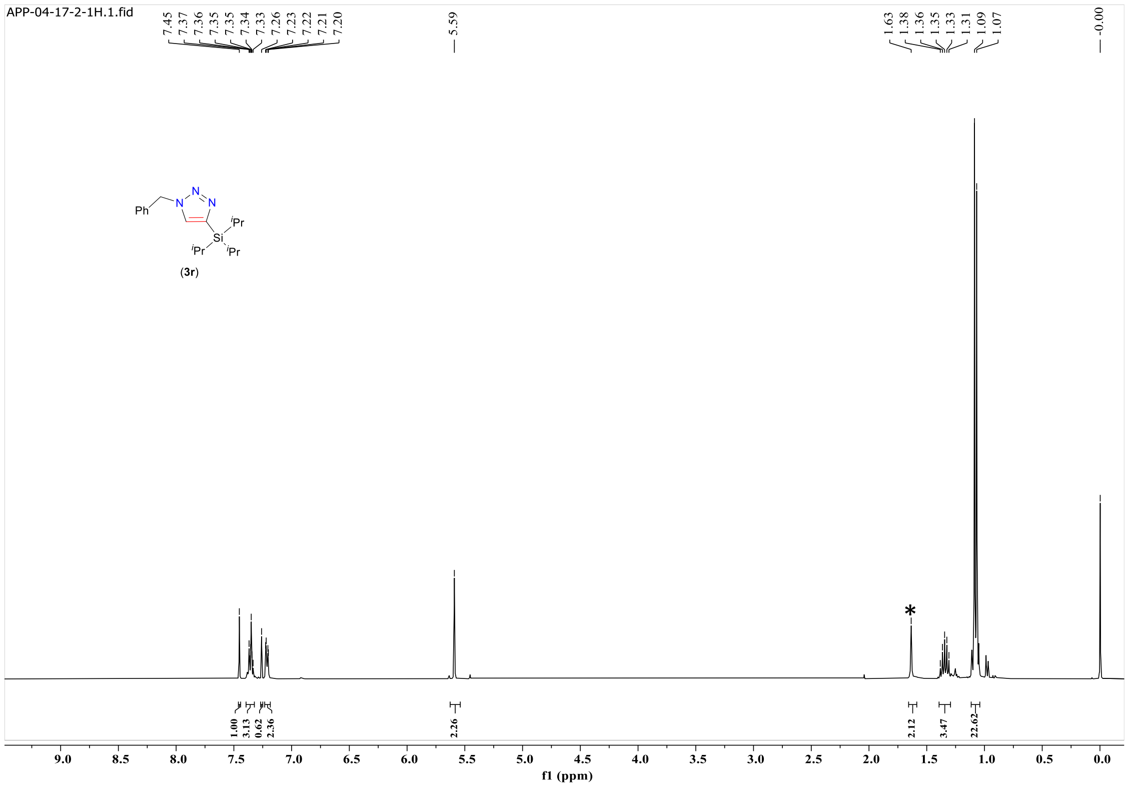

Figure S73. ^{1}H NMR of 1-benzyl-4-(triisopropylsilyl)-1H-1,2,3-triazole (**3r**) in $CDCl_3$. * H_2O.

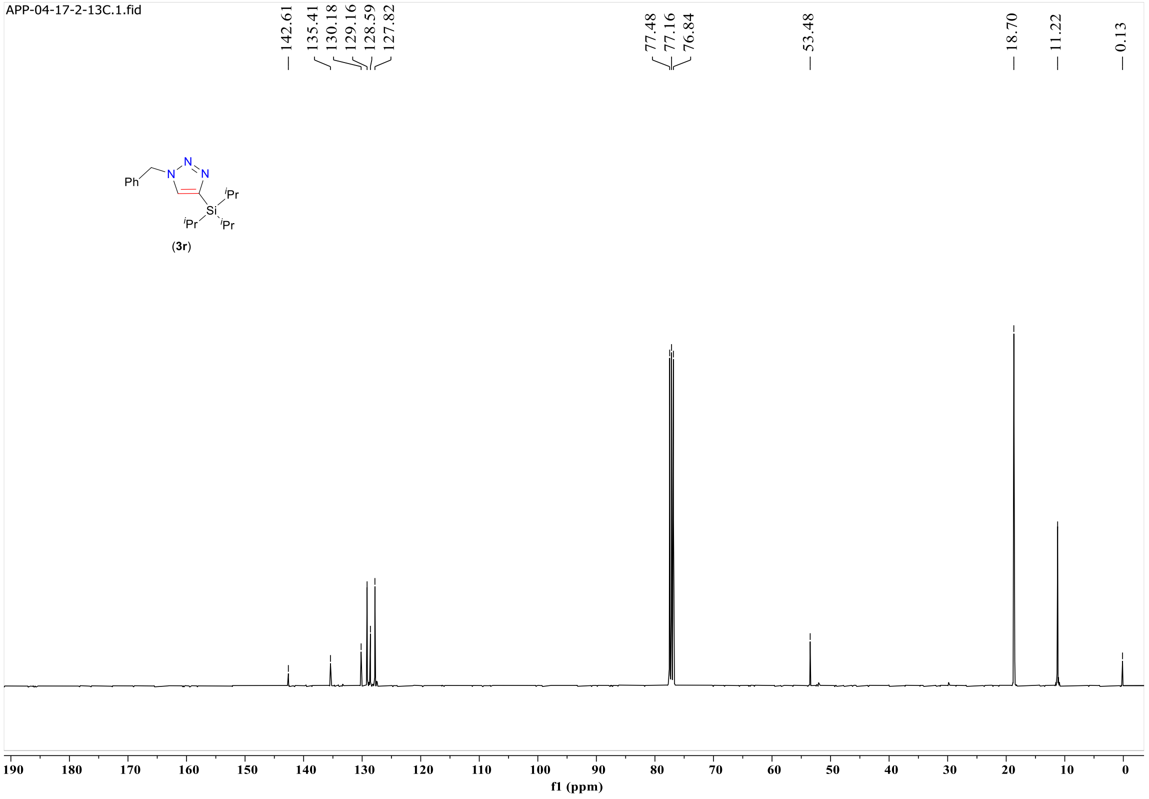

Figure S74. $^{13}C\{^{1}H\}$ NMR of 1-benzyl-4-(triisopropylsilyl)-1H-1,2,3-triazole (**3r**) in $CDCl_3$.

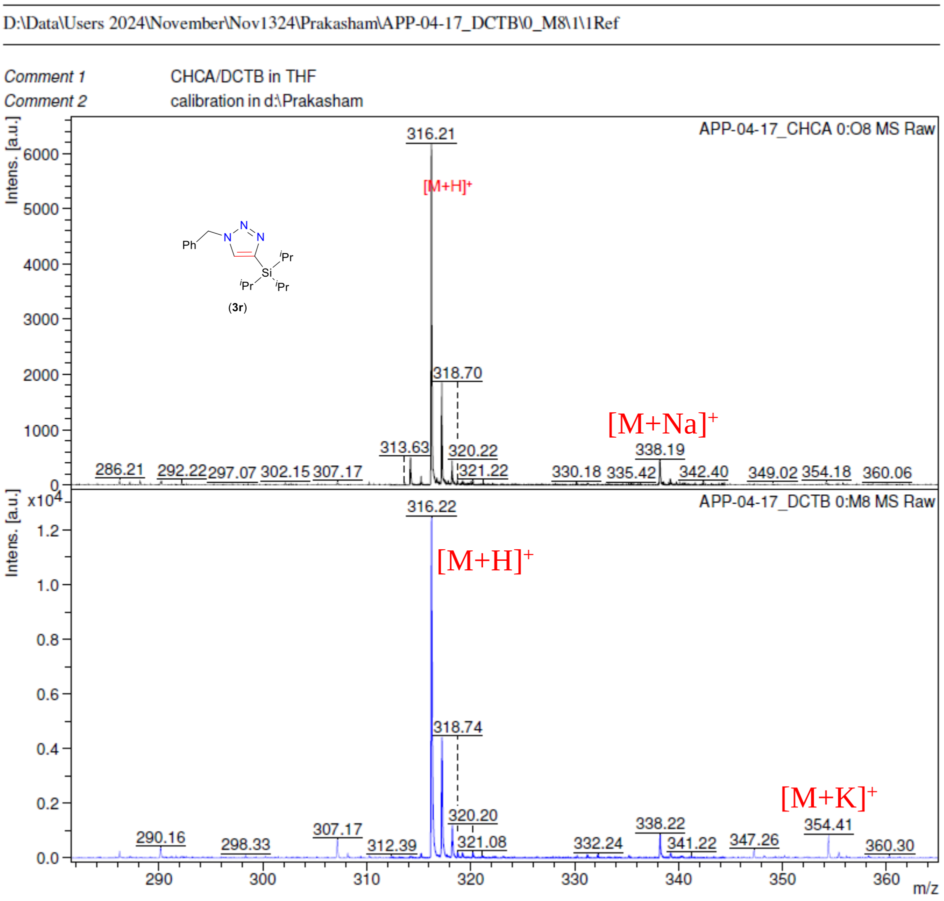

Figure S75. MALDI-ToF-MS spectra of 1-benzyl-4-(triisopropylsilyl)-1H-1,2,3-triazole (**3r**).

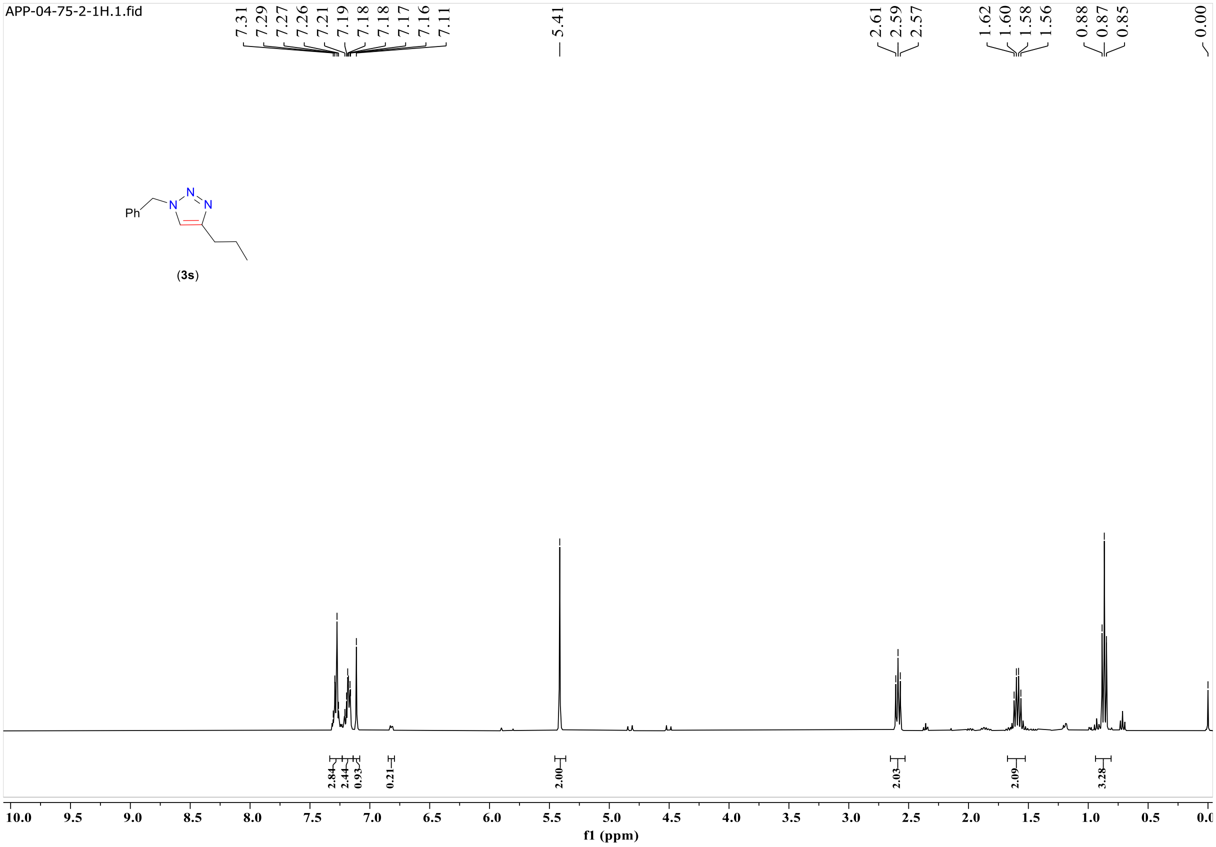

Figure S76. ^{1}H NMR of 1-benzyl-4-propyl-1H-1,2,3-triazole (**3s**) in $CDCl_3$.

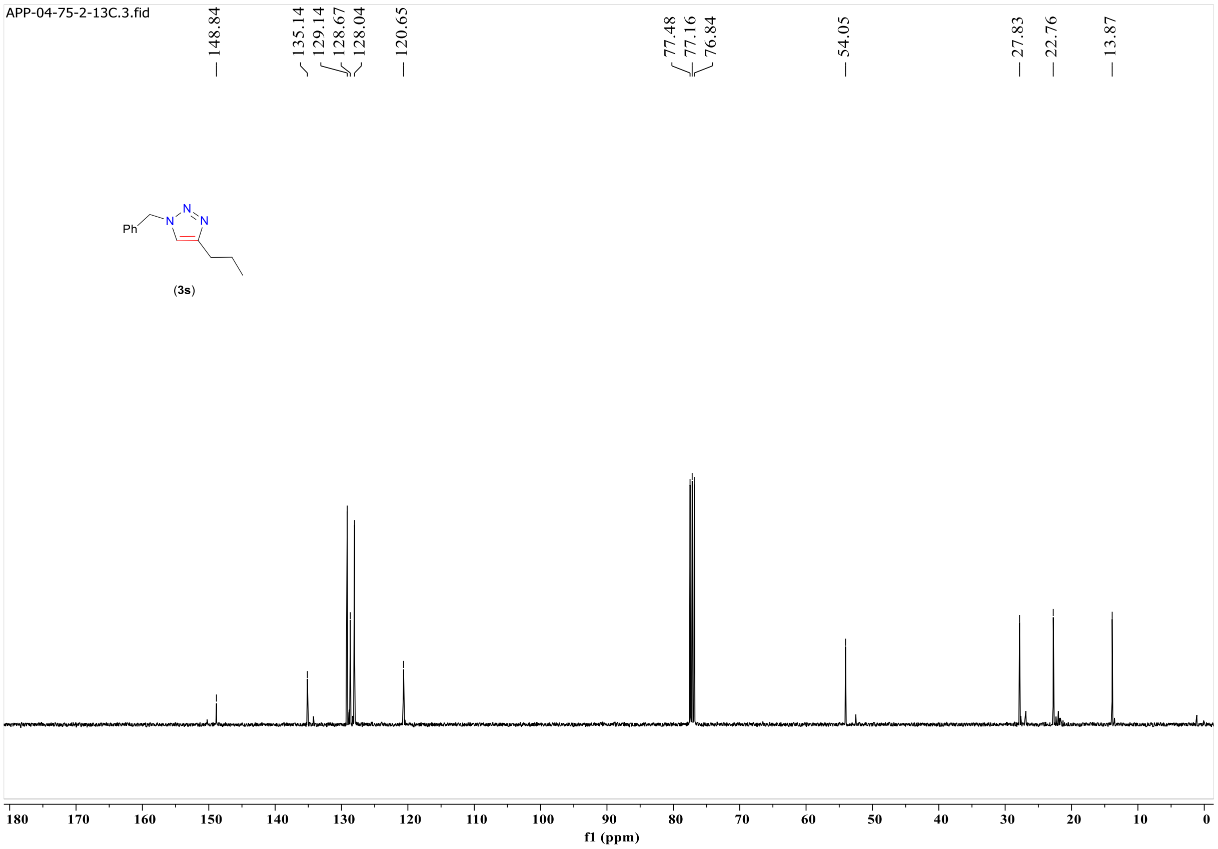

Figure S77. $^{13}C\{^{1}H\}$ NMR of 1-benzyl-4-propyl-1H-1,2,3-triazole (**3s**) in $CDCl_3$.

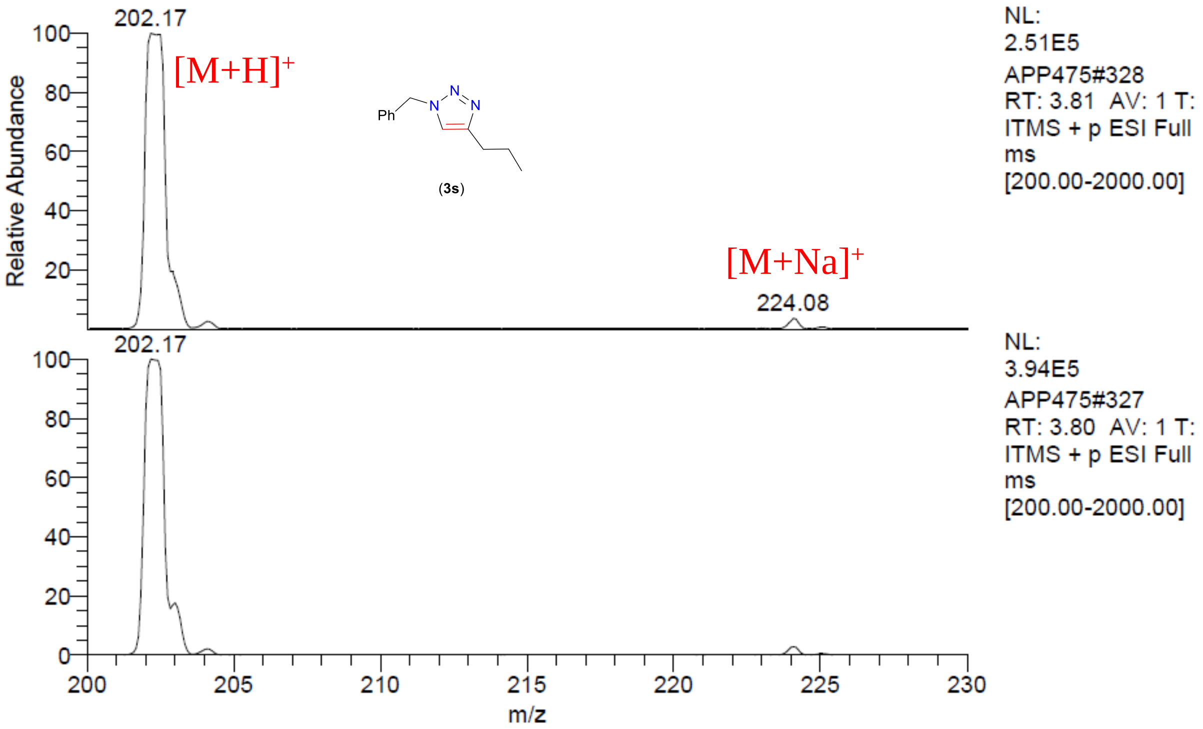

Figure S78. LCMS (ESI) trace of 1-benzyl-4-propyl-1H-1,2,3-triazole (**3s**).

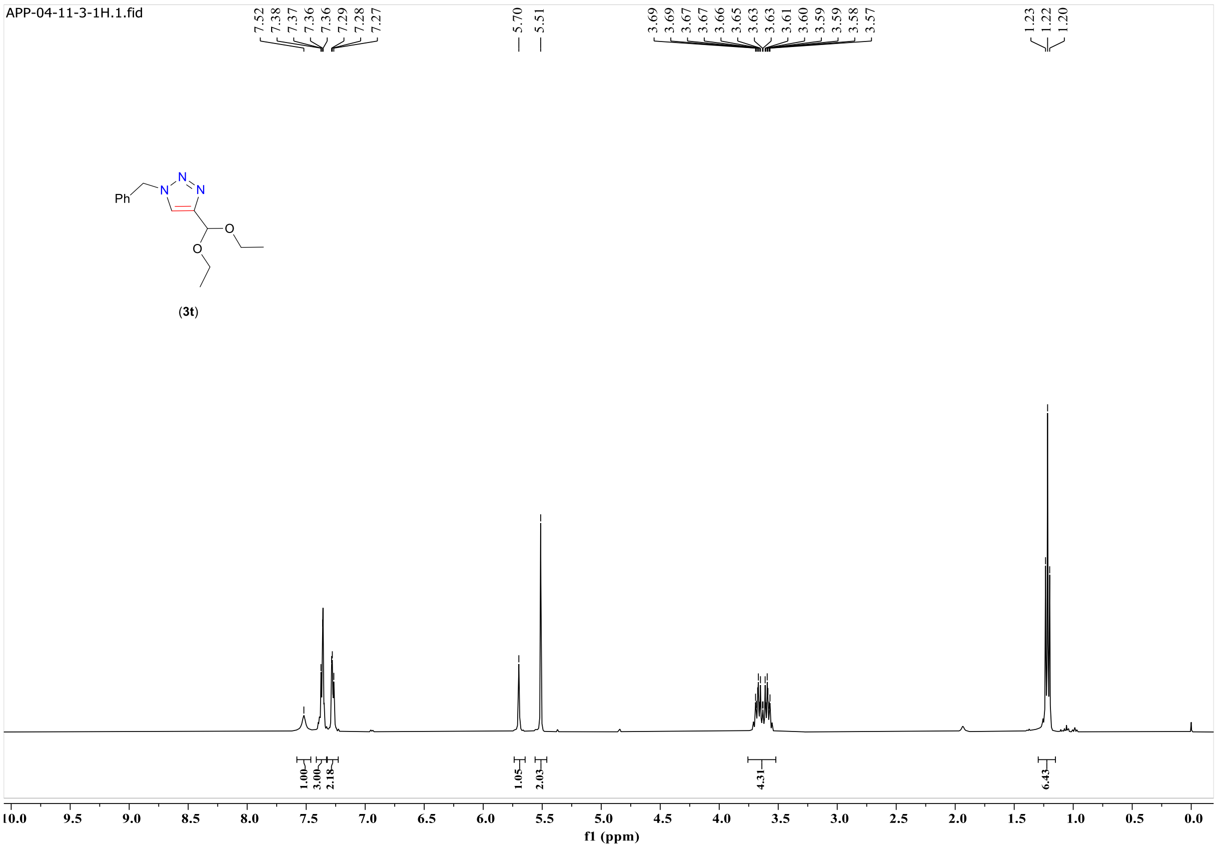

Figure S79. ^{1}H NMR of 1-benzyl-4-(diethoxymethyl)-1H-1,2,3-triazole (**3t**) in $CDCl_3$.

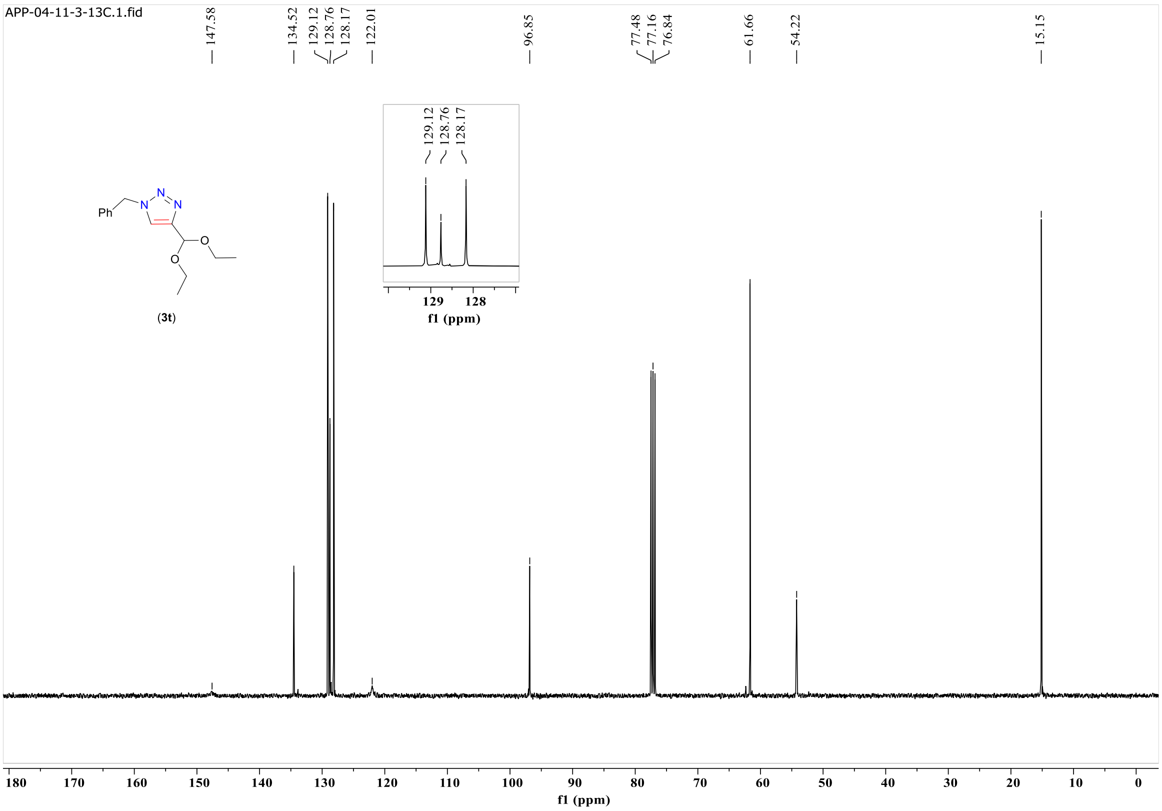

Figure S80. ^{13}C{^{1}H} NMR of 1-benzyl-4-(diethoxymethyl)-1H-1,2,3-triazole (**3t**) in $CDCl_3$.

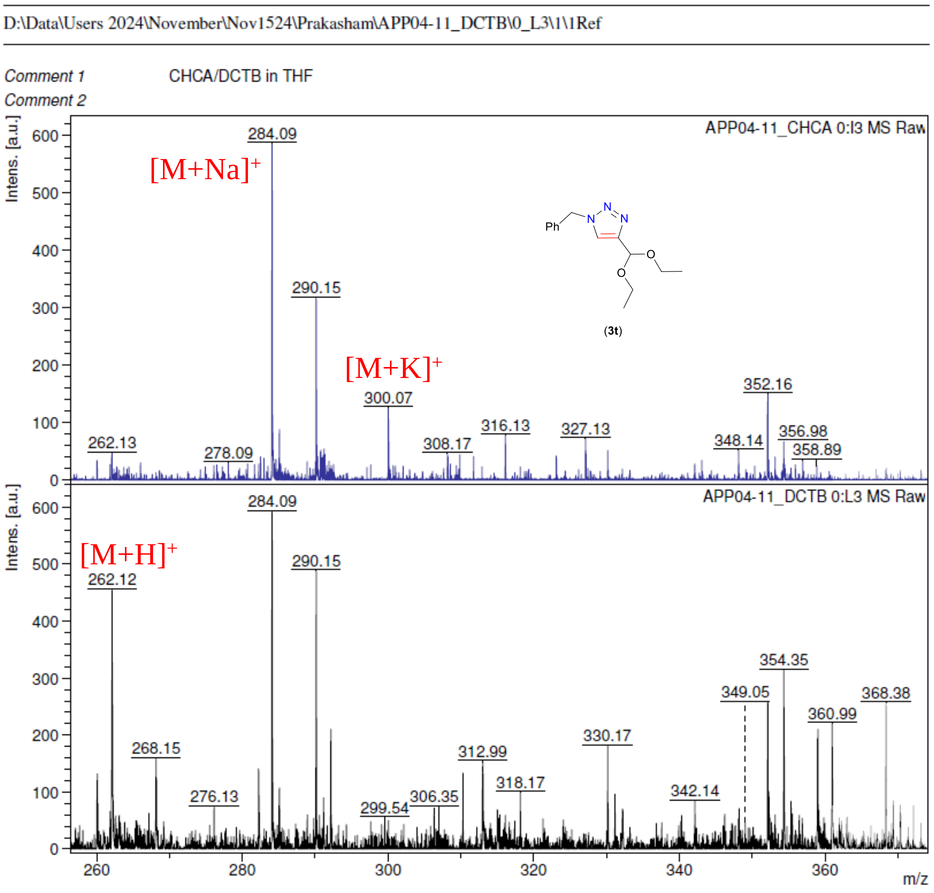

Figure S81. MALDI-ToF-MS spectra of 1-benzyl-4-(diethoxymethyl)-1H-1,2,3-triazole (**3t**).

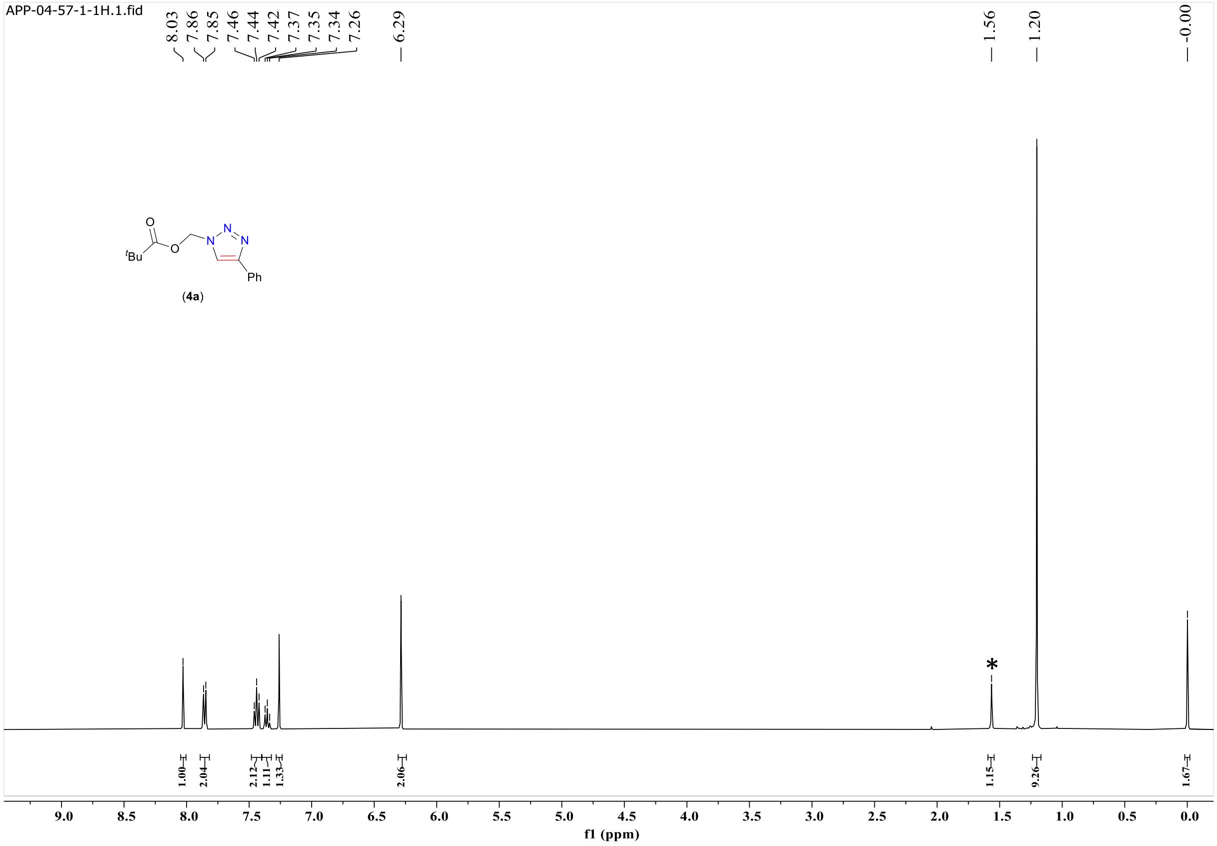

Figure S82. ^{1}H NMR of (4-phenyl-1H-1,2,3-triazol-1-yl)methyl pivalate (**4a**) in $CDCl_3$. * H_2O.

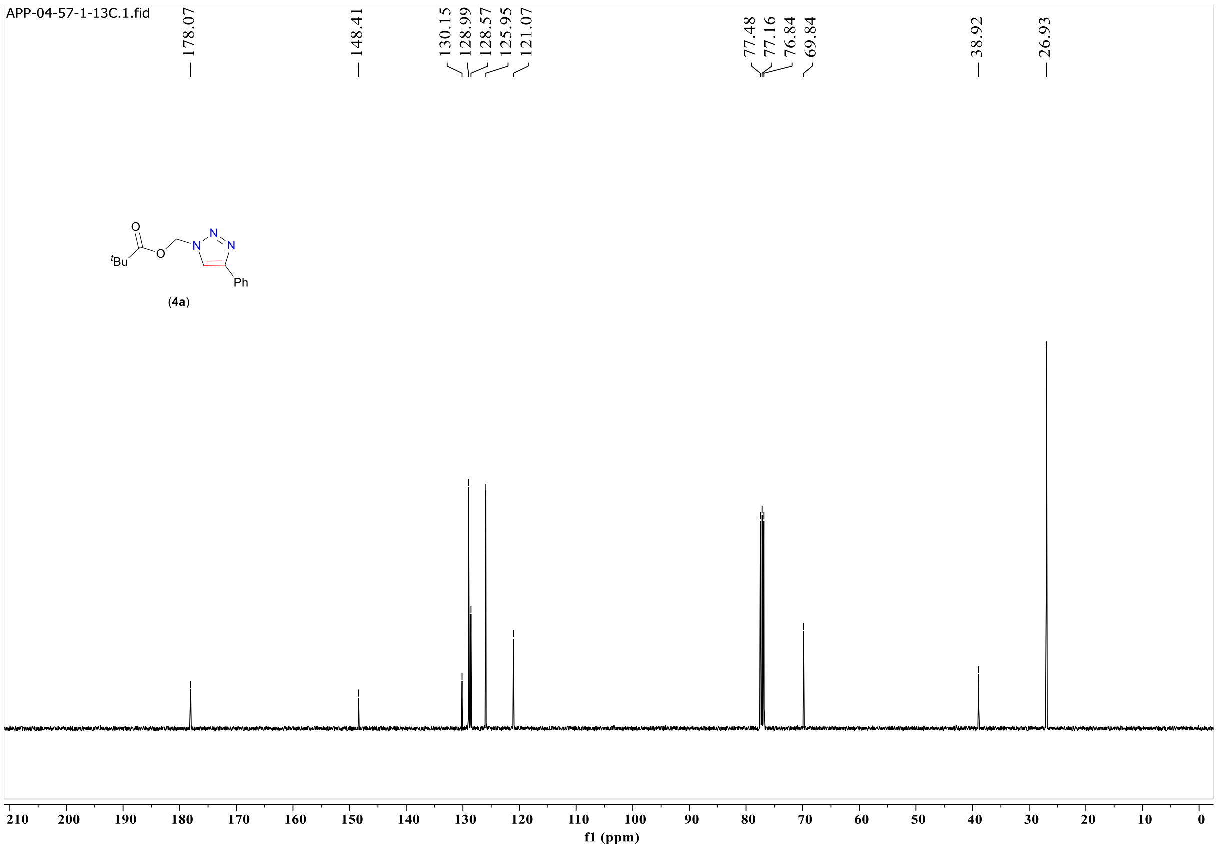

Figure S83. $^{13}C\{^{1}H\}$ NMR of (4-phenyl-1H-1,2,3-triazol-1-yl)methyl pivalate (**4a**) in $CDCl_3$.

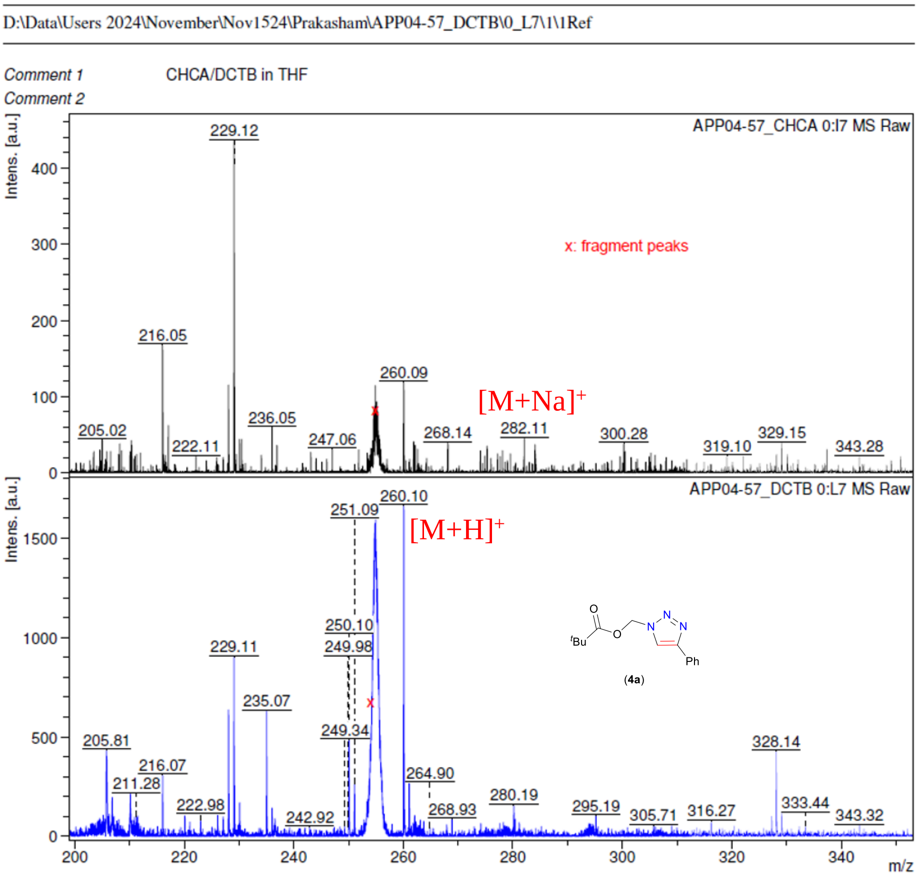

Figure S84. MALDI-ToF-MS spectra of (4-phenyl-1H-1,2,3-triazol-1-yl)methyl pivalate (**4a**).

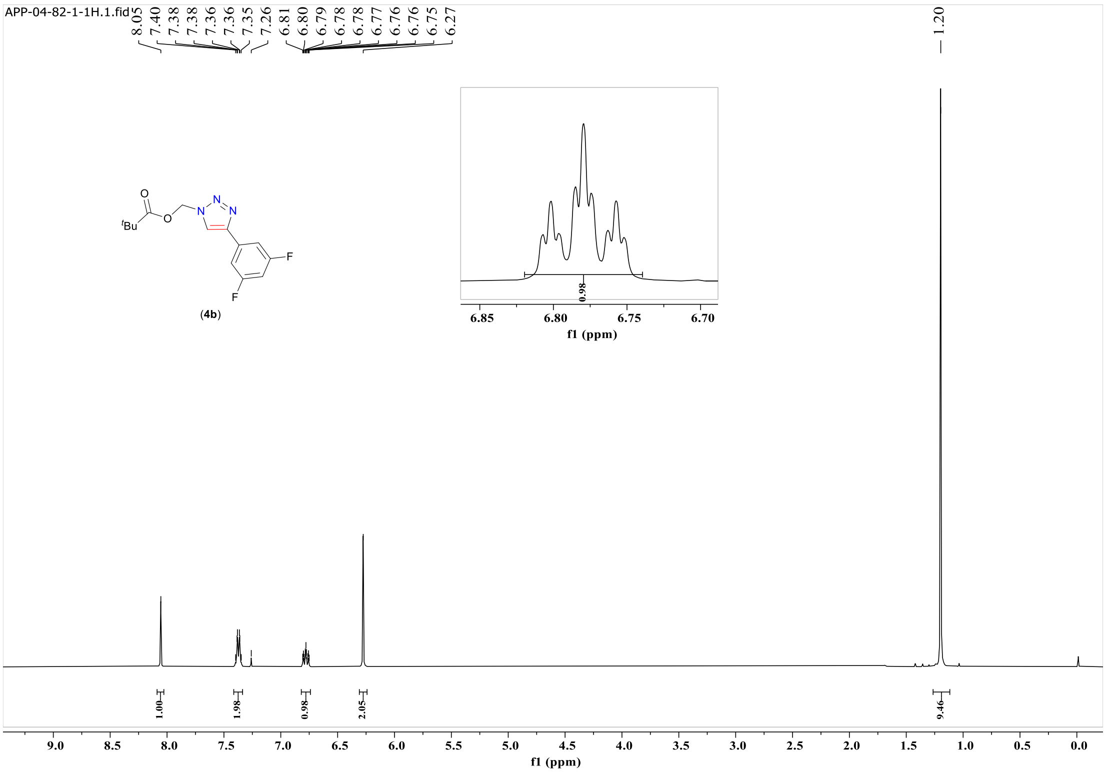

Figure S85. ^{1}H NMR of (4-(3,5-difluorophenyl)-1H-1,2,3-triazol-1-yl)methyl pivalate (**4b**) in $CDCl_3$.

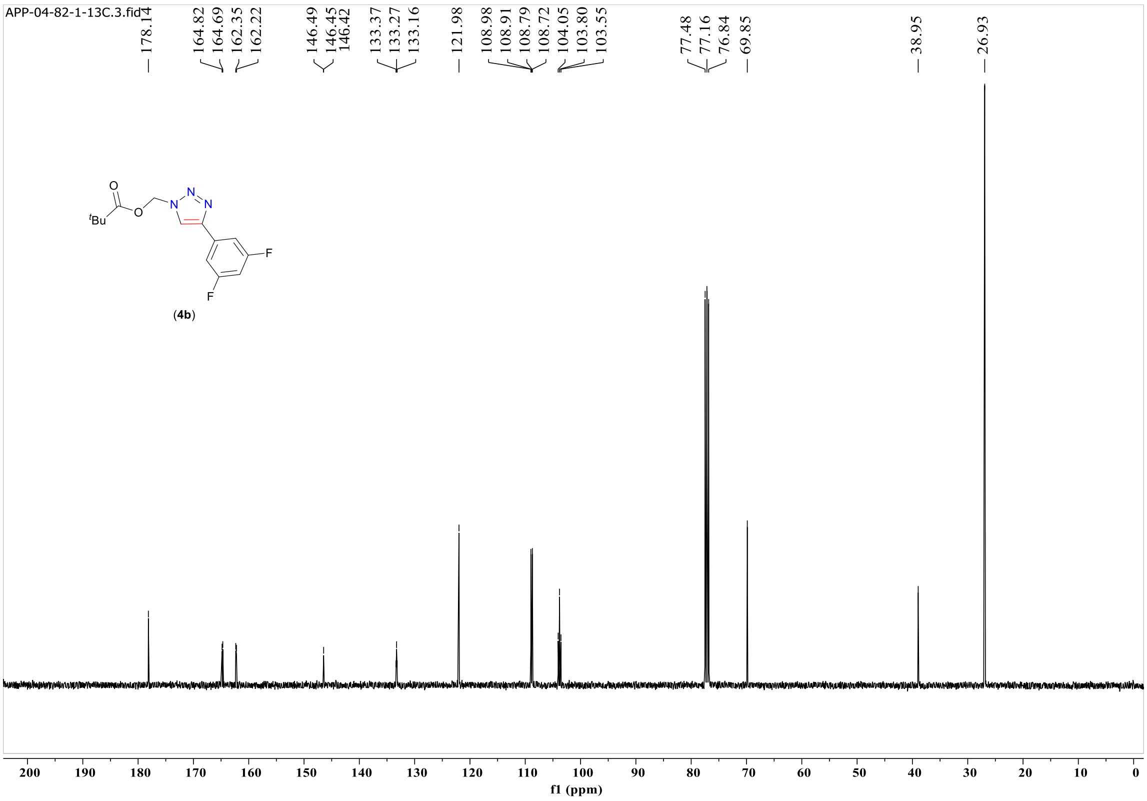

Figure S86. $^{13}C\{^{1}H\}$ NMR of (4-(3,5-difluorophenyl)-1H-1,2,3-triazol-1-yl)methyl pivalate (**4b**) in $CDCl_3$.

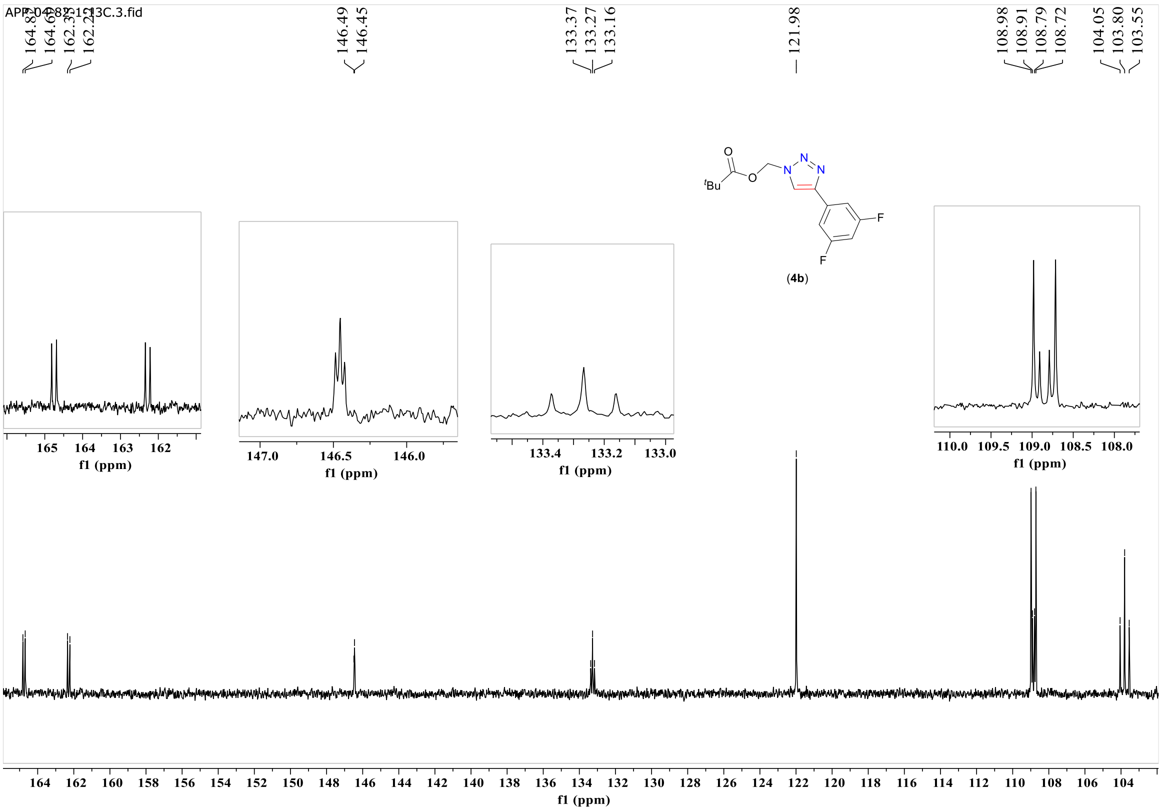

Figure S87. Expanded $^{13}C\{^1H\}$ NMR of (4-(3,5-difluorophenyl)-1H-1,2,3-triazol-1-yl)methyl pivalate (**4b**) in $CDCl_3$.

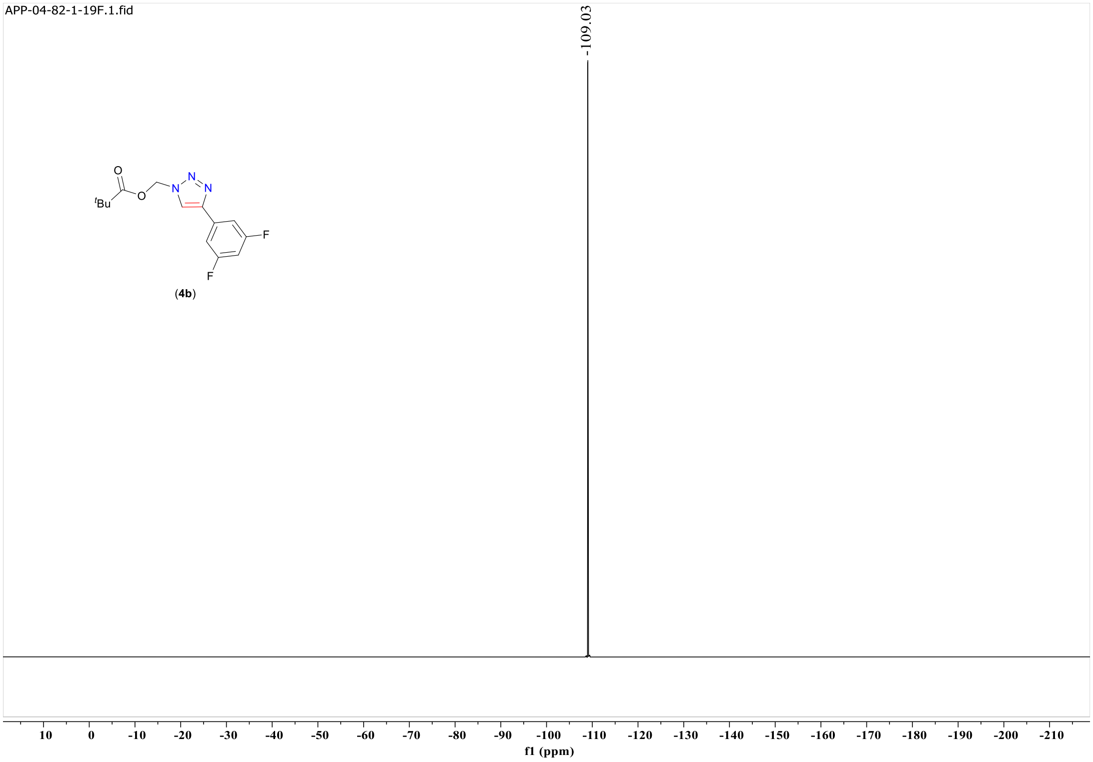

Figure S88. $^{19}F\{^1H\}$ NMR of (4-(3,5-difluorophenyl)-1H-1,2,3-triazol-1-yl)methyl pivalate (**4b**) in $CDCl_3$.

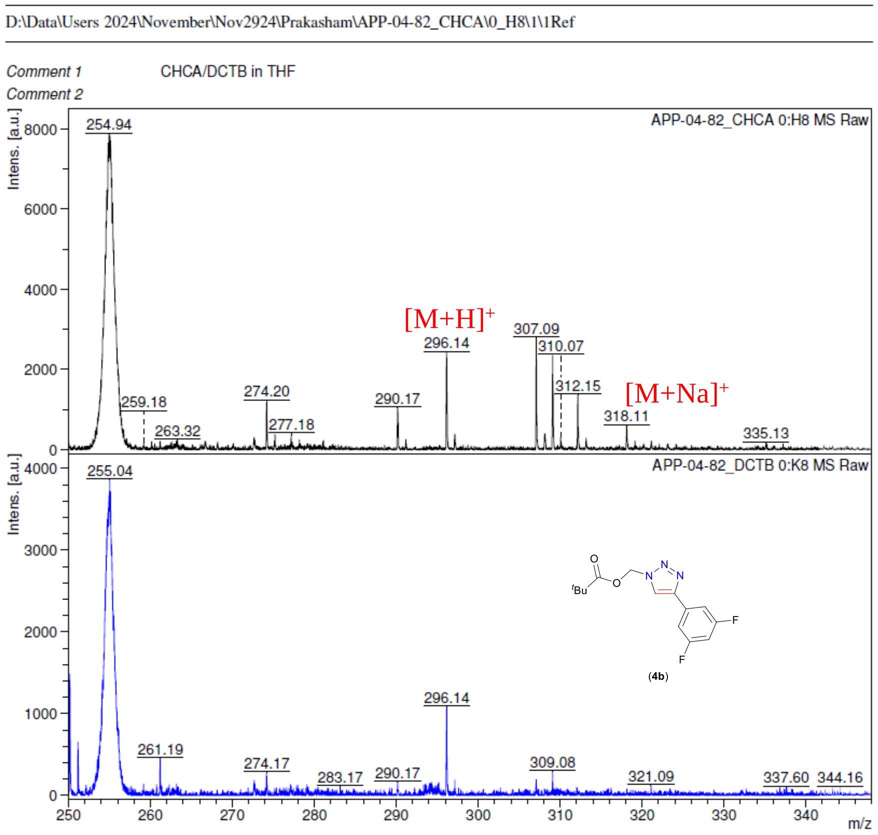

Figure S89. MALDI-ToF-MS spectra of (4-(3,5-difluorophenyl)-1H-1,2,3-triazol-1-yl)methyl pivalate (**4b**).

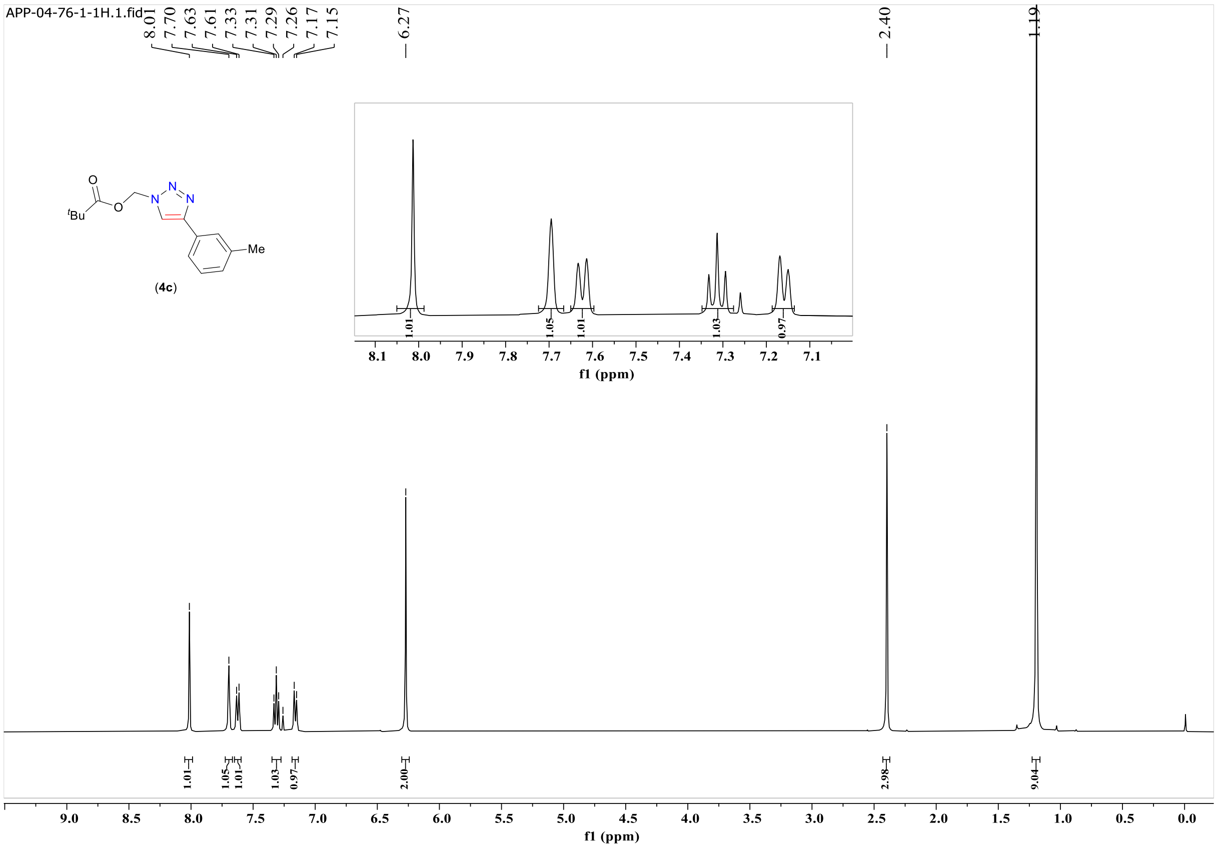

Figure S90. ^{1}H NMR of (4-(*m*-tolyl)-1H-1,2,3-triazol-1-yl)methyl pivalate (**4c**) in $CDCl_3$.

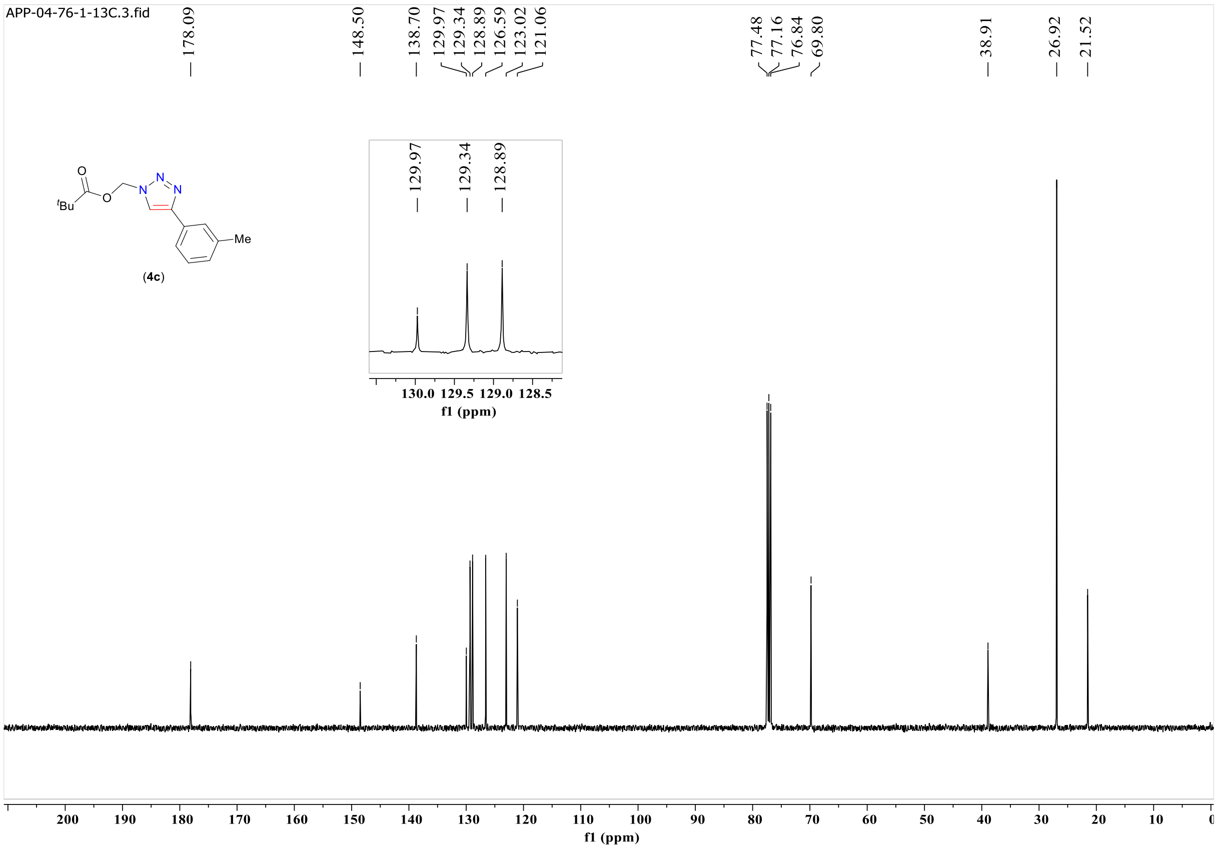

Figure S91. $^{13}C\{^{1}H\}$ NMR of (4-(*m*-tolyl)-1H-1,2,3-triazol-1-yl)methyl pivalate (**4c**) in $CDCl_3$.

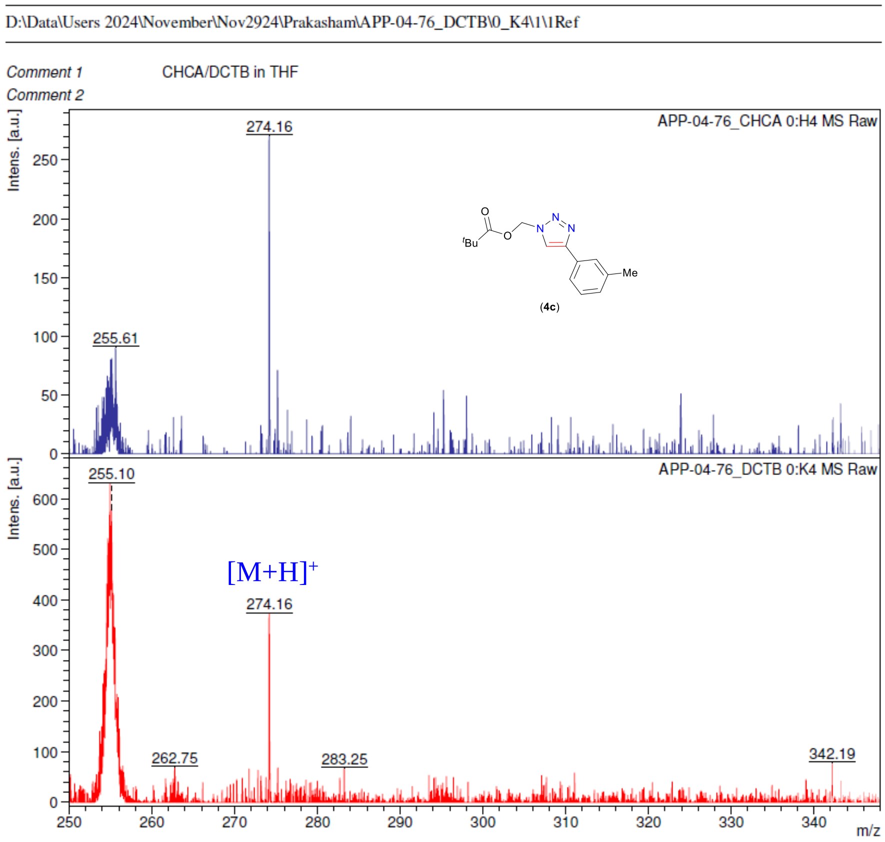

Figure S92. MALDI-ToF-MS spectra of (4-(*m*-tolyl)-1H-1,2,3-triazol-1-yl)methyl pivalate (**4c**).

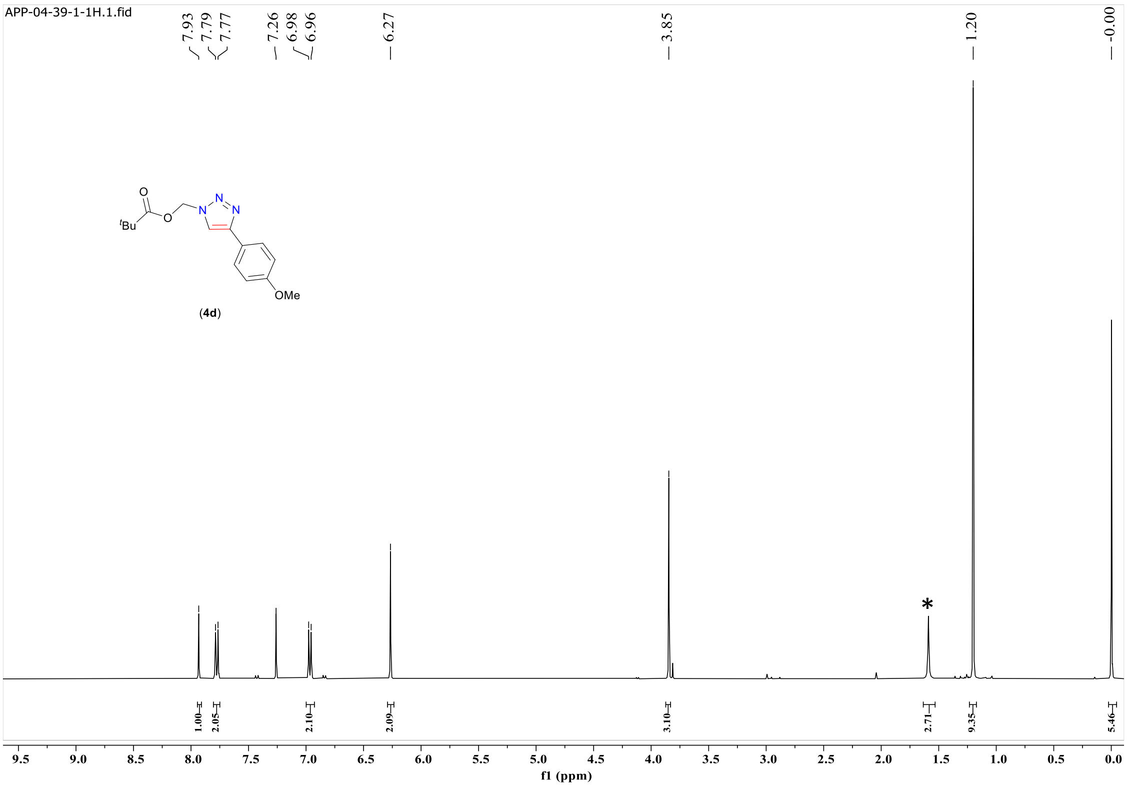

Figure S93. ^{1}H NMR of (4-(4-methoxyphenyl)-1H-1,2,3-triazol-1-yl)methyl pivalate (**4d**) in $CDCl_3$. * H_2O.

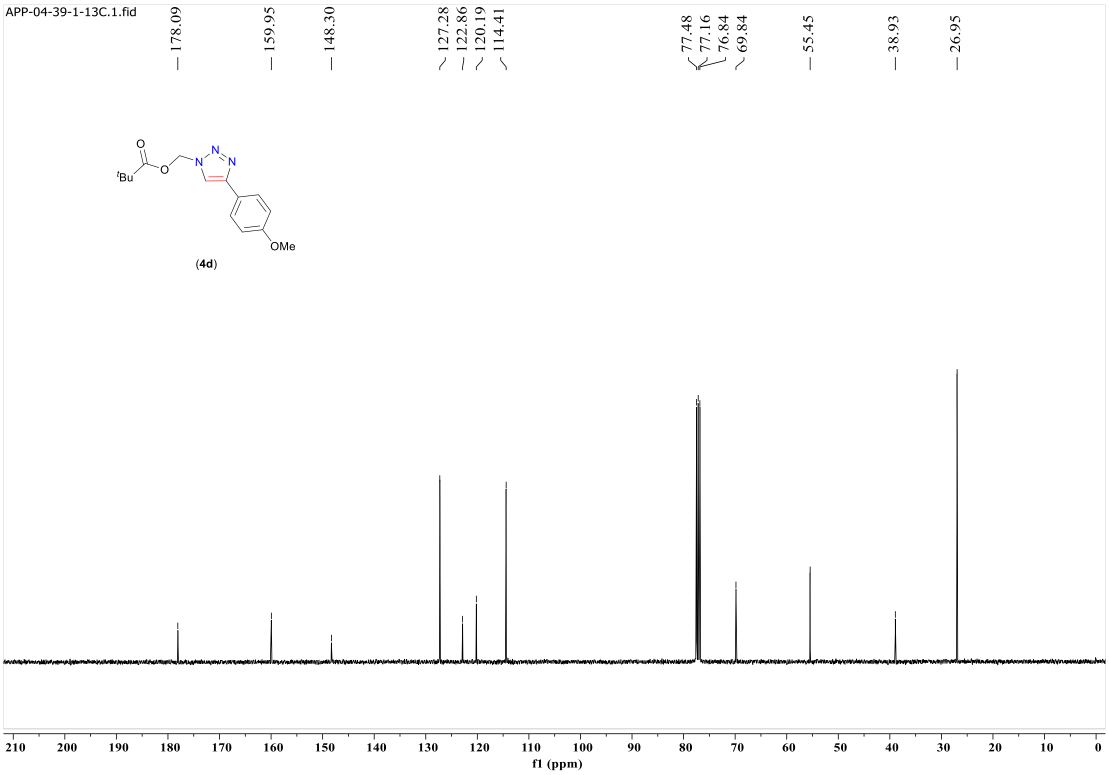

Figure S94. $^{13}C\{^{1}H\}$ NMR of (4-(4-methoxyphenyl)-1H-1,2,3-triazol-1-yl)methyl pivalate (**4d**) in $CDCl_3$.

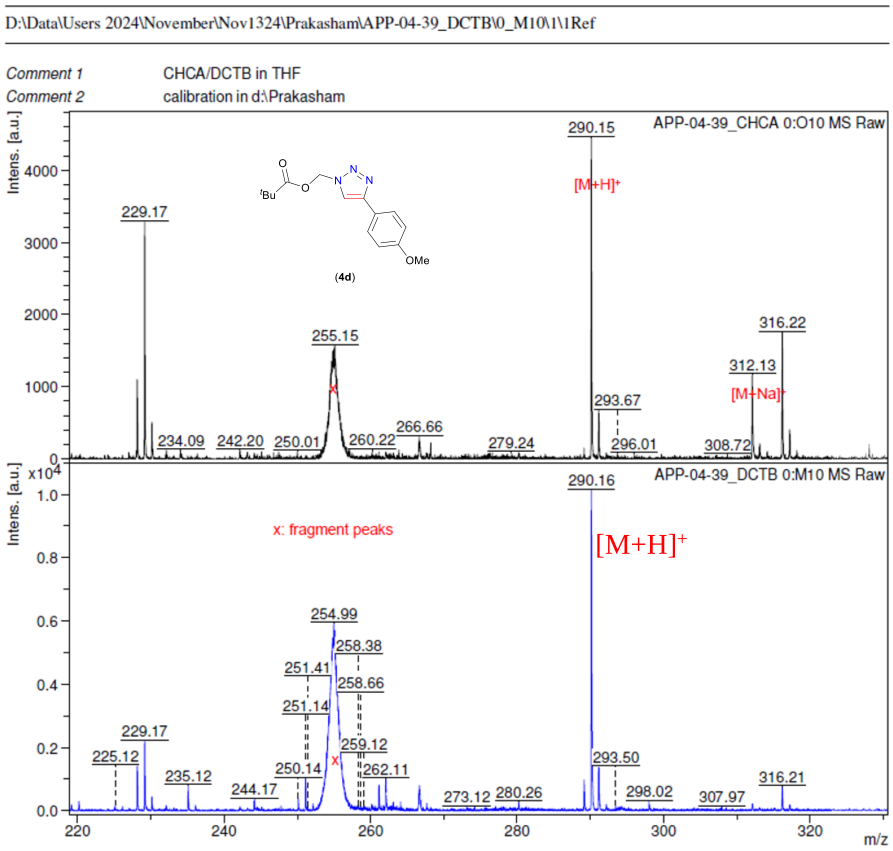

Figure S95. MALDI-ToF-MS spectra of (4-(4-methoxyphenyl)-1H-1,2,3-triazol-1-yl)methyl pivalate (**4d**).

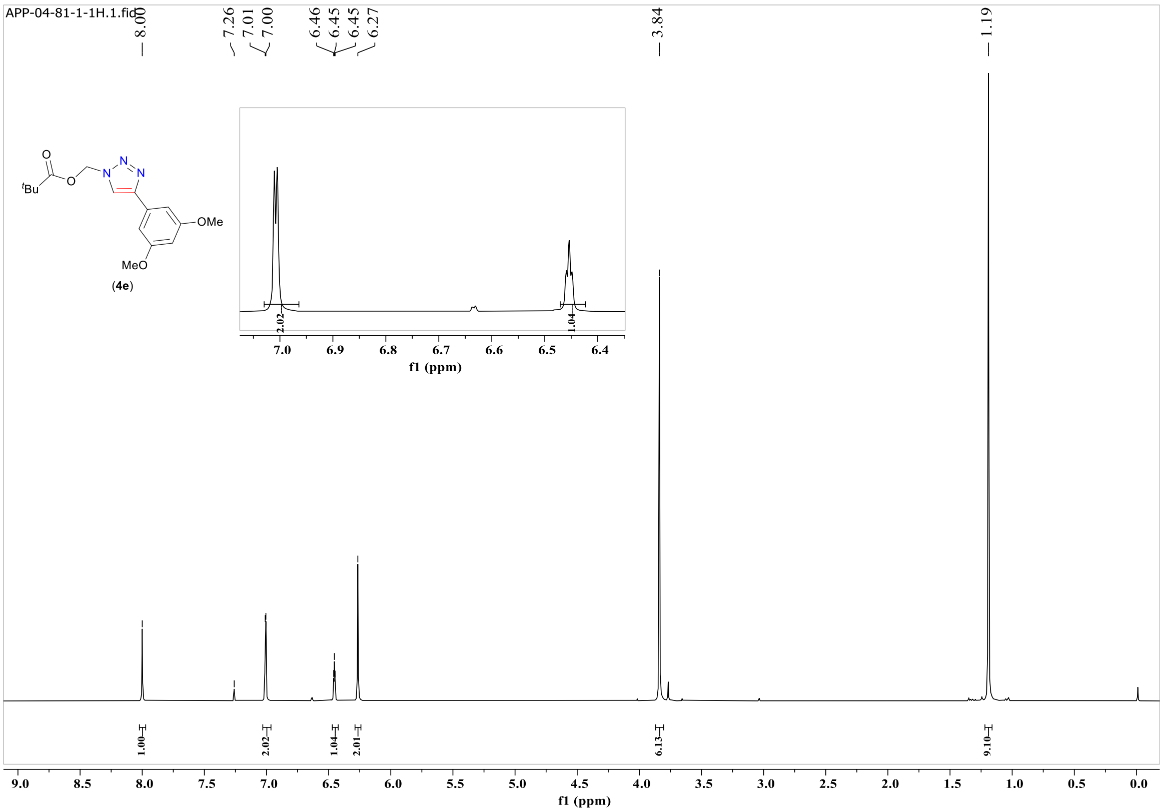

Figure S96. ^{1}H NMR of (4-(3,5-dimethoxyphenyl)-1H-1,2,3-triazol-1-yl)methyl pivalate (**4e**) in $CDCl_3$.

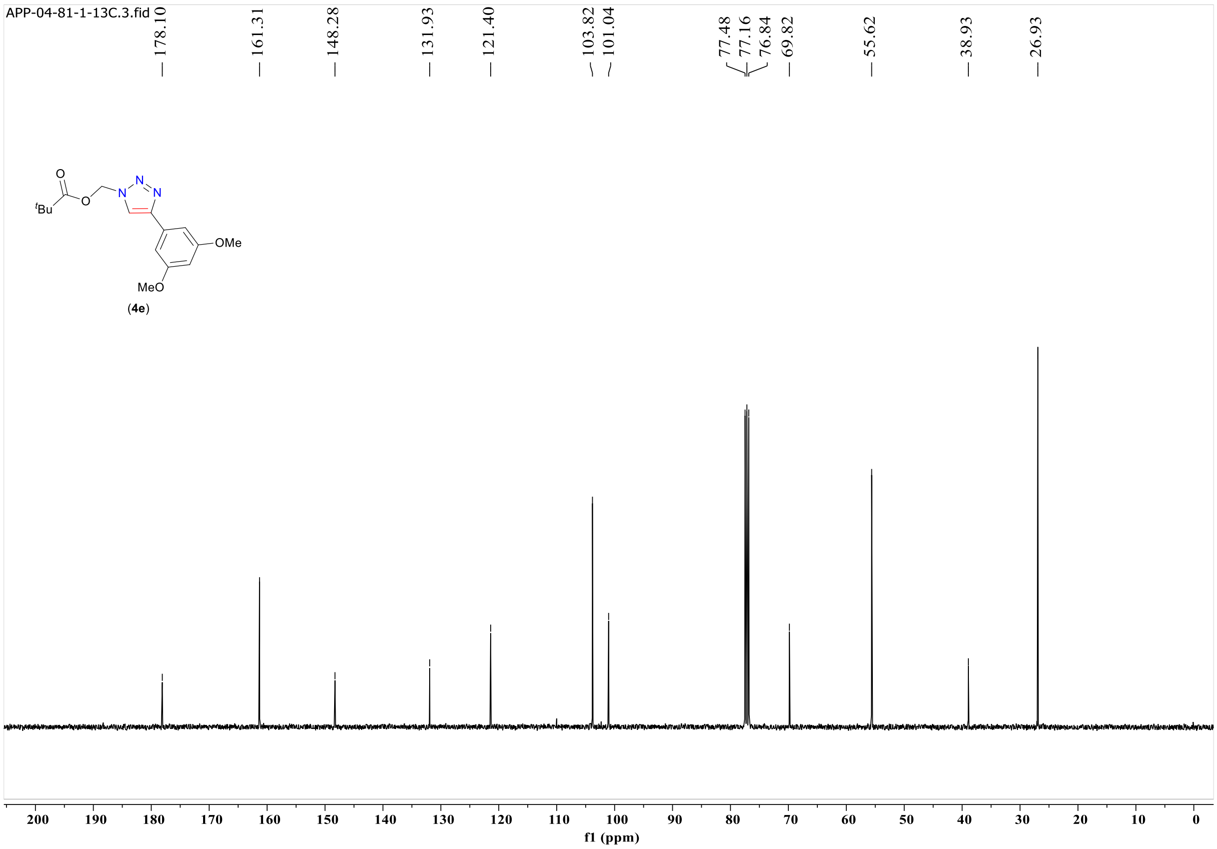

Figure S97. $^{13}C\{^{1}H\}$ NMR of (4-(3,5-dimethoxyphenyl)-1H-1,2,3-triazol-1-yl)methyl pivalate (**4e**) in $CDCl_3$.

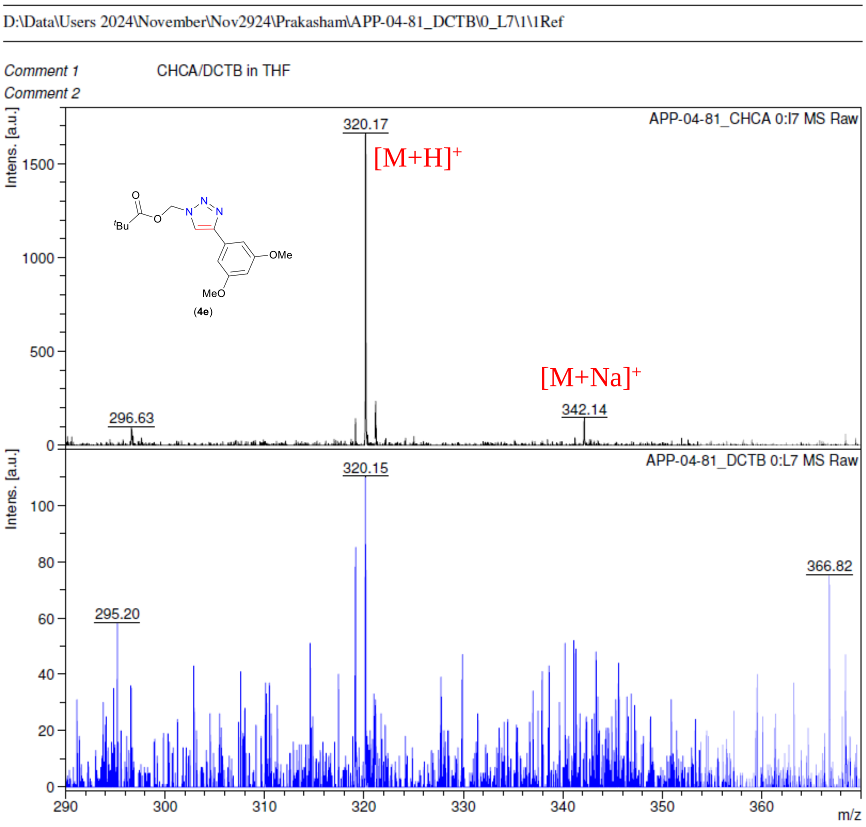

Figure S98. MALDI-ToF-MS spectra of (4-(3,5-dimethoxyphenyl)-1H-1,2,3-triazol-1-yl)methyl pivalate (**4e**).

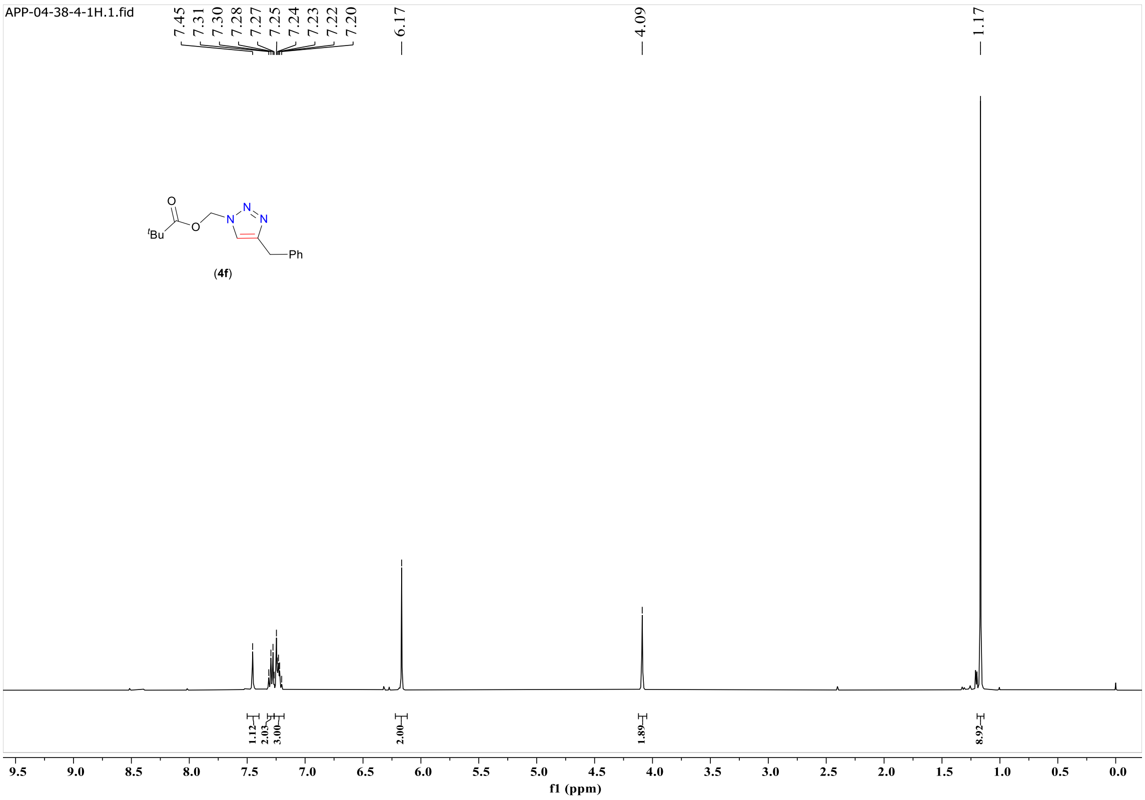

Figure S99. ^{1}H NMR of (4-benzyl-1H-1,2,3-triazol-1-yl)methyl pivalate (**4f**) in $CDCl_3$.

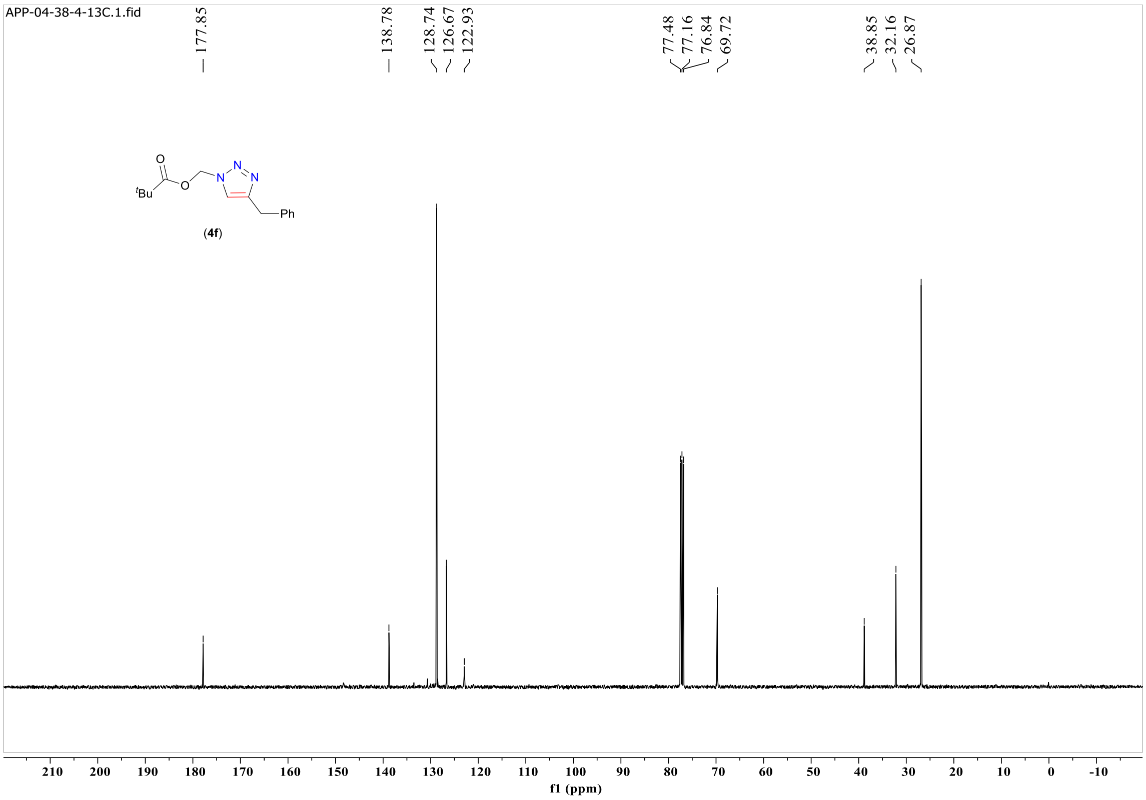

Figure S100. $^{13}C\{^{1}H\}$ NMR of (4-benzyl-1H-1,2,3-triazol-1-yl)methyl pivalate (**4f**) in $CDCl_3$.

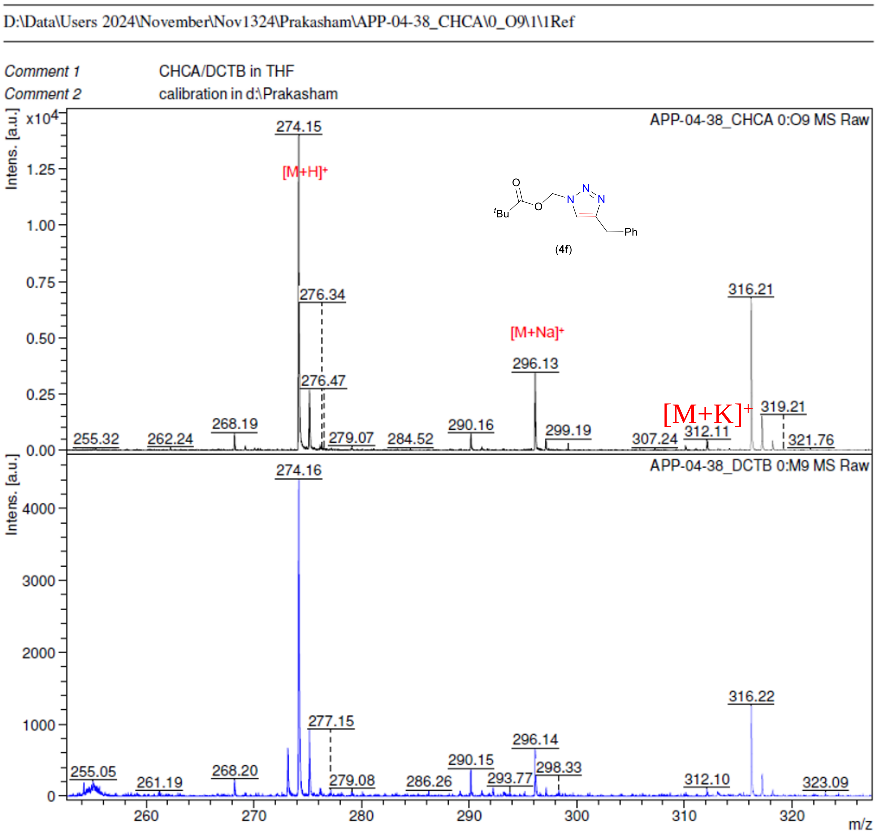

Figure S101. MALDI-ToF-MS spectra of (4-benzyl-1H-1,2,3-triazol-1-yl)methyl pivalate (**4f**).

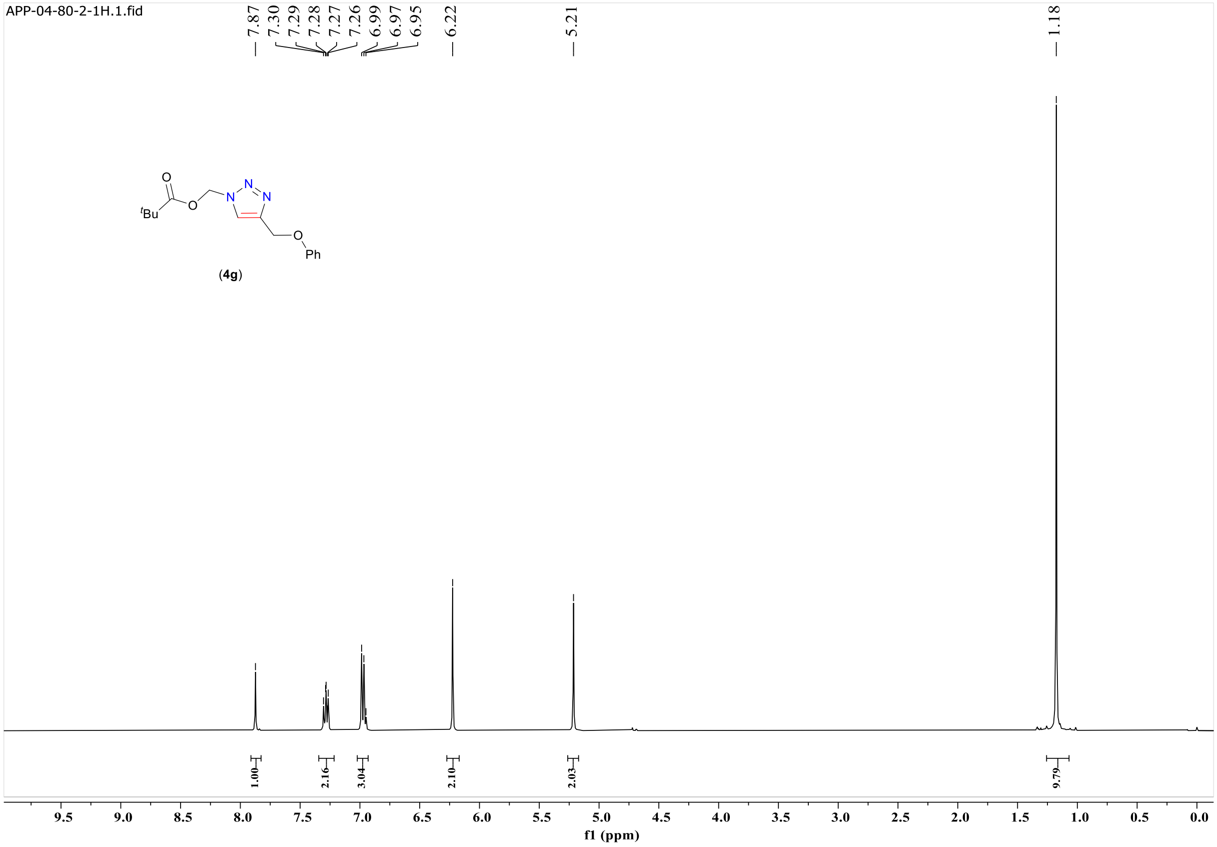

Figure S102. ^{1}H NMR of (4-(phenoxymethyl)-1H-1,2,3-triazol-1-yl)methyl pivalate (**4g**) in $CDCl_3$.

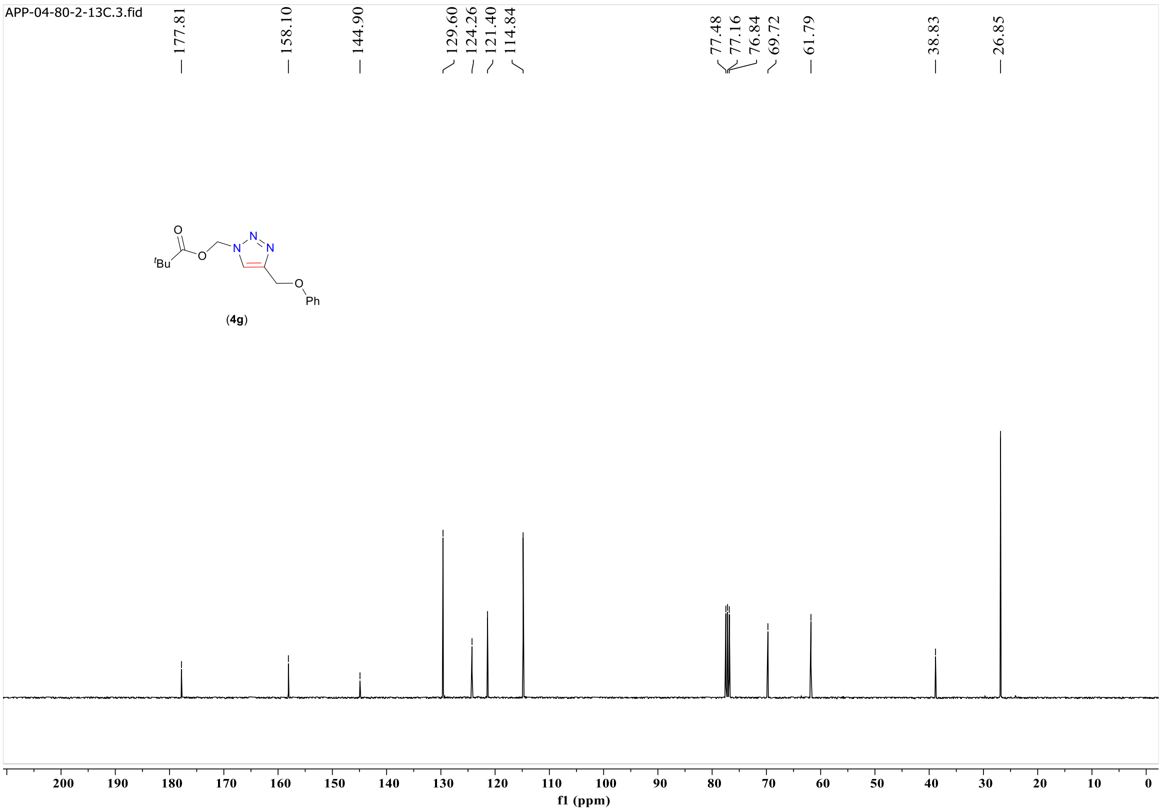

Figure S103. $^{13}C\{^{1}H\}$ NMR of (4-(phenoxymethyl)-1H-1,2,3-triazol-1-yl)methyl pivalate (**4g**) in $CDCl_3$.

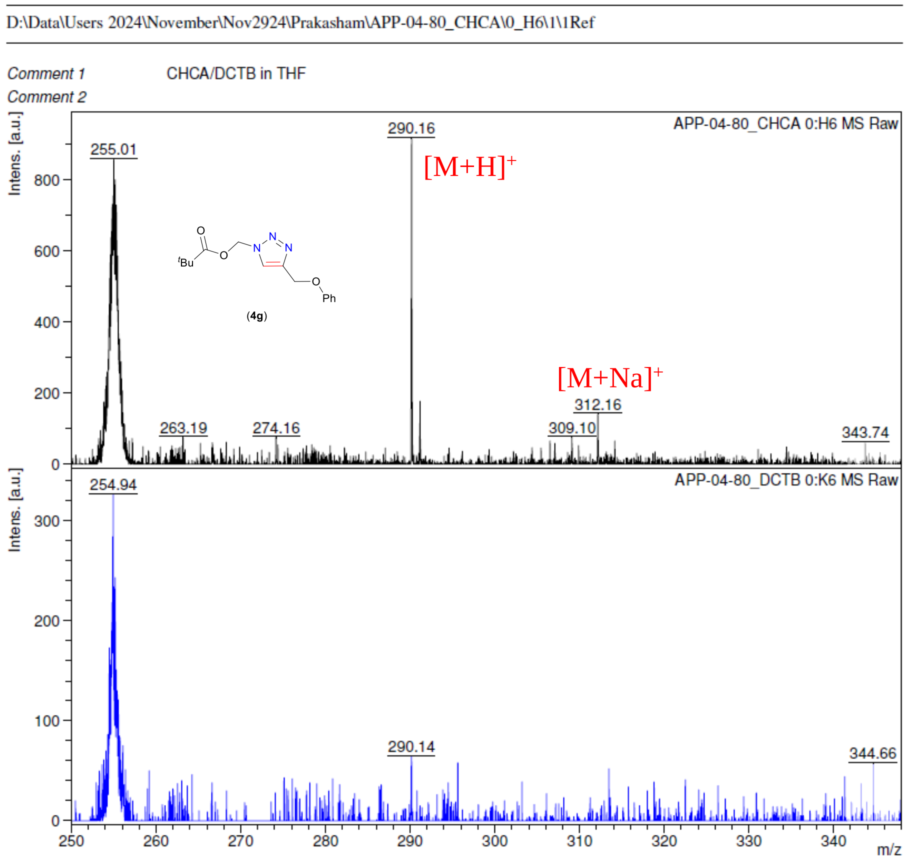

Figure S104. MALDI-ToF-MS spectra of (4-(phenoxymethyl)-1H-1,2,3-triazol-1-yl)methyl pivalate (**4g**).

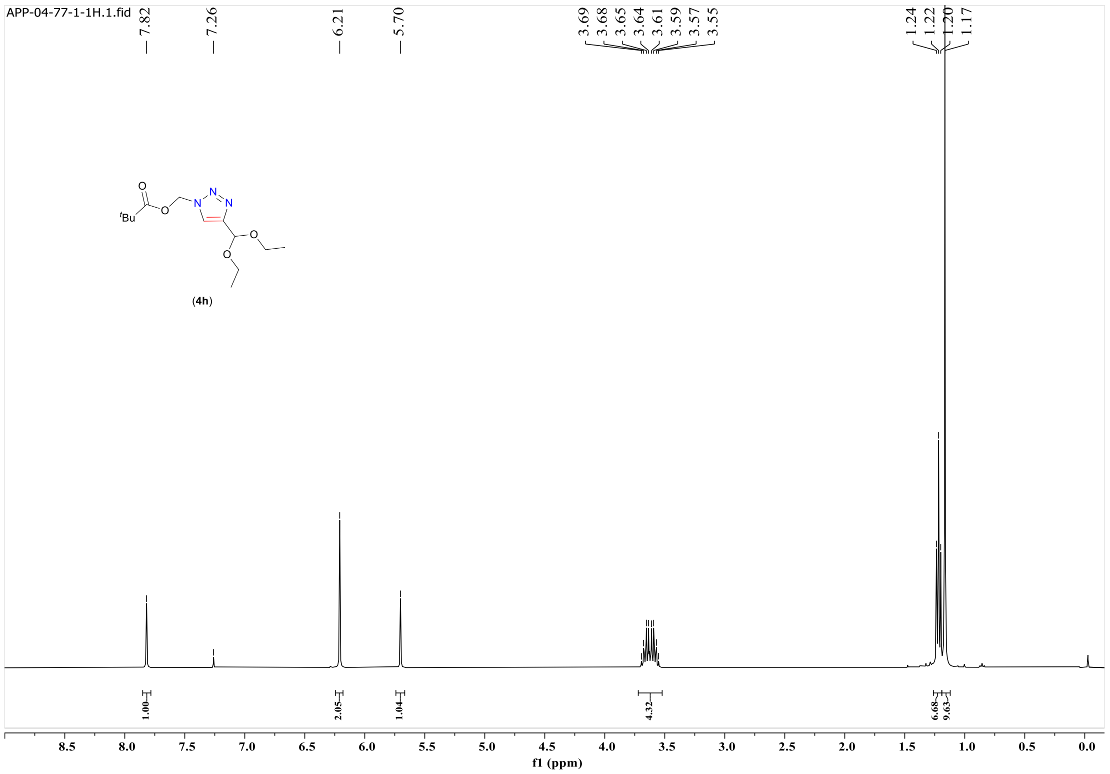

Figure S105. ^{1}H NMR of (4-(diethoxymethyl)-1H-1,2,3-triazol-1-yl)methyl pivalate (**4h**) in $CDCl_3$.

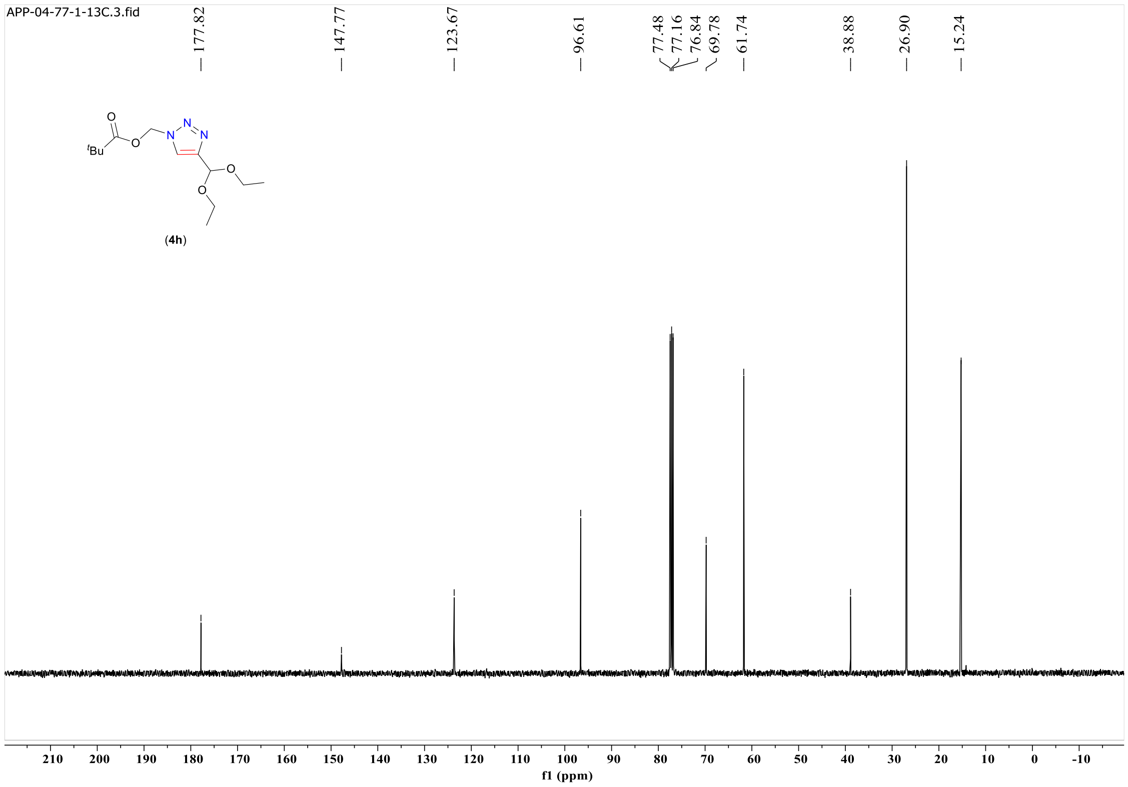

Figure S106. $^{13}C\{^{1}H\}$ NMR of (4-(diethoxymethyl)-1H-1,2,3-triazol-1-yl)methyl pivalate (**4h**) in $CDCl_3$.

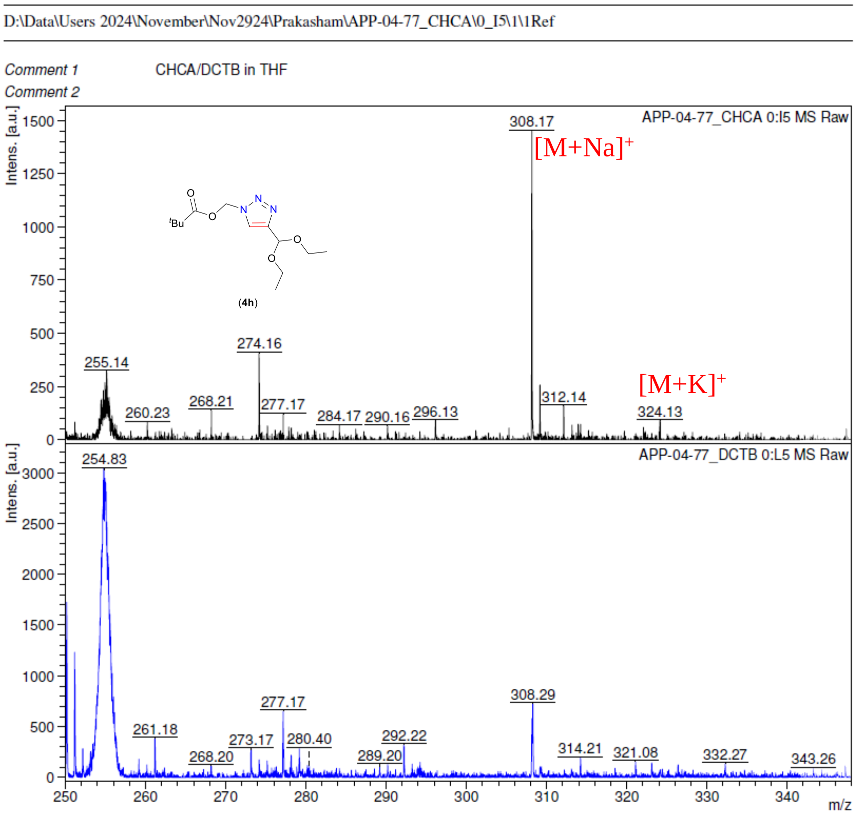

Figure S107. MALDI-ToF-MS spectra of (4-(diethoxymethyl)-1H-1,2,3-triazol-1-yl)methyl pivalate (**4h**).

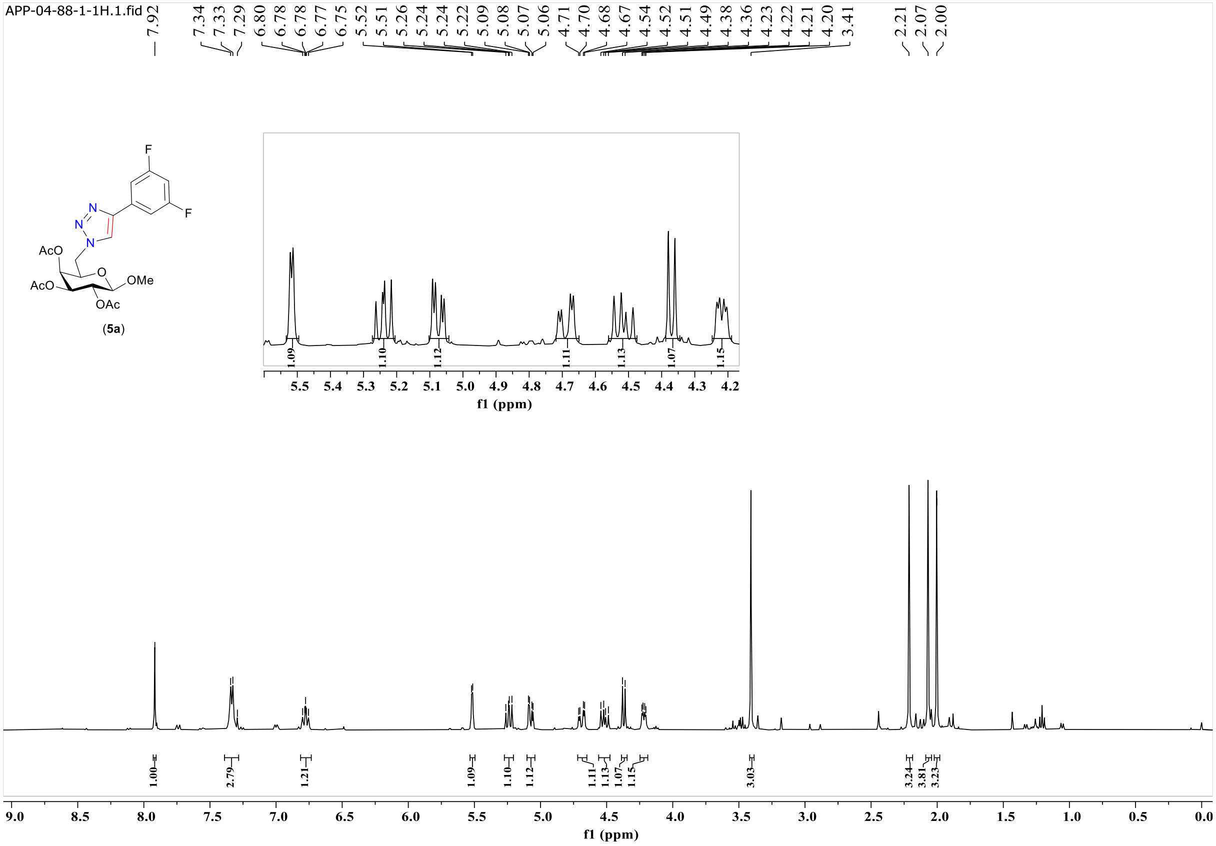

Figure S108. ^{1}H NMR of β-D-galactopyranoside, methyl 6-deoxy-6-(4-(3,5-difluorophenyl)-1H-1,2,3-triazol-1-yl)-, 2,3,4-triacetate (**5a**) in $CDCl_3$.

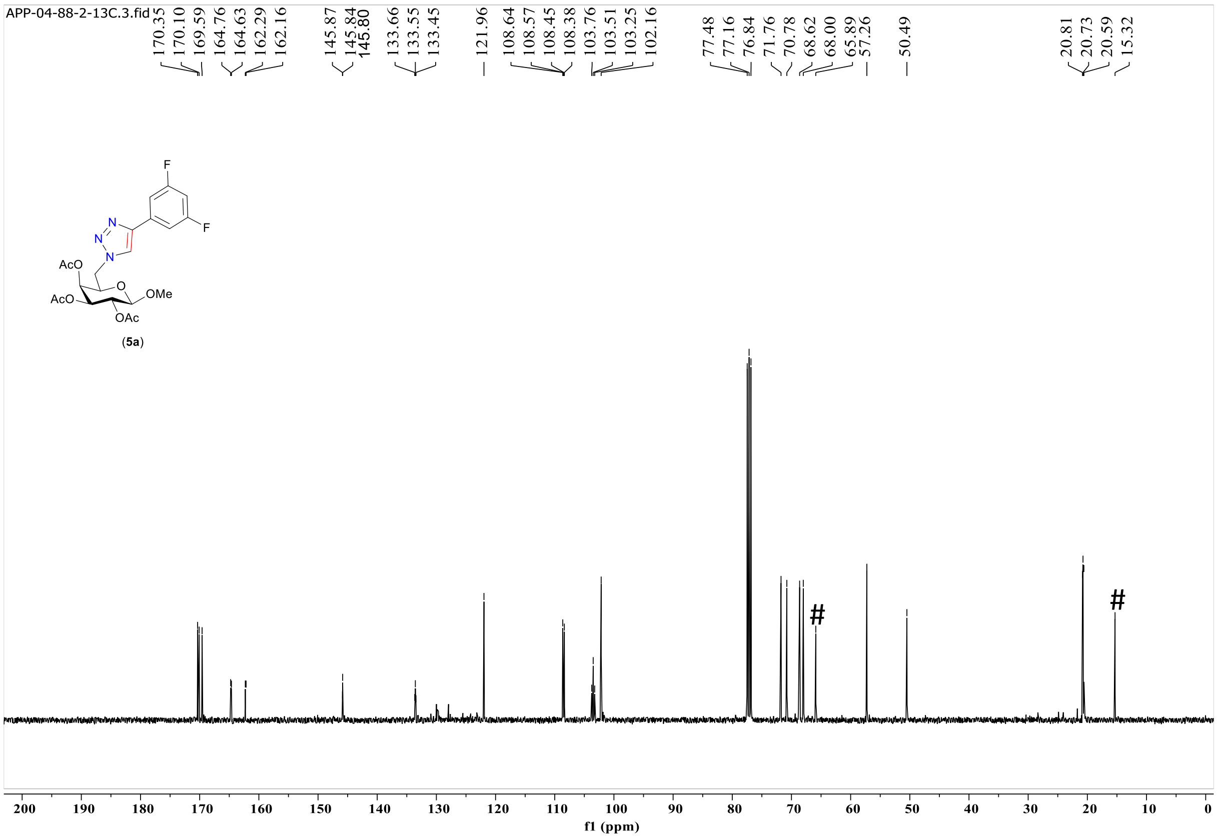

Figure S109. $^{13}C\{^1H\}$ NMR of β-D-galactopyranoside, methyl 6-deoxy-6-(4-(3,5-difluorophenyl)-1H-1,2,3-triazol-1-yl)-, 2,3,4-triacetate (**5a**) in $CDCl_3$. # Et_2O.

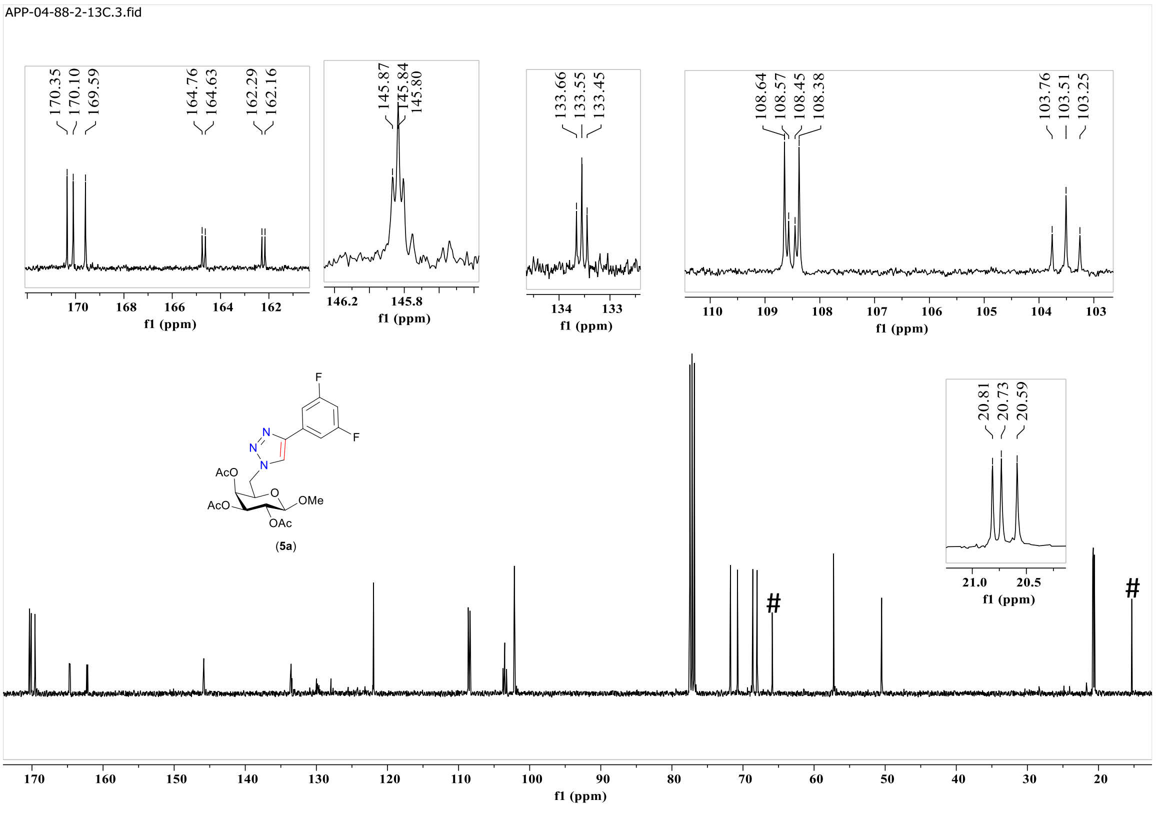

Figure S110. Expanded $^{13}C\{^1H\}$ NMR of β-D-galactopyranoside, methyl 6-deoxy-6-(4-(3,5-difluorophenyl)-1H-1,2,3-triazol-1-yl)-, 2,3,4-triacetate (**5a**) in $CDCl_3$. # Et_2O.

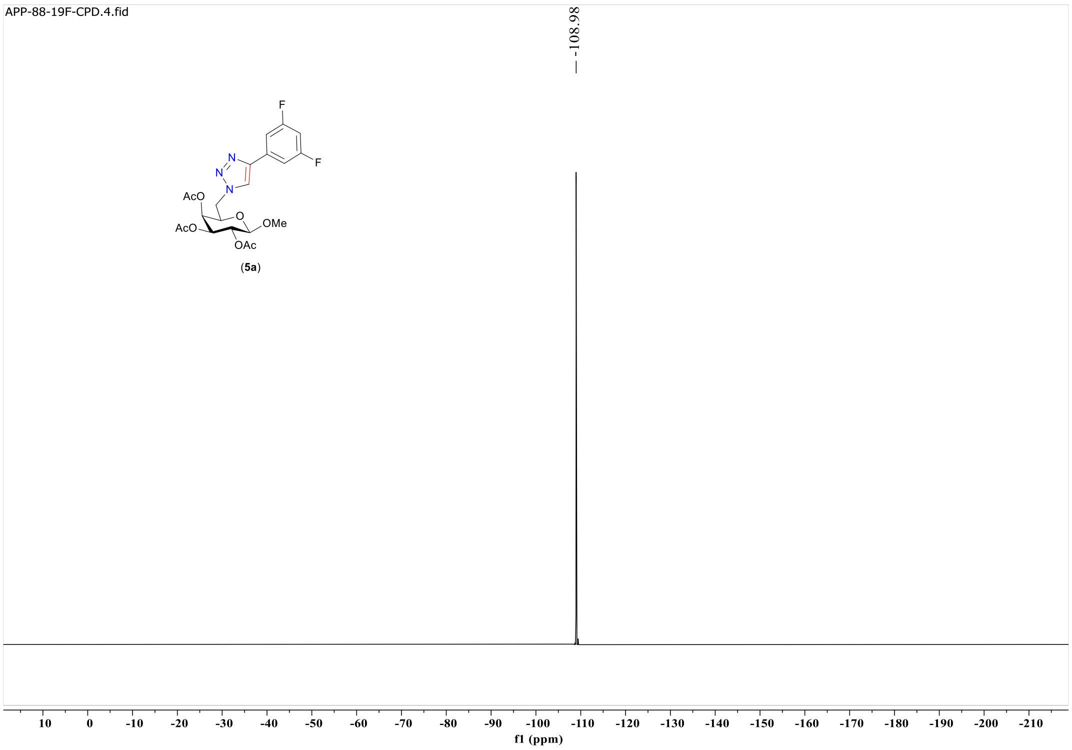

Figure S111. $^{19}F\{^{1}H\}$ NMR of β-D-galactopyranoside, methyl 6-deoxy-6-(4-(3,5-difluorophenyl)-1H-1,2,3-triazol-1-yl)-, 2,3,4-triacetate (**5a**) in $CDCl_3$.

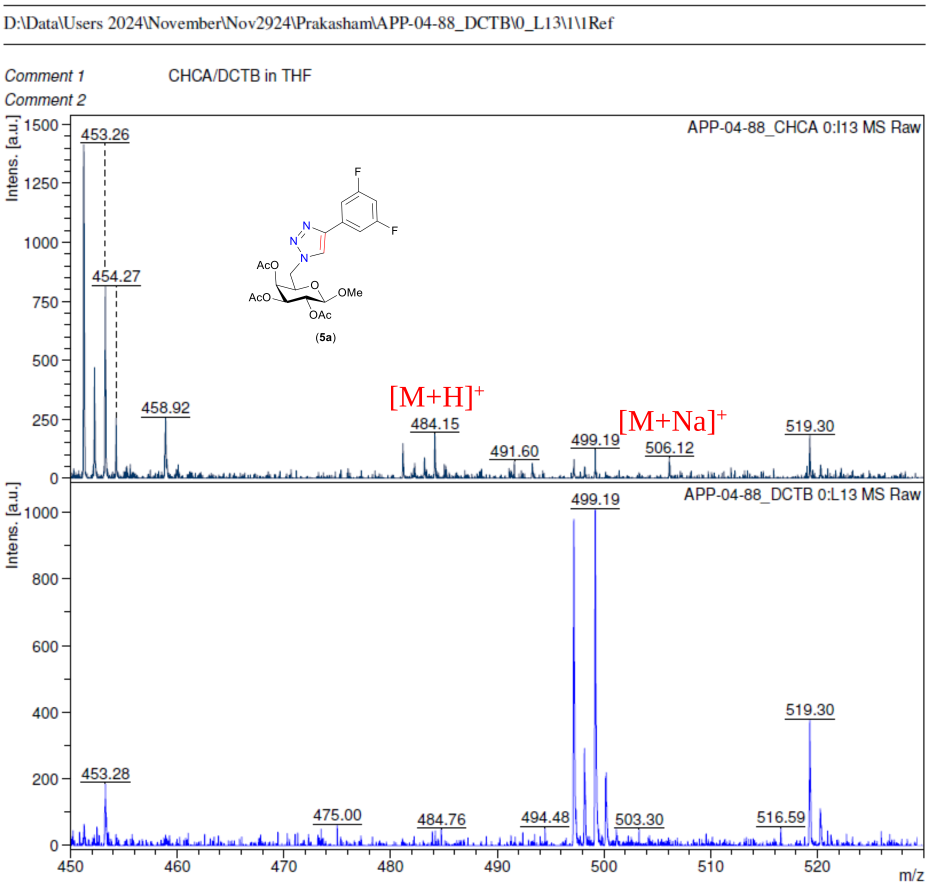

Figure S112. MALDI-ToF-MS spectra of β-D-galactopyranoside, methyl 6-deoxy-6-(4-(3,5-difluorophenyl)-1H-1,2,3-triazol-1-yl)-, 2,3,4-triacetate (**5a**).

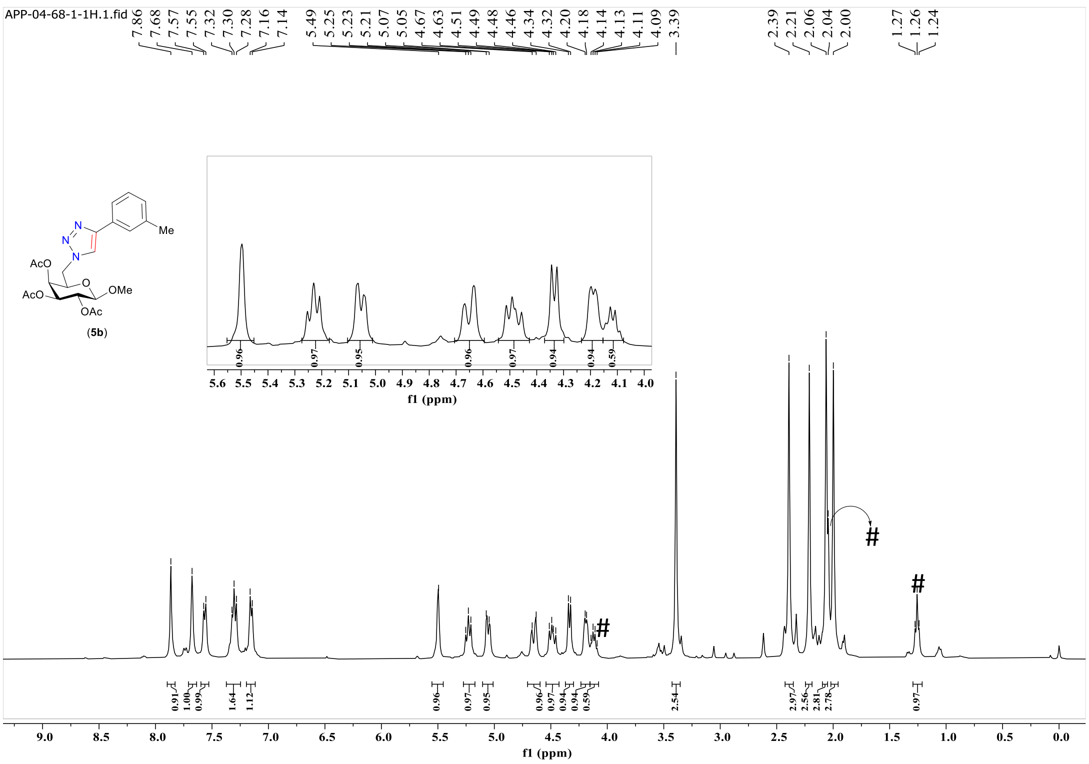

Figure S113. ^{1}H NMR of β-D-Galactopyranoside, methyl 6-deoxy-6-(4-(*m*-tolyl)-1H-1,2,3-triazol-1-yl)-, 2,3,4-triacetate (**5b**) in $CDCl_3$. # EtOAc.

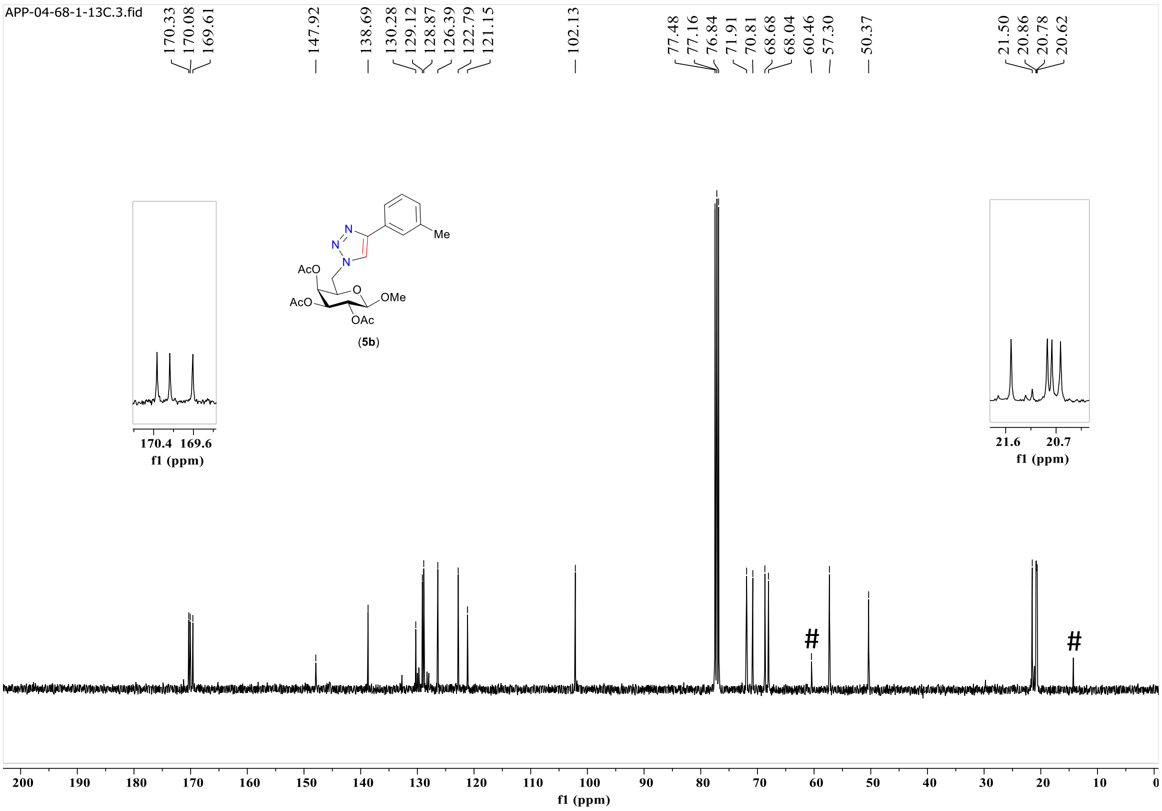

Figure S114. $^{13}C\{^{1}H\}$ NMR of β-D-Galactopyranoside, methyl 6-deoxy-6-(4-(*m*-tolyl)-1H-1,2,3-triazol-1-yl)-, 2,3,4-triacetate (**5b**) in $CDCl_3$. # EtOAc.

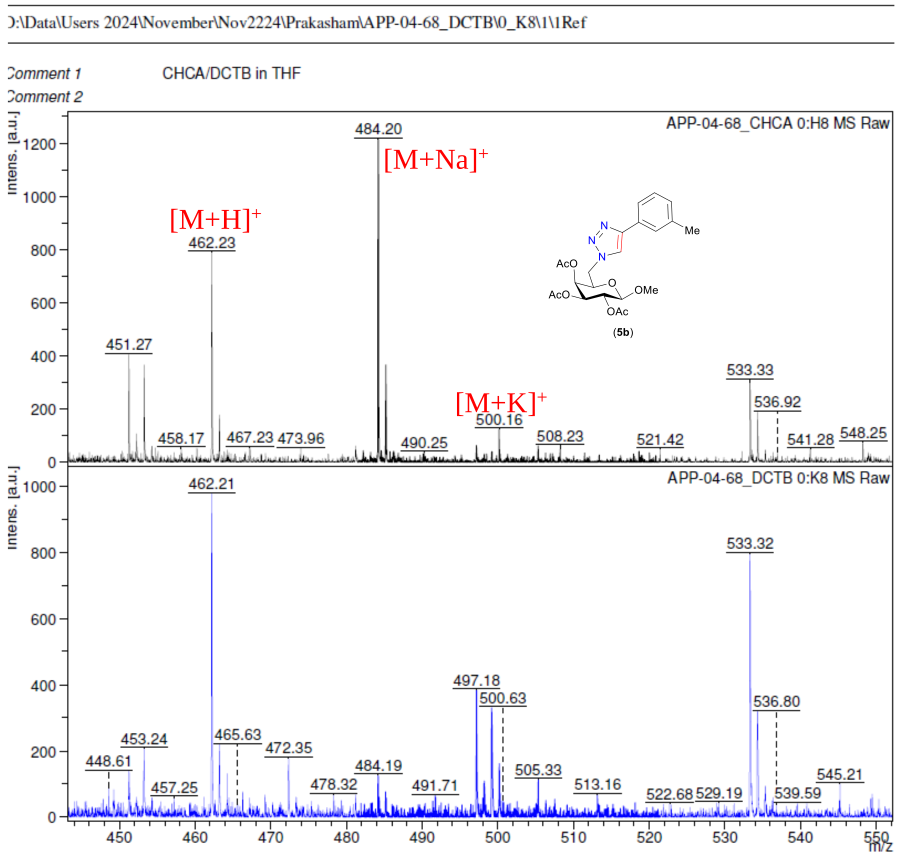

Figure S115. MALDI-ToF-MS spectra of β-D-Galactopyranoside, methyl 6-deoxy-6-(4-(*m*-tolyl)-1H-1,2,3-triazol-1-yl)-, 2,3,4-triacetate (**5b**).

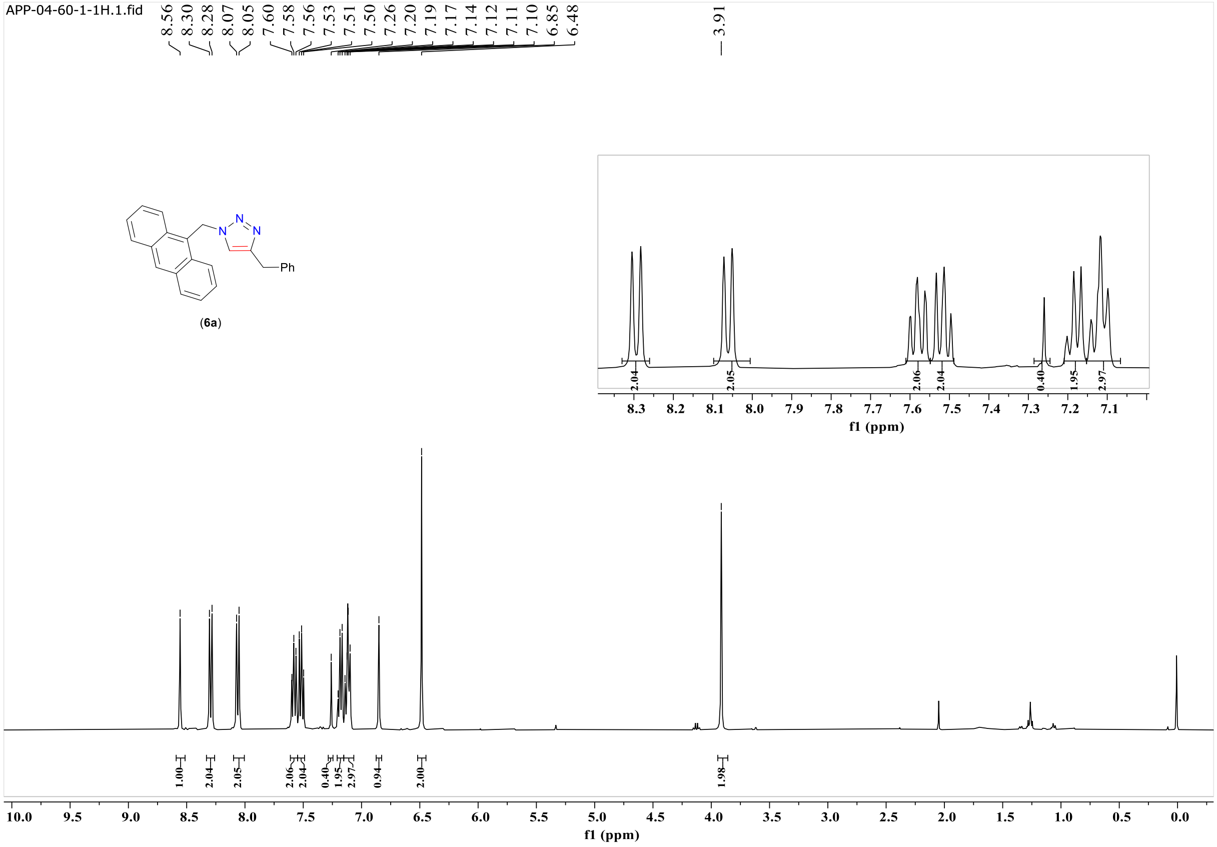

Figure S116. ^{1}H NMR of 1-(anthracen-9-ylmethyl)-4-benzyl-1H-1,2,3-triazole (**6a**) in $CDCl_3$.

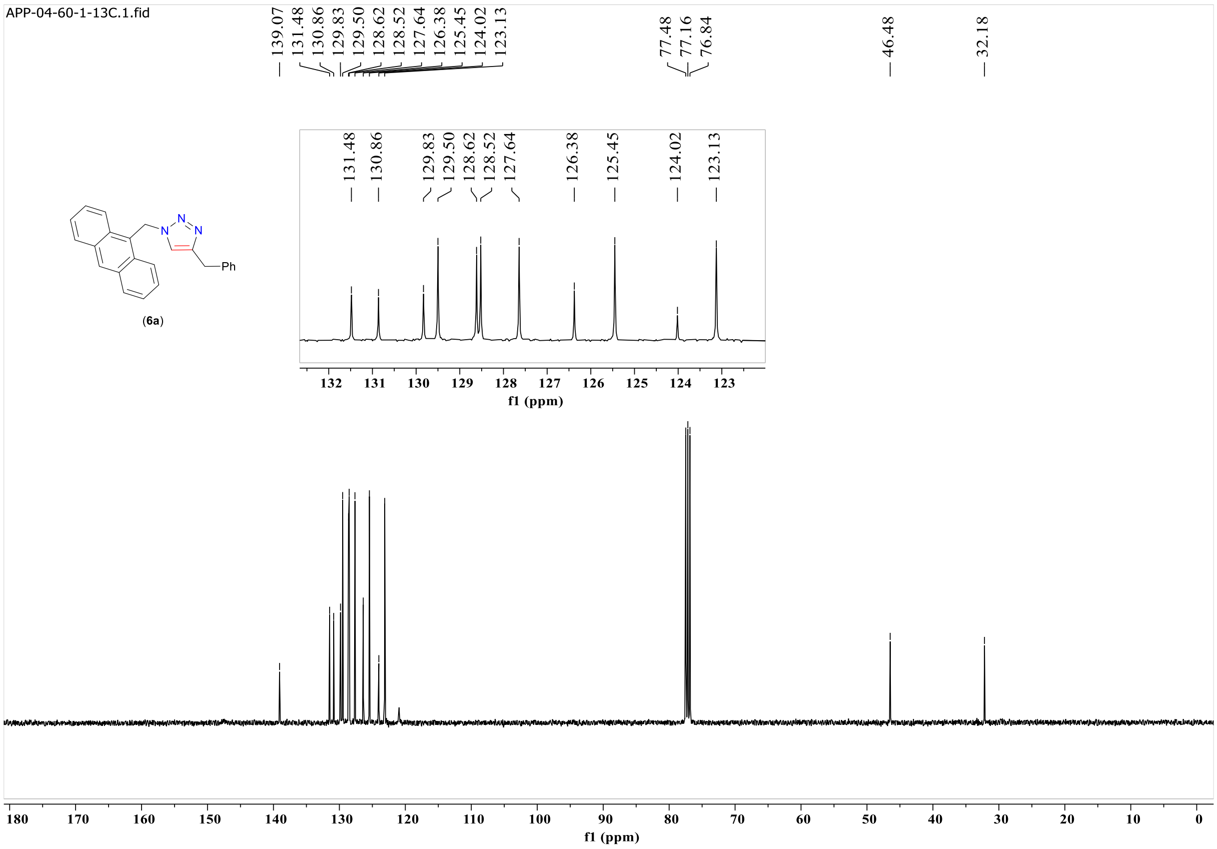

Figure S117. $^{13}C\{^{1}H\}$ NMR of 1-(anthracen-9-ylmethyl)-4-benzyl-1H-1,2,3-triazole (**6a**) in $CDCl_3$.

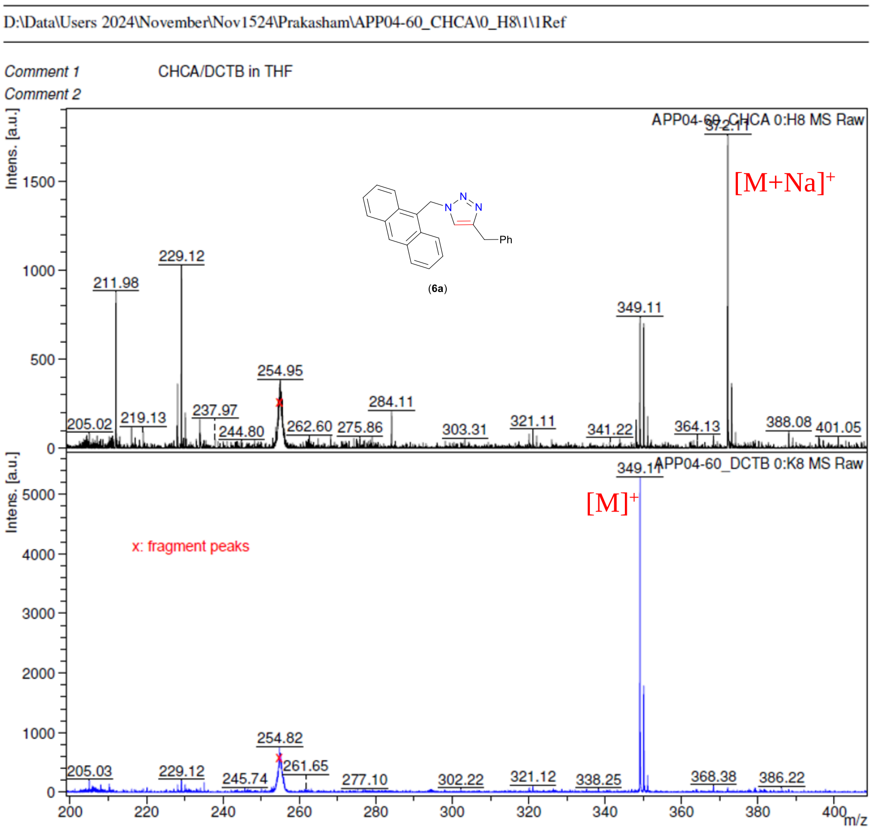

Figure S118. MALDI-ToF-MS spectra of 1-(anthracen-9-ylmethyl)-4-benzyl-1H-1,2,3-triazole (**6a**).

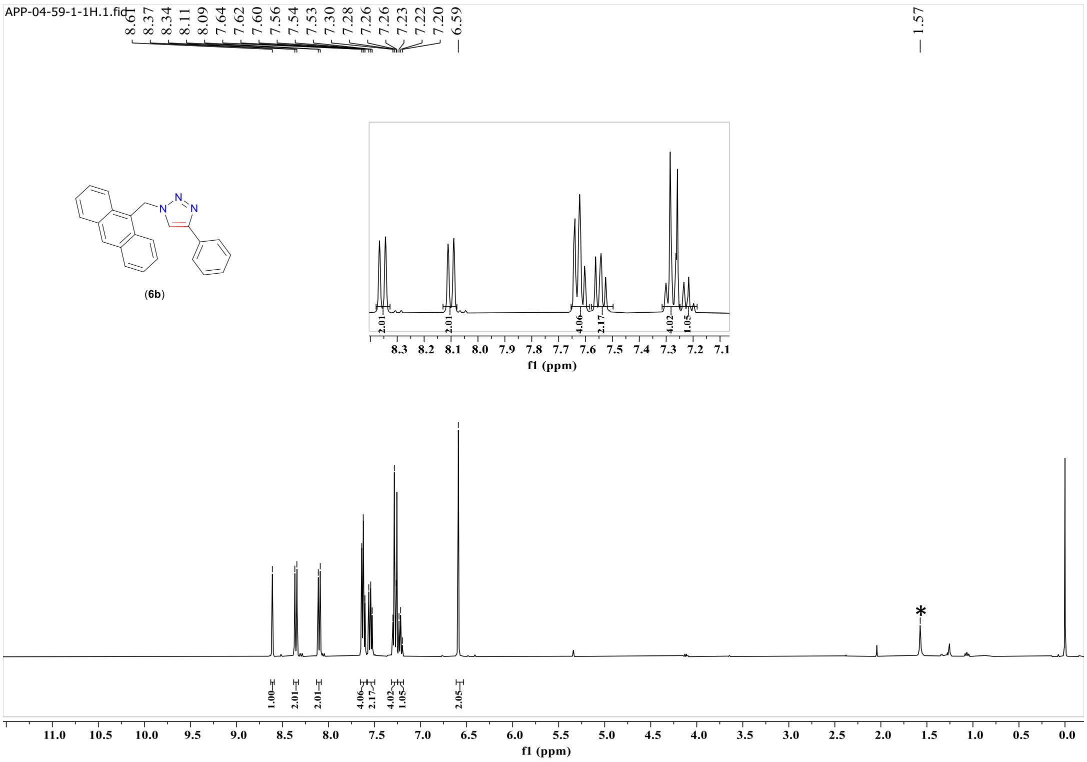

Figure S119. ^{1}H NMR of 1-(anthracen-9-ylmethyl)-4-phenyl-1H-1,2,3-triazole (**6b**) in $CDCl_3$. * H_2O.

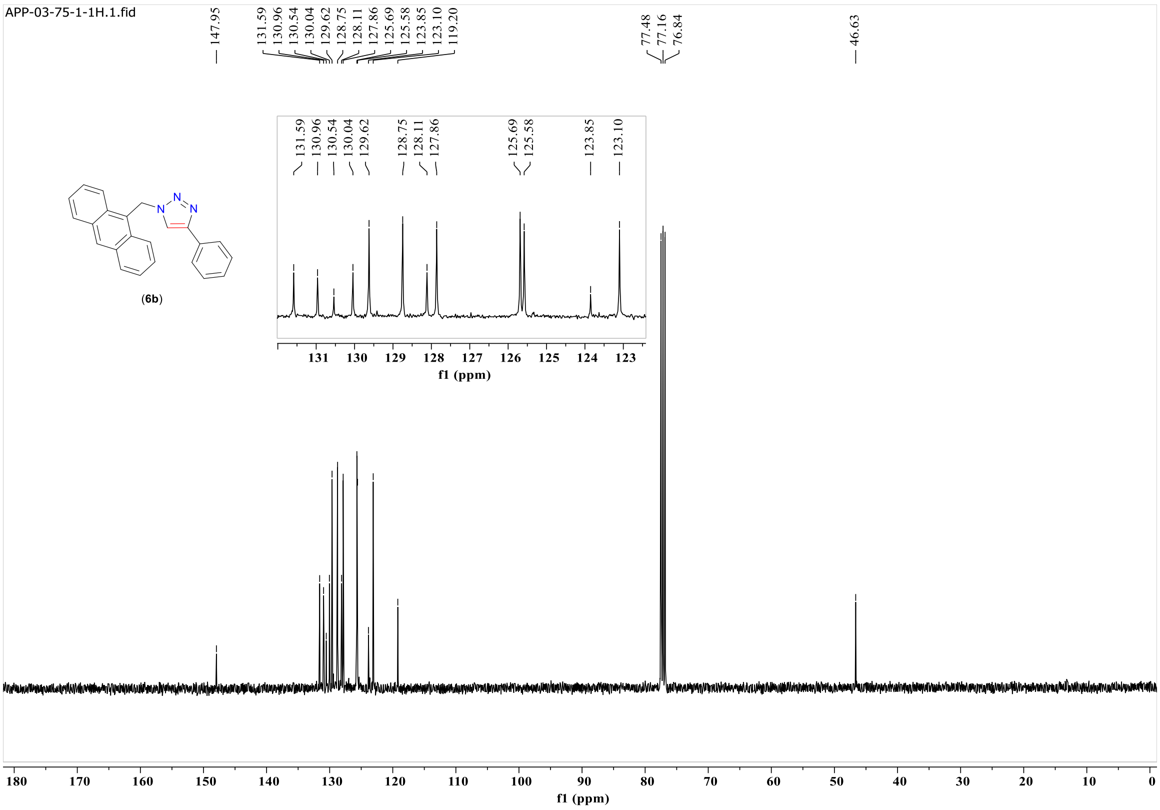

Figure S120. $^{13}C\{^1H\}$ NMR of 1-(anthracen-9-ylmethyl)-4-phenyl-1H-1,2,3-triazole (**6b**) in $CDCl_3$.

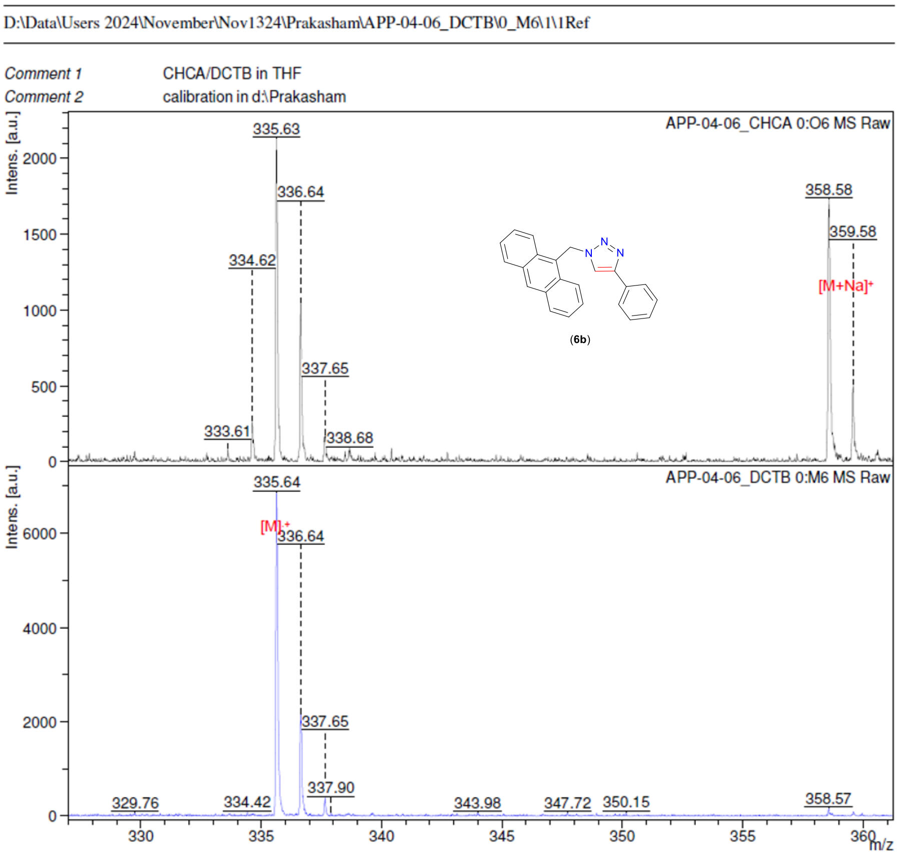

Figure S121. MALDI-ToF-MS spectra of 1-(anthracen-9-ylmethyl)-4-phenyl-1H-1,2,3-triazole (**6b**).

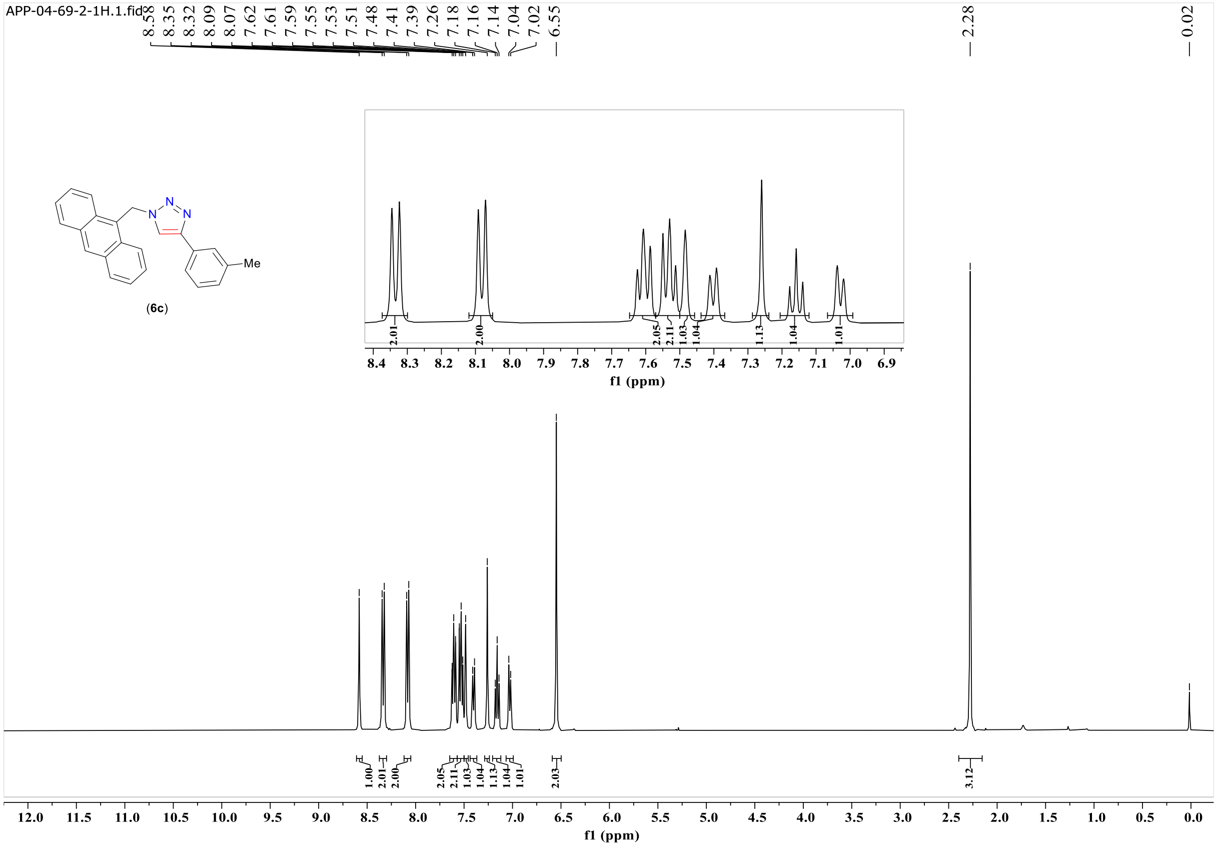

Figure S122. ^{1}H NMR of 1-(anthracen-9-ylmethyl)-4-(*m*-tolyl)-1H-1,2,3-triazole (**6c**) in $CDCl_3$.

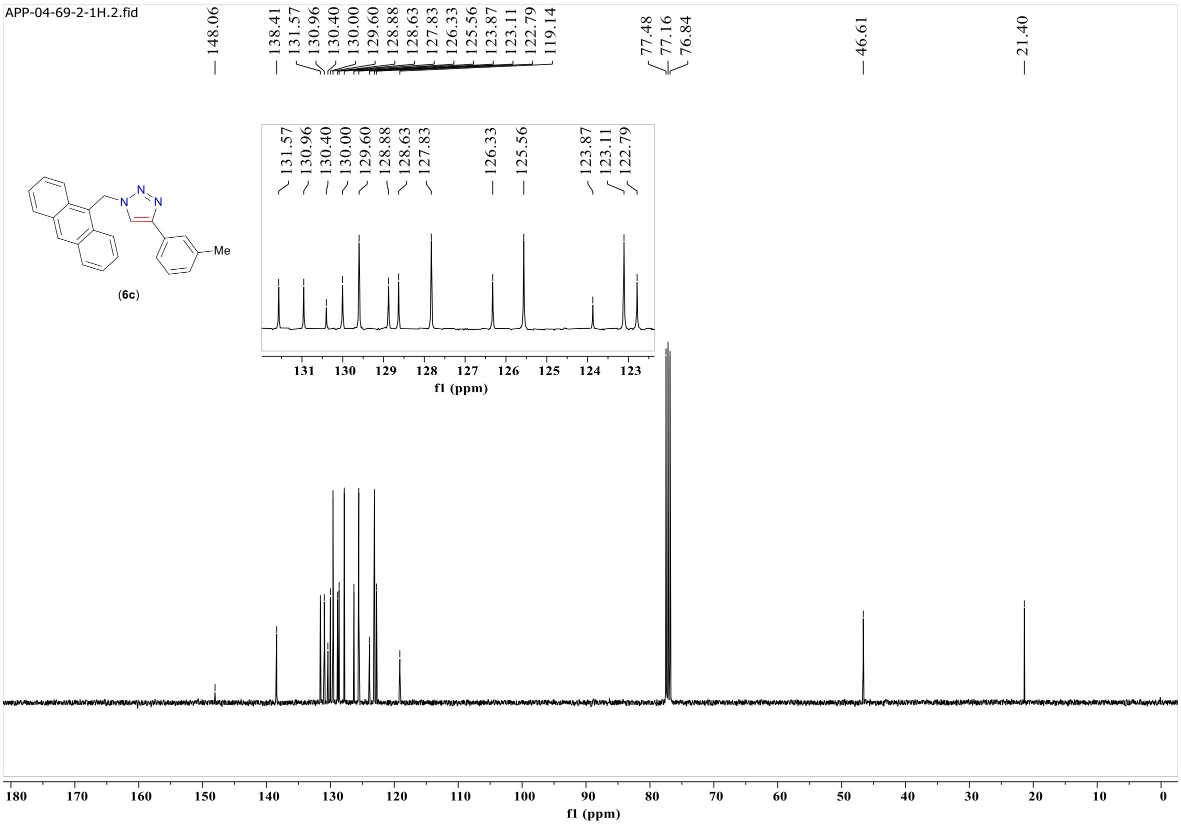

Figure S23. $^{13}C\{^{1}H\}$ NMR of 1-(anthracen-9-ylmethyl)-4-(*m*-tolyl)-1H-1,2,3-triazole (**6c**) in $CDCl_3$.

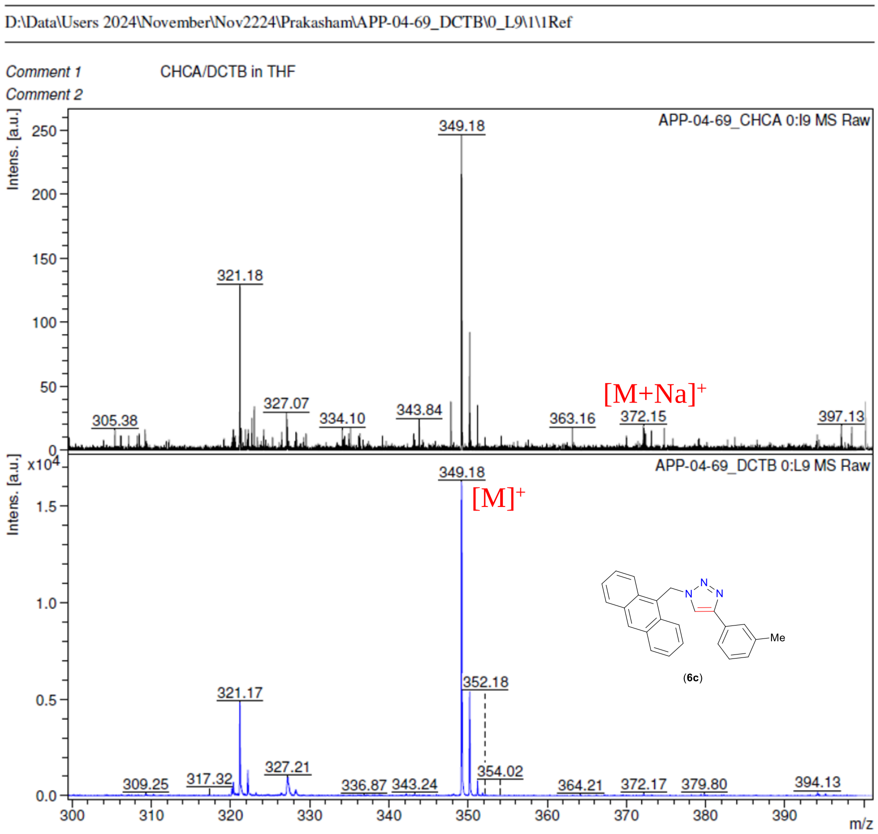

Figure S124. MALDI-ToF-MS spectra of 1-(anthracen-9-ylmethyl)-4-(*m*-tolyl)-1H-1,2,3-triazole (**6c**).

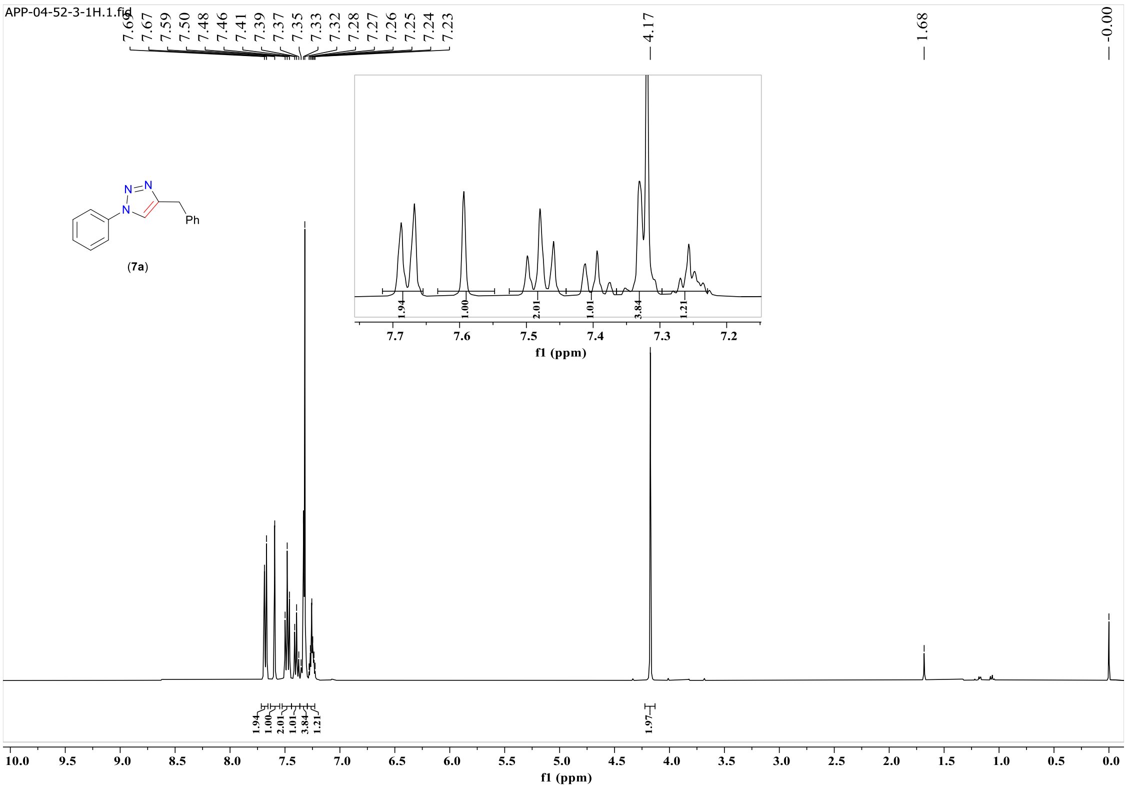

Figure S125. ^{1}H NMR of 4-benzyl-1-phenyl-1H-1,2,3-triazole (**7a**) in $CDCl_3$.

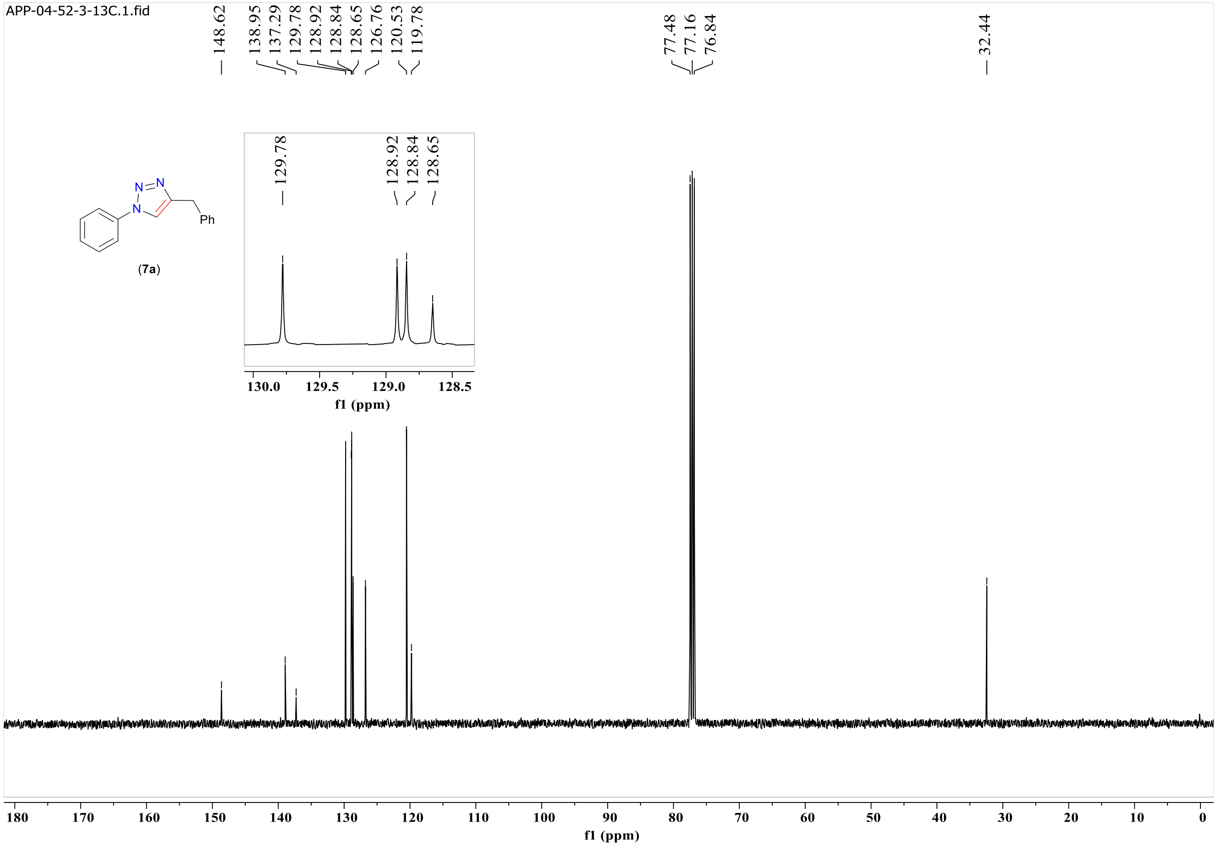

Figure S126. $^{13}C\{^1H\}$ NMR of 4-benzyl-1-phenyl-1H-1,2,3-triazole (**7a**) in $CDCl_3$.

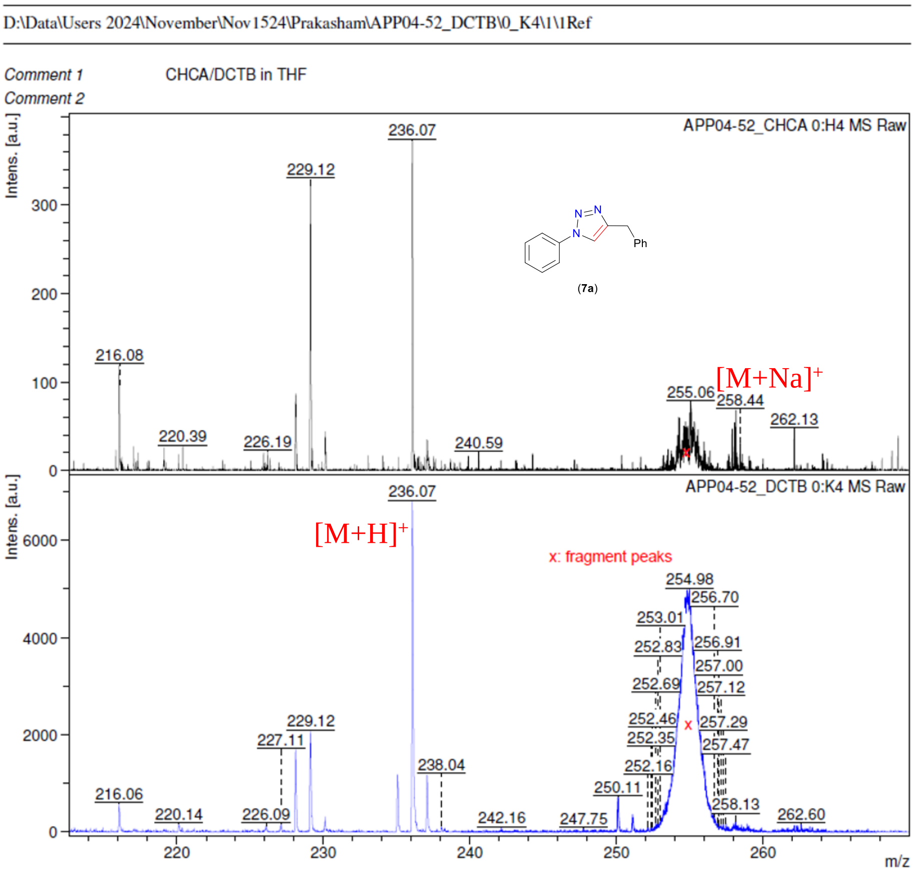

Figure S127. MALDI-ToF-MS spectra of 4-benzyl-1-phenyl-1H-1,2,3-triazole (**7a**).

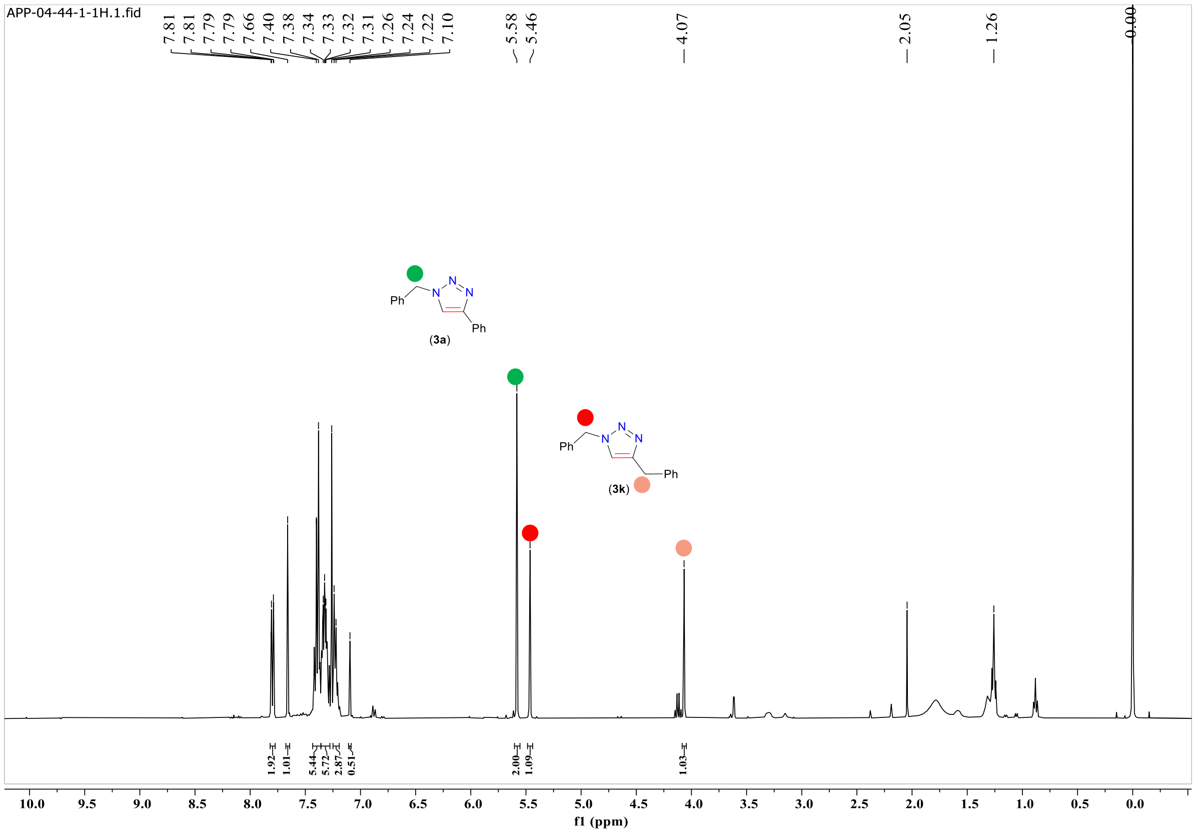

Figure S128. Catalytic activity of the 1 mol % Cu(I)-NHC after the first run (benzyl azide + phenylacetylene) and consecutive second reaction (benzyl azide + benzylacetylene) in one pot.

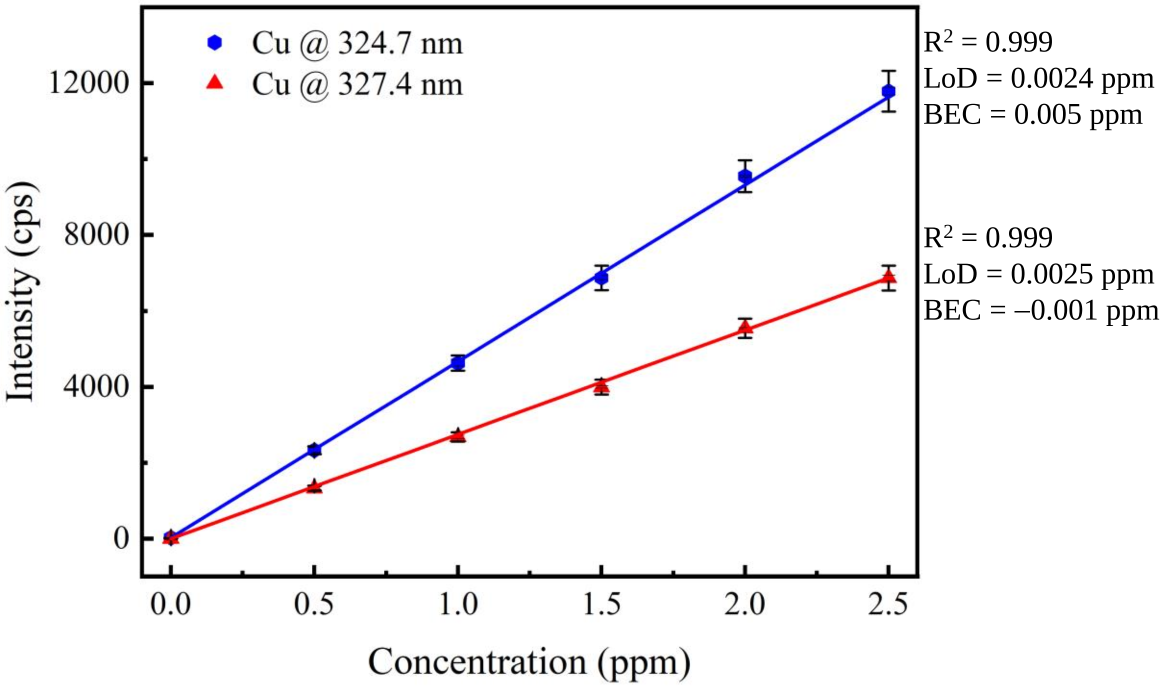

**Cu-concentration (ppm)**	**Cu @ 324.7 nm (cps)**	**Cu @327.4 nm (cps)**	**Observed (ppm)**
0	23.05	-2.77	0
0.5	2325.24	1327.82	0.489
1.0	4622.37	2675.49	0.981
1.5	6871.33	3991.93	1.461
2.0	9547.21	5541.62	2.029
2.5	11787.07	6865.40	2.510
Sample 1	66.72	22.58	0.009
Sample 2	27.87	-0.29	0.001
Sample 3	41.84	8.45	0.004

Figure S129. Calibration curves for the copper encapsulation efficiency measurements by ICP-OES.

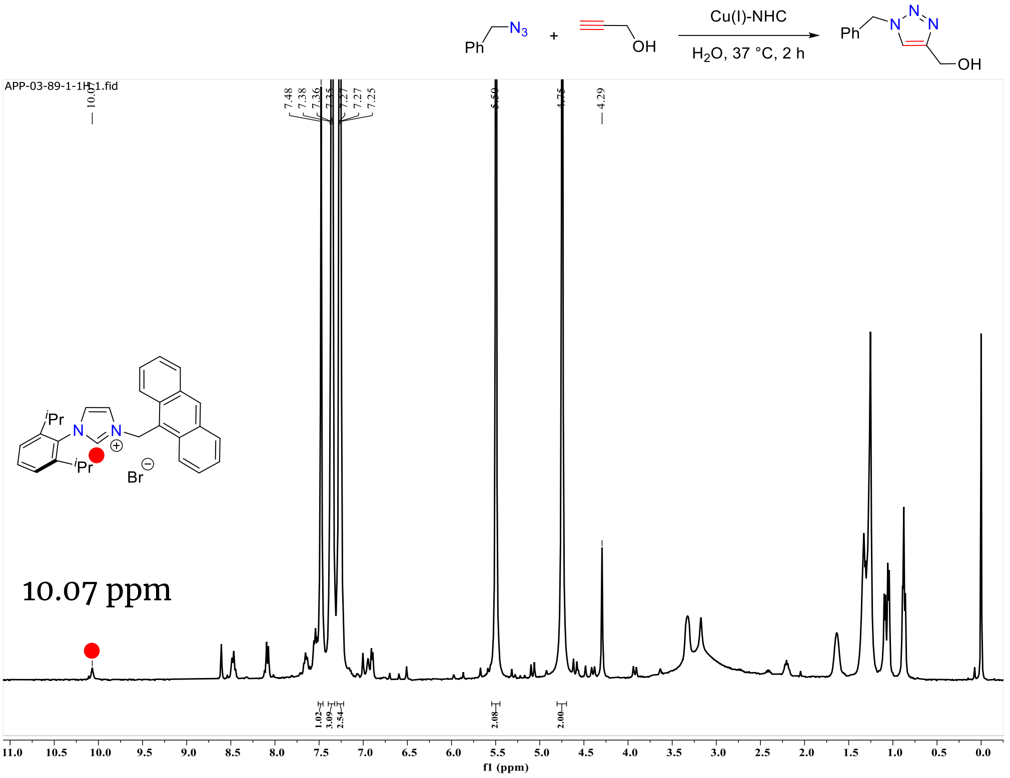

Figure S130. Crude ^{1}H NMR of CuAAC reaction showing the formation of NHC•HBr salt.

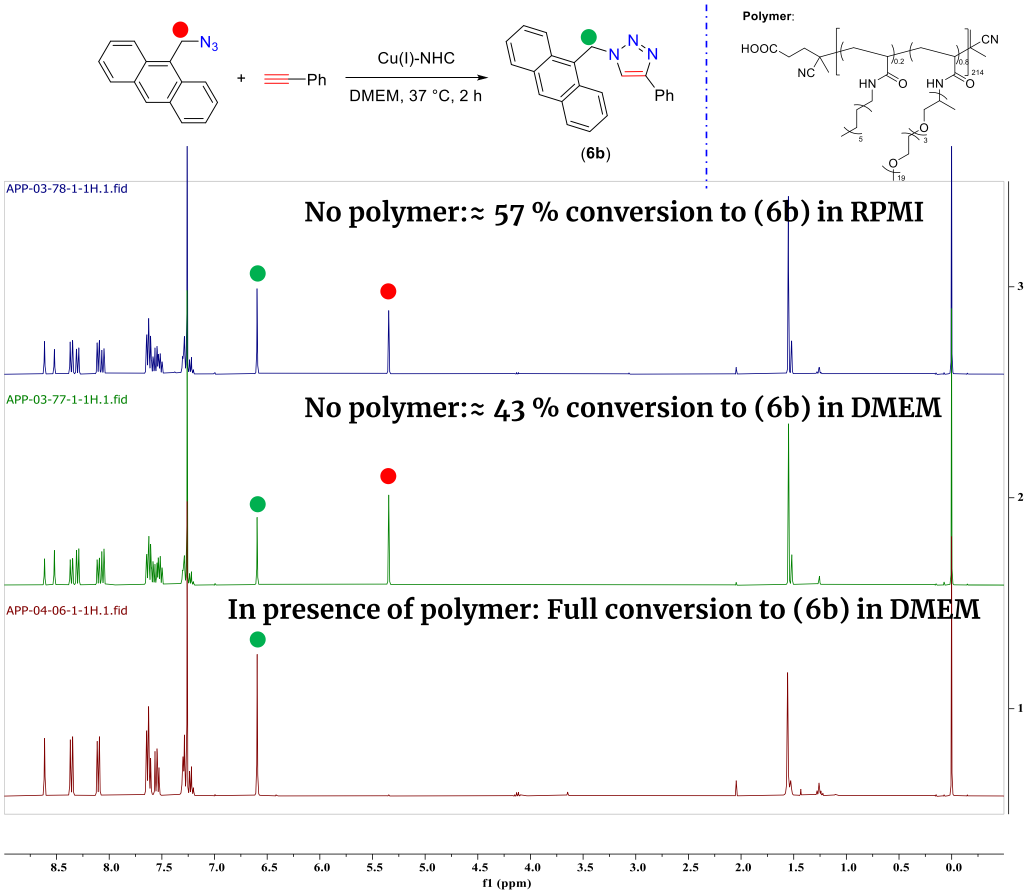

Figure S131. ^{1}H NMR (in $CDCl_3$); comparison of 1-(anthracen-9-ylmethyl)-4-phenyl-1H-1,2,3-triazole (**6b**) obtained from RPMI and DMEM media in absence/presence of amphiphilic polymer.

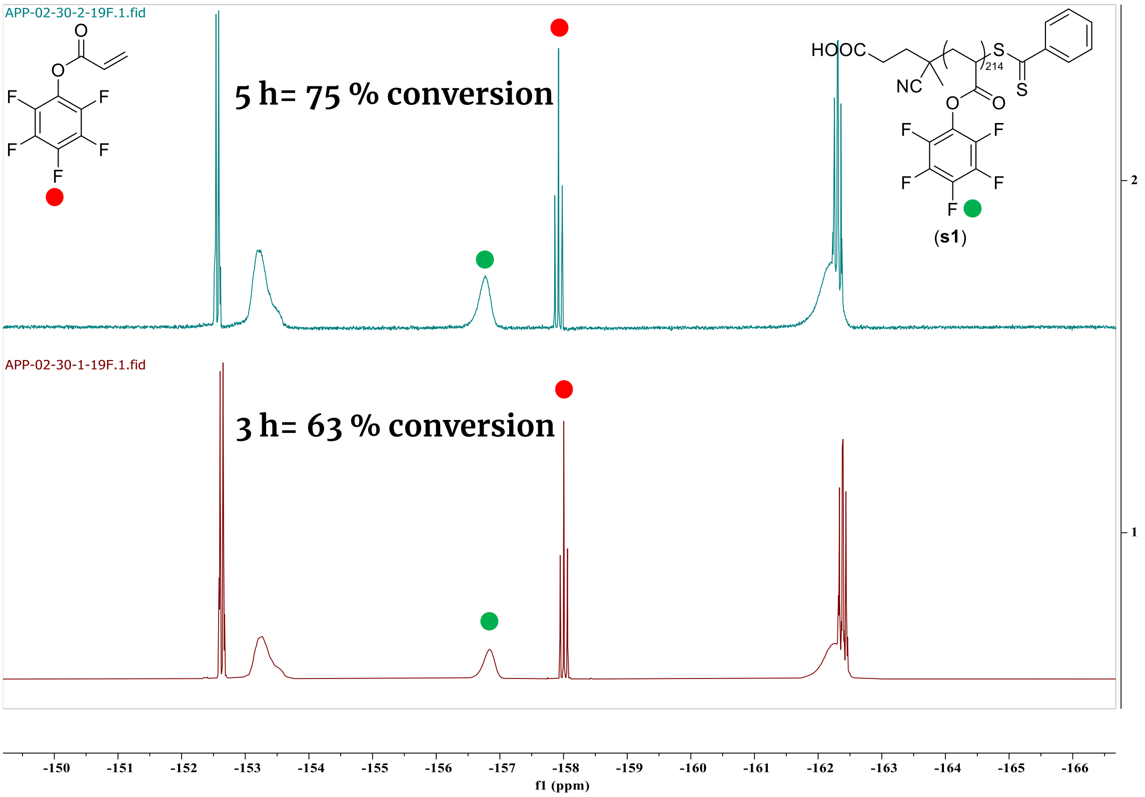

Figure S132: Monitoring (**s1**) polymer conversion by ^{19}F NMR spectrum in $CDCl_3$.

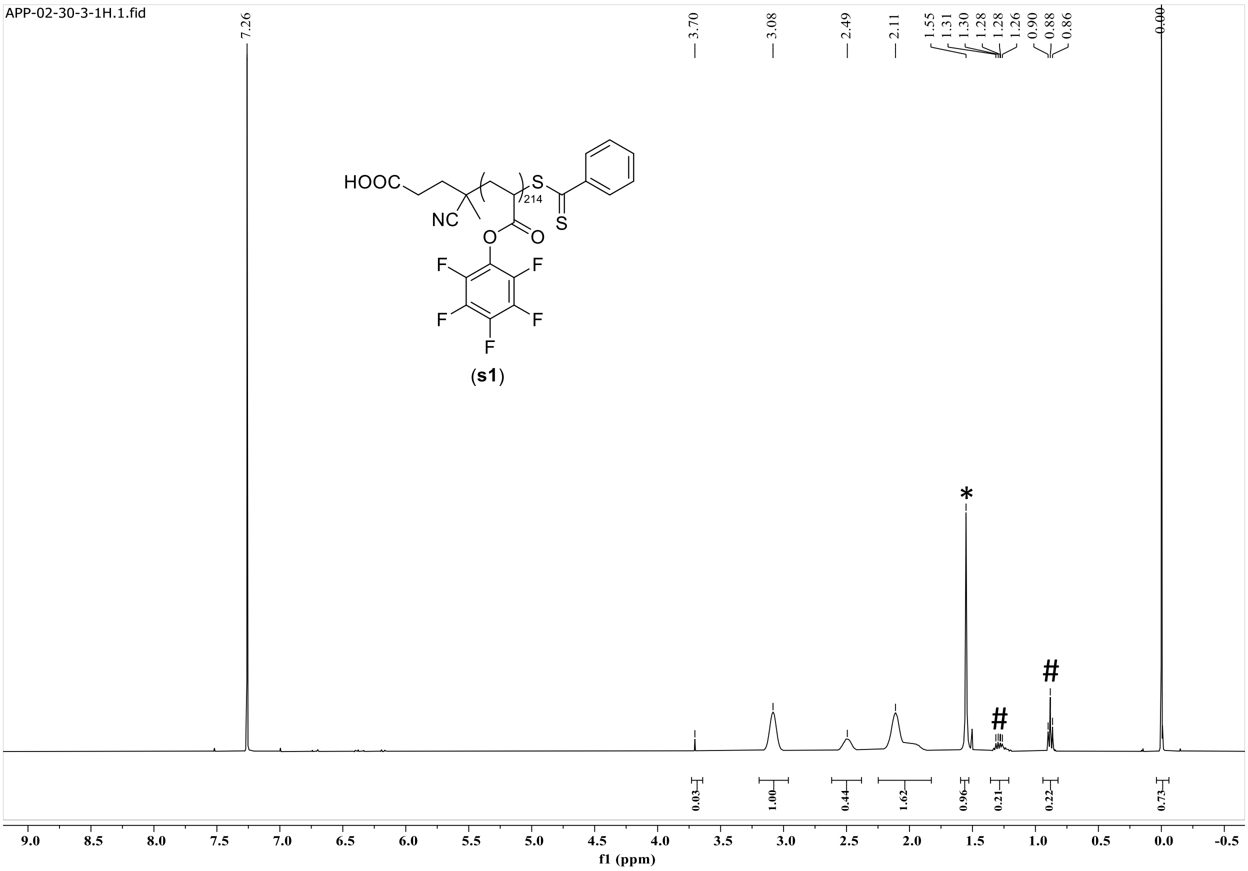

Figure S133: ^{1}H NMR of polymer (**s1**) in $CDCl_3$. # pentane, * H_2O.

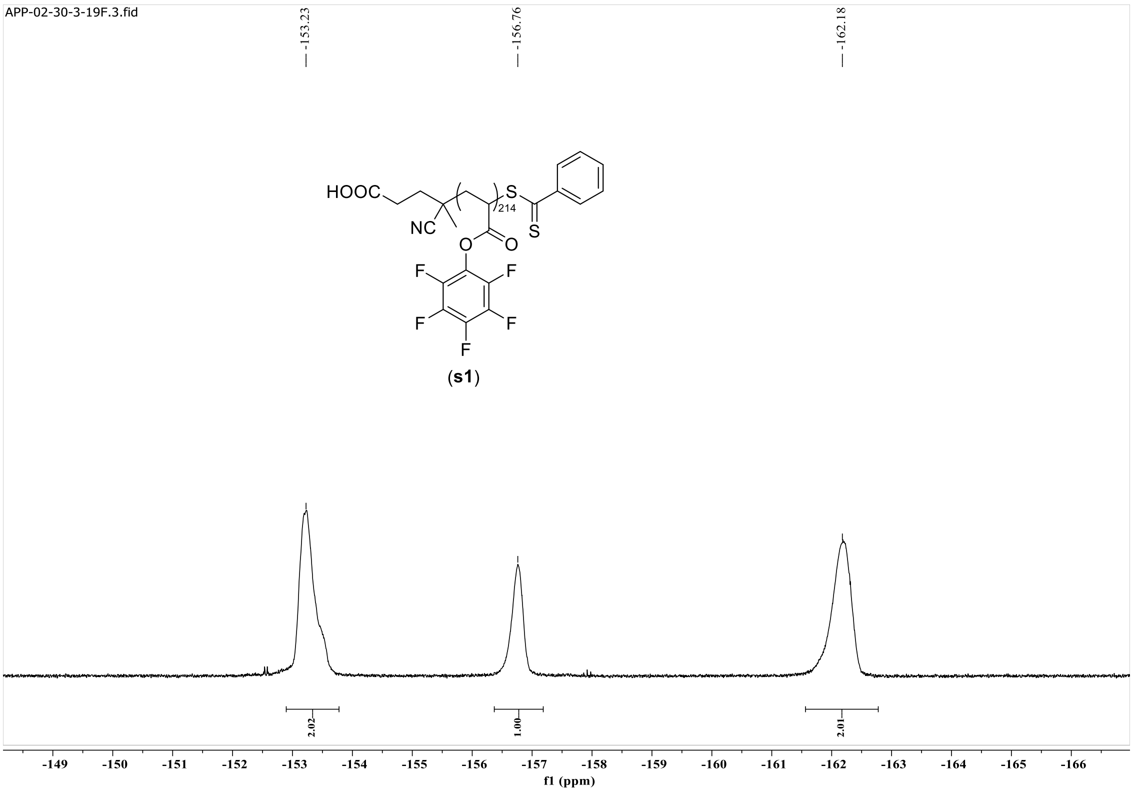

Figure S134: ^{19}F NMR of polymer (**s1**) in $CDCl_3$.

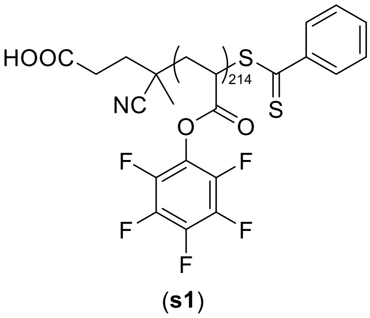

Sample Name	: APP-02-30-1
Tray#	: 1
Vail#	: 1
Injection Volume	: 10 uL
Data Filename	: APP-02-30-1.lcd
Method Filename	: Calibration_right_THF_Aug1023.lcm
Batch Filename	: THF-method.lcb
Report Filename	: DEFAULT.lsr
Date Acquired	: 8/10/2023 9:38:37 AM
Data Processed	: 8/10/2023 12:10:11 PM

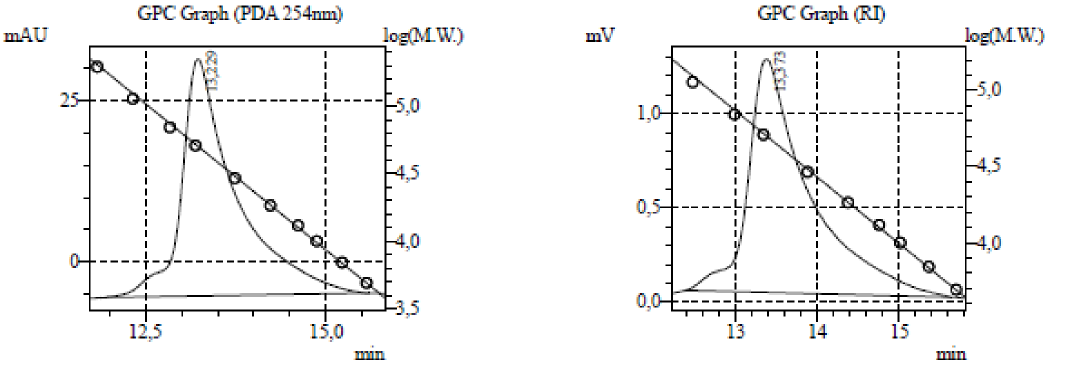

Total Average (PDA 254 nm)

Chromatogram PDA Ch1

#	Mn	Mw	Mz	Mw/Mn
1	32161	42625	52094	1,32537
	32161	42625	52094	1,32537

Total Average(RI)

Chromatogram Detector 2 Ch1

#	Mn	Mw	Mw/Mn
1	32012	41930	1,30981
	32012	41930	1,30981

Figure S135: SEC trace of polymer (**s1**) in THF.

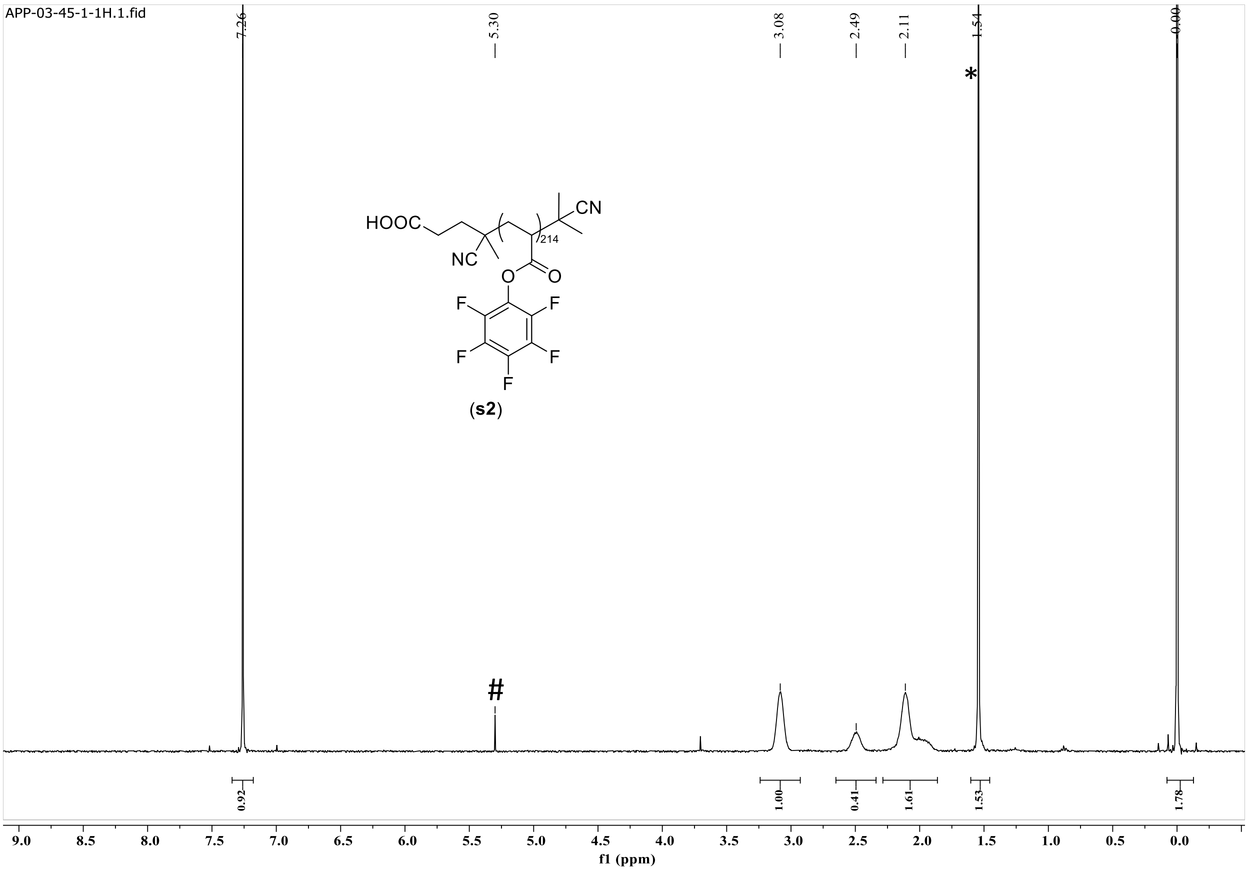

Figure S136: ^{1}H NMR of polymer (**s2**) in $CDCl_3$. # DCM, * H_2O.

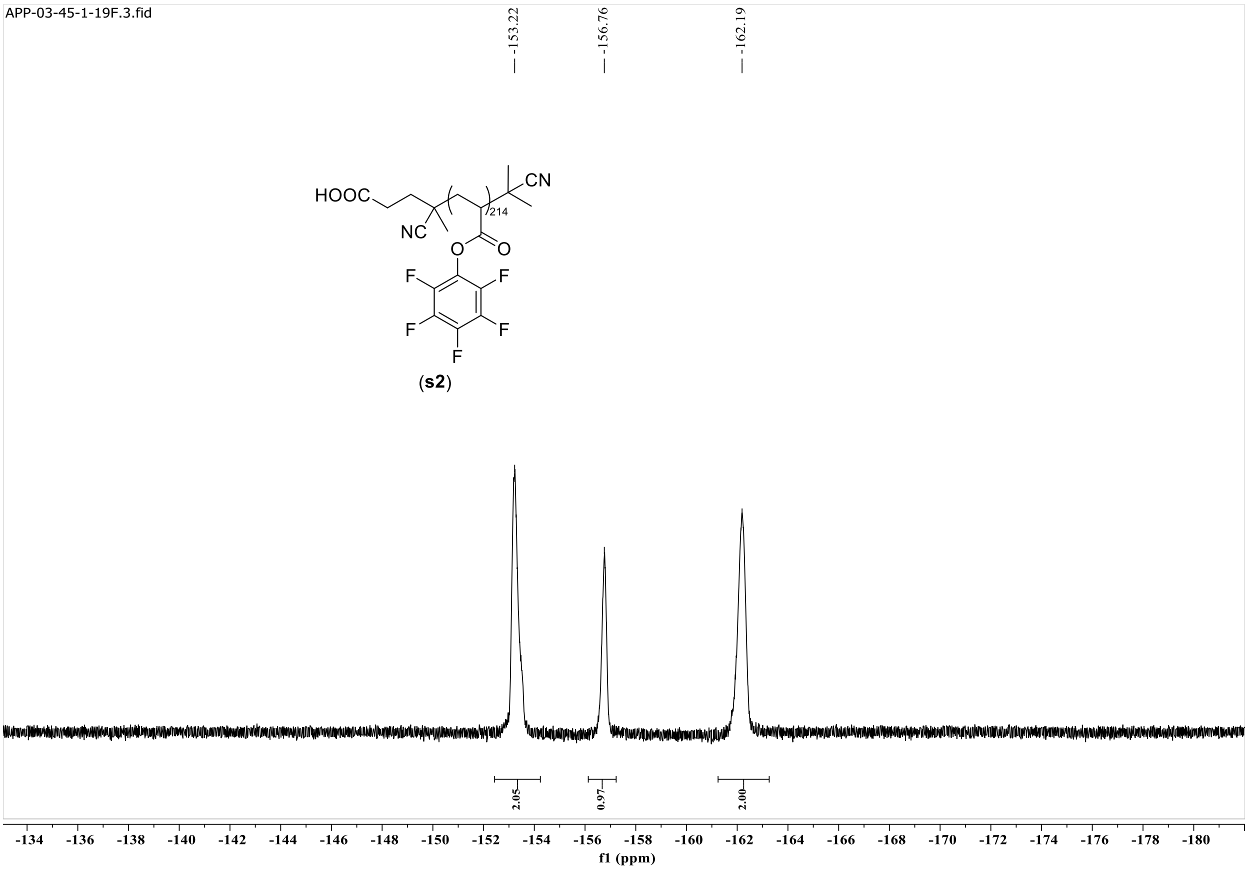

Figure S137: ^{19}F NMR of polymer (**s2**) in $CDCl_3$.

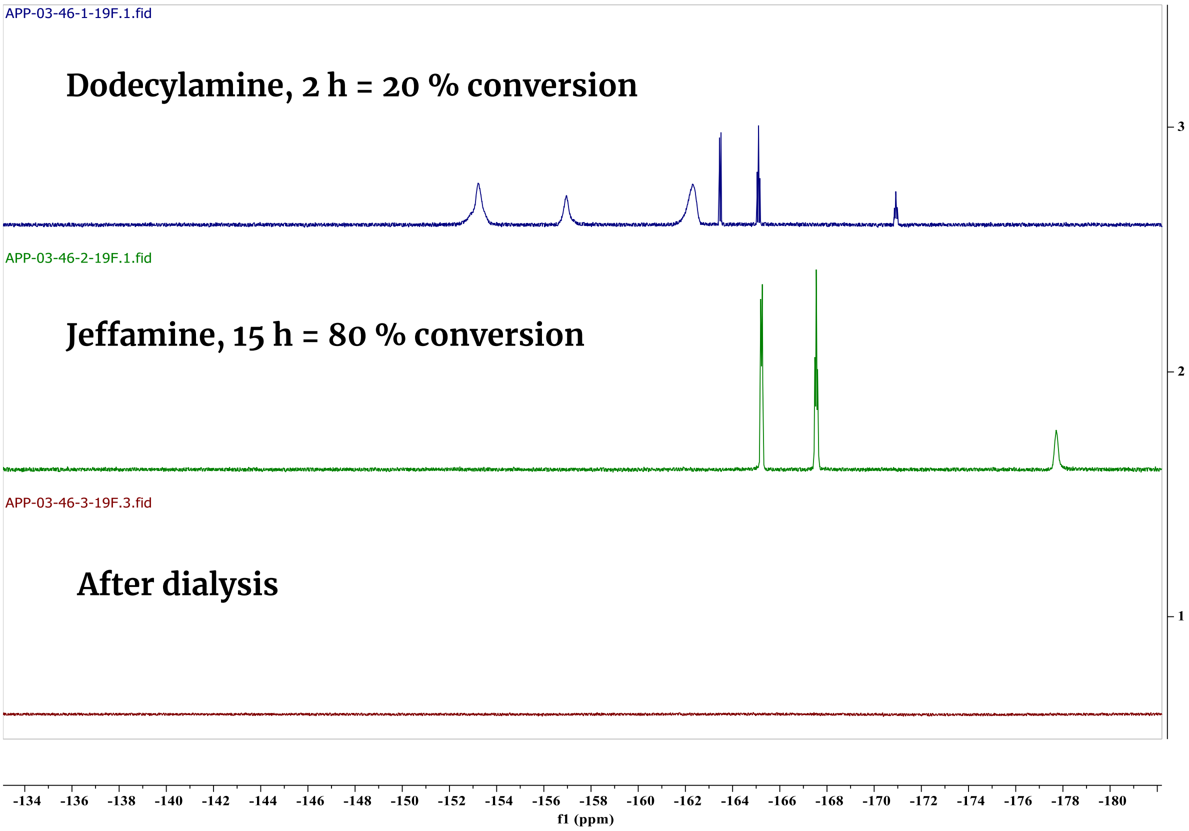

Figure S138: Monitoring the progress of the incorporation in (**8**) by ^{19}F NMR spectrum in $CDCl_3$.

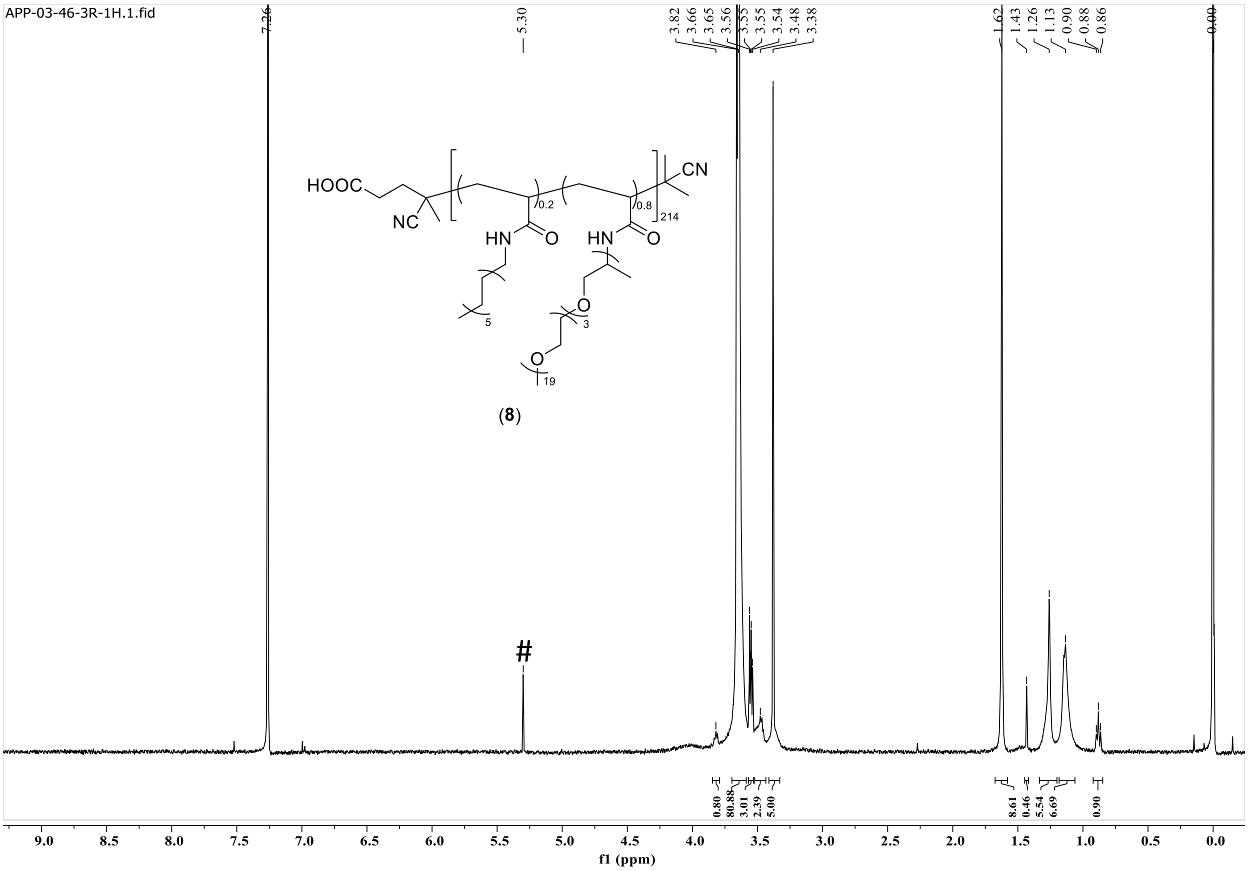

Figure S139: ^{1}H NMR of amphiphilic polymer (**8**) in $CDCl_3$. # DCM.

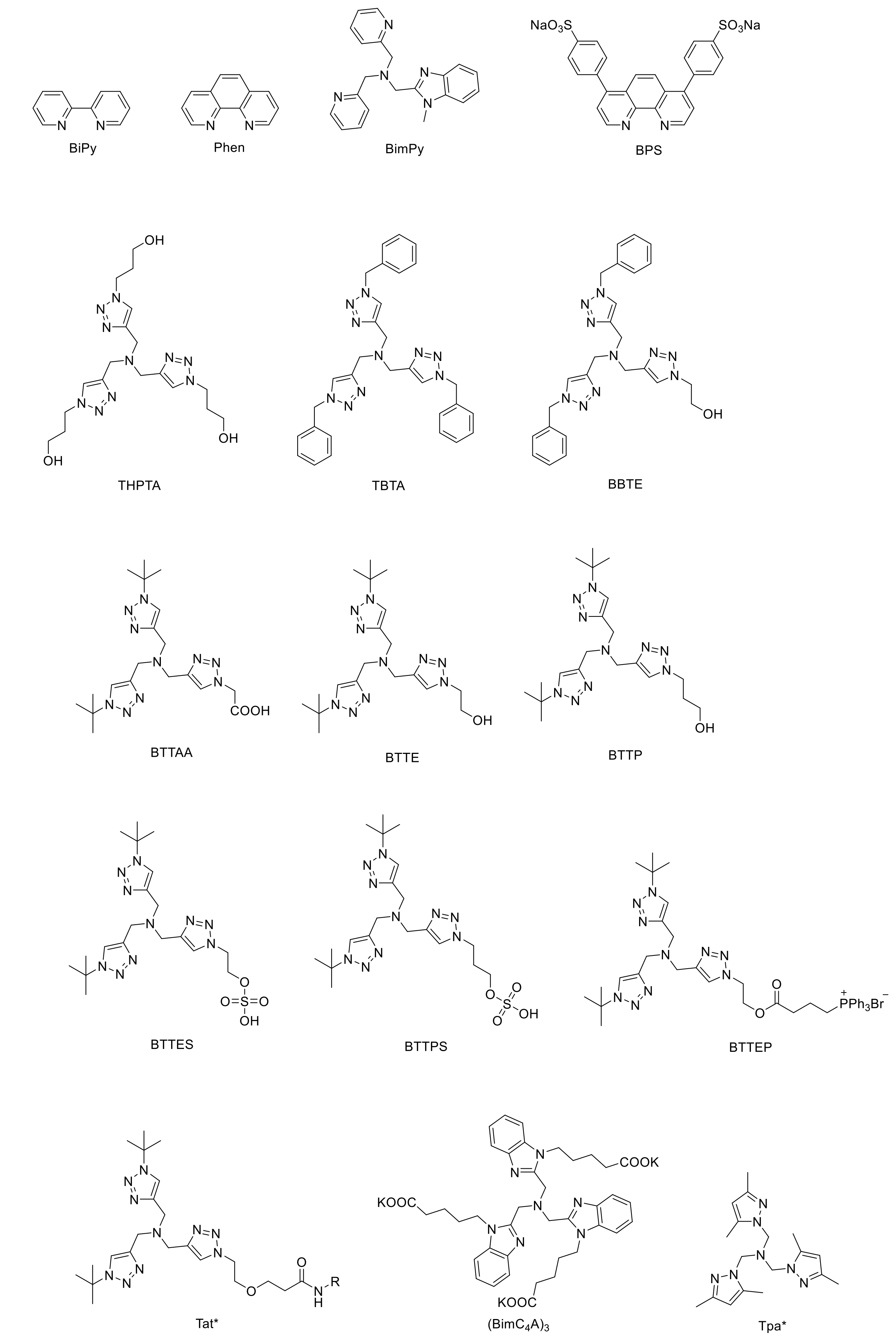

Figure S140: Various Cu(I) stabilizing ligands used in the literature.

4. References

1 T. P. Nicholls, Z. Jia and J. M. Chalker, *Chem. Eur. J.,* 2024, **30**, e202303161.

2 I. Carvalho, P. Andrade, V. L. Campo, P. M. M. Guedes, R. Sesti-Costa, J. S. Silva, S. Schenkman, S. Dedola, L. Hill, M. Rejzek, S. A. Nepogodiev and R. A. Field, *Bioorg. Med. Chem.*, 2010, **18**, 2412–2427.

3 M. B. Sims, J. J. Lessard, L. Bai and B. S. Sumerlin, *Macromolecules*, 2018, **51**, 6380–6386.

4 A. Sathyan, S. Croke, A. M. Pérez-López, B. F. M. de Waal, A. Unciti-Broceta and A. R. A. Palmans, *Mol. Syst. Des. Eng.*, 2022, **7**, 1736–1748.

5 M. Riethmann, S. A. Föhrenbacher, H. Keiling, N. V. Ignat'ev, M. Finze and U. Radius, *Inorg. Chem.*, 2024, **63**, 8351–8365.

6 A. Yagmurlu, N. Buğday, Ş. Yaşar, H. Boulebd, L. Mansour, W. S. Koko, N. Hamdi and S. Yaşar, *Appl. Organomet. Chem.*, 2024, **38**, e7545.

7 M. A. Topchiy, A. A. Ageshina, P. S. Gribanov, S. M. Masoud, T. R. Akmalov, S. E. Nefedov, S. N. Osipov, M. S. Nechaev and A. F. Asachenko, *Eur. J. Org. Chem.*, 2019, 1016–1020.

8 M. González-Lainez, M. Gallegos, J. Munarriz, R. Azpiroz, V. Passarelli, M. V. Jiménez and J. J. Pérez-Torrente, *Organometallics*, 2022, **41**, 2154–2169.

9 T. G. Brevé, M. Filius, C. Araman, M. P. van der Helm, P. Hagedoorn, C. Joo, S. I. van Kasteren and R. Eelkema, *Angew. Chem. Int. Ed.,* 2020, **59**, 9340–9344.

10 C. Gaulier, A. Hospital, B. Legeret, A. F. Delmas, V. Aucagne, F. Cisnetti and A. Gautier, *Chem. Commun.*, 2012, **48**, 4005.

11 A. Szadkowska, R. Pawłowski, E. Zaorska, S. Staszko, D. Trzybiński and K. Woźniak, *Appl. Organomet. Chem.*, 2019, **33**, e4983.

12 A. Islam Sk, A. Ghosh, K. Kundu, I. Murugan and P. K. Kundu, *Chem. Eur. J.,* 2024, **30**, e202402381.

13 K. S. Shinde, P. Michael, D. Fuhrmann and W. H. Binder, *Macromol. Chem. Phys.*, 2023, **224**, 2200207.

14 P. Michael, S. K. Sheidaee Mehr and W. H. Binder, *J. Polym. Sci. A Polym. Chem.*, 2017, **55**, 3893–3907.

15 S. Funtan, P. Michael and W. H. Binder, *Biomimetics*, 2019, **4**, 24.

16 C. Schotten, J. Manson, T. W. Chamberlain, R. A. Bourne, B. N. Nguyen, N. Kapur and C. E. Willans, *Catal. Sci. Technol.*, 2022, **12**, 4266–4272.

17 S. Díez-González and S. P. Nolan, *Angew. Chem. Int. Ed.,* 2008, **47**, 8881–8884.

18 S. Díez-González, A. Correa, L. Cavallo and S. P. Nolan, *Chem. Eur. J.,* 2006, **12**, 7558–7564.

19 L. T. Mitchell, E. Barnett, M. Hexom, A. Ruiz and A. Schoffstall, *Catalysts*, 2024, **14**, 702.

20 F. C. Pouget, J.-M. Andanson and A. Gautier, *RSC Sustain.*, 2023, **1**, 1826–1832.

21 A. Olivelli, C. Olelewe, L. G. Wolff, S. Parkin, C. Edwin Webster, S. G. Awuah and A. J. Huckaba, *Chem. Eur. J.,* 2024, **30**, e202402887.

22 M. van de L'Isle, S. Croke, T. Valero and A. Unciti-Broceta, *Chem. Eur. J.,* 2024, **30**, e202400611.

23 W. Wang, S. Hong, A. Tran, H. Jiang, R. Triano, Y. Liu, X. Chen and P. Wu, *Chem. Asian J.*, 2011, **6**, 2796–2802.

24 H. Jiang, T. Zheng, A. Lopez-Aguilar, L. Feng, F. Kopp, F. L. Marlow and P. Wu, *Bioconjug. Chem.*, 2014, **25**, 698–706.

25 C. Besanceney-Webler, H. Jiang, T. Zheng, L. Feng, D. Soriano del Amo, W. Wang, L. M. Klivansky, F. L. Marlow, Y. Liu and P. Wu, *Angew. Chem. Int. Ed.,* 2011, **50**, 8051–8056.

26 D. Soriano del Amo, W. Wang, H. Jiang, C. Besanceney, A. C. Yan, M. Levy, Y. Liu, F. L. Marlow and P. Wu, *J. Am. Chem. Soc.*, 2010, **132**, 16893–16899.

27 X. Wang, Y. Liu, X. Fan, J. Wang, W. S. C. Ngai, H. Zhang, J. Li, G. Zhang, J. Lin and P. R. Chen, *J. Am. Chem. Soc.*, 2019, **141**, 17133–17141.

28 F. Wang, Y. Zhang, Z. Liu, Z. Du, L. Zhang, J. Ren and X. Qu, *Angew. Chem. Int. Ed.,* 2019, **58**, 6987–6992.

29 S. Li, L. Wang, F. Yu, Z. Zhu, D. Shobaki, H. Chen, M. Wang, J. Wang, G. Qin, U. J. Erasquin, L. Ren, Y. Wang and C. Cai, *Chem. Sci.*, 2017, **8**, 2107–2114.

30 Y. Bai, X. Feng, H. Xing, Y. Xu, B. K. Kim, N. Baig, T. Zhou, A. A. Gewirth, Y. Lu, E. Oldfield and S. C. Zimmerman, *J. Am. Chem. Soc.*, 2016, **138**, 11077–11080.

31 D. C. Kennedy, C. S. McKay, M. C. B. Legault, D. C. Danielson, J. A. Blake, A. F. Pegoraro, A. Stolow, Z. Mester and J. P. Pezacki, *J. Am. Chem. Soc.*, 2011, **133**, 17993–18001.

32 J. Miguel-Ávila, M. Tomás-Gamasa, A. Olmos, P. J. Pérez and J. L. Mascareñas, *Chem. Sci.*, 2018, **9**, 1947–1952.

33 C. Uttamapinant, A. Tangpeerachaikul, S. Grecian, S. Clarke, U. Singh, P. Slade, K. R. Gee and A. Y. Ting, *Angew. Chem. Int. Ed.,* 2012, **51**, 5852–5856.

34 J. Huang, L. Wang, P. Zhao, F. Xiang, J. Liu and S. Zhang, *ACS Catal.*, 2018, **8**, 5941–5946.

35 Y. Liu, S. Pujals, P. J. M. Stals, T. Paulöhrl, S. I. Presolski, E. W. Meijer, L. Albertazzi and A. R. A. Palmans, *J. Am. Chem. Soc.*, 2018, **140**, 3423–3433.

36 J. Clavadetscher, S. Hoffmann, A. Lilienkampf, L. Mackay, R. M. Yusop, S. A. Rider, J. J. Mullins and M. Bradley, *Angew. Chem. Int. Ed.,* 2016, **55**, 15662–15666.

37 D. J. Stone, M. Macias-Contreras, S. M. Crist, C. F. T. Bucag, G. Seo and L. Zhu, *Org. Biomol. Chem.*, 2023, **21**, 7419–7436.

38 V. Bevilacqua, M. King, M. Chaumontet, M. Nothisen, S. Gabillet, D. Buisson, C. Puente, A. Wagner and F. Taran, *Angew. Chem. Int. Ed.*, 2014, **53**, 5872–5876.

39 A. Sathyan, T. Loman, L. Deng and A. R. A. Palmans, *Nanoscale*, 2023, **15**, 12710–12717.

40 P. N. Liu, J. Li, F. H. Su, K. D. Ju, L. Zhang, C. Shi, H. H. Y. Sung, I. D. Williams, V. V. Fokin, Z. Lin and G. Jia, *Organometallics*, 2012, **31**, 4904–4915.

41 X. Meng, X. Xu, T. Gao and B. Chen, *Eur. J. Org. Chem.*, 2010, 5409–5414.

42 S. Saha, M. Kaur and J. K. Bera, *Organometallics*, 2015, **34**, 3047–3054.

43 G. A. Rance, W. A. Solomonsz and A. N. Khlobystov, *Chem. Commun.*, 2013, **49**, 1067.

44 Y. He, Z. Bian, C. Kang, Y. Cheng and L. Gao, *Chem. Commun.*, 2010, **46**, 3532.

45 A. Szadkowska, S. Staszko, E. Zaorska and R. Pawłowski, *RSC Adv.*, 2016, **6**, 44248–44253.

46 J. Wen, K. Wu, D. Yang, J. Tian, Z. Huang, A. S. Filatov, A. Lei and X.-M. Lin, *ACS Appl. Mater. Interfaces*, 2018, **10**, 25930–25935.

47 T. S. Rodrigues, A. G. M. da Silva, L. C. de Oliveira, A. M. da Silva, R. R. Teixeira and P. H. C. Camargo, *Tetrahedron Lett.*, 2017, **58**, 590–595.

48 S. Ø. Scott, E. L. Gavey, S. J. Lind, K. C. Gordon and J. D. Crowley, *Dalton Trans.*, 2011, **40**, 12117.

49 S. Saha, M. Kaur and J. K. Bera, *Organometallics*, 2015, **34**, 3047–3054.

50 L. Bahsis, M. El Himri, H. Ben El Ayouchia, H. Anane, E. Ablouh, M. Julve and S. Stiriba, *Macromol. Chem. Phys.*, 2019, **220**, 1900311.

51 J. H. Kim and S. Kim, *RSC Adv.*, 2014, **4**, 26516.

52 F. Cuevas, A. Oliva and M. Pericàs, *Synlett*, 2010, 1873–1877.

53 G. A. Chesnokov, M. A. Topchiy, P. B. Dzhevakov, P. S. Gribanov, A. A. Tukov, V. N. Khrustalev, A. F. Asachenko and M. S. Nechaev, *Dalton Trans.*, 2017, **46**, 4331–4345.

54 B. Mohan, H. Kang and K. H. Park, *Inorg. Chem. Commun.*, 2013, **35**, 239–241.

55 R. Thompson, K. Damodaran, D. Luebke and H. Nulwala, *Synlett*, 2013, **24**, 1093–1096.

56 O. S. Taskin, S. Dadashi-Silab, B. Kiskan, J. Weber and Y. Yagci, *Macromol. Chem. Phys.*, 2015, **216**, 1746–1753.

57 A. Tripathi, C. V. Rode, J. Llop, S. P. Chavan and S. M. Joshi, *Tetrahedron Lett.*, 2020, **61**, 151662.
